# Supplementary material for: Reassessment of the Early Cretaceous North American “troodontid” Geminiraptor suarezarum as a therizinosaurian and mosaic evolution of coelurosaurian maxillae
Source: PLoS One. 2026 Sep 30;21(9):e0356471. doi: 10.1371/journal.pone.0356471 (PMC13626363; doi:10.1371/journal.pone.0356471)
Supplement: Supplementary file 1 [file pone.0356471.s004.pdf]

PLOS One

Reassessment of the Early Cretaceous North American “troodontid” Geminiraptor suarezarum as a therizinosaurian and mosaic evolution of coelurosaurian maxillae

--Manuscript Draft--

**Manuscript Number:**	PONE-D-26-07743
**Article Type:**	Research Article
**Full Title:**	Reassessment of the Early Cretaceous North American “troodontid” Geminiraptor suarezarum as a therizinosaurian and mosaic evolution of coelurosaurian maxillae
**Short Title:**	Reassessment of <i>Geminiraptor</i> as a therizinosaurian
**Corresponding Author:**	William Freimuth<br>North Carolina State University<br>Raleigh, NC UNITED STATES OF AMERICA
**Keywords:**	theropod; Maniraptora; dinosaur; Cedar Mountain Formation; herbivory; morphology; taxonomy; phylogenetics; morphometrics
**Abstract:**	The only recorded member of the bird-like Troodontidae from Early Cretaceous North America is Geminiraptor suarezarum, known from an isolated maxilla recovered from the Suarez Site bonebed of the basal Cedar Mountain Formation (Utah, USA). Geminiraptor would be one of the geologically oldest and largest-bodied troodontids on the continent and a key taxon for understanding evolutionary, ecological, and biogeographic patterns in this clade. However, the association of the holotype specimen with a monodominant therizinosaurian bonebed, a poor understanding of early therizinosaurian crania, and a host of newly available comparative material since its original description, call for a reassessment of this important specimen. Here we redescribe the holotype maxilla of Geminiraptor and other cranial bones from the Suarez Site bonebed using updated anatomical, morphometric, and phylogenetic datasets. Several features of the holotype maxilla and other cranial bones (including a frontal, postorbitals, a quadrate, dentaries, a possible lacrimal, and a fragmentary braincase) are now known to characterize early diverging therizinosaurians or are more broadly distributed within Theropoda. Computed tomography data reveal previously unrecognized replacement teeth in the maxilla with a stout, lanceolate, labially convex morphology, a prominent lingual median ridge and basal swelling, and minute denticles. These features are consistent with the dentition of early therizinosaurians (e.g., Falcarius, Jianchangosaurus, Beipiaosaurus) instead of troodontids. Morphometric analyses of coelurosaurian maxillae demonstrate substantial overlap in morphospace between early therizinosaurians, troodontids, and Geminiraptor. Phylogenetic analyses recover the specimen as the sister taxon to Falcarius utahensis at the base of Therizinosauria. Accordingly, we reidentify “Geminiraptor” as a therizinosaurian and establish a novel combination, Falcarius suarezarum. This introduces a fourth therizinosaurian to the earliest Cretaceous of North America and restricts definitive, large-bodied troodontids to the Late Cretaceous. Shared maxillary proportions of early coelurosaurians may reflect morphological constraints and a non-carnivorous diet, converged upon by late-branching troodontids.
**Order of Authors:**	William J. Freimuth
	Joshua R. Lively
	Lindsay E. Zanno
**Additional Information:**	
**Question**	**Response**
**Financial Disclosure**<br><br>Enter a financial disclosure statement that describes the sources of funding for the work included in this submission. Review the submission guidelines for detailed	Yes

Powered by Editorial Manager® and ProduXion Manager® from Aries Systems Corporation

requirements. View published research articles from *PLOS ONE* for specific examples.<br><br>This statement is required for submission and **will appear in the published article** if the submission is accepted. Please make sure it is accurate.<br><br>**Funded studies**<br>Enter a statement with the following details:<br>• Initials of the authors who received each award<br>• Grant numbers awarded to each author<br>• The full name of each funder<br>• URL of each funder website<br>• Did the sponsors or funders play any role in the study design, data collection and analysis, decision to publish, or preparation of the manuscript?<br><br>Did you receive funding for this work?	
Please add funding details.<br>as follow-up to "**Financial Disclosure**<br><br>Enter a financial disclosure statement that describes the sources of funding for the work included in this submission. Review the submission guidelines for detailed requirements. View published research articles from *PLOS ONE* for specific examples.<br><br>This statement is required for submission and **will appear in the published article** if the submission is accepted. Please make sure it is accurate.	Funding was provided by the Paleontological Society (https://www.paleosoc.org/), the Jurassic Foundation (https://www.jurassicfoundation.org/), the Paleontological Research Institution (https://www.priweb.org/), and the Systematics Association (https://systass.org/) to WJF, and National Science Foundation (https://www.nsf.gov/) award #1925973 to LEZ.

Powered by Editorial Manager® and ProduXion Manager® from Aries Systems Corporation

**Funded studies**<br>Enter a statement with the following details:<br>• Initials of the authors who received each award<br>• Grant numbers awarded to each author<br>• The full name of each funder<br>• URL of each funder website<br>• Did the sponsors or funders play any role in the study design, data collection and analysis, decision to publish, or preparation of the manuscript?<br><br>Did you receive funding for this work?"	
Please select the country of your main research funder (please select carefully as in some cases this is used in fee calculation).<br>as follow-up to "**Financial Disclosure**<br><br>Enter a financial disclosure statement that describes the sources of funding for the work included in this submission. Review the submission guidelines for detailed requirements. View published research articles from *PLOS ONE* for specific examples.<br><br>This statement is required for submission and **will appear in the published article** if the submission is accepted. Please make sure it is accurate.<br><br>**Funded studies**<br>Enter a statement with the following details:<br>• Initials of the authors who received each award<br>• Grant numbers awarded to each author<br>• The full name of each funder<br>• URL of each funder website<br>• Did the sponsors or funders play any role in the study design, data collection and analysis, decision to publish, or preparation of the manuscript?<br><br>Did you receive funding for this work?"	UNITED STATES - US
**Competing Interests**	The authors have declared that no competing interests exist.

Use the instructions below to enter a competing interest statement for this submission. On behalf of all authors, disclose any competing interests that could be perceived to bias this work—acknowledging all financial support and any other relevant financial or non-financial competing interests.<br><br>This statement is required for submission and **will appear in the published article** if the submission is accepted. Please make sure it is accurate and that any funding sources listed in your Funding Information later in the submission form are also declared in your Financial Disclosure statement.<br><br>View published research articles from *PLOS ONE* for specific examples.<br><br>**NO authors have competing interests**<br><br>Enter: *The authors have declared that no competing interests exist.*<br><br>**Authors with competing interests**<br><br>Enter competing interest details beginning with this statement:<br><br>*I have read the journal's policy and the authors of this manuscript have the following competing interests: [insert competing interests here]*<br><br>* typeset	
**Ethics Statement**<br><br>Enter an ethics statement for this submission. This statement is required if the study involved:<br><br>• Human participants<br>• Human specimens or tissue<br>• Vertebrate animals or cephalopods<br>• Vertebrate embryos or tissues	All specimens collected from the Suarez Site (Prehistoric Museum [CEUM] site Gr 286v) were excavated under permits issued by the Bureau of Land Management to the Prehistoric Museum (UT06-0038E and UT11-009E, respectively).

Powered by Editorial Manager® and ProduXion Manager® from Aries Systems Corporation

• Field research<br><br>Write "N/A" if the submission does not require an ethics statement.<br><br>General guidance is provided below. Consult the submission guidelines for detailed instructions. **Make sure that all information entered here is included in the Methods section of the manuscript.**	

**Format for specific study types**<br><br>**Human Subject Research (involving human participants and/or tissue)**<br>• Give the name of the institutional review board or ethics committee that approved the study<br>• Include the approval number and/or a statement indicating approval of this research<br>• Indicate the form of consent obtained (written/oral) or the reason that consent was not obtained (e.g. the data were analyzed anonymously)<br><br>**Animal Research (involving vertebrate animals, embryos or tissues)**<br>• Provide the name of the Institutional Animal Care and Use Committee (IACUC) or other relevant ethics board that reviewed the study protocol, and indicate whether they approved this research or granted a formal waiver of ethical approval<br>• Include an approval number if one was obtained<br>• If the study involved *non-human primates*, add *additional details* about animal welfare and steps taken to ameliorate suffering<br>• If anesthesia, euthanasia, or any kind of animal sacrifice is part of the study, include briefly which substances and/or methods were applied<br><br>**Field Research**<br><br>Include the following details if this study involves the collection of plant, animal, or other materials from a natural setting:<br>• Field permit number<br>• Name of the institution or relevant body that granted permission	
**Data Availability**<br><br>Authors are required to make all data underlying the findings described fully available, without restriction, and from the time of publication. PLOS allows rare exceptions to address legal and ethical concerns. See the PLOS Data Policy and FAQ for detailed information.	Yes - all data are fully available without restriction

A Data Availability Statement describing where the data can be found is required at submission. Your answers to this question constitute the Data Availability Statement and **will be published in the article**, if accepted.<br><br>**Important:** Stating 'data available on request from the author' is not sufficient. If your data are only available upon request, select 'No' for the first question and explain your exceptional situation in the text box.<br><br>Do the authors confirm that all data underlying the findings described in their manuscript are fully available without restriction?	
**Describe where the data may be found in full sentences. If you are copying our sample text, replace any instances of XXX with the appropriate details.**<br><br>• If the data are **held or will be held in a public repository**, include URLs, accession numbers or DOIs. If this information will only be available after acceptance, indicate this by ticking the box below. For example: *All XXX files are available from the XXX database (accession number(s) XXX, XXX.).*<br>• If the data are all contained **within the manuscript and/or Supporting Information files**, enter the following: *All relevant data are within the manuscript and its Supporting Information files.*<br>• If neither of these applies but you are able to provide **details of access elsewhere**, with or without limitations, please do so. For example:<br><br>*Data cannot be shared publicly because of [XXX]. Data are available from the XXX Institutional Data Access / Ethics Committee (contact via XXX) for researchers who meet the criteria for access to confidential data.*<br><br>*The data underlying the results presented in the study are available from (include the name of the third party*	All 3D models and TIF stacks are available from Morphosource projects XXX and XXX. All data underlying the results of this study are within the manuscript and its Supporting Information files.

Powered by Editorial Manager® and ProduXion Manager® from Aries Systems Corporation

and contact information or URL).<br>• This text is appropriate if the data are owned by a third party and authors do not have permission to share the data.<br><br>* typeset	
Additional data availability information:	Tick here if the URLs/accession numbers/DOIs will be available only after acceptance of the manuscript for publication so that we can ensure their inclusion before publication.

Powered by Editorial Manager® and ProduXion Manager® from Aries Systems Corporation

Manuscript

Click here to access/download;Manuscript;Freimuth_et_al_Geminiraptor_sub1

Reassessment of the Early Cretaceous North American "troodontid" *Geminiraptor suarezarum* as a therizinosaurian and mosaic evolution of coelurosaurian maxillae

William J. Freimuth[1,2*], Joshua R. Lively[3,4], Lindsay E. Zanno[1,2,5–9]

[1] Paleontology, North Carolina Museum of Natural Sciences, Raleigh, North Carolina, USA

[2] Department of Biological Sciences, North Carolina State University, Raleigh, North Carolina, USA

[3] Prehistoric Museum, Utah State University Eastern, Price, Utah, USA

[4] Department of Geosciences, Utah State University, Logan, Utah, USA

[5] Department of Geology, Field Museum of Natural History, Chicago, IL, USA

[6] Paleontology, Natural History Museum of Utah, Salt Lake City, UT, USA

[7] Vertebrate Paleontology, Sam Noble Museum, Norman, OK, USA

[8] Department of Earth Sciences, Stellenbosch University, Stellenbosch, South Africa

[9] Evolutionary Studies Institute, University of Witwatersrand, Johannesburg, South Africa

*Corresponding author

E-mail: willie.j.freimuth@gmail.com

Abstract

The only recorded member of the bird-like Troodontidae from Early Cretaceous North America is *Geminiraptor suarezarum*, known from an isolated maxilla recovered from the Suarez Site bonebed of the basal Cedar Mountain Formation (Utah, USA). *Geminiraptor* would be one of the geologically oldest and largest-bodied troodontids on the continent and a key taxon for understanding evolutionary, ecological, and biogeographic patterns in this clade. However, the association of the holotype specimen with a monodominant therizinosaurian bonebed, a poor understanding of early therizinosaurian crania, and a host of newly available comparative material since its original description, call for a reassessment of this important specimen. Here we redescribe the holotype maxilla of *Geminiraptor* and other cranial bones from the Suarez Site bonebed using updated anatomical, morphometric, and phylogenetic datasets. Several features of the holotype maxilla and other cranial bones (including a frontal, postorbitals, a quadrate, dentaries, a possible lacrimal, and a fragmentary braincase) are now known to characterize early diverging therizinosaurians or are more broadly distributed within Theropoda. Computed tomography data reveal previously unrecognized replacement teeth in the maxilla with a stout, lanceolate, labially convex morphology, a prominent lingual median ridge and basal swelling, and minute denticles. These features are consistent with the dentition of early therizinosaurians (e.g., *Falcarius, Jianchangosaurus, Beipiaosaurus*) instead of troodontids. Morphometric analyses of coelurosaurian maxillae demonstrate substantial overlap in morphospace between early therizinosaurians, troodontids, and *Geminiraptor*. Phylogenetic analyses recover the specimen as the sister taxon to *Falcarius utahensis* at the base of Therizinosauria. Accordingly, we reidentify "*Geminiraptor*" as a therizinosaurian and establish a novel combination, *Falcarius suarezarum*. This introduces a fourth therizinosaurian to the earliest Cretaceous of North

America and restricts definitive, large-bodied troodontids to the Late Cretaceous. Shared maxillary proportions of early coelurosaurians may reflect morphological constraints and a non-carnivorous diet, converged upon by late-branching troodontids.

Introduction

Mosaic evolution describes evolutionary processes that result in a mixture of ancestral and derived features [1–3]. First used to describe the combination of reptilian and avian features of *Archaeopteryx* [1], mosaicism is now known to characterize many aspects in the evolution of avian and non-avian theropod dinosaurs ("theropods" hereafter; [4–6]). Examples of mosaicism in theropod evolution include the presence and distribution of postcranial pneumaticity [7,8], pygostyle-like structures [4], edentulous rhamphothecae [9], and several flight-related morphological features [6,10,11], among others. This mosaicism can potentially complicate the taxonomic identification and phylogenetic placement of certain taxa, particularly those known from fragmentary or isolated remains [12].

One such example of a fragmentary yet important coelurosaurian specimen is *Geminiraptor suarezarum* from the lower Yellow Cat Member of the Cedar Mountain Formation in Utah, USA. Originally described based on an incomplete left maxilla, this specimen was diagnosed as the earliest known North American member of the bird-like clade Troodontidae [13]. Troodontids have close affinities to birds in several morphological [14], behavioral [15,16], and reproductive traits [17–19], occasionally forming the sister clade to avialans [20,21] or grouped with dromaeosaurids in Deinonychosauria [22,23] as the avialan sister clade [11,24]. The fossil record of troodontids is best known from the Campanian–Maastrichtian deposits of North America [14,25–30] and Asia [31–38], and the Early Cretaceous strata of China,

Mongolia, and Japan [15,16,39–46]. Importantly, putative troodontids are also known from the Jurassic of North America, including a partial skeleton designated *Hesperornithoides miessleri* [47] and a tooth taxon, *Koparion douglassi* [48]. Late Cretaceous troodontids are generally larger-bodied (up to ~60 kg; [29]) and tend to possess teeth with characteristically large denticles (e.g., [14]) compared to their small-bodied, mostly adenticulate Early Cretaceous and possible Jurassic counterparts. Among troodontids, *Geminiraptor* is considered one of the most important specimens because it serves as the sole Early Cretaceous representative from North America for comparison with better-represented Asian taxa from this time period [49]. Moreover, the presence of a proportionally large-bodied troodontid in the Early Cretaceous of North America has important implications for body-size evolution and biogeographic patterns of the clade.

Despite the importance of *Geminiraptor* in understanding troodontid evolution, several aspects of its taxonomic assignment warrant reassessment. The holotype maxilla is the only specimen referred to Troodontidae from the Suarez Site, a monodominant bonebed with abundant skeletal remains attributed to an early therizinosaurian [13]. The taphonomy and near-monotaxic composition of the Suarez Site are similar to the nearby Crystal Geyser Quarry, a mass-death assemblage of the early therizinosaurian *Falcarius utahensis* [50–53]. The original fossil materials ascribed to *Fa. utahensis* lacked a well-preserved maxilla [50,53], and the only representative therizinosaurian skull described at the time was *Erlikosaurus andrewsi* [54,55], which exhibits a radically derived morphology compared to *Geminiraptor*. Subsequent descriptions of early diverging therizinosaurians from China either lacked appropriate comparative materials [56,57] or yielded conflicting interpretations of salient anatomical features [58,59]. However, recent discoveries of new maxillae for *Fa. utahensis* demonstrate convergent features with troodontids and exhibit a striking similarity to the maxilla of *Geminiraptor* [60].

Moreover, a host of newly described troodontid specimens [30,33,34,37,42,43,61] since the initial description of *Geminiraptor* provide an updated comparative framework to assess the taxonomic status of this important holotype specimen.

Here, we redescribe and rediagnose the holotype maxilla of *Geminiraptor suarezarum* alongside other theropod cranial remains from the Suarez Site using computed tomography (CT) imaging and an updated comparative dataset. We additionally test the taxonomic affinities of the holotype specimen using taxon-rich morphometric and phylogenetic datasets. We discuss the implications of our results on the broader ecological, evolutionary, and biogeographical patterns of troodontids and the earliest Cretaceous theropod faunas in North America represented in the lower Yellow Cat Member.

Geological setting

The holotype specimen of *Geminiraptor suarezarum* is from the Suarez Site (CEUM site Gr 286v), a monodominant therizinosaurian bonebed from the lower Yellow Cat Member of the Cedar Mountain Formation in eastern Utah, USA (Fig 1). The site occurs ~800 meters northwest of the holotype locality of *Falcarius utahensis* at the Crystal Geyser Quarry (**CGQ**; [50–53]), and ~200 meters north of where the holotype specimen of *Martharaptor greenriverensis* [62] was recovered. In this area, the lower Yellow Cat Member unconformably overlies the Jurassic Morrison Formation and is only 2–3 m thick. The division between the lower and upper Yellow Cat Members is demarcated by a regional marker, the calcrete "caprock," thought to represent the onset of a rain shadow and arid conditions associated with the Sevier Orogeny [63–65]. Stratigraphically, the Suarez Site bonebed resides ~20–30 cm below this caprock unit and ~1.2 m

above the unconformable contact with the Morrison Formation, whereas the CGQ bonebed occurs ~1 m below the caprock and sits directly on top of the Morrison contact (Fig 1).

Fig 1. Stratigraphic sections of the Crystal Geyser Quarry (A) and the Suarez Site (B). Composite Crystal Geyser Quarry section adapted from Quarry Profile 1 of C. Suarez et al. [51] and M. Suarez et al. [52].

Age constraints on the Yellow Cat Member are complicated by a lack of dateable ash beds, low deposition rates, reworking of underlying Jurassic-age sediments, and regional salt-tectonic processes [64–67]. Mori [68] reported ages derived from detrital zircons from a sandstone unit just stratigraphically above the CGQ bonebed at 121.8 ± 2.2 Ma, and from just above the Suarez Site at 122.6 ± 0.7 Ma (Barremian–Aptian); however, low sample sizes and stratigraphic inconsistencies with the currently accepted framework cast doubt on the veracity of these age data [65]. An age of 135.1 ± 0.3 Ma was recently reported from strata east of our study area, a few meters stratigraphically above the regional caprock [67,69], which implies CGQ and the Suarez Site are Berriasian–Valanginian in age. Because of the "time-rich" nature of the lower Yellow Cat Member, reworking of older materials [67], and a lack of reliable dates from the present study area, assigning a more precise age constraint on the Suarez Site and CGQ beyond a possible Berriasian–Aptian range is difficult. Given the stratigraphic and lithological differences between the two bonebeds (see below), it can be inferred that the Suarez Site is younger than CGQ, but the amount of absolute time represented between them is difficult to assess with the available data. Current estimates suggest slow sedimentation rates and the potential for more than a million years of temporal separation [67] (see Discussion).

Suarez Site stratigraphy, lithology, and taphonomy

The Suarez Site represents a monodominant bonebed composed primarily of the numerous disarticulated remains of an early therizinosaurian theropod. A series of conference abstracts and publications have referred these remains to an indeterminate species of *Falcarius* [13,62,65,70] or an indeterminate therizinosaurian [53,71]. The holotype maxilla of *Geminiraptor suarezarum* comes from this bonebed, as does a large polacanthid ankylosaur [65]. The near-monotaxic nature of the Suarez Site closely resembles the composition of the CGQ bonebed [52].

Whereas detailed sedimentological and geochemical studies have been conducted on the CGQ bonebed [51,52], comprehensive analyses are lacking for the Suarez Site. Nevertheless, we provide a few preliminary observations here to contextualize the primary systematics work. The bone-bearing section at the Suarez Site appears to be contained entirely within a yellowish coarse to gravelly trough-cross-bedded sandstone unit. The base of this sandstone occurs ~1.2 m above the Morrison Formation contact. The lithology below the bone-bearing sandstone consists of mottled mudstones (interpreted as paleosols). Bones are exposed within a ~60-cm-thick section from the base of the sandstone unit, and this horizon is capped by a trough-cross-bedded gravelly sandstone with interbedded carbonate concretions. This gravelly sandstone unit is overlain by the regionally significant caprock [65], which is pebbly at its base and fines upwards for a total thickness of ~50–60 cm. The observations here are made from a single stratigraphic section in the western part of the site and support prior descriptions in the study area [52,65]. Additional sections are necessary to capture any lateral lithologic variation within the bonebed.

Unlike the CGQ bonebed, detailed taphonomic assessments of the Suarez Site bonebed are lacking. Preliminary observations indicate that abrasion on the ends of long bones and irregularities in the color of bone surfaces (bleached white to dark brown) are common, suggestive of some degree of subaerial exposure and transport prior to burial. A minimum of 30 individuals are present based on the number of left femora.

These initial observations of the Suarez Site demonstrate that it is stratigraphically and lithologically distinct from the CGQ bonebed, indicating independent depositional mechanisms for each site. The coarse, cross-bedded, bone-bearing sandstone at the Suarez Site likely represents gravel bars or a braided environment compared to the debris-flow, colluvial deposition and spring deposits inferred at CGQ [51,52]. However, there are some shared features between the two sites, including high bone density, prevalence of disarticulation, near-monotaxic composition, and individuals of similar size classes represented (suggestive of comparable ontogenetic stages; detailed osteohistological study of this material is in progress). These shared features hint at a mass-death-type assemblage, which was invoked as a causal explanation of the Crystal Geyser Quarry assemblage [51,52], but future taphonomic and sedimentological analyses are necessary to support these initial interpretations.

Materials and Methods

Specimens were described via firsthand visual inspection of fossil materials and segmented CT models. Measurements of cranial specimens are included in the Supporting Information. Comparisons were made from a combination of firsthand observations, reference photographs, 3D models, and published images and descriptions. Institutional abbreviations: CEUM, Prehistoric Museum, Utah State University Eastern, Price, Utah, USA; UMNH, Natural

History Museum of Utah, Salt Lake City, Utah, USA; NCSM, North Carolina Museum of Natural Sciences, Raleigh, North Carolina, USA; IVPP, Institute of Vertebrate Paleontology and Paleoanthropology, Beijing, China; MPC-D, Mongolian Institute of Paleontology, Ulaanbaatar, Mongolia; STM, Shandong Tianyu Museum of Nature, Pingyi, Shandong, China.

Excavation, Preparation, and Curation

All specimens collected from the Suarez Site were excavated under permits issued by the Bureau of Land Management to former curators of the Prehistoric Museum, Drs. Reese Barrick and Kenneth Carpenter (UT06-0038E and UT11-009E, respectively). Initial testing of the site was undertaken in July and September 2005, and full-scale excavation of the quarry was conducted from April 2006 to June 2014. A total of 2049 field numbers were assigned at the site. Many of those field numbers represent individual bones, but some were field jackets containing multiple elements. Preparation was undertaken at the Prehistoric Museum in Price, UT, with work on therizinosaurian cranial material described herein having been completed by former chief preparator John Bird and volunteer Bill Heffner. Preparation of these specimens was completed between 2006 and 2016 using standard mechanical techniques, including airscribe, dental picks, and toothbrush + water. The primary adhesive used by the lab at that time was PaleoBOND, with a “finishing coat” of vinac (polyvinyl acetate in acetone) applied to the exterior of some bones.

Owing to the taphonomy of the Suarez Site, associating elements with individual animals is not feasible. Therefore, individual catalog numbers were assigned to each element from the bonebed. The only two cranial elements described herein that were found in close proximity were CEUM 73787 and CEUM 73788. Those two elements were excavated in a small block

containing five vertebrae, an ulna, and a tooth, suggesting they were associated by chance. Field numbers and preparation timelines are included in the Supporting Information. Full field logs and preparation notes are housed at the Prehistoric Museum and are available upon request.

Terminology

We follow Nomina Anatomica Veterinaria [72] for directional terms, using 'rostral/cranial' and 'caudal' over 'anterior' and 'posterior'. Terminology for theropod maxillae features follows Hendrickx and Mateus [73] and Witmer [74]. Phylogenetic and taxonomic definitions for Therizinosauria follow Zanno [75].

Imaging

Specimens were photographed using a Canon EOS 760D camera with a Canon EF-S 60 mm macro lens. Three-dimensional renderings of surface scans were created using a combination of the iOS application Abound (formerly named "Metascan" [76]) and the Artec 3D Spider handheld scanner and Artec Studio 16 and 17 Professional software. The *G. suarezarum* holotype maxilla was CT-scanned at the Preclinical Imaging Core Facility at the University of Utah with an XYZ spacing of 0.04086 mm. Image stacks in TIF format were manually segmented in Aviso 3D 2022.2. All 3D files (both CT and 3D surface data) were exported and oriented in Blender (version 3.4), and figures were created in Adobe Photoshop (version 23.5.2) and Illustrator (version 26.5.1). All 3D and CT models and TIF stacks are available on Morphosource (see Supporting Information).

Phylogenetic analysis

Theropod Working Group matrix

To test the broader evolutionary relationships of *Geminiraptor suarezarum*, we scored the holotype maxilla into the Theropod Working Group (TWiG) coelurosaurian data matrix, iteratively constructed over 20+ years based on direct observations of specimens. We used the core version of this character matrix from Brusatte et al. [77], which was subsequently used and iteratively modified by Nesbitt et al. [78], Pei et al. [11], and Hattori et al. [79]. Here we use the Pei et al. [11]iteration and include *Fukuivenator paradoxus* scorings from Hattori et al. [79]. In addition to the updated scorings for the *Geminiraptor suarezarum* maxilla, we updated scorings for *Falcarius utahensis* crania based on new material described by Freimuth and Zanno [60]. This resulted in a data matrix of 166 taxa spanning the major coelurosaurian lineages and 853 morphological characters. All annotated data matrices are included in the Supporting Information.

Kobayashi et al. (2025) matrix

We ran additional phylogenetic analyses using a therizinosaurian-focused matrix modified from Kobayashi et al. [80] (the most recent iteration of Zanno's [75] dataset) to investigate the impact of *Geminiraptor suarezarum* on therizinosaurian in-group relationships. We scored *G. suarezarum* and *Fukuivenator paradoxus* into this matrix and updated scorings for *Falcarius utahensis* based on cranial material described by Freimuth and Zanno [60]. A total of 82 taxa and 356 morphological characters were employed.

Maximum parsimony analysis

We analyzed both TWiG and therizinosaurian-specific datasets in wTNT (version 1.6; [81]) under maximum parsimony optimality. We follow previous TWiG protocols [34,77–79] and used *Allosaurus* as the outgroup to root the tree. A total of 141 characters were ordered (additive) in the TWiG analysis (those listed in Nesbitt et al. [78]; see Supporting Information), and 20 characters were ordered in the therizinosaurian-focused analyses (those of Kobayashi et al. [80]; Supporting Information). After increasing the tree memory to 99,999, we analyzed both Pei et al. [11] and Kobayashi et al. [80] matrices using the 'New Technology' search option, with sectorial search, ratchet, tree drift, and tree fuse options activated with default parameters under equal weights. The minimum length tree (MPT) was run until it was hit 15 times, checking every three hits, a process that samples as many tree islands as possible [78]. The RAM-stored trees were subsequently analyzed using tree bisection and reconnection branch swapping (TBR) for an additional search of 99,999 trees—this allows for more full exploration of each tree island [47,78]. Finally, a strict consensus tree for both the Pei et al. [11] and Kobayashi et al. [80] datasets was produced (with zero-length branch-collapsing rule) and is presented with Bremer support values, where high values indicate greater support for a given node. For the therizinosaurian analysis, we also produced a 50% majority-rule tree following Kobayashi et al. [80]. All TNT files are included in the Supporting Information.

Bayesian likelihood analysis

We additionally performed Bayesian phylogenetic analyses using the Pei et al. [11]dataset in MrBayes (version 3.2.7; [82]). As in the parsimony analysis, we used *Allosaurus* as the outgroup and additionally constrained the backbone of the tree using *Sinraptor dongi.* The same suite of characters used in the parsimony analyses was designated as additive in both

analyses. Because MrBayes limits multi-state characters to a maximum of six states, we altered character 108 in the Pei et al. [11] matrix (number of sacral vertebrae) for *Lithornis* and five extant birds. For these taxa, states 5–7 were scored as a single state, combined to "10 or more" sacral vertebrae. We set constant characters among all taxa as unobservable ("coding=variable") and used the Mkv Model and log-normally distributed character rate variation, which has been favored over gamma distributions for discrete morphological data with slow evolvability [83]. We ran each analysis for 100 million generations with four runs, four chains, and three swaps per cycle. We executed a 25% burn-in and sampled every 1000 generations. Analyses were terminated when the average standard deviation of split frequencies reached a threshold of 0.01, indicative of convergence of the MCMC chain post-burn-in; this occurred at roughly 44 million generations for the Pei et al. [11] dataset. Finally, we modeled coelurosaurian evolution under the fossilized birth-death model (FBD; [84]) with independent (unlinked) parameters. Tip-dates were included based on published age ranges for TWiG taxa from Ding et al. [85], with updated dates on Yellow Cat Member taxa from Kirkland et al. [69]. Trees were visualized in FigTree (v1.4.4), and figures were modified in Adobe Illustrator. All nexus files are included in the Supporting Information.

Morphometrics

To compare the shape and proportions of the maxilla of *Geminiraptor suarezarum* with other coelurosaurians, we updated the dromaeosaurid-focused dataset of Powers et al. [86] and performed a principal component analysis. We used 15 linear variables acquired via a combination of firsthand measurements and digital measurements in ImageJ (Fig 2). We included 16 dromaeosaurid taxa (from [86]), eight troodontids, six therizinosaurians, seven

ornithomimosaurians, four early diverging tyrannosauroids, two avialans (three specimens), *Haplocheirus sollers*, *Incisivosaurus gauthieri*, *Ornitholestes hermanni*, and *Zuolong salleei*. All raw measurements were imported into PAST (version 4.0.3) and log-transformed. Variables that were missing or could not be confidently assessed were entered as blank.

Fig 2. Maxillary measurements employed in principal component analysis, following Powers et al. (2020). *Geminiraptor suarezarum* (CEUM 73719) is used to illustrate the variables. Note that missing or incomplete variables on specimens (in this example, AntFen.H, AntFen.L, VentMH.post, L9Alv) are excluded from analyses.

We ran additional iterations of this dataset after correcting for size biases. We scaled all measurements by dividing them by maxilla height (MxH) because most taxa preserved this measurement (*Utahraptor ostrommaysi* has an incomplete maxilla and was excluded from this iteration). To determine which principal components yield the most informative variation, we used the broken stick method in PAST [86,87]. By this method, only the first two principal components in all analyses were deemed informative, but we include PC 3 in our plots as well. Raw measurements and PCA data are available in the Supporting Information.

Systematic Paleontology

Dinosauria Owen 1842

Saurischia Seeley 1887

Theropoda Marsh 1881

Coelurosauria von Huene 1914

Maniraptora Gauthier 1986

Therizinosauria Russel and Dong 1993 (*sensu* Zanno 2010b)

Falcarius Kirkland et al. 2005

Emended Diagnosis

Falcarius is a mid-sized primitive therizinosaurian characterized by the following combination of characters (Figs 3 and 4; asterisks denote putative autapomorphies, specimens in which diagnostic features are visible indicated in parentheses): (1) accessory pneumatic excavation of antorbital fossa that emanates from the promaxillary fenestra and extends caudodorsally to level with the midpoint of the maxillary fenestra (absent in known therizinosaurians [except possibly STM 31-1] but common and sporadically distributed among tetanurans [74]; *Fa. suarezarum* CEUM 73719); (2) jugal with pronounced concavity of the ventral margin of the maxillary process* (*Fa. utahensis* NCSM 39115); (3) elongated, dorsolaterally oriented facet for postorbital contact rostral to the postorbital process and rostral margin of the frontoparietal fossa on the frontal* (*Fa. utahensis* UMNH VP 14525, 14525, NCSM 39077; *Fa. suarezarum* CEUM 52574); (4) paired dorsoventrally expanded depressions near midline of parietals at frontoparietal suture* (degree of expression variable in sample; *Fa. utahensis* NCSM 39068, 39117); (5) thin, ridge-like sagittal crest on parietal (unique among therizinosaurians but shared with deinonychosaurs and other non-maniraptoriform coelurosaurians; *Fa. utahensis* NCSM 39068, 39077, 39117); (6) partial envelopment of squamosal process of postorbital by dorsal lip of squamosal at their contact (shared with at least one ornithomimosaurian, TMP 95.110.001; *Fa. utahensis* NCSM 39076); (7) expansive, deeply depressed, and highly pneumatic subcondylar and subotic recesses each possessing multiple

pneumatic fossae (*Fa. utahensis* UMNH VP 15000, 15001); (8) inflated, highly pneumatic basisphenoid that houses multiple pneumatic fossae within the basipterygoid recess and possesses hypertrophied basisphenoidal recess (*Fa. utahensis* UMNH VP 15000, 15001); (9) absence of lateral shelf on dentary (shared with *Fukuivenator paradoxus* among possible therizinosaurians; *Fa. utahensis* UMNH VP 14527–14529, 15259, NCSM 33476, 33523; *Fa. suarezarum* CEUM 52511, 77263, 81539); (10) rostral dentary teeth conical and adenticulate to fifth tooth position (*Fa. utahensis* UMNH VP 14528, 14529, 15259, NCSM 33476, 33523; *Fa. suarezarum* CEUM 52511, 77263, 81539); (11) median ridge dividing ventral sulcus of cervical vertebrae (positionally variable among cervical series; numerous specimens of *Fa. utahensis* and *Fa. suarezarum*); (12) infraprezygapophyseal fossa on cranial dorsal vertebrae divided into three accessory fossae (*Fa. utahensis* UMNH VP 14534); (13) accessory caudal centrodiapophyseal lamina on cranial dorsal vertebra (*Fa. utahensis* UMNH VP 14534); (14) elongate (>4:1 length:width) distal caudal centra (shared with *Fu. paradoxus* among possible therizinosaurians; numerous specimens of *Fa. utahensis* and *Fa. suarezarum*); (15) mid-series chevrons possessing markedly distinct cranial tubercles (prominence of tubercles varies positionally; representative *Fa. utahensis* UMNH VP 12390, 12395; representative *Fa. suarezarum* CEUM 78314); (16) proximal pubic tubercle well developed and caudolaterally oriented, occurring at cranialmost margin of acetabulum (representative *Fa. utahensis* UMNH VP 14539; representative *Fa. suarezarum* CEUM 78261); (17) hypertrophied obliquely oriented entepicondyle, with concave caudal margin, and well-defined groove extending proximally up humeral shaft (representative *Fa. utahensis* UMNH VP 12284; representative *Fa. suarezarum* CEUM 77102); (18) PI-2 flexor tubercle with deep distally positioned collateral ligament pits (representative *Fa. utahensis* UMNH VP 12314; representative *Fa. suarezarum* CEUM 78330).

Fig 3. Diagnostic cranial features of *Falcarius*. Numbered features correspond to those in the genus diagnosis. (A) Left maxilla (*Fa. suarezarum* CEUM 73719) in lateral view. (B) Right jugal (*Fa. utahensis* NCSM 39115) in lateral view. (C) Frontals (*Fa. utahensis* UMNH VP 14524 and 14525) in dorsal view. (D) Right frontal (*Fa. suarezarum* CEUM 52574) in dorsal view. (E) Parietals (*Fa. utahensis* NCSM 39068) in oblique (rostrodorsolateral) view. (F) Right squamosal (*Fa. utahensis* NCSM 39076) in lateral view. (G) Braincase (*Fa. utahensis* UMNH VP 15000) in caudal view. (H) Braincase (*Fa. utahensis* UMNH VP 15000) in right lateral view. (I) Partial basisphenoid (*Fa. suarezarum* CEUM 77254) in right lateral view. (J) Right dentary (*Fa. utahensis* UMNH VP 14529) in lateral view. (K) Left dentary (*Fa. suarezarum* CEUM 52511) in lateral view. Scale bars 1 cm.

Fig 4. Diagnostic postcranial elements of *Falcarius*. Numbered features correspond to those in the genus diagnosis. (A) Cervical vertebra (*Fa. utahensis* NCSM 35019) in ventral view. (B) Cranial dorsal vertebra (*Fa. utahensis* UMNH VP 14534) in right lateral view. (C) Distal caudal vertebra (*Fa. utahensis* NCSM 28026) in left lateral view. (D) Manual ungual PI-2 (*Fa. utahensis* UMNH VP 12314) in lateral view. (E) Manual ungual PI-2 (*Fa. suarezarum* CEUM 78330) in lateral view. (F) Chevron (*Fa. suarezarum* CEUM 78314) in left lateral view. (G) Right humerus (*Fa. utahensis* UMNH VP 12284) in cranial view. (H) Right humerus (*Fa. suarezarum* CEUM 77102) in cranial view. (I) Left pubis (*Fa. utahensis* UMNH VP 14539) in lateral view. (J) Left pubis (*Fa. suarezarum* CEUM 78261) in lateral view. Scale bars 1 cm (A–F), 5 cm (G–J).

Remarks

The paired depressions near the parietal midline (proposed as autapomorphic) are absent in all known therizinosaurians and other theropods, to our knowledge, but this anatomical view is rarely figured and/or preserved in a manner that can be observed in many taxa. Of potential interest, incipient depressions in this location are present in the parietals of the thescelosaurine ornithischian *Fona herzogae* [88]. Future analyses may reveal that this feature is more widespread in dinosaurs than currently recognized.

We emended Zanno's [75] diagnostic character regarding the postorbital contact on the frontal to include its dorsolateral orientation and rostral location with respect to the postorbital process and rostral margin of the defined frontoparietal fossa, providing a combination of features that are autapomorphic. Similarly, we emphasized the pneumatic features of the basipterygoid recess in addition to the hypertrophied basisphenoid recess.

Although we refer all therizinosaurian material from the Crystal Geyser Quarry and Suarez Site bonebeds to *Falcarius*, we recognize that the largely isolated preservation and lack of confident association between skeletal elements within each bonebed present potential problems with their referral to a single taxon to the species level. Taxonomic identifications are further complicated by a range of sizes and ontogenetic stages represented in each bonebed, in addition to other potential sources of intraspecific variation (e.g., sexual dimorphism, individual variation). Similarly, subtle species-level differences may not be detectable when comparisons are limited to isolated elements. Although these and other bonebeds hypothesized as monospecific or monodominant offer refined spatiotemporal context for assessing patterns of morphological variation, careful anatomical analyses that consider a range of intra- and

interspecific sources of variation (ideally grounded by patterns in extant study systems) are necessary for accurate taxonomic assessments.

Falcarius utahensis Kirkland et al. 2005

Holotype

UMNH VP 15000, partial subadult braincase.

Horizon and Locality

Crystal Geyser Quarry (UMNH.VP.LOC.157), Early Cretaceous (?Berriasian–Valanginian) [50,65,75] lower Yellow Cat Member, Cedar Mountain Formation, Utah, USA. Bonebed sits directly atop the Jurassic Morrison Formation and ~1 m below regional calcrete "caprock" (Fig 1) [51,52,65].

Referred specimens

Paratype (UMNH VP 15001, braincase) and thousands of specimens from the Crystal Geyser Quarry reposited at the Natural History Museum of Utah (Salt Lake City, Utah, USA) and the North Carolina Museum of Natural Sciences (Raleigh, North Carolina, USA).

Emended Diagnosis

Falcarius utahensis is a mid-sized, primitive therizinosaurian with the following combination of characters that differ from *Fa. suarezarum* (Fig 5; specimens in which diagnostic

features are visible indicated in parentheses): (1) transversely thin maxillary interfenestral bar with concave caudal margin, deeply incised ventrally by the antorbital sinus (NCSM 39000); (2) gracile frontal process of postorbital with straight dorsal margin (NCSM 39106, 39075, 39093, UMNH VP 14567); (3) concave dorsal margin between frontal and squamosal processes of postorbital (NCSM 39106, 39075, UMNH VP 14567); (4) ridge for circumorbital membrane on medial surface of postorbital gently arched along ventral margin of squamosal process (NCSM 39106); (5) orbital surface on jugal process of postorbital visible in medial view (NCSM 39106, 39075, 39093, UMNH VP 14567); (6) curved, rostroventrally projected jugal process of postorbital (NCSM 39106, 39075, 39093); (7) medial and lateral mandibular condyles of the quadrate project to same distal extent (NCSM 39072, 33484, UMNH VP 14559, 14558); (8) strongly recurved non-incisiform (i.e., distal to position d5) dentary teeth with reduced basal swelling and lingual inflection of basal mesial carina (UMNH VP 15259, 14527).

Remarks

Referral of specimens to *Fa. utahensis* is challenged by the disarticulation of skeletons from the holotype locality. Kirland et al. [50] designated a paratype (UMNH VP 15001, braincase) based on morphological overlap. Other specimens were referred by Kirkland et al. [50] based on therizinosaurian-consistent morphology and the nature of the locality (thousands of individuals in a bonebed of near mono-dominant composition). Given these factors, all therizinosaurian material from the Crystal Geyser Quarry are also likely referrable to this taxon. A best-practice approach would prevent us from referring elements that lack overlap with the designated holotype specimen; however, this would render the morphology of *Fa. utahensis* virtually unknown. Here, we adopt a practical approach, referring specimens of the most

abundant morphotypes, exemplified by other therizinosaurians, to *Fa*. *utahensis*. We consider each of these referrals a hypothesis that can be overturned with additional study and/or new data. It should be noted that we have identified rare coelurosaurian elements from the quarry that are morphologically distinct from the specimens referred to *Fa*. *utahensis*, supporting our interpretations.

***Falcarius suarezarum* comb. nov.**

Geminiraptor suarezarum Senter et al. 2010

Holotype

CEUM 73719, partial left maxilla.

Horizon and Locality

Suarez Site (Gr 286v), Early Cretaceous (?Berriasian–Valanginian) [65] lower Yellow Cat Member, Cedar Mountain Formation, Utah, USA. Bonebed occurs ~1.2 m above contact with Jurassic-age Morrison Formation, ~0.4 m below regional marker calcrete unit (Fig 1) [65].

Referred Material

Thousands of specimens from the Suarez Site, reposited at the Prehistoric Museum (CEUM), Utah State University–Eastern, Price, Utah, USA.

Emended Diagnosis

Falcarius suarezarum is a mid-sized, primitive therizinosaurian with the following combination of characters that differ from *Fa. utahensis* (Fig 5; specimens in which diagnostic features are visible indicated in parentheses): (1) transversely thickened maxillary interfenestral bar, housing maxillary antrum through its dorsoventral extent (CEUM 73719); (2) attenuated and sinuous dorsal margin of the frontal process of the postorbital (CEUM 73942); (3) straight dorsal margin between frontal and squamosal processes of postorbital (CEUM 73942, 73787); (4) ridge for circumorbital membrane on medial surface of postorbital forms near-right angle and extends to dorsal margin of squamosal process (CEUM 73942, 73787); (5) orbital surface of postorbital obscured by rostral margin of the jugal process in medial view (CEUM 73787); (6) straight, ventrally projected jugal process of postorbital (CEUM 73787); (7) medial mandibular condyle of the quadrate projects further distally than the lateral mandibular condyle (CEUM 73773); (8) weakly recurved non-incisiform (i.e., distal to position d5) dentary teeth possessing prominent basal swelling and lacking lingual inflection of basal mesial carina (CEUM 77263).

Fig 5. Differentially diagnostic features of *Falcarius utahensis* (A, C, F, H) and *Fa. suarezarum* (B, D, E, G, I). Numbered features correspond to those listed in the *Fa. utahensis* and *Fa. suarezarum* species diagnoses. (A–B) Left maxillae in caudal view (A, NCSM 39000; B, CEUM 73719). (C–E) Postorbitals in medial view (C, NCSM 39106 [reversed]; D, CEUM 73942; E, CEUM 73787). (F–G) Quadrates in caudal view (F, UMNH VP 14559 [right quadrate]; G, CEUM 73773 [left quadrate]). (H–I) Dentary teeth in lingual view (H, 14th dentary tooth of UMNH VP 15259; I, 12th dentary tooth of CEUM 77263). Scale bar is 1 cm (A–G), 1 mm (H–I).

Remarks

The features used in the original diagnosis of *"Geminiraptor" suarezarum* are now known to characterize early branching therizinosaurians or are more broadly distributed within Theropoda. These features include: maxilla with extensive pneumatization internal to the antorbital fossa, inflating the bone so it has a triangular cross-section (present in *Falcarius utahensis* and *Erlikosaurus andrewsi*, discussed below); a large, craniocaudally elongate maxillary fenestra (present in early diverging therizinosauroids, troodontids, and early diverging alvarezsaurians; [34,58,89–91]; promaxillary fenestra visible in lateral view (widely if sporadically distributed among tetanurans [73,74]); craniocaudally narrow promaxillary strut and interfenestral strut (present in therizinosaurians and troodontids); small, square dental alveoli with bony septa between them (present in *Fa. utahensis* and other therizinosaurians, present in *Urbacodon* [61] but absent in most other troodontids).

Given the similarities of the *"Geminiraptor" suarezarum* holotype maxilla and the maxillae referred to *Falcarius utahensis*, the number of shared diagnostic postcranial features between *Falcarius utahensis* and the Suarez Site material, and relatively few differences in the maxillae and other cranial elements between the two bonebeds, we assign specimens from both bonebeds to the genus *Falcarius* and form a new combination, *Falcarius suarezarum*.

Following our preferred practical approach (described above for *Fa. utahensis*), we tentatively refer cranial elements from the Suarez Site bonebed that exhibit therizinosaurian morphology to *Fa. suarezarum* and use these to expand the diagnosis beyond maxillary features. Thousands of additional therizinosaurian postcranial elements from the Suarez Site likely also represent *Fa. suarezarum*. However, a detailed study of each of these elements is necessary before confident referral.

Results

Description

The disarticulated remains from the Suarez Site represent most of the skeleton for *Falcarius suarezarum*. Cranial elements represented include the holotype maxilla, a possible lacrimal, a frontal, two postorbitals, a quadrate, at least eight dentaries, and braincase fragments, many of which overlap with *Fa. utahensis* (Fig 6). These cranial elements are described here, and postcranial remains are subject to a more thorough osteological description in the future.

Fig 6. Overview of cranial remains for *Falcarius utahensis* (A) and *Fa. suarezarum* (B). Some elements are rescaled and mirrored for best fit. Composite *Fa. utahensis* skull derived from Freimuth and Zanno (2025). For specimen numbers and digital models, see the Supporting Information.

Maxilla

The holotype left maxilla (CEUM 73719) of *Falcarius* (“*Geminiraptor*”) *suarezarum* is missing the rostralmost portion of the rostral body and the jugal ramus beneath the antorbital fenestra, but it retains its three-dimensional morphology (Figs 7 and 8). CEUM 73719 is overall rostrocaudally long and dorsoventrally low, unlike the short and tall maxillae of *Ornitholestes hermanni*, *Incisivosaurus gauthieri*, and *Fukuivenator paradoxus* [79,92]. The angle of the dorsal margin of the rostral body is offset ~22–23° from the level of the tooth row, and the ascending ramus arcs to become subparallel with the tooth row. This angle is similar to

troodontids and some therizinosaurians, including *Fa. utahensis* (~21–25°) and *Erlikosaurus andrewsi* (~23°) [59,60].

Fig 7. Holotype maxilla of *Falcarius suarezarum* comb. nov. Left maxilla CEUM 73719 in lateral (A), medial (B), dorsal (C), ventral (D), caudolateral (E), and rostral (F) views. Scale bar is 1 cm.

Fig 8. Computed tomography renderings of *Fa. utahensis* and *Fa. suarezarum* maxillae. NCSM 39000 left maxilla of *Falcarius utahensis* (A, C, E, G, I, K), and CEUM 73719, left maxilla of *Falcarius suarezarum* (B, D, F, H, J, L), in dorsal (A, B), lateral (C, D), medial (E–H), ventral (I, J), and rostral (K, L) view. Scale bar 1 cm.

The rostral body is broken rostrally, exposing the inflated chamber of the promaxillary sinus. The contact with the premaxilla is missing. The lateral surface of the rostral body is perforated by at least two small foramina, similar to *Fa. utahensis* (NCSM 39118) and *J. yixianensis*, but unlike the numerous foramina of *E. andrewsi* [58–60]. Medially, the rostral body is expanded to form a broad palatal shelf and a large, pneumatized internal chamber that is triangular in cross-section, a feature used to originally diagnose this taxon (Fig 7) [13]. However, this broad palatal shelf and inflated rostral body are more ubiquitously distributed in therizinosaurians (*Fa. utahensis* [60], *E. andrewsi* [59]) and troodontids (*Xixiasaurus henanensis* [37], *Byronosaurus jaffei* [38], *Urbacodon* sp. [61]). In particular, *Fa. utahensis* exhibits a similar degree of medial expansion and the same triangular rostral outline in three specimens (NCSM 39000, NCSM 39118, UMNH VP 21794), although each exhibits variable taphonomic

damage [60]. Curiously, the rostral body of *E. andrewsi* appears medially inflated and internally pneumatized, but it apparently lacks an external promaxillary fenestra [55,59]. In any case, the pneumatization internal to the antorbital fossa and triangular cross-section of the maxillary rostral body are not unique to *Fa. suarezarum*.

The antorbital fossa of the maxilla is large and possesses openings for an inferred large antorbital fenestra, a large and elongated maxillary fenestra, and a tall, slit-like promaxillary fenestra. The promaxillary fenestra is mostly tucked within the rostral concavity of the antorbital fossa but partially visible in lateral view [13]. The presence of a promaxillary fenestra is widespread among coelurosaurians [74], including in the early therizinosaurians *Fa. utahensis* [60] and *Fu. paradoxus* [79], the early oviraptorosaurian *Incisivosaurus gauthieri* [92], the early alvarezsaurians *Haplocheirus sollers* [90] and *Aorun zhaoi* [91], and early diverging troodontids [15,42–45], whereas this opening is absent in the late-diverging therizinosaurid *E. andrewsi* [55,59] and late-diverging troodontids [31,33,34,37,38,61].

A deeply excavated narrow recess forms the rostrodorsal margin of the antorbital fossa as a continuation of the promaxillary fenestra. In this area, an elongated, shallow, trough-like depression (interpreted as a pneumatic excavation *sensu* Witmer [74] and Hendrickx and Mateus [73]) emerges from the promaxillary fenestra and incises the lateral surface of the antorbital fossa along the dorsal margin of the maxillary fenestra, terminating at a level just caudal to the midpoint of this opening (Fig 7A). This region is poorly preserved *Fa. utahensis* and cannot be assessed; however, a pneumatic excavation on the lateral surface of the antorbital fossa is absent in later branching therizinosaurians (except possibly STM 31-1 [89]) and troodontids [33,34,47,58,59,61]. A pneumatic excavation is present dorsal to the maxillary fenestra of *Fu. paradoxus,* some tyrannosauroids, and several non-coelurosaurian tetanurans, but it extends

further caudally to near the level of the antorbital fenestra in these taxa [73,74,79,93,94]. The prominence of this excavation varies between the right and left maxilla of *Fu. paradoxus* (fig 4 of [79]) and similar variable patterns have been noted in pneumatic features in other theropods [74,95]. Despite variation in the prominence of pneumatic features, their presence or absence and anatomical position appear less variable (at least in *Fa. utahensis* [60] and extant *Alligator* [96]). Thus, the presence of this excavation in *Fa. suarezarum* and its apparent absence in any later diverging therizinosauroids may represent a differentially diagnostic feature.

The promaxillary strut—the bony pillar between the promaxillary and maxillary fenestrae—is relatively craniocaudally narrow in *Fa. suarezarum* and proportionally similar to the therizinosaurians *Fa. utahensis* [60] and *J. yixianensis* [58], as well as the early alvarezsaurians *H. sollers* [90]and *A. zhaoi* [91]. It is also similar to that of the troodontid *Sinovenator changii* [39], whereas this structure is wide in *Sinusonasus magnodens* [45] and *Hesperornithoides miessleri* [47] and absent in later diverging troodontids (due to the absence of a promaxillary fenestra). The promaxillary strut is crescentic and follows the curvature of the maxillary and promaxillary fenestrae, as in *Fa. utahensis* [60], but unlike the vertical strut in *Sino. changii* [39].

The maxillary fenestra is proportionally large and elongated, pinching slightly at its rostral and caudal margins to form a fusiform shape. As noted by Senter et al. [13], its shape resembles late-diverging troodontids. However, the fusiform shape and rounded dorsal and ventral borders of the maxillary fenestra in *Fa. suarezarum* differs from the subtriangular shape and straight ventral border in most troodontids [31,33,34,37,42,43], as well as the more circular shape seen in the early diverging troodontid *Sino. changii* [39], *Fu. paradoxus* [79], *O. hermanni,* and dromaeosaurids [24,97]. Instead, the shape and size of the maxillary fenestra in

Fa. suarezarum resembles the large oval-shaped fenestrae in specimens of *Fa. utahensis* [58] and the large openings of *J. yixianensis*, *Beipiaosaurus* sp. (STM 31-1), and the therizinosauroid IVPP V 18597, although the shape of the maxillary fenestra in these specimens is somewhat taphonomically altered [58,89]. The rostral and ventral borders of the maxillary fenestra are offset from the borders of the antorbital fossa in *Fa. suarezarum*, therizinosaurians [58–60,79], and *Sinovenator* [39]. This contrasts with other troodontids, in which the margin of the antorbital fossa forms the ventral margin of the maxillary fenestra (*Sinu. magnodens* [45], *Xixiasaurus henanensis* [37], *Almas ukhaa* [34], *Liaoningvenator curriei* [43]), or forms both the rostral and ventral maxillary fenestra margins (*Zanabazar junior*, *Saurornithoides mongoliensis* [31], *Gobivenator mongoliensis* [33]. *Fa. suarezarum* differs from *Hesperornithoides miesserli* (and dromaeosaurids generally [97]) in that the maxillary fenestra is not dorsally positioned [47].

The interfenestral bar is transversely thickened in caudal view, which differs from the concave caudal surface of *Fa. utahensis* and the thin bar of *Fu. paradoxus*, as well as all other therizinosaurians and troodontids, to our knowledge. In lateral view, the interfenestral bar is pinched near its midpoint to form an hourglass shape, as in *Fa. utahensis* [60] and *Sino. changii* [39] but unlike the comparatively straight margins in early alvarezsaurians [90,91]. Whereas the dorsal aspect of the interfenestral bar is continuous with the lateral surface of the maxilla [13], the ventral aspect is medially recessed from the ventral margin of the maxilla, separated by a shallow groove that denotes the floor of the antorbital fossa, as in the early diverging therizinosaurians *Fu. paradoxus* [79], *Fa. utahensis* [60], and *J. yixianensis* [58]. This differs from most troodontids, in which the interfenestral bar is completely inset dorsally and ventrally from the lateral surface of the maxilla [31,33,39,42,43,45,61], but also differs from *Byronosaurus jaffei* [38] and *Linhenvenator tani* [32] in which the entire interfenestral bar is

flush with the lateral maxillary surface. Although the ventral portion of the interfenestral bar is damaged in *Fa. suarezarum*, this taxon further aligns with known therizinosaurians [60,79] and early oviraptorosaurians [92] in that the rostral margin of the interfenestral bar is kinked near its midpoint to accommodate the maxillary fenestra (except *J. yixianensis* [58] where it is vertical), and the caudal margin is D-shaped to form the border of the antorbital fenestra. This contrasts with most troodontids, in which the interfenestral bar is kinked at or near the maxilla ventral margin, forming a trapezoidal outline and a triangular to quadrangular rostral margin of the antorbital fenestra [31,34,42,43,45]. In a referred specimen of *Sino. changii*, the interfenestral bar is kinked at its midpoint, but it is inclined rostrally to form a quadrangular rostral margin of the antorbital fenestra (fig 1 of [98]).

CEUM 73719 preserves parts of 11 alveoli (the cranial- and caudalmost alveoli are badly damaged and only observable with CT data). As noted by Senter et al. [13], alveoli are subcircular and separated by bony septa. The total maxillary tooth count is impossible to assess, but the more rostral alveoli are larger than the caudal alveoli, and all are relatively equally distributed. This pattern is similar to therizinosaurians (*Fa. utahensis*, *E. andrewsi*, *J. yixianensis* [58–60]), but unlike troodontids, in which the rostral alveoli beneath the maxillary fenestra are small and tightly packed, transitioning to larger, widely spaced alveoli caudally [31,37,38,45,61]. Unlike troodontids [30,33,37] (except possibly *Byronosaurus* [38]) but similar to *Fa. utahensis* [60] and *E. andrewsi* [55,59], *Fa. suarezarum* lacks a row of nutrient foramina on the palatal surface that parallel the toothrow. The palatal surface is dorsoventrally flat (to accommodate the expanded internal pneumatic sinuses of the maxilla) and flush with the level of the alveoli as in *Fa. utahensis* and *I. gauthieri* [60,92], but this is unlike the vaulted palatal surface of many troodontids [30,31,38,42,61], as well as tyrannosauroids [99].

Paranasal sinuses of the maxilla

Computed tomography data elucidate the morphology of the paranasal sinuses of the maxilla and their associated openings in both *Fa. utahensis* and *Fa. suarezarum* (Figs 8–10). Three pneumatic sinuses are present and communicate with the lateral maxillary surface via their corresponding fenestrae: the promaxillary sinus via the promaxillary fenestra, the maxillary antrum via the maxillary fenestra, and the antorbital sinus via the antorbital fenestra. Although taphonomic damage in both *Fa. suarezarum* and *Fa. utahensis* limits comparisons of homologous structures, some similarities are present.

Fig 9. Segmented paranasal sinuses of *Falcarius suarezarum* (CEUM 73719). Maxilla in lateral (A–C; inset box in (B) corresponds to (C)), dorsal (D), ventral (E), rostral (F, G) and medial view (H, I). Scale bars are 1 cm (B, D–H are to scale).

Fig 10. Segmented paranasal sinuses of *Falcarius utahensis* (NCSM 39000). Maxilla in lateral (A–C; inset box in (B) corresponds to (C)), dorsal (D), ventral (E), rostral (F, G) and medial view (H, I). Scale bars are 1 cm (B, D–I are to scale).

The promaxillary sinus (promaxillary recess *sensu* Hendrickx and Mateus [73]) in *Fa. suarezarum* inflates the rostral body, creating a triangular prism-like cavity enclosed dorsally and medially by the vestibular bulla (Figs 7 and 9). The vestibular bulla is similarly enclosed in *Marshosaurus bicentesimus* and possibly *Ornitholestes hermanni* and *Deinonychus antirrhopus*, but it is apparently open medially in *Allosaurus*, *Sinraptor dongi*, some tyrannosauroids, and *Fu.*

paradoxus [73,74,79]. The area of the vestibular bulla is too damaged to assess in *Fa. utahensis*. The promaxillary sinus in both *Fa. suarezarum* and *Fa. utahensis* is dorsally and ventrally extensive (Figs 9A–C, 10A–C). A long, finger-like projection of the promaxillary sinus extends along the interior of the ascending ramus (best seen in *Fa. suarezarum* [Fig 9A–C]), partially preserved in *Fa. utahensis* [Fig 10A–C]), which is similar to but more extensive than that seen in *Alioramus altai* [95] and *Tyrannosaurus* [100]. In *Fa. suarezarum*, a small circular opening (an accessory foramen) occurs on the medial surface of the ascending ramus near the midpoint of the dorsal extension of the promaxillary sinus (Fig 9H–I). Presumably, this opening allows communication between the promaxillary sinus and the passageway to the external naris. This area is damaged and unable to be assessed in *Fa. utahensis*, but such an opening appears present and more extensive in *Sinraptor dongi* (although it may be taphonomically affected [101]), but absent in other early tetanurans [73], tyrannosaurids [74,93,95,100], *Erlikosaurus andrewsi* [59], and the troodontids *Urbacodon* sp. [61], *Byronosaurus jaffei* [38], and *Troodon formosus* [30]. In both *Fa. suarezarum* and *Fa. utahensis*, the promaxillary sinus appears confluent with the dorsal alveolar canal, forming a braided, web-like series of interconnecting spaces between the two structures (Figs 9C and 10C). This differs from *Neovenator* [102] and apparently *Alioramus* [95], in which the promaxillary sinus approaches but does not communicate with any portion of the maxillary neurovascular canal system. In both *Fa. utahensis* and *Fa. suarezarum*, the rostral extent of the dorsal alveolar canal cannot be gleaned due to taphonomic damage, but it extends caudally to at least the level of the rostral border of the interfenestral bar (Figs 9A and 10A). In both taxa, along the preserved length of the canal (the caudal portion is damaged in *Fa. utahensis*), the dorsal alveolar canal sends ventrally projecting intralveolar offshoots that open to the lateral surface of bone via the maxillary alveolar canals.

In *Fa. suarezarum*, CT data reveal an elongated trough present on the rostromedial surface of the maxilla; this may represent a portion of the articular facet for the vomer (Figs 8H, 8L, 9F). This area is poorly preserved in *Fa. utahensis*, but preserved bone chips follow a similar trough-like outline and may represent the same contact.

The maxillary antrum in both *Fa. suarezarum* and *Fa. utahensis* is large and oval-shaped. The preantral strut between the promaxillary sinus and the maxillary antrum is damaged in *Fa. utahensis* and the presence of a rostromedial maxillary fenestra [73] (=fenestra communicans of [74]) is unclear. In *Fa. suarezarum*, the preantral strut is represented by a transversely extensive but damaged sheet of bone; if a rostromedial maxillary fenestra was present, it was likely small (Fig 8L). A rostromedial maxillary fenestra is present in *Allosaurus fragilis*, tyrannosauroids [73,74], and the troodontid *X. henanensis* (although the latter specimen is damaged [37], but this aperture is apparently absent in *O. hermanni* and *M. bicentesimus* [74]. In *Fa. utahensis*, the ventromedial and caudal walls of the maxillary antrum are missing, but the dorsal half appears to have formed an extensive suprantral strut that is level with the dorsal margin of the maxillary fenestra. The medial wall of the maxillary antrum is similarly damaged in *Fa. suarezarum*, but it also appears to have possessed an enclosed ceiling (suprantral strut) at or just dorsal to the dorsal margin of the maxillary fenestra. Both *Fa. suarezarum* and *Fa. utahensis* differs from *Fu. paradoxus*, in which the suprantral strut only reaches the mid-dorsal height of the maxillary fenestra [79]. In three Early Cretaceous Chinese therizinosauroids (*J. yixianensis*, STM31-1, and IVPP V 18957), a sheet of vertically oriented bone is visible within the maxillary fenestra, suggesting that the maxillary antrum was enclosed in these taxa; however, CT data (and retrodeformation) are necessary to test this hypothesis. The maxillary antrum is apparently unenclosed in *Ornithomimus edmontonicus* [103] and tyrannosauroids, but the thin sheet of bone

that typifies this structure is easily lost to taphonomic processes [74]. Caudally, the maxillary antrum in *Fa. suarezarum* is bordered medially by a vertical bony wall, forming a narrow, tall epiantral recess as a caudodorsal extension of the maxillary antrum (Figs 9A–B and 9H–I). A horizontal strut of bone appears to delineate a separate dorsal outpocketing from the main area of the epiantral recess (Figs 9A–B and 9H–I). The rest of this area (i.e., postantral strut, caudomedial maxillary fenestra) is damaged, but this may represent a connective opening with the antorbital sinus. The epiantral recess in *Fa. utahensis* is similarly deep, but unlike *Fa. suarezarum*, the caudodorsal aspect of the maxillary antrum in *Fa. utahensis* appears transversely swollen and lacks any inward pinching; this swelling is likely a preservational artifact.

The extent of the antorbital sinus in *Fa. suarezarum* and *Fa. utahensis* is difficult to assess due to taphonomic damage, but sufficient overlap in their preserved portions permits comparisons. The rostrodorsal half of the antorbital sinus is observable in *Fa. suarezarum,* whereas the rostroventral portion is visible in *Fa. utahensis*. Both *Fa. suarezarum* and *Fa. uthensis* lack the distinct openings on the caudal margin of the interfenestral bar for the external nares and the caudomedial maxillary fenestra (=interfenestral canal) that are visible laterally through the antorbital fenestra in the troodontids *B. jaffei* [38] and possibly *Urbacodon* sp. (fig 2a–b of [61]). In *Fa. utahensis*, the antorbital sinus deeply incises the interfenestral bar rostroventrally, forming a large V-shaped pocket separated from the epiantral recess and maxillary antrum by a caudally inclined caudal surface of the interfenestral bar (Fig 10). This differs from *Fa. suarezarum*, where the thickened interfenestral bar houses the maxillary antrum at the same level, excluding the antorbital sinus (Fig 9). The nature of the caudomedial maxillary fenestra is unclear in both *Fa. utahensis* and *Fa. suarezarum*, but it appears absent on the

preserved areas of the interfenestral bar in both specimens and was likely ventromedially located (if present). This differs from *Fu. paradoxus* and tyrannosauroids, in which the rostroventral extent of the antorbital sinus is bound by a thin vertical postantral strut and interconnects with the maxillary antrum via an oval-shaped caudomedial maxillary fenestra [73,74,79]. A caudomedial maxillary fenestra is also present in *H. sollers* and some ornithomimosaurians, but the maxillary antrum is apparently unenclosed in these taxa [90]. A caudomedial maxillary fenestra is apparently absent in *O. hermanni* and *M. bicentesimus* [74].

Lacrimal

An incomplete L-shaped element, CEUM 78177, is tentatively identified as a right lacrimal (Fig 11). It possesses an elongated rostral process (damaged distal tip), a short caudal process (nearly complete), and a subvertical ventral process (damaged distally). The proportions of these processes are similar to tyrannosauroids [104,105], ornithomimosaurians [106], *Aorun* [91], and therizinosaurians [55,58,59], but unlike the T-shaped lacrimal of many paravians and some oviraptorosaurs [24,31,38,107].

Fig 11. Possible lacrimal of *Falcarius suarezarum*. Right lacrimal CEUM 78177 in dorsal (A), rostral (B), lateral (C), caudal (D), medial (E), and ventral (F) view. Scale bar 1 cm.

The rostral process is damaged at its rostral end, but it is apparently elongated and proportionally similar to the ventral process, as in tyrannosauroids [104,105], ornithomimosaurs [106,108], therizinosaurians [55,58], and deinonychosaurs [31,109], but unlike the abbreviated rostral process of *Incisivosaurus gauthieri* and other oviraptorosaurs [92,107]. The dorsal margin

of the rostral process is unusually arched dorsally above the level of the caudal process, more similar to early diverging tyrannosauroids [104,105,110], *Shenzhousaurus orientalis* [111], *Aorun zhaoi* [91], and *Ornitholestes hermanni*, but unlike the straight rostral process of other ornithomimosaurians [106], other alvarezsaurians [90,112], other therizinosaurians [59,79], and troodontids [37,38,113]. The primary axis of the rostral process forms an obtuse (~108°) angle with the primary axis of the ventral process. This is slightly larger than the angle of these processes in *Jianchangosaurus yixianensis* (~94° [58]), *Fukuivenator paradoxus* (~90°; fig 6j of [79]), *Ornitholestes hermanni* (~93°), and *Hesperornithoides miessleri* (~94°; fig 8 of [47]) but much larger the acute angle of *Zuolong sallei* (66°; fig 2 of [114]), *Garudimimus brevipes* (~84°; fig 2 of [106]), *Haplocheirus sollers* (~55°; fig 3 of [90]), *Erlikosaurus andrewsi* (~85°; fig 5c of [59]), *Sinovenator changii* (~77°; fig 5 of [113]) and *Byronosaurus jaffei* (~74°; fig 3 of [38]). The dorsal surface of the rostral process is smooth.

The rostral process tapers distally and forms the caudodorsal margin of the antorbital fenestra (Fig 11A–C). The lateral surface of the rostral process is unusually flat and contiguous with the lateral surfaces of the caudal and ventral processes. The lateral surface at the lacrimal angle is unperforated in CEUM 78177, and there is no enlarged external lacrimal fenestra or foramen as in some tetanurans [101], tyrannosauroids [95,110], *Ornitholestes hermanni*, *Incisivosaurus gauthieri* [92], and *Fukuivenator paradoxus* [79], or a small foramen as in *Nqwebasaurus* [108], dromaeosaurids [109,115], and troodontids [113]. Despite the presence of a caudal opening for the nasolacrimal duct (Fig 11D), there is no evidence for the cranial opening on the preserved lateral or medial surface. Moreover, the broken rostral process apparently lacks the rostral aperture present in *Erlikosaurus andrewsi*, which connects the rostral opening of the lacrimal sinus with the caudal nasolacrimal duct (“lacrimal foramen” of

Lautenschalger et al. [59]). This suggests that the internal lacrimal sinus in *Fa. suarezarum* (its presence inferred by the caudal nasolacrimal opening) is restricted and terminates within the rostral process, but CT data are necessary to confirm these interpretations.

The caudal process is the shortest of the three lacrimal processes. It tapers to a point, as in *Z. salleeii* [114], early alvarezsaurians [90,91], and *Shenzhousaurus orientalis* [111], and it is damaged distally. The caudal process overhangs the dorsally positioned circular opening of the nasolacrimal duct (Fig 11). Caudomedially, a roughened but damaged area may represent a portion of the prefrontal contact; the proportions and position of this contact are similar to some tetanurans [101] and tyrannosauroids [93]. If our interpretation of this contact is correct, it differs from the ventrally extensive lacrimal-prefrontal contact in *Garudimimus brevipes* [106] and *A. zhaoi* [91], and from the more dorsally oriented contact in *E. andrewsi* [59].

The ventral process is damaged distally but would have formed a vertically oriented preorbital bar (Fig 11). The preserved portion is relatively straight and rostrocaudally longer than transversely wide, forming a subtriangular cross-section. A well-defined ridge emarginates the antorbital fenestra rostrally and an oblique-caudally facing surface demarcates the orbit. Although the dorsal portion of the ventral process is straight, the degree to which the ventral portion twists (minor [58,79] or none [59] in therizinosaurians, completely everted in troodontids [113]) and expands to contact the jugal and/or maxilla is impossible to discern.

Frontal

A right frontal, CEUM 52574, is well-preserved but eroded around the rostral, orbital, and caudal margins (Fig 12). The frontal is rostrocaudally elongated and subrectangular in dorsal and ventral views, similar to *Fukuivenator paradoxus* [79]. An open midline sutural surface

suggests it was unfused to its missing contralateral frontal. The dorsal surface of the frontal is slightly depressed at about the level of the midorbit, as in early tyrannosauroids, *Fa. utahensis* [53,60] and *Jianchangosaurus yixianensis* [58], and similar to the flattened surface of *Beipiaosaurus inexpectus* [57], but unlike the comparatively domed condition of *E. andrewsi* [59]. This depression is not longitudinally extensive, nor is there a longitudinal midline ridge as in some early tyrannosauroids (*Guanlong* IVPP V 14531) and troodontids [31,113].

Fig 12. Frontal of *Falcarius suarezarum*. Right frontal CEUM 52574 in dorsal (A), caudal (B), lateral (C), rostral (D), ventral (E), and medial (F) views. Scale bar is 1 cm.

Rostrally, a caudally tapering slot at the craniolateral margin likely represents a portion of the prefrontal contact (Fig 12A, 12C, 12D). The preserved portion is restricted to the dorsolateral aspect of the frontal, similar to *Fu. paradoxus* [79] and *H. sollers* [90], but unlike the ventrally contained prefrontal contact in *E. andrewsi* [59]. The caudal extent of the prefrontal contact occurs near the level of the rostral margin of the olfactory fossae, which is similar to *Fu. paradoxus* and *E. andrewsi* [59,79]. The remaining rostral portion of the frontal is damaged, and the contact with the nasal cannot be assessed. The orbital margin is damaged, but it appears to have formed a dorsally elevated supraorbital ridge and contributed to a large, rounded orbit, as in *Fa. utahensis, I. gauthieri*, and many maniraptorans [53,60,79,90,92].

The postorbital process is damaged caudally and laterally, and the contact with the parietal cannot be ascertained. The rostral preserved portion of the postorbital process is emarginated by a prominent concave ridge that denotes the rostral border of the subtriangular frontoparietal fossa [116]. This strong emargination is similar to *Fa. utahensis* [60], *Fu.*

paradoxus [79], and some early diverging tyrannosauroids [105] and alvarezsaurians [117] but reduced in troodontids [113] and absent in later diverging therizinosauroids [55,59] and ornithomimosaurians [106]. The position and morphology of the postorbital contact is virtually identical to *Fa. utahensis* [53,60]; it is a small, dorsolaterally oriented, oval-shaped pit that occurs rostral to the rostral emargination of the frontoparietal fossa.

Ventrally, a well-developed crista crania is kinked at its midpoint, as in *Fa. utahensis* and some troodontids [31,50,53,60], but unlike the rounded margin of *Fu. pardoxus* and *E. andrewsi* [59,79]. This ridge separates the orbital and endocranial portions of the frontal. The proportions of the cerebral and olfactory fossae are similar to *Fa. utahensis*, and the lateral extent of the olfactory fossa approaches but does not reach the lateral extent of the cerebral fossa as in this taxon [50,53,60]. Caudally, the crista crania gives way to a flattened (but damaged) surface, which resembles the laterosphenoid contact of *Fa. utahensis* [60].

Postorbital

Two right postorbitals are represented: CEUM 73787 and CEUM 73943 (Fig 13). As is typical of theropods, they possess a rostral frontal process, a caudal squamosal process, and a ventral jugal process, although all are variably damaged distally in both specimens.

Fig 13. Postorbitals of *Falcarius suarezarum*. Right postorbital CEUM 73943 (A) and right postorbital 73787 (B) in dorsal (A1, B1), lateral (A2, B2), rostral (A3, B3), medial (A4, B4), caudal (A5, B5), and ventral (A6, B6) views. Scale bar 1 cm.

The frontal process is best preserved in CEUM 73943, but its distal contact with the frontal is damaged and missing. It is the most robust and transversely widest of the three processes. The lateral surface has a mildly rugose texture, and the base expands laterally, similar to some *Fa. utahensis* postorbitals [60]. The dorsal margin of the frontal process near the level of the laterosphenoid contact is sinuous in *Fa. suarezarum* (CEUM 73942), whereas this same margin is straight and gracile in *Fa. utahensis* (NCSM 39106, 39093, UMNH VP 14567). An elongated fossa occurs on the dorsomedial surface of the frontal process, similar in position and proportion to *Fa. utahensis* [60]. The laterosphenoid contact, present in both CEUM 73797 and CEUM 73943, occurs at the proximal end of this fossa and is represented by an oval-shaped concavity along the medial orbital margin, as in *Fa. utahensis* [60]. The dorsal rim of the laterosphenoid contact extends further medially than the ventral margin. As in *Fa. utahensis* and other theropods, a small foramen for the temporo-orbital vasculature occurs caudal to the laterosphenoid contact (Fig 8) [118]. At the base of the frontal process along the orbital margin, the bone is transversely swollen (CEUM 73943) or expanded into a small shelf (CEUM 73787) (Fig 13). The degree of transverse inflation in both *Fa. suarezarum* specimens is greater than smaller specimens of *Fa. utahensis*, but less than the largest specimen NCSM 39093 [60].

In both postorbitals of *Fa. suarezarum*, the dorsal margin between the frontal and squamosal processes is nearly straight, which is similar to *Ornitholestes hermanni* and earlier diverging coelurosaurians but differs from all known *Fa. utahensis* postorbitals and most maniraptorans, which exhibit a dorsally elevated frontal process [24,31,60,90].

The squamosal process is the smallest and most gracile of the three processes, similar to *Fa. utahensis* [60] and *H. sollers* [90], but unlike *O. hermanni* (cast YPM 53262) and early tyrannosauroids [104] in which the frontal and squamosal processes exhibit subequal

proportions. Although broken in both specimens, it seems to taper distally to contact the missing squamosal. The ridge that forms the dorsal margin of the squamosal process is continuous with the straight ridge that delineates the dorsal margin of the frontal process. In CEUM 73787, a small foramen occurs on the lateral surface where the squamosal process meets the main body of the postorbital, but this is absent in CEUM 73943 (Fig 13B). On the medial surface of the squamosal process, a well-developed ridge denotes the circumorbital membrane attachment [118], emanating from the dorsal margin and sweeping rostroventrally to merge with the orbital margin at the base of the jugal process (Fig 13A4, 13B4). In specimens of *Fa. utahensis*, this ridge forms a broader angle and aligns with the ventral margin of the base of the squamosal process [60]. This ridge complex is well developed in both *Fa. suarezarum* specimens and is similar to the prominence of the *Fa. utahensis* postorbital NCSM 39106, whereas the development of this ridge is otherwise individually variable in *Fa. utahensis* [60].

The jugal process is nearly complete in CEUM 73943. Distally, it tapers in lateral and medial view but maintains a relatively consistent transverse width of the orbital surface until it tapers at the level of the jugal contact. In medial view, the rostral margin of the jugal process obscures the orbital surface. This contrasts with specimens of *Fa. utahensis*, where the rostral margin of the jugal process is caudally displaced such that the orbital surface is just visible in medial view [60]. The caudal margin of this process is demarcated by a prominent longitudinal ridge that is confluent with the ventral margin of the squamosal process and forms a subrectangular, gently rounded rostrodorsal border of the infratemporal fenestra. The jugal process appears relatively straight and vertically oriented in *Fa. suarezarum*, similar to *O. hermanni* (cast YPM 53262), *I. gauthieri* [92], and *Ga. brevipes* [106], but unlike the rostroventral orientation in *Fa. utahensis* [60], *H. sollers* [90], and early tyrannosauroids

[104,105]. The jugal contact occurs medial to the caudal ridge on the jugal process as an elongated tapering groove, similar to *Fa. utahensis* [60].

Quadrate

CEUM 74773 is a left quadrate that is well-preserved but missing the squamosal capitulum (Fig 14). It is morphologically similar to the quadrate of *Fa. utahensis,* and similar in size to the larger *Fa. utahensis* specimens NCSM 39072 and UMNH VP 14558. The main shaft is gently arched caudally, similar to *Fa. utahensis* [53,60] but greater than *Fu. paradoxus* [79] and less than *E. andrewsi* [59]. The mandibular condyles likely would have been slightly rostrally displaced relative to the squamosal capitulum. The edges of the pterygoid flange are eroded, but it is well-preserved three-dimensionally, rostrally oriented, D-shaped in lateral view, and possesses a fossa at its base on the medial surface (which probably accepts the quadrate wing of the pterygoid), similar to *Fa. utahensis* [60]. Just below the ventral margin of the pterygoid flange is a poorly developed medial pneumatic fossa (*sensu* [119]) in a nearly identical position as *Fa. utahensis* and similar to some late-branching tyrannosaurids [93]. This contrasts the apneumatic quadrate of *Fu. paradoxus* [79], *Aorun zhaoi* [91], and early tyrannosauroids [120,121], and the shallow “external pneumatic chamber” in *E. andrewsi* that occurs on the lateral side of the pterygoid flange [59]. On the lateral surface, the ventrally projecting process that would likely enclose the quadrate foramen (together with the quadratojugal) is damaged, but the dorsal and medial preserved portions of the quadrate foramen indicate it is dorsoventrally tall and transversely thin, as in *Fa. utahensis*. A caudal pneumatic recess occurs dorsal to the quadrate foramen, similar to *Fa. utahensis* and *Fu. paradoxus* but absent in *E. andrewsi* [59,60,79]. The ventral quadratojugal contact is defined by an oval-shaped roughened concavity.

The ventral margin of the quadratojugal contact reaches the articular margin of the lateral mandibular condyle, similar to *Fa. utahensis*, but unlike the ventral extent of *Fa. paradoxus*, which does not reach the articular margin of the mandibular condyle [79].

Fig 14. Quadrate of *Falcarius suarezarum*. Right quadrate CEUM 74773 in dorsal (A), rostral (B), lateral (C), caudal (D), medial (E), and ventral (F) views. Dashed lines indicate interpreted missing portions. Scale bar is 1 cm.

The articular surfaces of the mandibular condyles, the intercondylar area, and a portion of the shaft just dorsal to the medial mandibular condyle are damaged, but the morphology of the condyles remains intact. Two mandibular condyles are present, unlike the tricondylar condition described for *E. andrewsi* [59] and the ornithomimosaurian *Sinornithomimus dongi* [122]. Similar to *Fa. utahensis*, the lateral mandibular condyle is oval-shaped in ventral view, and its long axis is oriented obliquely (rostromedial–caudolateral) with respect to the rostrocaudal axis [60]. It is smaller than the square-shaped, medially inclined medial mandibular condyle. The intercondylar sulcus appears well developed (more so than in early tyrannosauroids [121] and *H. sollers* [90]), but its prominence is taphonomically enhanced due to damage in this area. *Fa. suarezarum* differs from *Fa. utahensis*, all other therizinosaurians, and many other theropods except the tyrannosauroids *Tanycolagreus topwilsoni* (fig 2.4 of [110]) and *Eotyrannus langi* (fig 10 of [121]) in that the medial mandibular condyle projects ventrally, well past the distal extent of the lateral mandibular condyle (Fig 14B, 14D).

Braincase

Two fragmentary elements, CEUM 77254 and CEUM 73788, are the only known elements that likely pertain to the braincase of *Fa. suarezarum*.

CEUM 77254 is a partial basisphenoid that preserves both basipterygoid processes, part of the right basipterygoid recess (left is broken and missing), and part of the basisphenoid recess (Fig 15A–G). Most of the dorsal aspect is missing, including any contacts with the prootic and/or laterosphenoid. The basipterygoid processes are rostroventrolaterally directed with dorsoventrally flattened, oval-shaped articular surfaces (the articular surface of the left basipterygoid process is damaged; Figs 15B, 15D, 15F). The morphology of the articular surfaces are similar to *Fu. paradoxus* but less bulbous than those of tyrannosaurids (Fig 15F) [123]. The preserved rostral portion of the basisphenoid lacks the prominent interbasipterygoid lamina of tyrannosaurids [123]. The observable base of the basipterygoid recess is highly pneumatic (similar to *Fa. utahensis* but more extensively pneumatized than *Fu. paradoxus*, *H. sollers*, and *I. gauthieri* [79,90,92]), invading the dorsal part of the basipterygoid process rostrally (Fig 15B, D). Taphonomic damage obscures the morphology of the dorsal aspect, and it is unclear if discrete pneumatic cavities were present, as in *Fa. utahensis* [60,124]. In ventral view, the lateral margin of the basisphenoid that extends from the basipterygoid process to the basioccipital contact is straight, more similar to *I. gauthieri* [92] than the angled margin of *Fu. paradoxus* [79] (Fig 15C).

Fig 15. Partial basisphenoid of *Falcarius suarezarum*. (A) Hypothesized represented portion compared to holotype braincase of *Fa. utahensis*. Basisphenoid (CEUM 77254) in dorsal (B), ventral (C), right lateral (D), left lateral (E), rostral (F), and caudal view (G). Scale bars 1 cm.

The basisphenoid recess is poorly preserved in CEUM 77254. Caudally, a true bony margin may denote the caudal portion of a deep basisphenoid recess seen in *Fa. utahensis*, but this homologous point is not easily recognized on the holotype (UMNH VP 15000) or referred (UMNH VP 15001) braincases of *Fa. utahensis* (Fig 15C). If the basisphenoid recess is deep in *Fa. suarezarum* as it is in *Fa. utahensis*, it differs from the shallow structure of *I. gauthieri* and *Fu. paradoxus* [79,92,124]. The caudal aspect of the basisphenoid recess apparently lacks the sagittal longitudinal ridge present in *I. gauthieri* and *Fu. paradoxus* that divides the recess in two [79,92]; this ridge is also absent in *Fa. utahensis, H. sollers,* and tyrannosaurids [60,90,123]. Caudally, a cup-like articular surface may represent part of the basioccipital contact, a morphology that appears similar to *Fu. paradoxus* and *I. gauthieri* (Fig 15G).

CEUM 73788 (not figured) is a fragmentary bone with rounded and deep pneumatic pockets and may pertain to the braincase. The size, shape, and relative position of these pockets are reminiscent of those within the subcondylar or subotic recesses of *Fa. utahensis* [50,60,124], but a definitive anatomical assignment is difficult to justify.

Dentary

The dentary of *Fa. suarezarum* is best represented by three well-preserved specimens: CEUM 52511 (left), CEUM 77263 (left), and CEUM 81539 (right) (Fig 16). These specimens are relatively yet variably complete, and they are all missing the caudal aspect. Several other fragmentary dentaries are also known (CEUM 73644, 77238, 81532, 73929, 53258), but they yield little additional morphological information.

Fig 16. Dentaries of *Falcarius suarezarum*. Left dentary CEUM 52511 (A), left dentary CEUM 77263 (B), and right dentary CEUM 81539 (C, reversed) in dorsal (A1–C1), lateral (A2–C2), caudal (A3–C3), medial (A4–C4), rostral (A5–C5), and ventral (A6–C6) views. Scale bar 1 cm.

The dentary is straight to slightly bowed and deepens caudally, similar to *Fa. utahensis* [50,53,60] but deeper than early ornithomimosaurians [111,125] and early alvarezsaurians [90,91]. The rostral end of the dentary is relatively acuminate, more so than early alvarezsaurians [90,91] but to a lesser degree than early ornithomimosaurians [111,125]. The rostral dentary is straight to slightly upturned and angled medially, similar to *Fa. utahensis* [50,53,60], *H. sollers* [90], *A. zhaoi* [91], *Pelecanimimus polydon* [125], and some troodontids [31,34], but unlike the downturned rostral dentary of later diverging therizinosauroids and ornithomimosaurians [57,58,106,126]. As in *Fa. utahensis*, the symphyseal facet is rhomboidal and medially inclined (Fig 16). Similar to *Fa. utahensis* and other therizinosauroids [58,60,126], the dentary possesses a cluster of small foramina at its rostral end on the lateral and ventral surfaces, and a single row of mental foramina that line the dorsal margin just ventral to the tooth row. This differs from some troodontids, in which the mental foramina are inset into a longitudinal groove [31,37,38,45,47], or form two rows, the dorsal of which are housed in a groove [40,61,127]. The lateral surface of all known *Fa. suarezarum* dentaries lack the labial shelf that characterizes therizinosauroids [57,58,126]. Instead they are similar to the early diverging taxa *Fa. utahensis* and *Fu. paradoxus* [53,60,79].

The Meckelian groove initiates just caudal to the symphyseal facet, near the level of the fifth alveolus, similar to *Fa. utahensis*. This differs from the troodontids *Urbacodon itemirensis, Xixiasaurus henanensis*, and *Zanabazar junior* in which the Meckelian groove extends rostrally

onto the symphyseal articulation [31,37,127]. As in *Fa. utahensis*, the Meckelian groove widens between the midpoint and two-thirds caudally along the dentary into a well-developed triangular fossa that would have received the splenial [53].

The tooth row is well-preserved in all specimens. Small triangular interdental plates are present and best seen in CEUM 52511, as in other therizinosaurians, but unlike troodontids, except *Urbacodon* [34,79,126,127]. The dentary groove present on the dorsolateral dentary surface in *Fu. paradoxus* [79] and the dorsomedial surface in *Urbacodon itemirensis* [127], is absent in *Fa. suarezarum* and *Fa. utahensis* [60]. Thirty alveoli are present in the complete tooth row of CEUM 52511, and >27 and >22 alveoli are present in the incomplete dentaries CEUM 77263 and CEUM 81539, respectively. This is slightly greater than the 28 alveoli present in *Fa. utahensis* and *Jianchangosaurus yixianensis* [53,58,60], and slightly lower than some troodontids for which the complete tooth row is known (e.g., 35 in *Zanabazar junior* [31]; 32 in *Urbacodon itemirensis* [127]). This tooth count is broadly similar to those of other early diverging coelurosaurians, including early tyrannosauroids (20 in *P. bradleyi* [120]), early alvarezsaurians *H. sollers* (30–40 [90]) and *Aorun zhaoi* (25–30 [91]), and later diverging therizinosauroids (variably 31–40; [57,58,126]). However, tooth counts in *Fa. suarezarum* and other early therizinosaurians are greater than those of other early diverging maniraptoriforms, including *Ornitholestes hermanni* (11), the early diverging oviraptorosaurian *Incisivosaurus gauthieri* (nine [92]), and the early diverging ornithomimosaurians *Harpymimus okladnikovi* (estimated 11 [128]) and *Shenzhousaurus orientalis* (estimated eight [111]).

Dentition

Maxillary dentition

CT scans reveal previously unrecognized replacement teeth present in the maxilla (Fig 17). Near-complete crowns are present in the fourth, seventh, and tenth preserved alveoli, capturing some positional variation in crown morphology. Although incomplete, the series indicates a general trend of decreasing overall crown size and decreasing crown height (i.e., stouter proportions) distally along the tooth row. This is similar to the therizinosaurians *Fa. utahensis*, *J. yixianensis*, and to a lesser extent *E. andrewsi* [53,55,58,60], but differs from the troodontids *Xixiasaurus henanensis, Sinovenator changii, Sinusonasus magnodens, Zanabazar junior*, and *Saurornithoides mongoliensis*, which exhibit subequal or increasing crown size distally along the toothrow in comparable positions [31,37,39,45].

Fig 17. Maxillary dentition of *Fa. suarezarum*. (A) Reference image of tooth view orientations and annotations. (B) Left maxilla CEUM 73719 showing preservation and position of replacement tooth crowns. CT renders from the best-preserved tooth crowns of the fourth (C), seventh (D), and tenth (E) preserved alveoli. Scale bar 1 cm (B) and 1 mm (C–E).

The maxillary tooth crowns of *Fa. suarezarum* are labially convex and lingually concave, as in the early therizinosaurians *Fa. utahensis* and *J. yixianensis* [53,58,60], but unlike the more symmetrical, convex labial and lingual surfaces of the troodontids *Daliansaurus liaoningensis, X. henanensis*, *Z. junior*, *Sa. mongoliensis*, and the Morrison Formation tooth taxon *Koparion douglassi* [31,37,42,48]. The distalmost preserved crown exhibits greater labial concavity than the preceding teeth (Fig 17E). In labial and lingual views, the crowns are lanceolate [129] with distinct inflection points along the mesial and distal carinae. The crowns have an asymmetrical profile, such that the inflection point along the mesial carina is apically offset from the infection

point on the distal carina, which is similar to *Fa. utahensis* and *J. yixianensis*, but unlike the more symmetrical crowns of *E. andrewsi* [53,55,58,60]. Most troodontids possess ziphodont to foliodont teeth that lack a distinct inflection on the mesial and distal carinae [31,37,38], but in those taxa that possess carina inflection points, they are more symmetrical and occur at the same apicobasal level (e.g., *Urbacodon;* fig 2 of [127], fig 4 of [61]). The teeth of *Fa. suarezarum* are constricted at the base, a common feature in therizinosaurians, troodontids, and a variety of other theropods [53,58,75,130].

The maxillary teeth of *Fa. suarezarum* are slightly recurved and possess a prominent median ridge on the lingual surface that extends from the apex to the crown base, forming two longitudinal concavities on either side, similar to *Fa. utahensis* [60]. The median ridge abuts a distally displaced basal swelling that extends from the crown base apically to about one-third of the crown height at the level of the inflection point of the distal carina. The basal swelling appears to diminish in prominence distally along the toothrow, and it is nearly absent in the last preserved crown (Fig 17E). This gives the crown base of the fourth preserved tooth an asymmetrical, pinched, oval-shaped cross-section, the seventh tooth a labiolingually wide J-shaped or lanceolate cross-section, and the tenth tooth a comparatively labiolingually compressed lanceolate cross-section (Fig 17C–E) [129,130]. Few definitive therizinosaurian maxillary teeth are available for comparison, but this combination of a median ridge and basal swelling is present in *Fa. utahensis* [60], as well as the dentary teeth of *J. yixianensis* [58] and the controversial, possibly anachronistic therizinosauroid *Eshanosaurus deguchiianus* [130–132]. The median ridge and basal swelling are less well-developed in the maxillary teeth of *Erlikosaurus andrewsi* [55]. Although a median ridge is noticeable in the maxillary teeth of some troodontids, it is typically strongly caudally angled towards the apex (recurved foliodont *sensu*

[129]), unlike the gentle recurvature in *Fa. suarezarum*; moreover, troodontids lack an accompanying swelling at the distal crown base [31,37].

The CT scans did not provide high enough resolution to definitively characterize denticle morphology. However, the presence of other fine-scale features (median ridge, basal swelling) suggests the absence of large denticles is a true morphological feature. Small denticles occur on the maxillary teeth of early diverging therizinosaurians [53,58,60,89] and some early diverging troodontids [39,42,45,47], whereas they are absent in other troodontids [15,33,34,37,38,43]. This contrasts with many Late Cretaceous, late-diverging troodontids that possess large, apically oriented denticles restricted to the distal carina [14,31]. In *Fa. suarezarum*, minor topographic variations are discernible apically along the mesial and distal carina until their respective inflection points likely represent small denticles (Fig 18); if interpreted correctly, this is nearly identical to the morphology of the early therizinosaurians *Fa. utahensis*, *J. yixianensis*, and STM 31-1 [53,58,60,89], but unlike troodontids with denticulate teeth, in which denticles extend to the level of the cervix [130].

Fig 18. Maxillary teeth of *Falcarius utahensis* and *Fa. suarezarum*. (A) NCSM 39070, *Fa. utahensis* maxillary tooth in mesial view. (B) NCSM 39073, *Fa. utahensis* maxillary tooth in mesial view. (C) CT rendering of seventh preserved maxillary tooth of *Fa. suarezarum* in mesial view. Scale bar is 1 mm.

Dentary dentition

The available dentary detention in *Fa. suarezarum* is broadly similar to that of *Fa. utahensis* and other early diverging therizinosaurians [53,58,60,75]. The rostral dentary dentition

is poorly preserved on all available specimens, but available crowns appear conical and adenticulate to at least the fifth alveolus, as in *Fa. utahensis* [53,60]; this is best seen in the exposed portion of the fifth crown of CEUM 52511 (Fig 19). These rostralmost teeth do not appear procumbent, as in *Masiakasaurus knopfleri* [133], *Proceratosaurus bradleyi* [120], and *O. hermanni* (cast YPM 53262). Mid-dentary dentition is best represented in CEUM 77263 (Fig 19). The mid-dentary teeth are asymmetrical in labial and lingual profiles, possessing an expanded distal carina and a reduced mesial carina as in *Fa. utahensis*. Serrations are present on both the mesial and distal carinae, but they are smaller and terminate midway along the mesial carina, whereas they are larger and more basally extensive along the distal carina (Fig 19). The lateral surface is convex and smooth. A prominent median ridge extends from the apex to the crown base, bisecting the lingual surface and merging with an inflated basal swelling. The crown base is constricted, a feature seen in other therizinosaurians and troodontids [53,58,75,126,130]. A single tooth crown is visible in the 29th tooth position of CEUM 52511 and best represents the distal dentary dentition (Fig 19C). This crown is overall smaller compared to more mesial tooth positions but maintains a similar morphology, following patterns of *Fa. utahensis*.

Fig 19. Dentary dentition of *Fa. suarezarum*. (A) Representative left dentary (CEUM 52511) in medial view, highlighting dentition positional variation (inset boxes correspond to numbers in figure). (B) Rostral left dentary dentition of CEUM 52511 with fragmentary teeth in positions d1, d3, and d5 in lingual view. (C) 29th tooth position of left dentary CEUM 52511 in lingual view. (D) Representative mid-dentary dentition of left dentary CEUM 77263 in lingual view. All scale bars 1 mm.

General dentition patterns

The features of both the maxillary dentition and dentary dentition in *Fa. suarezarum* (asymmetrical lanceolate profile, convex labial and concave lingual surfaces, lingual median ridge, distally offset basal swelling, minute apically-restricted denticles) differ from the labiolingually compressed, recurved morphology in the North American Jurassic paravian *Hesperonithoides miessleri* [47] and the symmetrical, labially and lingually convex crown of *Koparion douglassi* that lacks a median ridge and possesses large, basally extensive denticles [47,48]. Moreover, the teeth of *Fa. suarezarum* lack the large, apically oriented denticles on the distal carina characteristic of many Late Cretaceous troodontids [14,31]. Compared to troodontids that lack denticles (e.g., *Mei, Xixiasaurus, Byronosaurus, Gobivenator, Almas*), the teeth of *Fa. suarezarum* are stouter, lack a pointed and/or strongly recurved apex, and possess a proportionally large basal swelling (which is otherwise absent in troodontids [15,33,34,37,38].

Principal Component Analysis (PCA)

Our PCA of log-transformed linear measurements of coelurosaurian maxillae recovers different clades occupying distinct regions of morphospace along the first two principal component axes (Fig 20). A notable exception to this pattern is the significant shared region of morphospace among early diverging therizinosaurs, troodontids, the early alvarezsaurian *Haplocheirus sollers*, and *Falcarius suarezarum* (Fig 20). Notably, *Fa. suarezarum* falls directly adjacent to *Fa. utahensis* maxilla NCSM 39118. The first principal component has positive loadings for all measured variables, suggesting that size is the primary source of variation along this axis (55.1%) and that this axis may be uninformative. The highest positive loadings for principal component 1 relate to the proximal and distal height of the rostral body (AntRH.d,

AntRH.p), and the height of the ventral margin rostrally and caudally (VentMH.ant, VentMH.post). The second principal component captures variation (20.7%) primarily in the length of the first nine alveoli (L9Alv, which possesses the lowest negative loading score) and secondarily in the size of the maxillary fenestra (maxillary fenestra long [MxFen.L] and short axis [MxFen.S] have the highest positive loading scores). The third principal component (6.1%) similarly relates to the size of the maxillary fenestra, its distance from the rostral margin of the antorbital fossa (AntAF-MxFen), and the length of the antorbital fossa (AntFL). Edentulous ornithomimosaurs occupy a distinct area of morphospace, whereas the hyperdentigerous *Pelecanimimus polydon* plots closer to other tooth-bearing coelurosaurs. Therizinosaurians and troodontids occupy a similar range along PC 2, despite some variation along PC 1 and PC 3. Of all plotted clades, dromaeosaurids and early diverging tyrannosauroids occupy the narrowest region along PC 2, and dromaeosaurids occupy the broadest region along PC 1 and PC 3. *Incisivosaurus gauthieri* and *Ornitholestes hermanni* fall next to one another along the first two principal component axes, near the edges of both dromaeosaurid and troodontid morphospace and closest to the microraptorines *Sinornithosaurus mellienii* and *Shanag ashile*, and the juvenile *Bambiraptor feinbergi* (AMNH FARB 30556).

Fig 20. Log-transformed linear measurements of select coelurosaur maxillae plotted in principal component space. (A) PC 1 on horizontal axis, PC 2 on vertical axis. (B) PC 3 on horizontal axis, PC 2 on vertical axis. Note little overlap in morphospace among clades, apart from early diverging therizinosaurians and troodontids. Images of *Fa. suarezarum, Fa. utahensis,* and *Z. junior* are not to scale. Avialan, dromaeosaurid, and tyrannosauroid silhouettes accessed under public domain (CC 1.0), ornithomimosaur by Jagged Fang Designs (CC 4.0),

therizinosaur by Scott Hartman (CC 3.0; https://creativecommons.org/licenses/by-nc-sa/3.0/), and troodontid by Jason Hogan (used with permission).

When measurements are scaled to maxilla height to correct for size-related bias, similar patterns of morphospace occupation occur (Fig 21). In these iterations, the significant shared morphospace between early therizinosaurians and troodontids is maintained, with additional overlap among avialans and partial overlap among early tyrannosauroids (Fig 21). The alvarezsaurian *Haplocheirus sollers* occurs just outside of troodontid and therizinosaur morphospace. Length-related features have the highest positive loadings on PC 1 (60.7%), including overall maxilla length (MxL), length of the antorbital fossa (AntFL) and fenestra (AntFenL), and length of the rostral ramus (AntRL). Accordingly, the long and low maxilla of *Pelecanimimus polydon* and *Byronosaurus jaffei* plot furthest positive along PC 1, whereas the short and tall maxillae of some eudromaeosaurians, *Ornitholestes hermanni*, and *Incisivosaurus gauthieri* are furthest negative along PC 1. Ornithomimosaurs and troodontids exhibit the greatest breadth across PC 1, whereas tyrannosauroids display the least. The length of the first nine alveoli (L9Alv) is the highest positive loading along PC 2 (16.6%). Edentulous ornithomimosaurs and denticulate dromaeosaurids occur on opposite ends of PC 2, both distinct from all other coelurosaurian clades. Ornithomimosaurs and dromaeosaurids possess the greatest breadth along PC 2, whereas therizinosauroids and early tyrannosauroids exhibit the least. The third principal component (9.4%) captures variation primarily in the length of the antorbital fossa (AntFL). When plotted against PC 1, there is significant overlap among all clades, with notable exceptions being *B. jaffei*, *P. polydon*, and *I. gauthieri*. *Falcarius suarezarum* occurs near *Fa.*

utahensis maxilla NCSM 39000 but just outside of therizinosaurian, troodontid, and avialan morphospace.

Fig 21. Maxilla-height corrected measurements of coelurosaurian maxillae plotted in principal component space. (A) PC 1 on horizontal axis, PC 2 on vertical axis. Note proximity of *Fa. suarezarum* (13) and *Fa. utahensis* (14), prominent overlap of therizinosaurians, troodontids, and avialans, and minor overlap of tyrannosauroids. (B) PC 1 on horizontal axis, PC 3 on vertical axis. Note shared space of all major coelurosaurian clades and *Fa. suarezarum* (13) sits adjacent to *Fa. utahensis* (15), just outside of therizinosaurian, avialan, and troodontid morphospace. Image credit same as Fig 20.

Phylogenetic results

With these updated anatomical observations, we tested the broader evolutionary relationships of *Falcarius suarezarum* in a series of phylogenetic analyses. Under almost all iterations of analysis or matrix, we recovered *Fa. suarezarum* as the sister taxon to *Fa. utahensis* at the base of Therizinosauria (Figs 22, 23, Supporting Information). The only exception to this topology is when only the *Fa. suarezarum* holotype maxilla (CEUM 73719) was scored and run under parsimony optimality using the recent therizinosaur-focused matrix of Kobayashi et al. [80] (a derivative of Zanno's [75] matrix). In this analysis, a 50% majority-rule consensus tree places *Fa. suarezarum* outside of Therizinosauria in an unresolved polytomy with *Fukuivenator paradoxus*, *Coelurus fragilis*, *Ornitholestes hermanni*, Alvarezsauria, Oviraptorosauria, and Therizinosauria (a strict consensus tree is even more poorly resolved; Supporting Information). Scoring all cranial elements for *Fa. suarezarum* into the Kobayashi et al. [80] matrix results in a

sister taxon relationship of *Fa. utahensis* and *Fa. suarezarum* in both 50% majority-rule and strict consensus trees (Supporting Information). Notably, there were no analyses in which we recovered CEUM 73719 within Troodontidae.

Fig 22. Hypothesized evolutionary relationships of *Falcarius suarezarum* (holotype maxilla only) under parsimony optimality. Strict consensus of 70 MPTs (99,999 trees post-TBR branch swapping; length = 3395, consistency index = 0.319, retention index = 0.764). Numbers at nodes indicate Bremer support values. Note that collapsed clades here consist of several individual OTUs included in the analysis but are abbreviated for visual ease in the figure; a complete tree is available in the Supporting Information. *Suzhousaurus* and small troodontid silhouettes used under CC 1.0, large troodontid by Jason Hogan (used with permission), *Falcarius* modified from original by Scott Hartman and used under CC 3.0 (https://creativecommons.org/licenses/by-nc-sa/3.0/).

Fig 23. Hypothesized evolutionary relationships of *Falcarius suarezarum* (holotype maxilla only) under Bayesian optimization. Time-calibrated, full compatibility consensus tree using the TWiG matrix of Pei et al. [11]. Numbers at nodes indicate posterior probabilities expressed as percentages. A, Coelurosauria; B, Tyrannosauroidea; C, Compsognathidae; D, Maniraptoriformes; E, Ornithomimosauria; F, Maniraptora; G, Alvarezsauria; H, Therzinosauria; I, Therizinosauroidea; J, Therizinosauridae; K, Pennaraptora; L, Oviraptorosauria; M, Paraves; N, Deinonychosauria; O, Avialae; P, Dromaeosauridae; Q, Troodontidae. *Suzhousaurus* and small troodontid silhouettes used under CC 1.0, large troodontid by Jason Hogan (used with

permission), *Falcarius* modified from original by Scott Hartman and used under CC 3.0 (https://creativecommons.org/licenses/by-nc-sa/3.0/).

Parsimony analysis

Using the taxon- and character-inclusive TWiG dataset of Pei et al. [11] under parsimony optimality (Fig 22), *Fa. suarezarum* occurs as the sister taxon to *Fa. utahensis* at the base of Therizinosauria. A monophyletic Therizinosauroidea and Therizinosauridae are recovered, but ingroup topology could not be resolved for the latter.

The overall tree topology does not differ greatly from other iterations of the TWiG analysis [11,77,78]. *Bicentenaria* and *Zuolong* occur as successive sister taxa and are the earliest diverging coelurosaurians [114]. The strict consensus tree did not resolve the position of Therizinosauria, which falls within Maniraptora but forms a polytomy with Alvarezsauria and a clade of *Fukuivenator* + Pennaraptora. The later-diverging placement of *Fukuivenator* differs from recent analyses that recovered it as the earliest branching therizinosaurian [79]. *Ornitholestes* and *Tugulusaurus* occur stemward of compsognathids and Maniraptoriformes; the former taxon is currently under study, and this position will likely change. In other analyses, *Tugulusaurus* has been recovered as an early diverging alvarezsaurian [117,134]. As in the analysis of Pei et al. [11], we recovered a monophyletic Deinonychosauria as sister to Avialae.

Bayesian analysis

In the time-calibrated full-compatibility tree from the Bayesian analysis, *Falcarius suarezarum* plots as the sister taxon to *Falcarius utahensis* with moderate support (77% posterior probability) near the base of a monophyletic Therizinosauria (97%), which occurs as the

outgroup to Pennaraptora (84%) within Maniraptora (Fig 23). In this analysis, the major coelurosaurian lineages are maintained as successive sister clades, as expected based on prior iterations of the TWiG data matrix [11,77,78]. From earliest to latest diverging, these include Tyrannosauroidea, Compsognathidae, Ornithomimosauria, Alvarezsauria, Therizinosauria, Oviraptorosauria, and Paraves. Within Paraves, we recover a monophyletic Deinonychosauria (Troodontidae + Dromaeosauridae) as the sister clade to Avialae (which includes Anchiornithidae), as in the parsimony analysis and the original published results of this dataset [11]. The exception to this typical clade arrangement is a poorly supported (51%) sister relationship of *Zuolong* and *Tugulusaurus*, which together form the immediate outgroup to Tyrannosauroidea with extremely poor support (26%). Similarly, *Ornitholestes* is posited the earliest-branching compsognathid with poor support (42%).

Unlike the parsimony analysis, the Bayesian analysis recovers *Fukuivenator* as the earliest branching therizinosaurian, corroborating results obtained by Hattori et al. [79]. Both Therizinosauroidea and Therizinosauridae are also monophyletic with high support (100%). Therizinosauridae is further split into two additional clades, with the three Bayanshiree taxa (*Segnosaurus, Erlikosaurus*, and *Enigmosaurus*) recovered as somewhat distantly related, unlike recent analyses that recover *Segnosaurus* and *Erlikosaurus* as sister taxa [80]. However, all node support within these two clades is low (≤ 57% posterior probability), and the half-compatibility consensus tree recovers all therizinosaurids in a polytomy (Fig S4).

Our Bayesian analysis provides estimated divergence (i.e., nodes) ages for the major coelurosaurian clades. In the full compatibility analysis, Coelurosauria is suggested to have originated ~189 Ma (181.3–197.4 Ma 95% highest posterior density (HPD)) and Maniraptora at

~179.2 Ma (173.1–185.7 Ma 95% HPD), both consistent with previous estimates of an Early Jurassic origin for these groups [85,135,136]. Similarly, we estimate the divergence of Avialae at ~169.4 Ma (165–174.5 Ma 95% HPD), consistent with other Middle Jurassic estimates for this clade [85,135,136].

Discussion

The combined anatomical, morphometric, and phylogenetic results presented herein indicate *Falcarius* (*"Geminiraptor"*) *suarezarum* is an early branching therizinosaurian, not a troodontid as originally described [13]. Therizinosaurian features present on the holotype maxilla (CEUM 73719) include: 1) large, elongated maxillary fenestra (present in early therizinosaurians [58,60,79,89], but reduced in *E. andrewsi* [59]); 2) broad palatal shelf near-level with the toothrow and lacking nutrient foramina [58–60,79]; 3) from rostral to caudal along the toothrow, maxillary alveoli are evenly distributed and maxillary teeth decrease in size [58–60,79]; and 4) apicobasally stout, lanceolate dentition with minute, apically restricted denticles, a prominent median ridge, and pronounced basal swelling distal to median ridge [58,60,130]. Although a large maxillary fenestra is also characteristic of troodontids, other traits of CEUM 73719 differ from known troodontids, which possess: 1) a broad but vaulted palatal shelf, well dorsal to the alveoli, that is typically pierced by a row of nutrient foramina [30,31,37,42,61]; 2) tightly packed rostral maxillary alveoli and sparsely distributed caudal maxillary alveoli [16,30,33,34,38,42,45,61]; and 3) apicobasally tall maxillary teeth that invariably lack a basal swelling, and variably possess a prominent median ridge and denticles (variably adenticulate [15,16,33,34,38,61,127,137], minute denticles [39,42], or large, apically hooked denticles [14,31]). The anatomical and morphometric similarities in the maxillae of early therizinosaurians

and troodontids understandably complicate taxonomic identifications based on isolated maxillary remains. Together, our results have implications for therizinosaurian and troodontid evolution and biogeography, convergence and mosaicism in coelurosaurian evolution, and the paleoecology of the earliest Cretaceous theropods in North America represented in the Yellow Cat fauna.

Intraspecific variation

The small sample sizes of cranial remains make it difficult to interpret intraspecific patterns in the cranium. However, the postorbitals display both invariable species-specific morphology and similar developmental trajectories between sister taxa *Fa. suarezarum* and *Fa. utahensis*. All referred *Fa. utahensis* postorbitals possess a concave dorsal margin between the frontal and squamosal processes, a gracile and elongated frontal process, a rostroventrally directed jugal process, and a ventrally placed circumorbital ligament ridge on the squamosal process. These differ from all *Fa. suarezarum* postorbitals (straight dorsal margin, attenuated frontal process, ventrally directed jugal process, circumorbital ligament ridge approaches dorsal margin of squamosal process) supporting the hypothesis that this represents species-level variation. However, both taxa also exhibit a laterally expanded shelf on the orbital margin that roughly corresponds to specimen size—this shelf is poorly developed in smaller specimens and becomes more prominent in larger specimens, which putatively points to ontogenetic variation (Fig 13). Despite the limited sample, this suggests the postorbital exhibits interspecific differences in morphology, yet similar developmental trajectories in two closely related species. The only other cranial element with multiple represented specimens for *Fa. suarezarum* is the dentary, which shows little morphological variation among well-preserved specimens (similar to

patterns in the dentary of *Fa. utahensis* [60]), perhaps related to constraints on the feeding apparatus. A wealth of postcranial remains provides large samples to assess morphological variation and will be addressed in a subsequent study.

Implications for Yellow Cat fauna

The re-analysis of *Fa. suarezarum* yields an additional therizinosaurian from the Early Cretaceous Yellow Cat Member of the Cedar Mountain Formation. *Falcarius utahensis* and *Fa. suarezarum* occur in the lower Yellow Cat Member but are stratigraphically separated (Fig 1). Additional possible therizinosaurian material is known from the Doelling's Bowl locality near the base of the lower Yellow Cat and may represent a third distinct taxon [138], estimated to be stratigraphically older than both *Fa. utahensis* and *Fa. suarezarum* [69]. Finally, the putative therizinosauroid, *Martharaptor greenriverensis*, is represented by a fragmentary postcranial skeleton from just above the caprock in the upper Yellow Cat Member [62,65].

These (potentially four) therizinosaurians are all apparently stratigraphically separated [65,69], and there is no evidence that any of these taxa were contemporaneous *sensu stricto*. A recent CA-ID-TIMS date from a few meters above the regional caprock marker at Utahraptor Ridge is estimated at 135.1 ± 0.3 Ma [67], implying all lower Yellow Cat taxa (and *Martharaptor* at the base of the upper Yellow Cat) are older than this date. Recent U–Pb LA-ICP-MS analyses on zircons from basal gravels just above the Morrison Formation contact at Utahraptor Ridge indicate an age of 145.8 ± 1.6 Ma, and just under four meters above these beds, age dates are estimated at 139.7 ± 2.2 Ma [66,67]. This gives a range of 2.3–9.9 Ma in just four meters of stratigraphy at the base of the lower Yellow Cat Member, which translates to extremely low average sedimentation rates of 0.0004–0.0017 mm/yr (= 404–1739 mm/Ma).

Extrapolating these average rates to the stratigraphic thickness between therizinosaurian taxa suggests a duration of 0.3–1.2 Ma between *Fa. utahensis* and *Fa. suarezarum,* and 0.6–2.5 Ma between *Fa. suarezarum* and *Marthraptor greenriverensis* (similar to estimates of Kirkland et al. [69]). The time represented between *Fa. suarezarum* and *M. greenriverensis* may be especially variable, as the regional caprock at the top of the lower Yellow Cat that separates the two is interpreted as the initiation of a Sevier rain shadow and increased aridity [64,65] and could represent hundreds of thousands to millions of years, depending on its mode of formation [139]. Explicit stratigraphic and geochronological sampling tying western Yellow Cat strata in the Green River area to eastern deposits will refine these age estimates and provide important paleoecological comparisons.

The taxonomic richness of therizinosaurians in the basal Cedar Mountain Formation is on par with therizinosaurian diversity observed in the Barremian–Aptian Jehol Biota of China (which hosts 4–5 taxa [4,56,89,140,141]) and the Cenomanian–Santonian Bayanshiree Formation of Mongolia (with at least four taxa [54,75,80,126]). Poor stratigraphic and geochronological constraints on taxa in these formations preclude confident assessments of any coeval ecological partitioning [75,126]. Nevertheless, the observed diversity in penecontemporaneous therizinosaurs is opportune for future analyses of ecomorphological disparity, isotope geochemistry, or other indicators of differential ecological and evolutionary trajectories in these taxa.

Phylogenetic implications

Nearly all iterations of our phylogenetic analyses recovered a robust sister-taxon relationship for *Fa. utahensis* and *Fa. suarezarum*, supporting our anatomical observations,

morphometric analyses, and taxonomic reassignment. Notably, there is no iteration in which CEUM 73719 was recovered within Troodontidae.

We find variation in the phylogenetic position of the enigmatic taxon *Fukuivenator paradoxus*. Originally described as an unknown early branching maniraptoran [5], *Fukuivenator* was more recently recovered as the earliest-diverging therizinosaurian [79]. We find support for the latter arrangement in our Bayesian analysis (Fig 23). However, in the parsimony analysis, we recover *Fu. paradoxus* as branching later than Therizinosauria, forming the outgroup to Pennaraptora (Fig 22). This *Fukuivenator* + Pennaraptora clade is supported by eight synapomorphies, including:

ch 10 [1] - basisphenoid recess divided into two small circular foramina (present in some dromaeosaurids, *Anchiornis*, *Chirostenotes*, and *Citipati;* other oviraptorosaurs, *Falcarius*, *Haplocheirus*, some ornithomimosaurs, and most tyrannosauroids have a single opening (10 [0])).

ch 109 [1] - fused sacral zygapophyses (present in Avialae, most paravians except *Sinovenator*, late alvarezsaurids; unfused (109 [0]) in some later avialans, therizinosaurians, ornithomimosaurians, and tyrannosauroids).

ch 137 [1] - scapula length nearly the same or shorter than humerus length (shared with most paravians, *Epidexipteryx* (at the base of Oviraptorosauria), *Erliansaurus*, *Guanlong*, and most ornithomimosaurs except *Nqwebasaurus*; earlier coelurosaurians have scapula longer than the humerus (137 [0])).

ch 183 [1] - femur, ridge on lateral surface distal to lesser and greater trochanters (homologous to the trochanteric shelf) distinctly raised from shaft as a pronounced, mound-like ridge (present

in *Avimimus* (recovered as a mid-grade oviraptorosaur)) and paravians except later diverging avialans; absent or represented by a faint ridge or rugosity (183 [0]) in all earlier diverging taxa except the problematic compsognathid *Mirischia*).

ch 345 [1] - coracoid, medial surface, area of foramen m. supracoracoideus flat to convex (present in many avialans, *Microvenator, Sinovenator,* late alvarezsaurids; strongly depressed (345 [0]) in *Falcarius*, dromaeosaurids, early alvarezsauroids, ornithomimosaurs, tyrannosauroids).

ch 460 [1] - orientation of articular surfaces between cervical vertebrae strongly slanted anteroventrally (present in tyrannosauroids, *Ornitholestes*, late branching oviraptorosaurs, deinonychosaurs, and early avialans; surfaces vertical to subvertical (460 [0]) in *Falcarius* and *Nothronychus*, compsognathids, ornithomimosaurs, alvarezsaurs, *Coelurus*, later-branching avialans).

ch 749 [1] - scapula acromion process approximately as long as or longer anteroposteriorly than deep dorsoventrally, with short reach beyond scapular blade and squared-off profile (present in oviraptorosaurs, paravians (including avialans) except Gondwanan dromaeosaurids, and ornithomimosaurs except *Nqwebasaurus*; much deeper dorsoventrally than long anteroposteriorly, generally tapering or triangular in shape (749 [0]) in tyrannosauroids, compsognathids, alvarezsaurids, late therizinosaurids).

ch 787 [0] - cervical vertebrae ventral surface of the centrum smoothly flat or concave (present in *Avimimus* and paravians except South American dromaeosaurids, and mid-grade and later tyrannosauroids; cervical centrum ventral surface with distinct depression anteriorly (at level of parapophyses) (787 [1]) in *Coelurus, Guanlong*, ornithomimosaurs, therizinosaurians, *Chirostenotes*).

There are several possible reasons for the discrepancies between our analyses and those of Hattori et al. [79]. The Pei et al. [11] dataset that we used includes a higher number of avialan taxa than most iterations of the TWiG, including that used by Hattori et al. [79]. The inclusion of these taxa (with a greater number of shared character states) may pull *Fukuivenator* outside of Therizinosauria in a position closer to pennaraptorans. We also find 43 total characters that are optimized along the branch leading to *Fukuivenator*, the highest on any single branch in the consensus tree of our analysis, and similar to the 46 characters identified by Hattori et al. [79]. In addition to the synapomorphies identified in the parsimony analysis, certain cranial features of *Fukuivenator* are more similar to early oviraptorosaurians (e.g., *I. gauthieri*) than early therizinosaurians, including a relatively short and tall maxilla (long and low in *Falcarius*, *Jianchangosaurus*, and STM 31-1), a tall and circular maxillary fenestra (elongated and ellipsoidal in *Falcarius*), a relatively low number of proportionally large teeth that lack denticles (numerous, small, denticulate teeth in *Falcarius* and *Jianchangosaurus*), and a large lacrimal foramen (absent in *Falcarius* and *Erlikosaurus*). Combined, these features may suggest closer oviraptorosaurian affinities for *Fukuivenator*, or possibly close affinities of oviraptorosaurians and therizinosaurians that were not recovered in our phylogenetic analyses. In any case, these anatomical features and phylogenetic results highlight the unique and mosaic anatomy of *Fukuivenator* [5,79], as well as the need for both refined phylogenetic character- and taxon-matrix constructions [47] and additional specimens to resolve the evolutionary history of these groups.

Body-size evolution and biogeographic implications

Our reinterpretation of *Falcarius suarezarum* has implications for global biogeographic patterns of both therizinosaurians and troodontids. The North American record of therizinosaurians maintains a >30-million-year gap between pre-Aptian Yellow Cat taxa and *Nothronychus* in the Turonian [75,142,143]. Recent advances in age-dating of the Yellow Cat Member reveal that these sequences reflect North America's oldest dinosaur faunas, with sauropod, ankylosaur, and iguanodontian lineages comparable to European "Wealden-type" assemblages [65,144,145]. Europe has previously been suggested as an important focal point in the global dispersal of several coelurosaurian lineages in the earliest Cretaceous [85]. To date, therizinosaurians are absent from these European assemblages around this time (with the possible exception of a single putative therizinosaurian tooth from the Middle Jurassic of England [146], and the enigmatic, putative oviraptorosaurian/therizinosaurian, *Thecocoelurus daviesi*, from the Barremian of England [147]), suggesting the rich therizinosaurian fauna of the Yellow Cat may reflect an endemic North American radiation. Close phylogenetic ties between *Nothronychus* and early Late Cretaceous Bayanshireenian therizinosauroids (this study; [75,80]) support an Early Cretaceous Laurasian interchange event (EKLInE) hypothesized for other dinosaurian lineages [148]. Additional discoveries and updated analyses of historically collected therizinosaurian material may help refine these biogeographic hypotheses.

Our reinterpretation of *"Geminiraptor" suarezarum* as a therizinosaurian has more profound effects on troodontid evolution and biogeography. The elimination of this Early Cretaceous troodontid taxon restricts all named North American troodontids to Campanian–Maastrichtian deposits, with isolated occurrences of possible troodontid material in some pre-Campanian units no earlier than the Cenomanian [149–152]. Putative troodontids are known from the Jurassic (Tithonian) Morrison Formation, including small-bodied *Hesperornithoides*

miessleri [47] and the tooth taxon *Koparion douglassi* [48]. Although Hartman et al. [47] recovered *H. miessleri* as a troodontid, they acknowledged that this taxon falls readily within other paravian groups in their phylogenetic analyses. Several features of this taxon are atypical of troodontids (especially those hypothesized as closely related to *H. miessleri* [47]), including a maxillary fenestra that is somewhat dorsally offset and caudally positioned relative to the rostral border of the antorbital fossa (common in dromaeosaurids [24,97]), an accessory caudoventral maxillary fossa (also in the dromaeosaurid *Zhenyuanlong* [153]), labiolingually compressed, apicobasally tall, non-isodont maxillary teeth with minute denticles (atypical of early and late-diverging troodontids, except *Xixiasaurus*), subequal robusticity of mt II and IV (atypical of reduced mt II in late-branching troodontids [46]), and distal caudal centra that lack lateral depressions (otherwise seen in some early diverging troodontids [41,44]).

If the troodontid affinities of the two Morrison taxa persist, this would leave a >45-million-year gap in the definitive troodontid fossil record on the North American continent (>65 million if only including named taxa). In the absence of "*Geminiraptor*", the global record of Early Cretaceous troodontids is restricted to small-bodied Asian forms (<5 kg) known from the Barremian-age Jehol assemblages of China [15,39,42,43,45], and the Aptian–Albian of China (*Sinornithoides youngi* [44]), Mongolia (MPC-D 100/44, 100/140 [40,41]), and Japan (*Hypnovenator matsubaraetoheorum* [46]). The elimination of the proportionally large-bodied "*Geminiraptor*" also restricts relatively large-bodied (up to ~60 kg [29]) troodontids to the Late Cretaceous of Laurasia. This further corroborates trends of generally increasing body size throughout troodontid evolution [154,155], a pattern perhaps driven by increased herbivory in these taxa [156,157]. Regardless of the taxonomic affinities of Campanian–Maastrichtian North American troodontids, they are consistently recovered as closely related to contemporaneous

larger-bodied Asian taxa (this study; [30,31]); however, isolated teeth of potential troodontid affinities have been noted from the Turonian–early Campanian Straight Cliffs Formation [152] and the Cenomanian Mussentuchit Member of the Cedar Mountain Formation [144,150], suggesting these groups are connected to a mid-Cretaceous (pre-Campanian) Laurasian interchange (e.g., [85]). Updated analyses of historically collected Aptian–Albian taxa of Asia [40,41,44] and continued discoveries of Cenomanian–Santonian taxa are key to filling gaps in our understanding of biogeographic trends, feeding ecology, and body-size evolution in troodontids.

Evolutionary trends in coelurosaurian maxillae

Our principal component analyses and comparative descriptions offer insight into the evolutionary patterns of coelurosaurian maxillae and potential paleobiological implications. In the log-transformed iteration of our PCA (Fig 20), the maxillae of early diverging members of several coelurosaurian clades plot near the origin in PCA space, including early alvarezsaurians, early therizinosaurias, and early tyrannosauroids. The early oviraptorosaur *Incisivosaurus* and the enigmatic coelurosaur *Ornitholestes* plot adjacent to one another and relatively close to these other early branching coelurosaurs. In the height-corrected analyses (Fig 21), early branching members exhibit some variation in morphospace based on the length of the rostrum; short-snouted *Incisivosaurus* and *Ornitholestes,* and long-snouted *Haplocheirus* and *Pelecanimimus* diverge from early therizinosaurians, tyrannosauroids, and avialans. Combined, this suggests a similar overall maxillary size and morphology (with some variation) near the base of major coelurosaurian clades that was subsequently modified along different lineages.

Late-diverging, large-bodied Laurasian troodontids converge on this ancestral maxillary morphology, exhibiting particularly noticeable overlap in morphospace with early diverging therizinosaurians. Our results corroborate qualitative similarities in these groups first noted by Pu et al. [58]. The similarities in the proportions of the maxilla and the distribution of the antorbital openings in both groups imply similar biological functions, likely related to dietary ecology. Although distinct from one another, the dentition of both early therizinosaurians and troodontids suggests an omnivorous to herbivorous diet [143,156–158], and similar maxillary proportions support this interpretation. Body-mass estimates based on femoral circumferences [159] are also comparable among late branching troodontids (~10 kg *Gobivenator mongoliensis* [33], ~50–60 kg *Troodon* [29]) and early branching therizinosaurians (~20 kg *Jianchangosaurus* [58], ~67 kg *Beipiaosaurus* [160]), further suggesting similar ecologies in these groups. An increasingly herbivorous diet throughout the evolutionary histories of both therizinosaurians and troodontids may have contributed to the evolution of varying body sizes and food-processing ecomorphological features in these clades.

Conclusions

1) The Early Cretaceous North American troodontid *"Geminiraptor" suarezarum* is herein reevaluated as an early branching therizinosaurian. Features used in the original diagnosis of the specimen (e.g., inflated promaxillary sinus, large maxillary fenestra, narrow interfenestral strut) are now known to characterize early diverging therizinosaurians, and CT data reveal bulbous, lanceolate, labially convex replacement teeth akin to other therizinosaurians but unlike troodontids.

2) The holotype maxilla and other therizinosaurian material from the Suarez Site are hypothesized as belonging to a single taxon, a novel combination, *Falcarius suarezarum*.
3) Anatomical differences in the maxillae, postorbitals, quadrate, and dentary dentition between *Fa. suarezarum* and *Fa. utahensis* are hypothesized as diagnostic interspecific morphological variation. Both species occur as sister taxa in phylogenetic analyses, further supporting our taxonomic reassignment.
4) Four potential therizinosaurians occur in the basalmost Cretaceous strata of North America, leaving a gap in the fossil record of this clade on the continent until the Turonian. Similarly, North American troodontids are restricted to possible Jurassic occurrences and the latest Cretaceous. European linkages that characterize other Yellow Cat dinosaurs in the earliest Cretaceous of North America may have been important to early therizinosaurians, whereas early Late Cretaceous Laurasian therizinosaurids likely dispersed via Early Cretaceous Laurasian interchange events. The close affinities of Campano–Maastrichtian troodontids in Asia and North America suggest ties to interchange events prior to this time.
5) A similar maxillary morphology, present in early diverging members of coelurosaurian lineages, is converged upon by late-branching troodontids. This suggests similar morphofunctional and biomechanical capabilities in these taxa, perhaps related to an omnivorous or herbivorous diet.

Acknowledgements

We thank the Bureau of Land Management, the Utah Geological Survey, and Jim Kirkland for permits and continued support. Thanks to the epithetical Suarez twins, Drs. Marina and Celina Suarez, for discovering the site and their continued dedication to the geology of the

Cedar Mountain Formation. Thanks to Ryan Tucker for discussions of Yellow Cat sedimentology. Thanks to Aaron Giterman, who expertly prepared NCSM 39000. For CT imaging, we thank Tyler Slater and the Preclinical Imaging Core at the University of Utah, the Analytical Instrumentation Facility at NCSU, and Haviv Avrahami for handling scan logistics and post-processing advice. Many thanks to Bridget Dale for assisting in surface scanning, and for arduous CT segmentation of NCSM 39000. Thanks to James Epps for enthusiastically segmenting paranasal sinuses. For loans, access to specimens, and other logistical help, we are extremely grateful to: Katy Cornelli, Matt Mers (CEUM); Lisa Herzog, Sean Moran, Christian Kammerer (NCMNS); Randy Irmis, Carrie Levitt-Bussian, Alyson Wilkins (NHMU); Khishigjav Tsogtbaatar, Tsogtbaatar Chinzorig, Sanjaadash Ulziitseren, Damdinsuren Idersaikhan, Chagnaa Bayardorj (IPMAS); Xu Xing, Chun-Chi Liao (IVPP); Chang Huali (HGM). Thanks to Tsogtbaatar Chinzorig for sharing specimen photos. For discussions and feedback at various steps, we are grateful to: Chun-Chi Liao, James Napoli, Haviv Avrahami, Josh Hedge, Kyla Beguesse, Tsogtbaatar Chinzorig, John Kanipe, Emi Bender, and Kyle Obuszewski. Funding was provided by the Paleontological Society, the Jurassic Foundation, the Paleontological Research Institution, the Systematics Association (WJF), and National Science Foundation award #1925973 (LEZ).

References

1. de Beer G. *Archaeopteryx lithographica*: a study based upon the British Museum specimen. No Title. 1954. Available: https://cir.nii.ac.jp/crid/1130000796727707648
2. Felice RN, Goswami A. Developmental origins of mosaic evolution in the avian cranium. Proc Natl Acad Sci. 2018;115: 555–560. doi:10.1073/pnas.1716437115

3. Clarke JA, Middleton KM. Mosaicism, Modules, and the Evolution of Birds: Results from a Bayesian Approach to the Study of Morphological Evolution Using Discrete Character Data. Syst Biol. 2008;57: 185–201. doi:10.1080/10635150802022231

4. Xu X, Tang Z, Wang X. A therizinosauroid dinosaur with integumentary structures from China. Nature. 1999;399: 350–354. doi:10.1038/20670

5. Azuma Y, Xu X, Shibata M, Kawabe S, Miyata K, Imai T. A bizarre theropod from the Early Cretaceous of Japan highlighting mosaic evolution among coelurosaurians. Sci Rep. 2016;6: 20478. doi:10.1038/srep20478

6. Xu X, Currie P, Pittman M, Xing L, Meng Q, Lü J, et al. Mosaic evolution in an asymmetrically feathered troodontid dinosaur with transitional features. Nat Commun. 2017;8: 14972. doi:10.1038/ncomms14972

7. Benson RBJ, Butler RJ, Carrano MT, O'Connor PM. Air-filled postcranial bones in theropod dinosaurs: physiological implications and the 'reptile'–bird transition. Biol Rev. 2012;87: 168–193. doi:10.1111/j.1469-185X.2011.00190.x

8. O'Connor PM, Claessens LPAM. Basic avian pulmonary design and flow-through ventilation in non-avian theropod dinosaurs. Nature. 2005;436: 253–256. doi:10.1038/nature03716

9. Lautenschlager S, Witmer LM, Altangerel P, Rayfield EJ. Edentulism, beaks, and biomechanical innovations in the evolution of theropod dinosaurs. Proc Natl Acad Sci. 2013;110: 20657–20662. doi:10.1073/pnas.1310711110

10. Xu X, Zhao Q, Norell M, Sullivan C, Hone D, Erickson G, et al. A new feathered maniraptoran dinosaur fossil that fills a morphological gap in avian origin. Chin Sci Bull. 2009;54: 430–435. doi:10.1007/s11434-009-0009-6

11. Pei R, Pittman M, Goloboff PA, Dececchi TA, Habib MB, Kaye TG, et al. Potential for Powered Flight Neared by Most Close Avialan Relatives, but Few Crossed Its Thresholds. Curr Biol. 2020;30: 4033-4046.e8. doi:10.1016/j.cub.2020.06.105

12. Pol D, Goloboff PA. The impact of unstable taxa in coelurosaurian phylogeny and resampling support measures for parsimony analyses. 2020. Available: https://ri.conicet.gov.ar/handle/11336/170477

13. Senter P, Kirkland JI, Bird J, Bartlett JA. A New Troodontid Theropod Dinosaur from the Lower Cretaceous of Utah. PLOS ONE. 2010;5: e14329. doi:10.1371/journal.pone.0014329

14. Currie PJ. Bird-like characteristics of the jaws and teeth of troodontid theropods (Dinosauria, Saurischia). J Vertebr Paleontol. 1987;7: 72–81. doi:10.1080/02724634.1987.10011638

15. Xu X, Norell MA. A new troodontid dinosaur from China with avian-like sleeping posture. Nature. 2004;431: 838–841. doi:10.1038/nature02898

16. Gao C, Morschhauser EM, Varricchio DJ, Liu J, Zhao B. A Second Soundly Sleeping Dragon: New Anatomical Details of the Chinese Troodontid *Mei long* with Implications for Phylogeny and Taphonomy. PLOS ONE. 2012;7: e45203. doi:10.1371/journal.pone.0045203

17. Varricchio DJ, Jackson F, Borkowski JJ, Horner JR. Nest and egg clutches of the dinosaur *Troodon formosus* and the evolution of avian reproductive traits. Nature. 1997;385: 247–250. doi:10.1038/385247a0

18. Hogan JD, Varricchio DJ. Do paleontologists dream of electric dinosaurs? Investigating the presumed inefficiency of dinosaurs contact incubating partially buried eggs. Paleobiology. 2021;47: 101–114. doi:10.1017/pab.2020.49

19. Hogan JD, Varricchio DJ. Chthonic severance: dinosaur eggs of the Mesozoic, the significance of partially buried eggs and contact incubation precursors. Philos Trans R Soc B Biol Sci. 2023;378: 20220144. doi:10.1098/rstb.2022.0144

20. Cau A, Beyrand V, Voeten DFAE, Fernandez V, Tafforeau P, Stein K, et al. Synchrotron scanning reveals amphibious ecomorphology in a new clade of bird-like dinosaurs. Nature. 2017;552: 395–399. doi:10.1038/nature24679

21. Gianechini FA, Makovicky PJ, Apesteguía S, Cerda I. Postcranial skeletal anatomy of the holotype and referred specimens of *Buitreraptor gonzalezorum* Makovicky, Apesteguía and Agnolín 2005 (Theropoda, Dromaeosauridae), from the Late Cretaceous of Patagonia. PeerJ. 2018;6: e4558. doi:10.7717/peerj.4558

22. Colbert EH, Russell DA. The small Cretaceous dinosaur *Dromaeosaurus*. Am Mus Novit No 2380. 1969. Available: https://agris.fao.org/search/en/providers/122376/records/6582f4f0ebe24a6dee898754

23. Gauthier J. Saurischian monophyly and the origin of birds. Mem Calif Acad Sci. 1986;8: 1–55.

24. Turner AH, Makovicky PJ, Norell MA. A Review of Dromaeosaurid Systematics and Paravian Phylogeny. Bull Am Mus Nat Hist. 2012;2012: 1–206. doi:10.1206/748.1

25. Currie PJ. Theropods, including birds. In: Currie PJ, Koppelhus EB, editors. Dinosaur Provincial Park: a spectacular ancient ecosystem revealed. Bloomington, IN: Indiana University Press; 2005. pp. 367–397. Available:

https://books.google.com/books?hl=en&lr=&id=lS55girEQsEC&oi=fnd&pg=PR13&dq=Curri+PJ.+Theropods,+Including+Birds.+Dinosaur+Provincial+Park:+A+spectacular+ancient+ecosystem+revealed.+2005:367.&ots=57fTbuahUs&sig=RPcAublprs8RZLWDUBWnW_vwnx4

26. Russell DA. A new specimen of *Stenonychosaurus* from the Oldman Formation (Cretaceous) of Alberta. Can J Earth Sci. 1969;6: 595–612. doi:10.1139/e69-059

27. Zanno LE, Varricchio DJ, O'Connor PM, Titus AL, Knell MJ. A New Troodontid Theropod, *Talos sampsoni* gen. et sp. nov., from the Upper Cretaceous Western Interior Basin of North America. PLOS ONE. 2011;6: e24487. doi:10.1371/journal.pone.0024487

28. Evans DC, Cullen TM, Larson DW, Rego A. A new species of troodontid theropod (Dinosauria: Maniraptora) from the Horseshoe Canyon Formation (Maastrichtian) of Alberta, Canada. Can J Earth Sci. 2017;54: 813–826. doi:10.1139/cjes-2017-0034

29. Varricchio DJ, Hogan JD, Freimuth WJ. Revisiting Russell's troodontid: autecology, physiology, and speculative tool use. Can J Earth Sci. 2021;58: 796–811. doi:10.1139/cjes-2020-0184

30. Varricchio DJ, Hogan JD, Gardner JD. Troodontid specimens from the Cretaceous Two Medicine Formation of Montana (USA) and the validity of *Troodon formosus*. J Paleontol. 2025;99: 219–240. doi:10.1017/jpa.2024.67

31. Norell MA, Makovicky PJ, Bever GS, Balanoff AM, Clark JM, Barsbold R, et al. A Review of the Mongolian Cretaceous Dinosaur *Saurornithoides* (Troodontidae: Theropoda). Am Mus Novit. 2009;2009: 1–63. doi:10.1206/648.1

32. Xu X, Tan Q, Sullivan C, Han F, Xiao D. A Short-Armed Troodontid Dinosaur from the Upper Cretaceous of Inner Mongolia and Its Implications for Troodontid Evolution. PLOS ONE. 2011;6: e22916. doi:10.1371/journal.pone.0022916

33. Tsuihiji T, Barsbold R, Watabe M, Tsogtbaatar K, Chinzorig T, Fujiyama Y, et al. An exquisitely preserved troodontid theropod with new information on the palatal structure from the Upper Cretaceous of Mongolia. Naturwissenschaften. 2014;101: 131–142. doi:10.1007/s00114-014-1143-9

34. Pei R, Norell MA, Barta DE, Bever GS, Pittman M, Xu X. Osteology of a New Late Cretaceous Troodontid Specimen from Ukhaa Tolgod, Ömnögovi Aimag, Mongolia. Am Mus Novit. 2017;2017: 1–47. doi:10.1206/3889.1

35. Pei R, Qin Y, Wen A, Zhao Q, Wang Z, Liu Z, et al. A new troodontid from the Upper Cretaceous Gobi Basin of inner Mongolia, China. Cretac Res. 2022;130: 105052. doi:10.1016/j.cretres.2021.105052

36. Lee S, Lee Y-N, Park J-Y, Kim S-H, Badamkhatan Z, Idersaikhan D, et al. The first troodontid (Dinosauria: Theropoda) from the Upper Cretaceous Baruungoyot Formation of Mongolia. J Vertebr Paleontol. 2023;43: e2364746. doi:10.1080/02724634.2024.2364746

37. Lü J, Xu L, Liu Y, Zhang X, Jia S, Ji Q. A New Troodontid Theropod from the Late Cretaceous of Central China, and the Radiation of Asian Troodontids. Acta Palaeontol Pol. 2010;55: 381–388. doi:10.4202/app.2009.0047

38. Makovicky PJ, Norell MA, Clark JM, Rowe T. Osteology and Relationships of *Byronosaurus jaffei* (Theropoda: Troodontidae). Am Mus Novit. 2003;2003: 1–32. doi:10.1206/0003-0082(2003)402<0001:OAROBJ>2.0.CO;2

39. Xu X, Norell MA, Wang X, Makovicky PJ, Wu X. A basal troodontid from the Early Cretaceous of China. Nature. 2002;415: 780–784. doi:10.1038/415780a

40. Barsbold R, Osmólska H, Kurzanov SM. On a new troodontid (Dinosauria, Theropoda) from the Early Cretaceous of Mongolia. Acta Palaeontol Pol. 1987;32: 121–132.

41. Tsuihiji T, Barsbold R, Watabe M, Tsogtbaatar K, Suzuki S, Hattori S. New material of a troodontid theropod (Dinosauria: Saurischia) from the Lower Cretaceous of Mongolia. Hist Biol. 2016;28: 128–138. doi:10.1080/08912963.2015.1005086

42. Shen C, Lü J, Liu S, Kundrát M, Brusatte SL, Gao H. A New Troodontid Dinosaur from the Lower Cretaceous Yixian Formation of Liaoning Province, China. Acta Geol Sin - Engl Ed. 2017;91: 763–780. doi:10.1111/1755-6724.13307

43. Shen C, Zhao B, Gao CL, Lü J, Kundrát M. A new troodontid dinosaur (*Liaoningvenator curriei* gen. et sp. nov.) from the Early Cretaceous Yixian Formation in western Liaoning province. Acta Geosci Sin. 2017;38: 359–371.

44. Russell DA, Dong Z-M. A nearly complete skeleton of a new troodontid dinosaur from the Early Cretaceous of the Ordos Basin, Inner Mongolia, People's Republic of China. Can J Earth Sci. 1993;30: 2163–2173. doi:10.1139/e93-187

45. Xu X, Wang X. A New Troodontid (Theropoda: Troodontidae) from the Lower Cretaceous Yixian Formation of Western Liaoning, China. Acta Geol Sin - Engl Ed. 2004;78: 22–26. doi:10.1111/j.1755-6724.2004.tb00671.x

46. Kubota K, Kobayashi Y, Ikeda T. Early Cretaceous troodontine troodontid (Dinosauria: Theropoda) from the Ohyamashimo Formation of Japan reveals the early evolution of Troodontinae. Sci Rep. 2024;14: 16392. doi:10.1038/s41598-024-66815-2

47. Hartman S, Mortimer M, Wahl WR, Lomax DR, Lippincott J, Lovelace DM. A new paravian dinosaur from the Late Jurassic of North America supports a late acquisition of avian flight. PeerJ. 2019;7: e7247. doi:10.7717/peerj.7247

48. Chure DJ. *Koparion douglassi*, a new dinosaur from the Morrison Formation (Upper Jurassic) of Dinosaur National Monument; the oldest troodontid (Theropoda: Maniraptora). Brigh Young Univ Geol Stud. 1994;40: 11–15.

49. Pittman M, O’Connor J, Tse E, Makovicky P, Field DJ, Ma W, et al. The fossil record of Mesozoic and Paleocene pennaraptorans. 2020. Available: https://www.repository.cam.ac.uk/items/e5839a51-9053-496e-b792-a97cf06e71b5

50. Kirkland JI, Zanno LE, Sampson SD, Clark JM, DeBlieux DD. A primitive therizinosauroid dinosaur from the Early Cretaceous of Utah. Nature. 2005;435: 84–87. doi:10.1038/nature03468

51. Suarez CA, Suarez MB, Terry DO Jr, Grandstaff DE. RARE EARTH ELEMENT GEOCHEMISTRY AND TAPHONOMY OF THE EARLY CRETACEOUS CRYSTAL GEYSER DINOSAUR QUARRY, EAST-CENTRAL UTAH. PALAIOS. 2007;22: 500–512. doi:10.2110/palo.2005.p05-126r

52. Suarez MB, Suarez CA, Kirkland JI, González LA, Grandstaff DE, Terry DO Jr. SEDIMENTOLOGY, STRATIGRAPHY, AND DEPOSITIONAL ENVIRONMENT OF THE CRYSTAL GEYSER DINOSAUR QUARRY, EAST-CENTRAL UTAH. PALAIOS. 2007;22: 513–527. doi:10.2110/palo.2006.p06-014r

53. Zanno LE. Osteology of *Falcarius utahensis* (Dinosauria: Theropoda): characterizing the anatomy of basal therizinosaurs. Zool J Linn Soc. 2010;158: 196–230. doi:10.1111/j.1096-3642.2009.00464.x

54. Perle A. A new segnosaurid from the Upper Cretaceous of Mongolia (in Russian). Jt Sov Paleontol Exped Trans. 1981;8: 45–55.

55. Clark JM, Altangerel P, Norell M. The skull of *Erlicosaurus andrewsi*, a late Cretaceous "Segnosaur" (Theropoda, Therizinosauridae) from Mongolia. American Museum novitates; no. 3115. 1994. Available: https://digitallibrary.amnh.org/bitstreams/d6131974-7b77-4733-b779-ce4f6d12f418/download

56. Yao X, Liao C-C, Sullivan C, Xu X. A new transitional therizinosaurian theropod from the Early Cretaceous Jehol Biota of China. Sci Rep. 2019;9: 5026. doi:10.1038/s41598-019-41560-z

57. Liao C-C, Xu X. Cranial osteology of *Beipiaosaurus inexpectus* (Theropoda: Therizinosauria). Vertebr Palasiat. 2019;57: 117.

58. Pu H, Kobayashi Y, Lü J, Xu L, Wu Y, Chang H, et al. An Unusual Basal Therizinosaur Dinosaur with an Ornithischian Dental Arrangement from Northeastern China. PLOS ONE. 2013;8: e63423. doi:10.1371/journal.pone.0063423

59. Lautenschlager S, Witmer LM, Altangerel P, Zanno LE, Rayfield EJ. Cranial anatomy of *Erlikosaurus andrewsi* (Dinosauria, Therizinosauria): new insights based on digital reconstruction. J Vertebr Paleontol. 2014;34: 1263–1291. doi:10.1080/02724634.2014.874529

60. Freimuth WJ, Zanno LE. New craniodental materials of *Falcarius utahensis* (Theropoda: Therizinosauria) reveal patterns of intraspecific variation and cranial evolution in early coelurosaurians. Anat Rec. 2025. doi:10.1002/ar.70080

61. Averianov A, Sues H-D. Troodontidae (Dinosauria: Theropoda) from the Upper Cretaceous of Uzbekistan. Cretac Res. 2016;59: 98–110. doi:10.1016/j.cretres.2015.11.005

62. Senter P, Kirkland JI, DeBlieux DD. *Martharaptor greenriverensis*, a New Theropod Dinosaur from the Lower Cretaceous of Utah. PLOS ONE. 2012;7: e43911. doi:10.1371/journal.pone.0043911

63. Suarez CA, González LA, Ludvigson GA, Kirkland JI, Cifelli RL, Kohn MJ. Multi-Taxa Isotopic Investigation of Paleohydrology In the Lower Cretaceous Cedar Mountain Formation, Eastern Utah, U.S.A.: Deciphering Effects Of the Nevadaplano Plateau On Regional Climate. J Sediment Res. 2014;84: 975–987. doi:10.2110/jsr.2014.76

64. Suarez MB, Suarez CA, Al-Suwaidi AH, Hatzell G, Kirkland JI, Salazar-Verdin J, et al. Terrestrial carbon isotope chemostratigraphy in the Yellow Cat Member of the Cedar Mountain Formation: complications and Pitfalls. Terrestrial Depositional Systems. Elsevier; 2017. pp. 303–336. Available: https://www.sciencedirect.com/science/article/pii/B978012803243500008X

65. Kirkland J, Suarez M, Suarez C, Hunt-Foster R. The Lower Cretaceous in east-central Utah—the Cedar Mountain Formation and its bounding strata. Geol Intermt West. 2016;3: 101–228.

66. Joeckel RM, Ludvigson GA, Möller A, Hotton CL, Suarez MB, Suarez CA, et al. Chronostratigraphy and terrestrial palaeoclimatology of Berriasian–Hauterivian strata of the Cedar Mountain Formation, Utah, USA. In: Wagreich M, Hart M, Sames B, Yilmaz IO, editors. Cretaceous Climate Events and Short-Term Sea-Level Changes. Geological Society of London; 2020. p. 0. doi:10.1144/SP498-2018-133

67. Joeckel RM, Suarez CA, McLean NM, Möller A, Ludvigson GA, Suarez MB, et al. Berriasian–Valanginian Geochronology and Carbon-Isotope Stratigraphy of the Yellow Cat Member, Cedar Mountain Formation, Eastern Utah, USA. Geosciences. 2023;13: 32. doi:10.3390/geosciences13020032

68. Mori H. Dinosaurian faunas of the Cedar Mountain Formation and LA-ICP-MS detrital zircon ages for three stratigraphic sections. Master's Thesis, Brigham Young University. 2009. Available: https://search.proquest.com/openview/c85585e3729786869dbe2f1ef592cf57/1?pq-origsite=gscholar&cbl=18750&diss=y&casa_token=mizTYKDX2Z4AAAAA:UgS2daKhmiglj5ViUVhpXZuVDYrd0TpW4IeKgzFGZa6H7kXkAzKxvQZe-Sf-rteGUJjuVJHgsf4

69. Kirkland JI, Sertich JJW, Titus AL. Dinosaur biostratigraphy of the non-marine Cretaceous of Utah. Geol Soc Lond Spec Publ. 2025;545: 65–90. doi:10.1144/SP545-2023-211

70. Rhodes MM, Henderson DM, Currie PJ. Maniraptoran pelvic musculature highlights evolutionary patterns in theropod locomotion on the line to birds. PeerJ. 2021;9: e10855. doi:10.7717/peerj.10855

71. Zanno LE, Kirkland JI, Herzog L. Second therizinosaurian mass death locality in the Lower Cretaceous Cedar Mountain Formation yields a new taxon. J Vertebr Paleontol SVP Program Abstr Book. 2014;2014: 260.

72. Nomina anatomica veterinaria. 6th ed. Hannover: International Committee on Veterinary Anatomical Nomenclature; 2017.

73. Hendrickx C, Mateus O. *Torvosaurus gurneyi* n. sp., the Largest Terrestrial Predator from Europe, and a Proposed Terminology of the Maxilla Anatomy in Nonavian Theropods. PLOS ONE. 2014;9: e88905. doi:10.1371/journal.pone.0088905

74. Witmer LM. The Evolution of the Antorbital Cavity of Archosaurs: A Study in Soft-Tissue Reconstruction in the Fossil Record with an Analysis of the Function of Pneumaticity. J Vertebr Paleontol. 1997;17: 1–76. doi:10.1080/02724634.1997.10011027

75. Zanno LE. A taxonomic and phylogenetic re-evaluation of Therizinosauria (Dinosauria: Maniraptora). J Syst Palaeontol. 2010;8: 503–543. doi:10.1080/14772019.2010.488045

76. Avrahami HM, Lindsey KP, Herzog L. Advances in 3D surface scanning: Mobile device applications set a new standard for the digitization of fossils and museum artifacts. 83rd Annual Meeting of the Society of Vertebrate Paleontology. 2023. pp. 18–21.

77. Brusatte SL, Lloyd GT, Wang SC, Norell MA. Gradual Assembly of Avian Body Plan Culminated in Rapid Rates of Evolution across the Dinosaur-Bird Transition. Curr Biol. 2014;24: 2386–2392. doi:10.1016/j.cub.2014.08.034

78. Nesbitt SJ, Denton RK, Loewen MA, Brusatte SL, Smith ND, Turner AH, et al. A mid-Cretaceous tyrannosauroid and the origin of North American end-Cretaceous dinosaur assemblages. Nat Ecol Evol. 2019;3: 892–899. doi:10.1038/s41559-019-0888-0

79. Hattori S, Kawabe S, Imai T, Shibata M, Miyata K, Xu X, et al. Osteology of *Fukuivenator paradoxus*: a bizarre maniraptoran theropod from the Early Cretaceous of Fukui, Japan. Mem Fukui Prefect Dinosaur Mus. 2021;20: 1–82.

80. Kobayashi Y, Zelenitsky DK, Fiorillo AR, Chinzorig T. Didactyl therizinosaur with a preserved keratinous claw from the Late Cretaceous of Mongolia. iScience. 2025;28. doi:10.1016/j.isci.2025.112141

81. Morales ME, Goloboff PA. New TNT routines for parallel computing with MPI. Mol Phylogenet Evol. 2023;178: 107643. doi:10.1016/j.ympev.2022.107643

82. Ronquist F, Teslenko M, van der Mark P, Ayres DL, Darling A, Höhna S, et al. MrBayes 3.2: Efficient Bayesian Phylogenetic Inference and Model Choice Across a Large Model Space. Syst Biol. 2012;61: 539–542. doi:10.1093/sysbio/sys029

83. Harrison LB, Larsson HCE. Among-Character Rate Variation Distributions in Phylogenetic Analysis of Discrete Morphological Characters. Syst Biol. 2015;64: 307–324. doi:10.1093/sysbio/syu098

84. Heath TA, Huelsenbeck JP, Stadler T. The fossilized birth–death process for coherent calibration of divergence-time estimates. Proc Natl Acad Sci. 2014;111: E2957–E2966. doi:10.1073/pnas.1319091111

85. Ding A, Pittman M, Upchurch P, O'Connor J, Field DJ, Xu X. The Biogeography of Coelurosaurian Theropods and Its Impact on Their Evolutionary History. 2020. doi:10.17863/CAM.59246

86. Powers MJ, Sullivan C, Currie PJ. Re-examining ratio based premaxillary and maxillary characters in Eudromaeosauria (Dinosauria: Theropoda): Divergent trends in snout morphology between Asian and North American taxa. Palaeogeogr Palaeoclimatol Palaeoecol. 2020;547: 109704. doi:10.1016/j.palaeo.2020.109704

87. Jackson DA. Stopping Rules in Principal Components Analysis: A Comparison of Heuristical and Statistical Approaches. Ecology. 1993;74: 2204–2214. doi:10.2307/1939574

88. Avrahami HM, Makovicky PJ, Tucker RT, Zanno LE. A new semi-fossorial thescelosaurine dinosaur from the Cenomanian-age Mussentuchit Member of the Cedar Mountain Formation, Utah. Anat Rec. 2024;307: 3717–3781. doi:10.1002/ar.25505

89. Xu X, Zheng X, You H. A new feather type in a nonavian theropod and the early evolution of feathers. Proc Natl Acad Sci. 2009;106: 832–834. doi:10.1073/pnas.0810055106

90. Choiniere JN, Clark JM, Norell MA, Xu X. Cranial Osteology of *Haplocheirus sollers* Choiniere et al., 2010 (Theropoda: Alvarezsauroidea). Am Mus Novit. 2014;2014: 1–44. doi:10.1206/3816.1

91. Choiniere JN, Clark JM, Forster CA, Norell MA, Eberth DA, Erickson GM, et al. A juvenile specimen of a new coelurosaur (Dinosauria: Theropoda) from the Middle–Late Jurassic Shishugou Formation of Xinjiang, People’s Republic of China. J Syst Palaeontol. 2014;12: 177–215. doi:10.1080/14772019.2013.781067

92. Balanoff AM, Xu X, Kobayashi Y, Matsufune Y, Norell MA. Cranial Osteology of the Theropod Dinosaur *Incisivosaurus gauthieri* (Theropoda: Oviraptorosauria). Am Mus Novit. 2009;2009: 1–35. doi:10.1206/644.1

93. Brusatte SL, Carr TD, Norell MA. The Osteology of *Alioramus,* A Gracile and Long-Snouted Tyrannosaurid (Dinosauria: Theropoda) from the Late Cretaceous of Mongolia. Bull Am Mus Nat Hist. 2012;2012: 1–197. doi:10.1206/770.1

94. Voris JT, Zelenitsky DK, Therrien F, Ridgely RC, Currie PJ, Witmer LM. Two exceptionally preserved juvenile specimens of *Gorgosaurus libratus* (Tyrannosauridae, Albertosaurinae) provide new insight into the timing of ontogenetic changes in

tyrannosaurids. J Vertebr Paleontol. 2021;41: e2041651. doi:10.1080/02724634.2021.2041651

95. Gold MEL, Brusatte SL, Norell MA. The Cranial Pneumatic Sinuses of the Tyrannosaurid *Alioramus* (Dinosauria: Theropoda) and the Evolution of Cranial Pneumaticity in Theropod Dinosaurs. Am Mus Novit. 2013;2013: 1–46. doi:10.1206/3790.1

96. Napoli JG. Resolving the “Ontogeny Problem” in Vertebrate Paleontology. bioRxiv; 2024. p. 2024.10.25.620216. doi:10.1101/2024.10.25.620216

97. Powers MJ, Fabbri M, Doschak MR, Bhullar B-AS, Evans DC, Norell MA, et al. A new hypothesis of eudromaeosaurian evolution: CT scans assist in testing and constructing morphological characters. J Vertebr Paleontol. 2021;41: e2010087. doi:10.1080/02724634.2021.2010087

98. Yu C, Watanabe A, Qin Z, Logan King J, Witmer LM, Ma Q, et al. Avialan-like brain morphology in *Sinovenator* (Troodontidae, Theropoda). Commun Biol. 2024;7: 168. doi:10.1038/s42003-024-05832-3

99. Averianov AO, Krasnolutskii SA, Ivantsov SV. A new basal coelurosaur (Dinosauria: Theropoda) from the Middle Jurassic of Siberia. Proc Zool Inst RAS. 2010;314: 42–57.

100. Witmer LM, Ridgely RC. The Paranasal Air Sinuses of Predatory and Armored Dinosaurs (Archosauria: Theropoda and Ankylosauria) and Their Contribution to Cephalic Structure. Anat Rec. 2008;291: 1362–1388. doi:10.1002/ar.20794

101. Currie PJ, Zhao X-J. A new carnosaur (Dinosauria, Theropoda) from the Jurassic of Xinjiang, People’s Republic of China. Can J Earth Sci. 1993;30: 2037–2081. doi:10.1139/e93-179

102. Barker CT, Naish D, Newham E, Katsamenis OL, Dyke G. Complex neuroanatomy in the rostrum of the Isle of Wight theropod *Neovenator salerii*. Sci Rep. 2017;7: 3749. doi:10.1038/s41598-017-03671-3

103. Tahara R, Larsson HCE. Cranial pneumatic anatomy of *Ornithomimus edmontonicus* (Ornithomimidae: Theropoda). J Vertebr Paleontol. 2011;31: 127–143. doi:10.1080/02724634.2011.539646

104. Xu X, Norell MA, Kuang X, Wang X, Zhao Q, Jia C. Basal tyrannosauroids from China and evidence for protofeathers in tyrannosauroids. Nature. 2004;431: 680–684. doi:10.1038/nature02855

105. Xu X, Clark JM, Forster CA, Norell MA, Erickson GM, Eberth DA, et al. A basal tyrannosauroid dinosaur from the Late Jurassic of China. Nature. 2006;439: 715–718. doi:10.1038/nature04511

106. Kobayashi Y, Barsbold R. Reexamination of a primitive ornithomimosaur, *Garudimimus brevipes* Barsbold, 1981 (Dinosauria: Theropoda), from the Late Cretaceous of Mongolia. Can J Earth Sci. 2005;42: 1501–1521. doi:10.1139/e05-044

107. Balanoff AM, Norell MA. Osteology of *Khaan mckennai* (Oviraptorosauria: Theropoda). Bull Am Mus Nat Hist. 2012;2012: 1–77.

108. Choiniere JN, Forster CA, de Klerk WJ. New information on *Nqwebasaurus thwazi*, a coelurosaurian theropod from the Early Cretaceous Kirkwood Formation in South Africa. J Afr Earth Sci. 2012;71–72: 1–17. doi:10.1016/j.jafrearsci.2012.05.005

109. Norell MA, Clark JM, Turner AH, Makovicky PJ, Barsbold R, Rowe T. A New Dromaeosaurid Theropod from Ukhaa Tolgod (Ömnögov, Mongolia). Am Mus Novit. 2006;2006: 1–51. doi:10.1206/0003-0082(2006)3545[1:ANDTFU]2.0.CO;2

110. Carpenter K, Miles C, Cloward K. New small theropod from the upper Jurassic Morrison formation of Wyoming. In: Carpenter K, editor. The Carnivorous Dinosaurs. Indiana University Press Bloomington; 2005. pp. 23–48.

111. Ji Q, Norell MA, Makovicky PJ, Gao K-Q, Ji S, Yuan C. An Early Ostrich Dinosaur and Implications for Ornithomimosaur Phylogeny. Am Mus Novit. 2003;2003: 1–19. doi:10.1206/0003-0082(2003)420<0001:AEODAI>2.0.CO;2

112. Chiappe LM, Norell MA, Clark JM. The skull of a relative of the stem-group bird *Mononykus*. Nature. 1998;392: 275–278. doi:10.1038/32642

113. Yin Y-L, Pei R, Zhou C-F. Cranial morphology of *Sinovenator changii* (Theropoda: Troodontidae) on the new material from the Yixian Formation of western Liaoning, China. PeerJ. 2018;6: e4977. doi:10.7717/peerj.4977

114. Choiniere JN, Clark JM, Forster CA, Xu X. A basal coelurosaur (Dinosauria: Theropoda) from the Late Jurassic (Oxfordian) of the Shishugou Formation in Wucaiwan, People's Republic of China. J Vertebr Paleontol. 2010;30: 1773–1796. doi:10.1080/02724634.2010.520779

115. Napoli JG, Ruebenstahl AA, Bhullar B-AS, Turner AH, Norell MA. A New Dromaeosaurid (Dinosauria: Coelurosauria) from Khulsan, Central Mongolia. Am Mus Novit. 2021;2021: 1–47. doi:10.1206/3982.1

116. Holliday CM, Porter WR, Vliet KA, Witmer LM. The frontoparietal fossa and dorsotemporal fenestra of archosaurs and their significance for interpretations of vascular and muscular anatomy in dinosaurs. Anat Rec. 2020;303: 1060–1074. doi:10.1002/ar.24218

117. Xu X, Choiniere J, Tan Q, Benson RBJ, Clark J, Sullivan C, et al. Two Early Cretaceous Fossils Document Transitional Stages in Alvarezsaurian Dinosaur Evolution. Curr Biol. 2018;28: 2853-2860.e3. doi:10.1016/j.cub.2018.07.057

118. Sampson SD, Witmer LM. CRANIOFACIAL ANATOMY OF *MAJUNGASAURUS CRENATISSIMUS* (THEROPODA: ABELISAURIDAE) FROM THE LATE CRETACEOUS OF MADAGASCAR. J Vertebr Paleontol. 2007;27: 32–104. doi:10.1671/0272-4634(2007)27[32:CAOMCT]2.0.CO;2

119. Hendrickx C, Araújo R, Mateus O. The non-avian theropod quadrate I: standardized terminology with an overview of the anatomy and function. PeerJ. 2015;3: e1245. doi:10.7717/peerj.1245

120. Rauhut OWM, Milner AC, Moore-Fay S. Cranial osteology and phylogenetic position of the theropod dinosaur *Proceratosaurus bradleyi* (Woodward, 1910) from the Middle Jurassic of England. Zool J Linn Soc. 2010;158: 155–195. doi:10.1111/j.1096-3642.2009.00591.x

121. Naish D, Cau A. The osteology and affinities of Eotyrannus lengi, a tyrannosauroid theropod from the Wealden Supergroup of southern England. PeerJ. 2022;10: e12727. doi:10.7717/peerj.12727

122. Kobayashi Y, Lu JC. A new ornithomimid dinosaur with gregarious habits from the Late Cretaceous of China. Acta Palaeontol Pol. 2003;48. Available: https://bibliotekanauki.pl/articles/20983.pdf

123. Paulina Carabajal A, Currie PJ, Dudgeon TW, Larsson HCE, Miyashita T. Two braincases of *Daspletosaurus* (Theropoda: Tyrannosauridae): anatomy and comparison. Can J Earth Sci. 2021;58: 885–910. doi:10.1139/cjes-2020-0185

124. Smith DK, Zanno LE, Sanders RK, Deblieux DD, Kirkland JI. New information on the braincase of the North American therizinosaurian (Theropoda, Maniraptora) Falcarius utahensis. J Vertebr Paleontol. 2011;31: 387–404. doi:10.1080/02724634.2011.549442

125. Pérez-Moreno BP, Luis Sanz J, Buscalioni AD, Moratalla JJ, Ortega F, Rasskin-Gutman D. A unique multitoothed ornithomimosaur dinosaur from the Lower Cretaceous of Spain. Nature. 1994;370: 363–367. doi:10.1038/370363a0

126. Zanno LE, Tsogtbaatar K, Chinzorig T, Gates TA. Specializations of the mandibular anatomy and dentition of *Segnosaurus galbinensis* (Theropoda: Therizinosauria). PeerJ. 2016;4: e1885. doi:10.7717/peerj.1885

127. Averianov AO, Sues H-D. A new troodontid (Dinosauria: Theropoda) from the Cenomanian of Uzbekistan, with a review of troodontid records from the territories of the former Soviet Union. J Vertebr Paleontol. 2007;27: 87–98. doi:10.1671/0272-4634(2007)27[87:ANTDTF]2.0.CO;2

128. Kobayashi Y, Barsbold R. Anatomy of *Harpymimus okladnikovi* Barsbold and Perle 1984 (Dinosauria; Theropoda) of Mongolia. In: Carpenter K, editor. The Carnivorous Dinosaurs. Bloomington, IN: Indiana University Press; 2005. pp. 97–126. Available: https://books.google.com/books?hl=en&lr=&id=Mr16gm7VRCAC&oi=fnd&pg=PA97&dq=+Kobayashi+Y+,+Barsbold+R+.+2005a+.+Anatomy+of+Harpymimus+okladnikovi+Barsbold+and+Perle+1984+(Dinosauria:+Theropoda)+of+Mongolia+.+In:+Carpenter+K+,+ed.+The+carnivorous+dinosaurs.+Bloomington+:+Indiana+University+Press+,+97+%E2%80%93126+.&ots=xB2DtWSjje&sig=7dyF01BiQjkRwTlBAU89QaTKQ1o

129. Hendrickx C, Mateus O, Araújo R. A proposed terminology of theropod teeth (Dinosauria, Saurischia). J Vertebr Paleontol. 2015;35: e982797. doi:10.1080/02724634.2015.982797

130. Hendrickx C, Mateus O, da Lourinhã M, Araújo R, da Lourinhã M, Choiniere J. The distribution of dental features in non-avian theropod dinosaurs: Taxonomic potential, degree of homoplasy, and major evolutionary trends. 2019. Available: http://palaeo-electronica.org/content/2019/2806-dental-features-in-theropods

131. Xu X, Zhao X, Clark JM. A new therizinosaur from the Lower Jurassic lower Lufeng Formation of Yunnan, China. J Vertebr Paleontol. 2001;21: 477–483. doi:10.1671/0272-4634(2001)021[0477:ANTFTL]2.0.CO;2

132. Barrett PM. The Affinities of the Enigmatic Dinosaur *Eshanosaurus deguchiianus* from the Early Jurassic of Yunnan Province, People's Republic of China. Palaeontology. 2009;52: 681–688. doi:10.1111/j.1475-4983.2009.00887.x

133. Carrano MT, Loewen MA, Sertich JJ. New materials of *Masiakasaurus knopfleri* Sampson, Carrano, and Forster, 2001, and implications for the morphology of the Noasauridae. Smithsonian Institution Scholarly Press; 2011. Available: https://dinodata.de/bibliothek/pdf_n/2011/newmaterialsmas95carr_dd.pdf

134. Kubo K, Kobayashi Y, Chinzorig T, Tsogtbaatar K. A new alvarezsaurid dinosaur (Theropoda, Alvarezsauria) from the Upper Cretaceous Baruungoyot Formation of Mongolia provides insights for bird-like sleeping behavior in non-avian dinosaurs. PLOS ONE. 2023;18: e0293801. doi:10.1371/journal.pone.0293801

135. Bapst DW, Wright AM, Matzke NJ, Lloyd GT. Topology, divergence dates, and macroevolutionary inferences vary between different tip-dating approaches applied to fossil theropods (Dinosauria). Biol Lett. 2016;12: 20160237. doi:10.1098/rsbl.2016.0237

136. Lloyd GT, Bapst DW, Friedman M, Davis KE. Probabilistic divergence time estimation without branch lengths: dating the origins of dinosaurs, avian flight and crown birds. Biol Lett. 2016;12: 20160609. doi:10.1098/rsbl.2016.0609

137. Ji Q, Ji S, Lü J, You H, Chen W, Liu Y, et al. First avialian birdfrom China (*Jinfengopteryx elegans* gen. et sp. nov.). Geol Bull China. 2005;24: 197–210.

138. Jorgensen RA, Kirkland JI, DeBlieux DD, Zanno LE, Loewen MA. Reassessment of *Yurgovuchia* Pelvic Material and Theropod Diversity in the Lower Yellow Cat Member of the Cedar Mountain Formation of Central Utah. 2023. Available: https://par.nsf.gov/biblio/10652166-reassessment-yurgovuchia-pelvic-material-theropod-diversity-lower-yellow-cat-member-cedar-mountain-formation-central-utah

139. Alonso-Zarza AM, Tanner LH. Carbonates in Continental Settings: Facies, Environments, and Processes. Elsevier; 2010.

140. Pu H, Kobayashi Y, Lü J, Xu L, Wu Y, Chang H, et al. An Unusual Basal Therizinosaur Dinosaur with an Ornithischian Dental Arrangement from Northeastern China. PLOS ONE. 2013;8: e63423. doi:10.1371/journal.pone.0063423

141. Liao C-C, Qin Z, Meade L, Xu X, Lautenschlager S. Head to herbivore: new evidence for cranial functional evolution in early-branching therizinosaurs. 85th Annual Meeting of the Society of Vertebrate Paleontology. 2025. p. 380.

142. Kirkland JI, Wolfe DG. First definitive therizinosaurid (Dinosauria; Theropoda) from North America. J Vertebr Paleontol. 2001;21: 410–414. doi:10.1671/0272-4634(2001)021[0410:FDTDTF]2.0.CO;2

143. Zanno LE, Gillette DD, Albright LB, Titus AL. A new North American therizinosaurid and the role of herbivory in 'predatory' dinosaur evolution. Proc R Soc B Biol Sci. 2009;276: 3505–3511. doi:10.1098/rspb.2009.1029

144. Kirkland JI, Britt B, Burge DL, Carpenter K, Cifelli R, DeCourten F, et al. Lower to middle Cretaceous dinosaur faunas of the central Colorado Plateau: a key to understanding 35 million years of tectonics, sedimentology, evolution, and biogeography. Brigh Young Univ Geol Stud. 1997;42: 69–104.

145. Royo-Torres R, Upchurch P, Kirkland JI, DeBlieux DD, Foster JR, Cobos A, et al. Descendants of the Jurassic turiasaurs from Iberia found refuge in the Early Cretaceous of western USA. Sci Rep. 2017;7: 14311. doi:10.1038/s41598-017-14677-2

146. Wills S, Underwood CJ, Barrett PM. Machine learning confirms new records of maniraptoran theropods in Middle Jurassic UK microvertebrate faunas. Pap Palaeontol. 2023;9: e1487. doi:10.1002/spp2.1487

147. Naish D, Martill DM. A reappraisal of Thecocoelurus daviesi (Dinosauria: Theropoda) from the Early Cretaceous of the Isle of Wight. Proc Geol Assoc. 2002;113: 23–30. doi:10.1016/S0016-7878(02)80003-7

148. Zanno LE, Makovicky PJ. On the earliest record of Cretaceous tyrannosauroids in western North America: implications for an Early Cretaceous Laurasian interchange event. Hist Biol. 2011;23: 317–325. doi:10.1080/08912963.2010.543952

149. Eaton JG, Cifelli RL, Hutchison JH, Kirkland JI, Parrish JM. Cretaceous vertebrate faunas from the Kaiparowits Plateau, south-central Utah. Vertebr Paleontol Utah. 1999;99: 345.

150. Avrahami HM, Gates TA, Heckert AB, Makovicky PJ, Zanno LE. A new microvertebrate assemblage from the Mussentuchit Member, Cedar Mountain Formation: insights into the

paleobiodiversity and paleobiogeography of early Late Cretaceous ecosystems in western North America. PeerJ. 2018;6: e5883. doi:10.7717/peerj.5883

151. Noto CR, D’Amore DC, Drumheller SK, Adams TL. A newly recognized theropod assemblage from the Lewisville Formation (Woodbine Group; Cenomanian) and its implications for understanding Late Cretaceous Appalachian terrestrial ecosystems. PeerJ. 2022;10: e12782. doi:10.7717/peerj.12782

152. McCuen N. Teeth and Taxonomy: The Dinosaur Microfauna of the Straight Cliffs Formation Reexamined. North Carolina State University. 2025. Available: https://search.proquest.com/openview/f4ee7c341c32d8a93a697bec7ef69c59/1?pq-origsite=gscholar&cbl=18750&diss=y&casa_token=T92V1tYVg64AAAAA:ORC8fFwe7uV7ndHrANMuxdKrIovvNDD7DMVHwV-R0sYNgSlt7SCsS5k1z4wqPAlgJ_qtDEYfVdQ

153. Lü J, Brusatte SL. A large, short-armed, winged dromaeosaurid (Dinosauria: Theropoda) from the Early Cretaceous of China and its implications for feather evolution. Sci Rep. 2015;5: 11775. doi:10.1038/srep11775

154. Turner AH, Pol D, Clarke JA, Erickson GM, Norell MA. A Basal Dromaeosaurid and Size Evolution Preceding Avian Flight. Science. 2007;317: 1378–1381. doi:10.1126/science.1144066

155. Wang S, Zhang Q, Tan Q, Jiangzuo Q, Zhang H, Tan L. New troodontid theropod specimen from Inner Mongolia, China clarifies phylogenetic relationships of later-diverging small-bodied troodontids and paravian body size evolution. Cladistics. 2022;38: 59–82. doi:10.1111/cla.12467

156. Holtz TR. Denticle morphometric and a possibly omnivorous feeding habit for the theropod dinosaur *Troodon*. GAIA Rev Geociências. 1998; 159.

157. Cullen TM, Cousens BL. New biogeochemical insights into Mesozoic terrestrial paleoecology and evidence for omnivory in troodontid dinosaurs. GSA Bull. 2023;136: 2689–2701. doi:10.1130/B37077.1

158. Zanno LE, Makovicky PJ. Herbivorous ecomorphology and specialization patterns in theropod dinosaur evolution. Proc Natl Acad Sci. 2011;108: 232–237. doi:10.1073/pnas.1011924108

159. Campione NE, Evans DC, Brown CM, Carrano MT. Body mass estimation in non-avian bipeds using a theoretical conversion to quadruped stylopodial proportions. Methods Ecol Evol. 2014;5: 913–923. doi:10.1111/2041-210X.12226

160. Liao C-C, Zanno LE, Wang S, Xu X. Postcranial osteology of *Beipiaosaurus inexpectus* (Theropoda: Therizinosauria). PLOS ONE. 2021;16: e0257913. doi:10.1371/journal.pone.0257913

Supporting Information

S1 File. Measurements and repository information for *Falcarius suarezarum* cranial elements. Microsoft Excel file with individual sheets that includes measurements of all cranial elements, physical and digital repository information for *Falcarius suarezarum* specimens, and links to CT slices and digital models on Morphosource project.

S2 File. Morphometric datasets. Folder containing a Microsoft Excel file with raw measurements, log-transformed, and height-corrected data for coelurosaurian maxillae

(copy and paste these data into PAST to run analyses). Also includes loadings, scree plots, and output files of all PCA iterations.

S3 File. Phylogenetic datasets. Folder containing a Microsoft Word document with supplemental tree figures; folder with annotated TWiG character list, TWiG TNT file, and TWiG nexus file for MrBayes; folder with Kobayashi et al. (2025) therizinosaurian phylogenetic character list and TNT files (one analysis with holotype maxilla only, one analysis with all *Falcarius suarezarum* crania scored).

Fig 1

Click here to access/download;Figure;Fig 1.tif

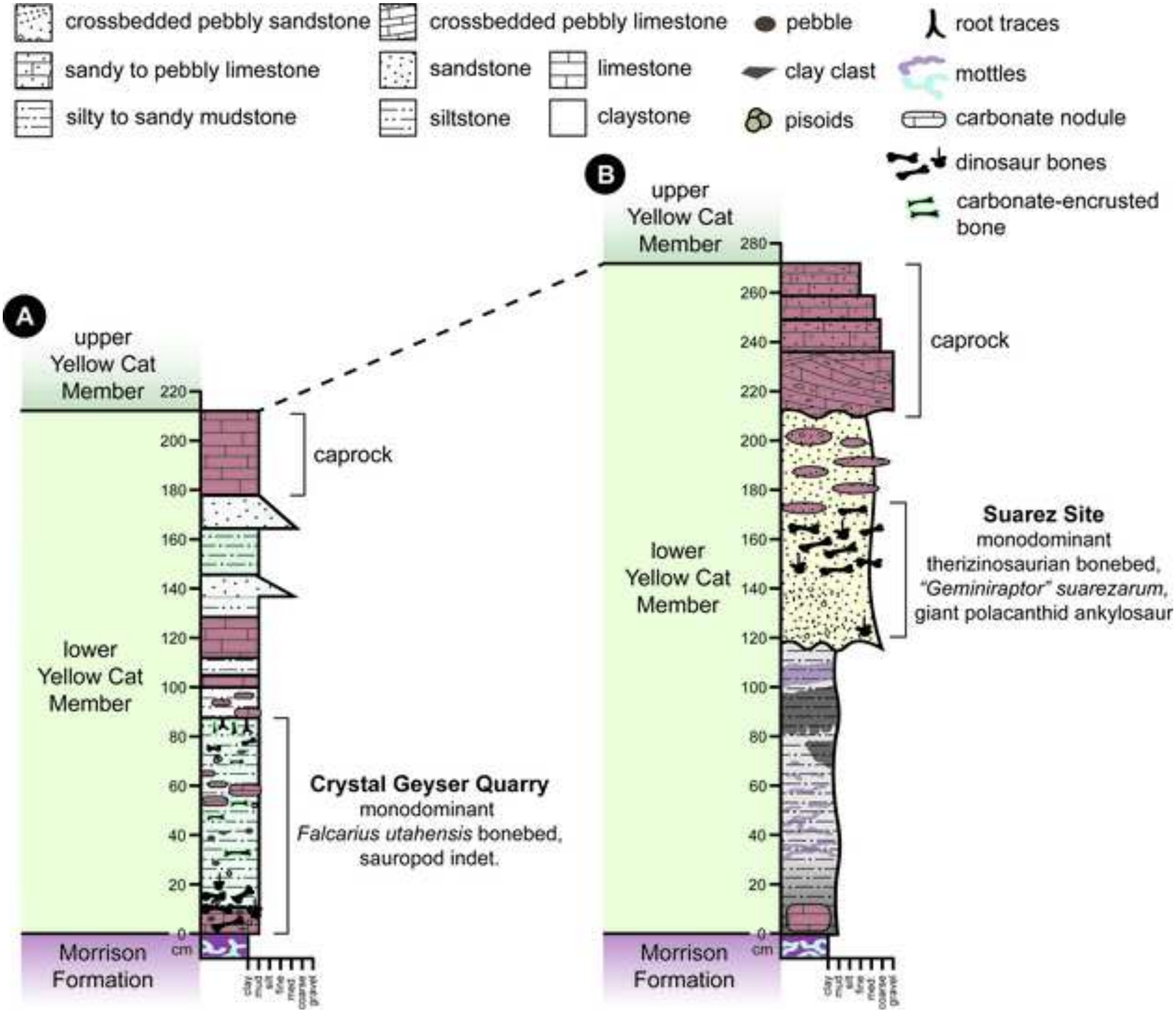

Fig 2

Click here to access/download;Figure;Fig 2.tif

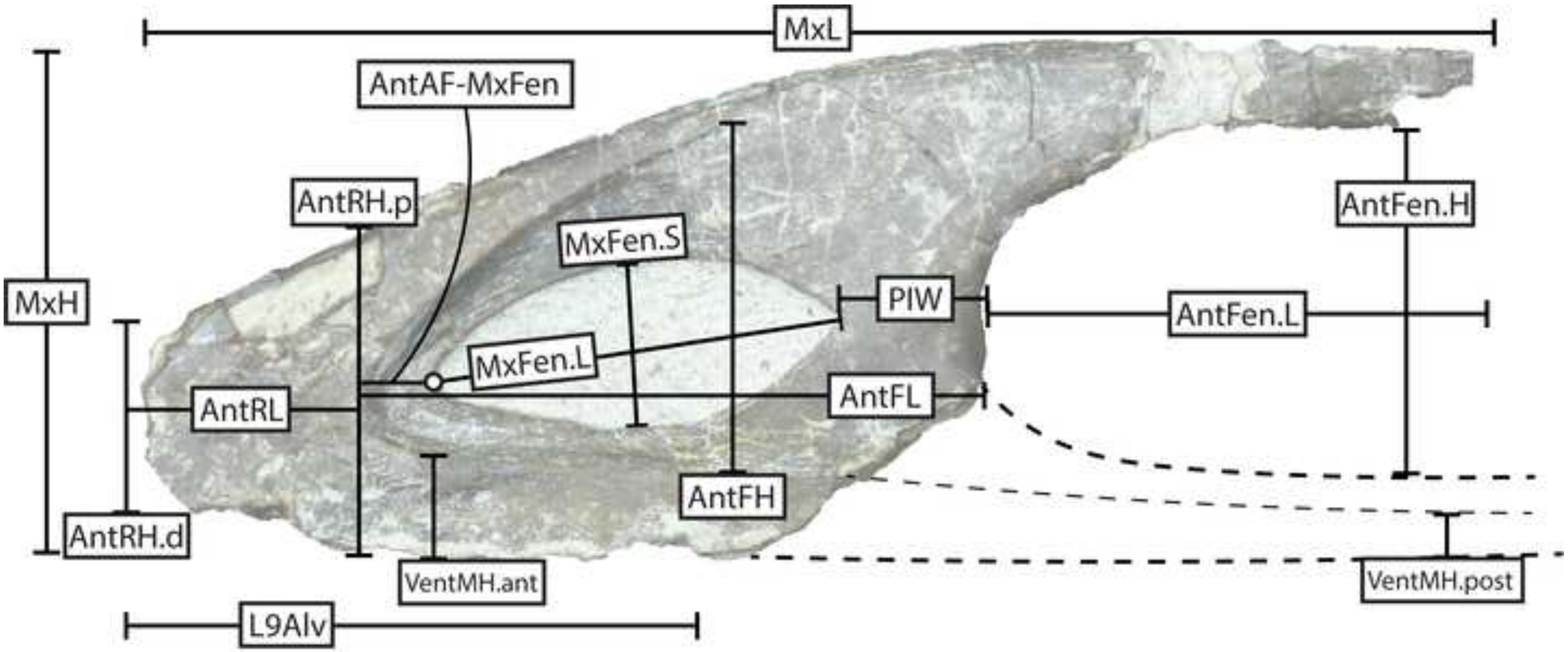

Maxilla length (MxL)
Maxilla height (MxH)
Anterior ramus length (AntRL)
Anterior ramus height distal (AntRH.d)
Anterior ramus height proximal (AntRH.p)
Antorbital fenestra height (AntFenH)
Antorbital fossa length (AntFL)
Antorbital fossa height (AntFH)

Anterior margin of antorbital fossa to maxillary fenestra (AntAF-MxFen)
Pila interfenestralis width (PIW)
Maxillary fenestra long axis (MxFen.L)
Maxillary fenestra short axis (MxFen.S)
Ventral margin heigh anterior (VentMH.ant)
Ventral margin height posterior (VentMH.post)
Length of the first 9 alveoli (L9Alv)

Click here to access/download;Figure;Fig 3.tif

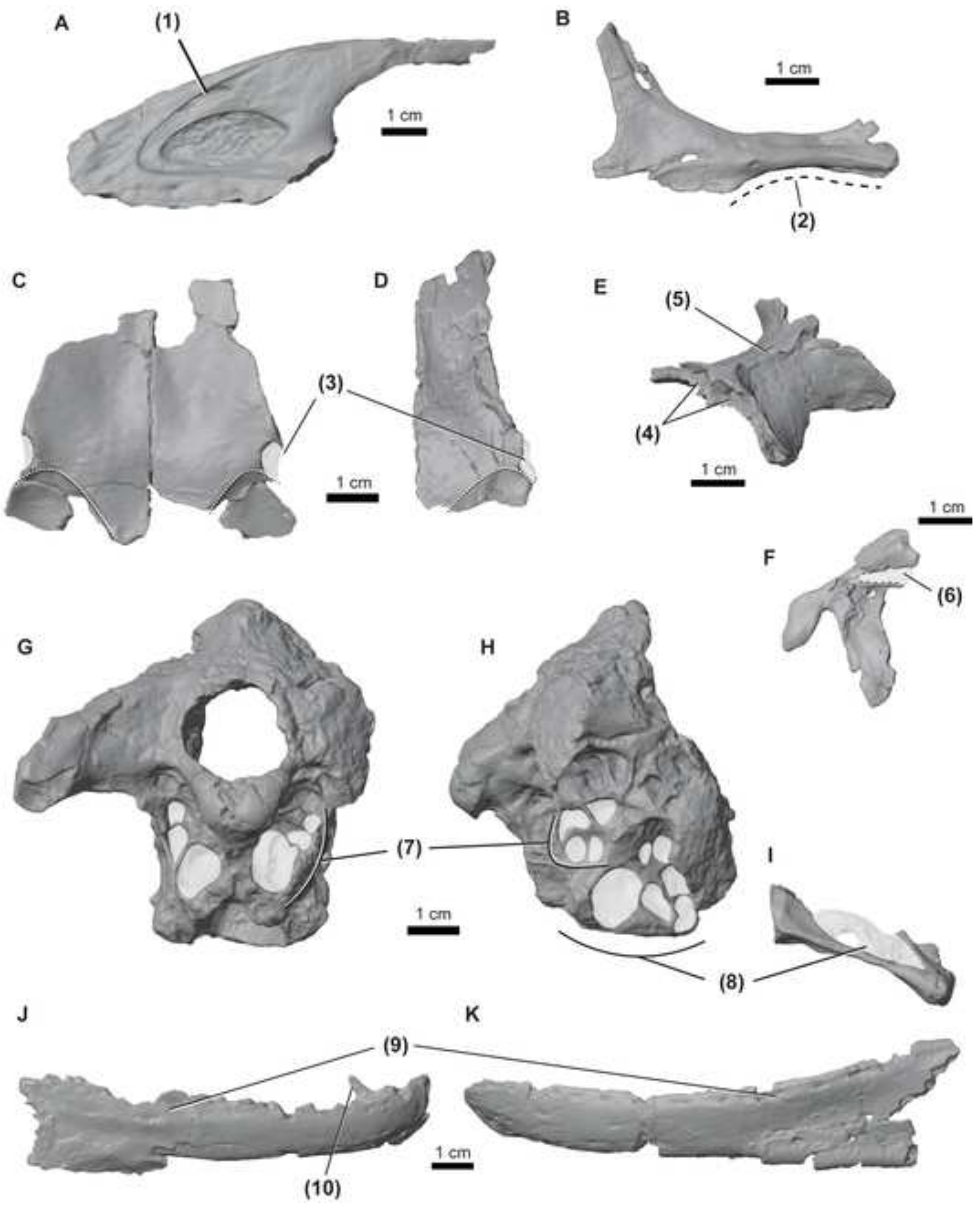

Click here to access/download;Figure;Fig 4.tif

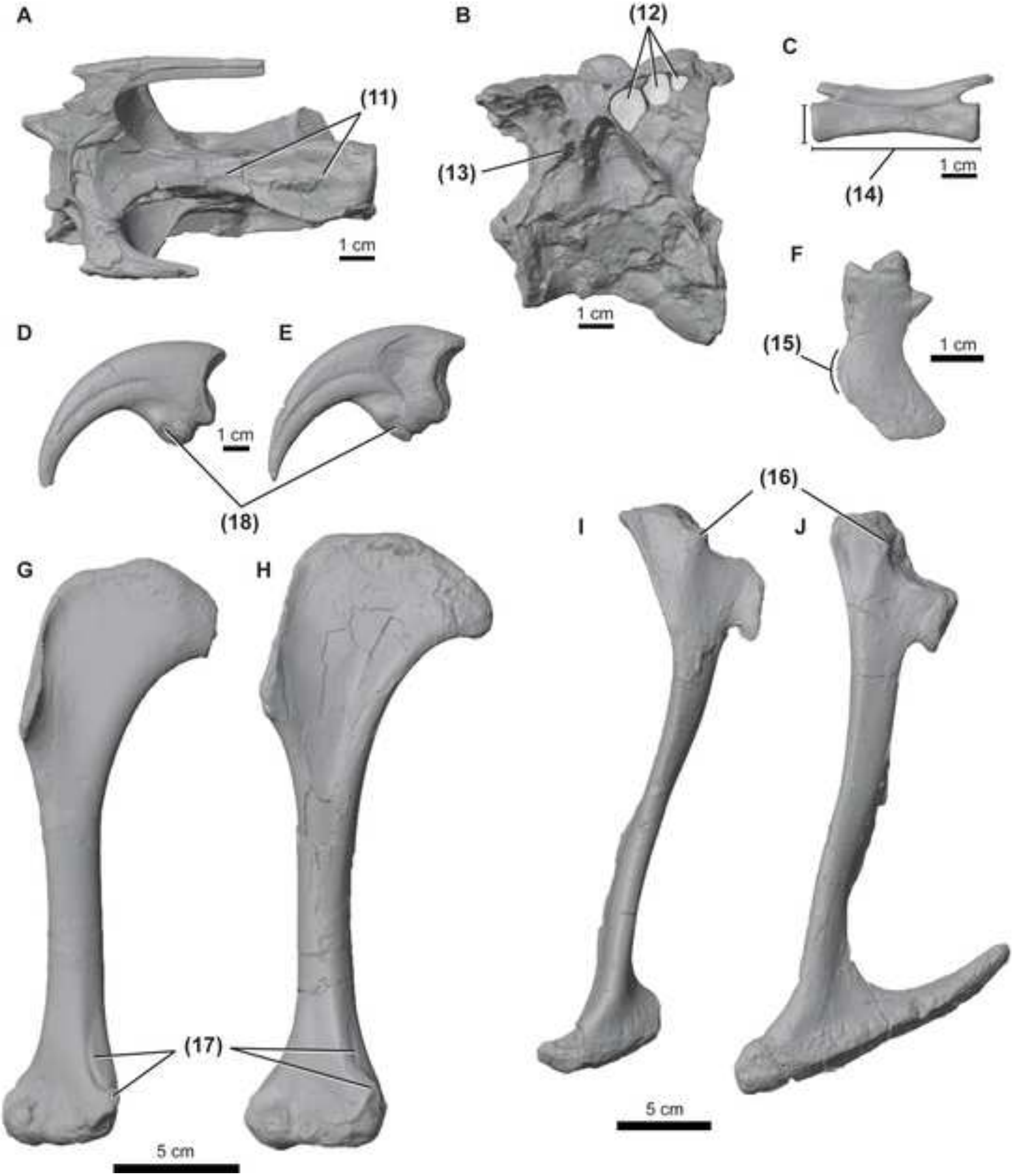

Click here to access/download;Figure;Fig 5.tif

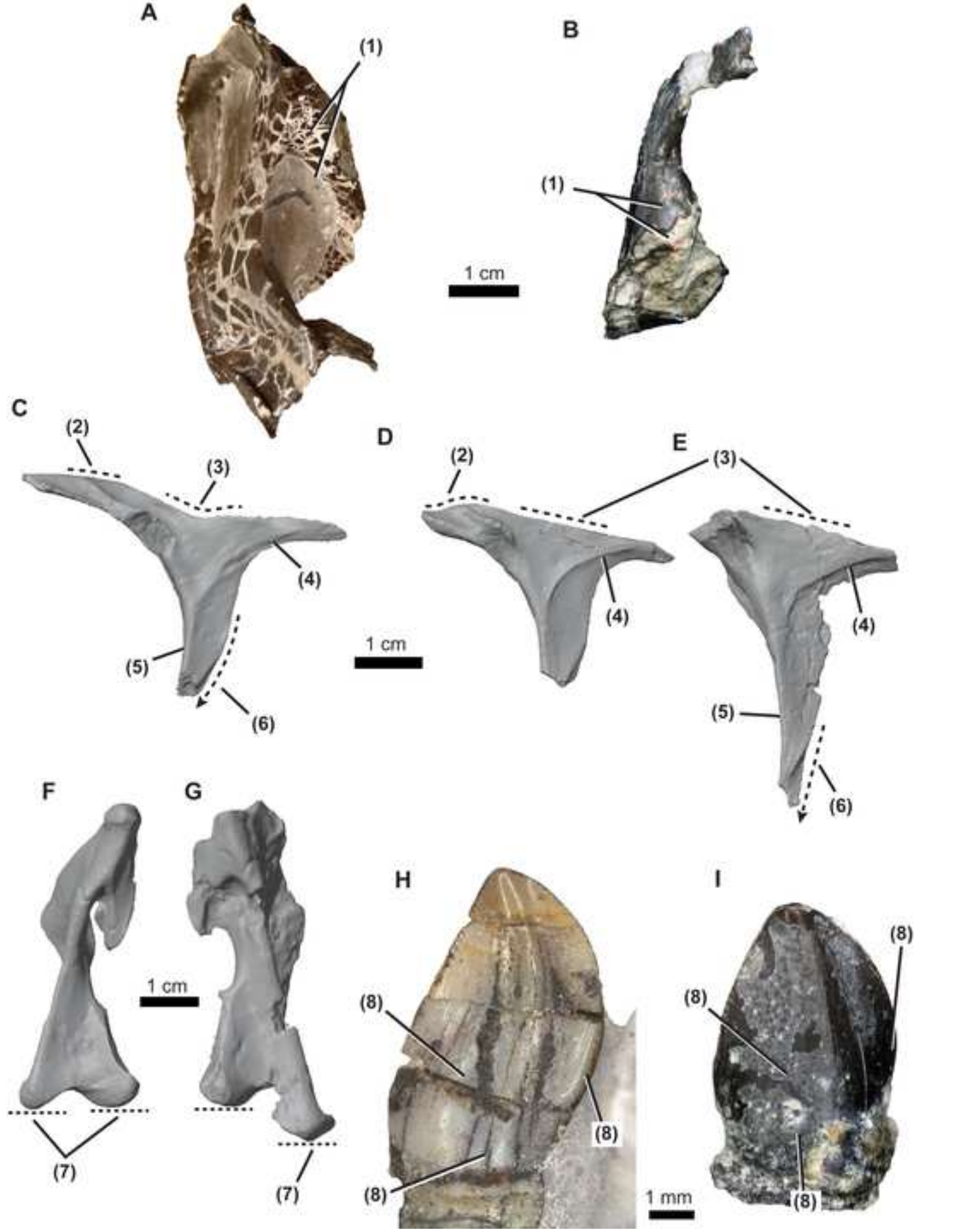

Fig 6

Click here to access/download;Figure;Fig 6.tif

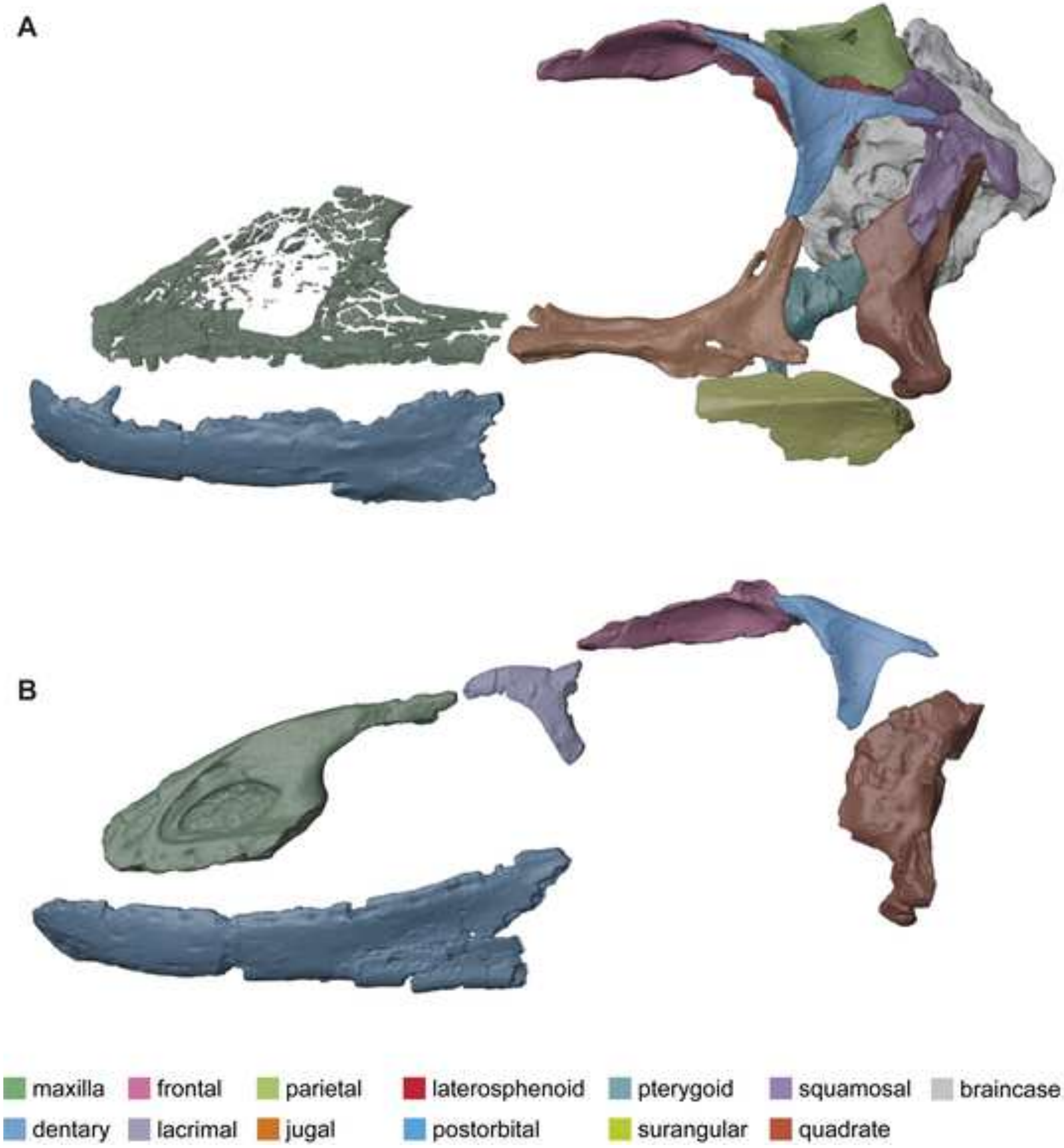

Click here to access/download;Figure;Fig 7.tif

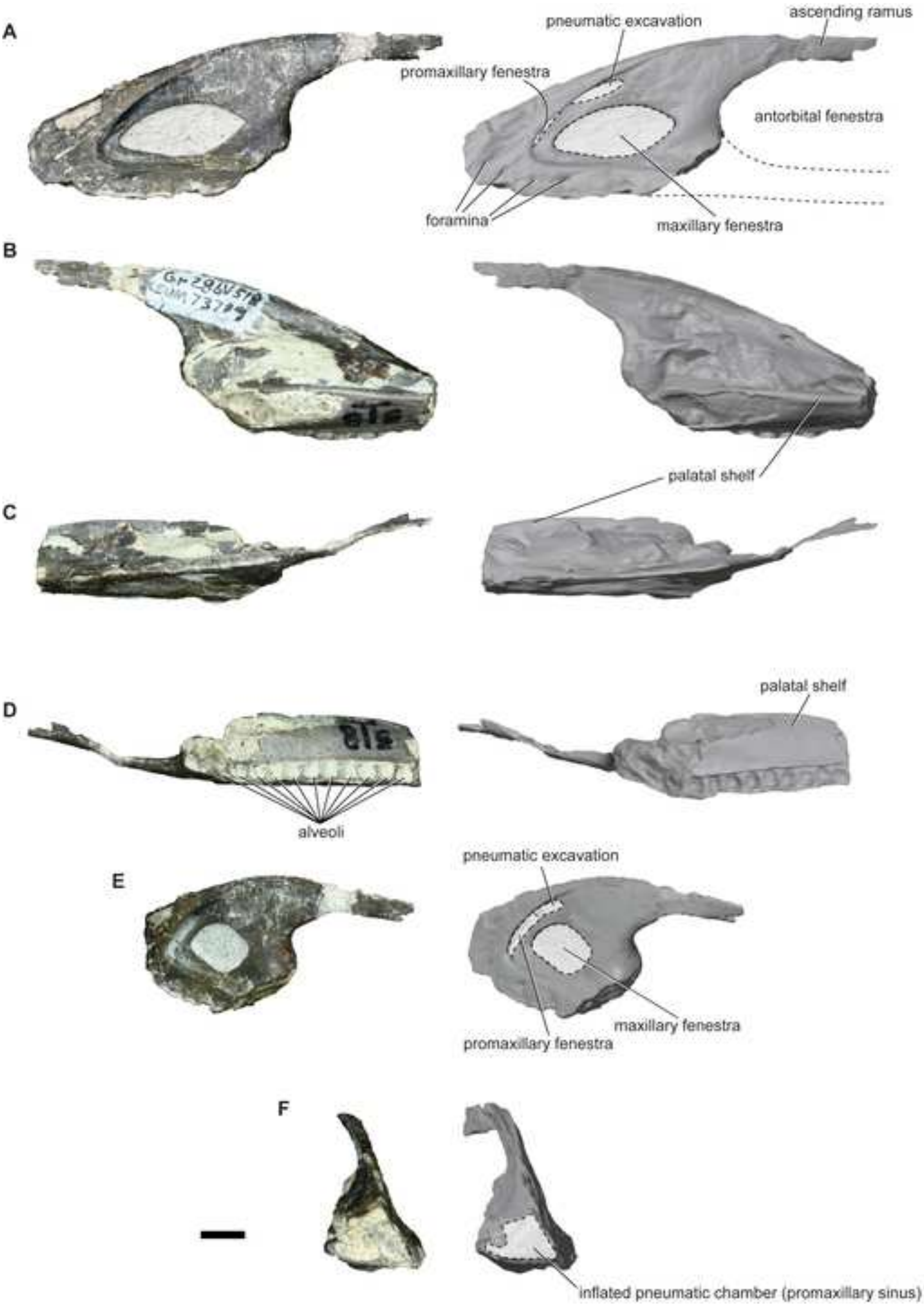

Fig 8

Click here to access/download;Figure;Fig 8.tif

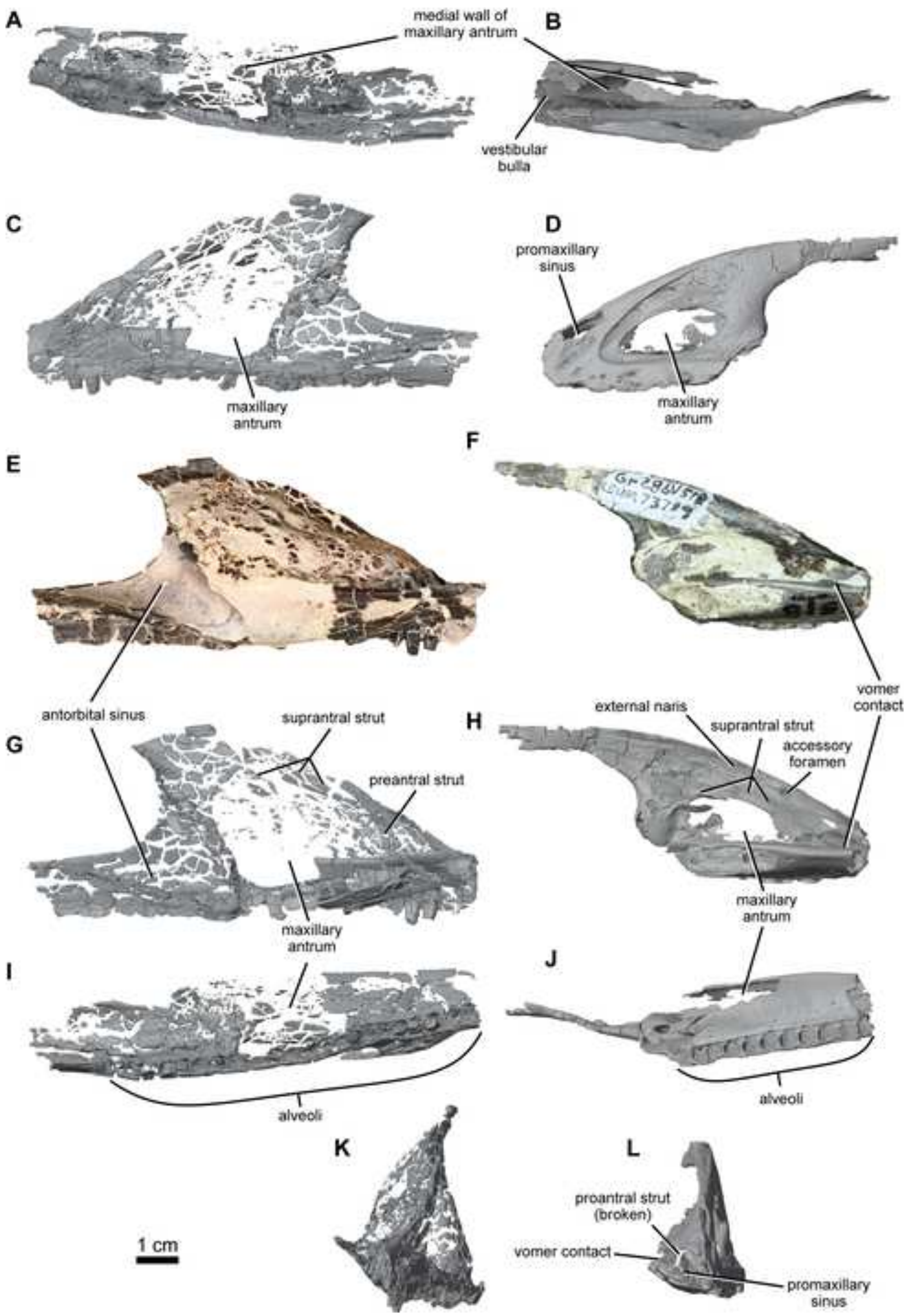

Fig 9

Click here to access/download;Figure;Fig 9.tif

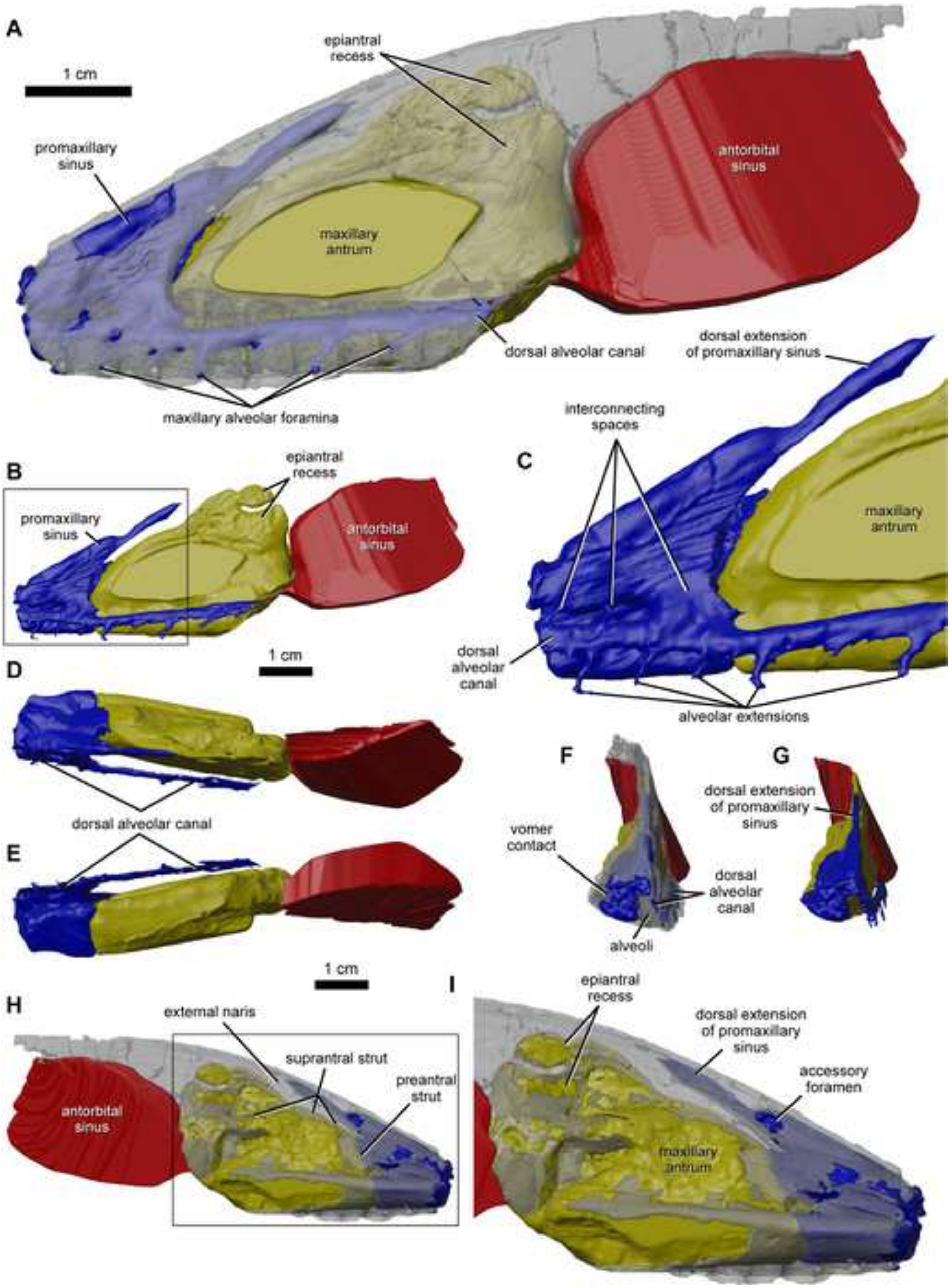

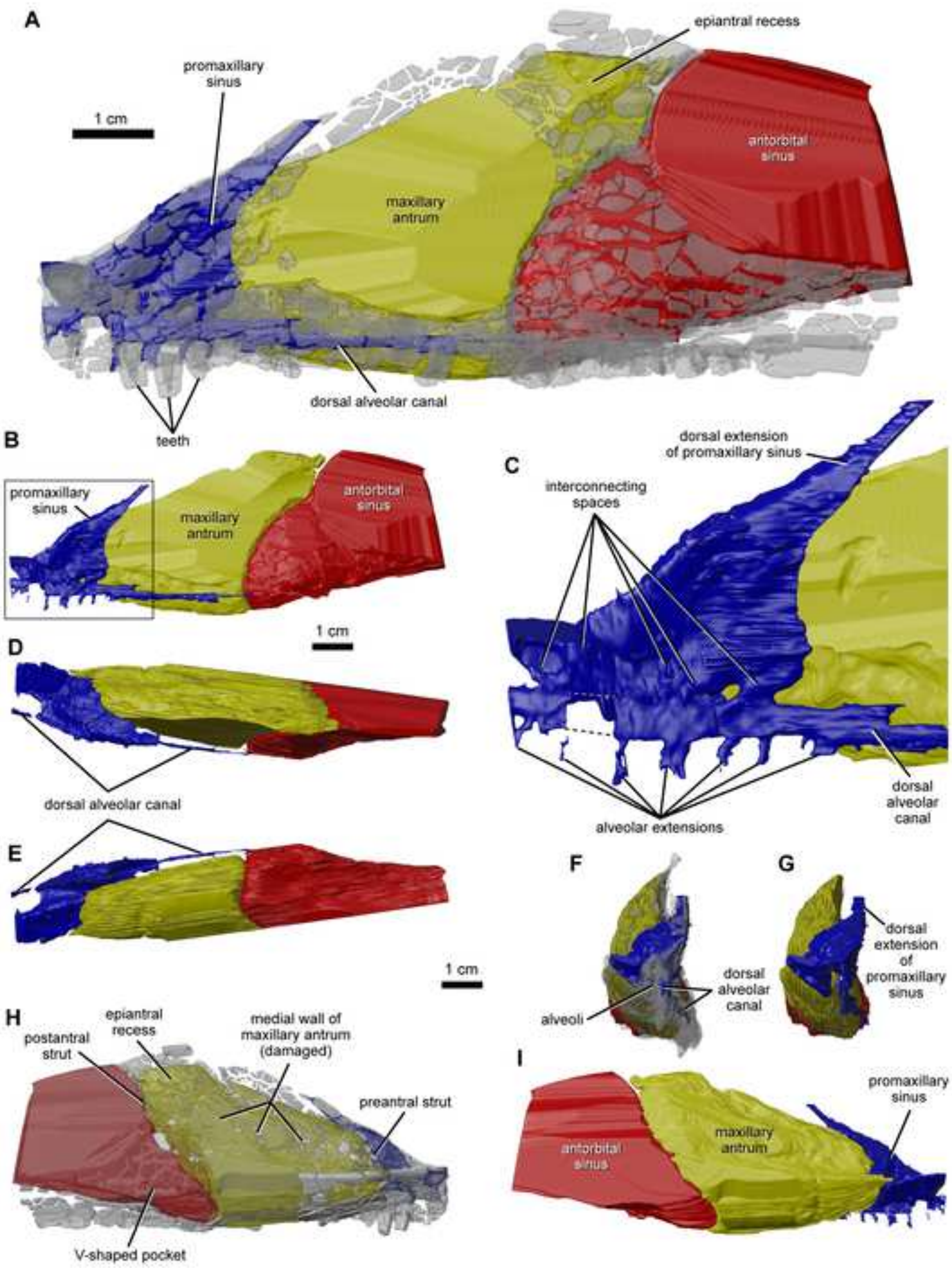
A
epiantral recess
promaxillary sinus
1 cm
antorbital sinus
maxillary antrum
dorsal alveolar canal
teeth
B
promaxillary sinus
maxillary antrum
antorbital sinus
1 cm
C
dorsal extension of promaxillary sinus
interconnecting spaces
dorsal alveolar canal
alveolar extensions
D
E
dorsal alveolar canal
F
G
dorsal extension of promaxillary sinus
dorsal alveolar canal
alveoli
1 cm
H
epiantral recess
postantral strut
medial wall of maxillary antrum (damaged)
preantral strut
V-shaped pocket
I
promaxillary sinus
maxillary antrum
antorbital sinus

Fig 11

Click here to access/download;Figure;Fig 11.tif

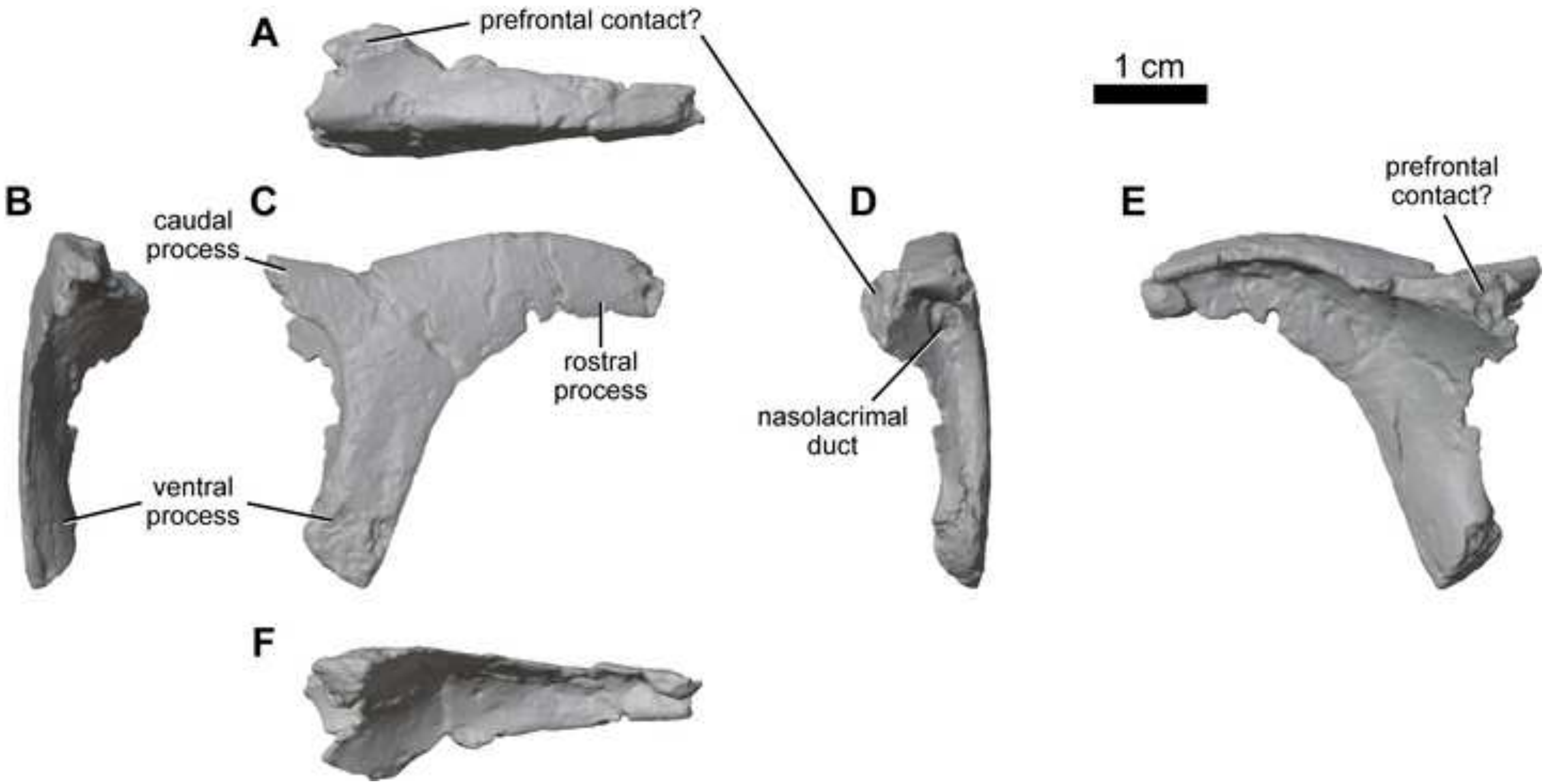

Click here to access/download;Figure;Fig 12.tif

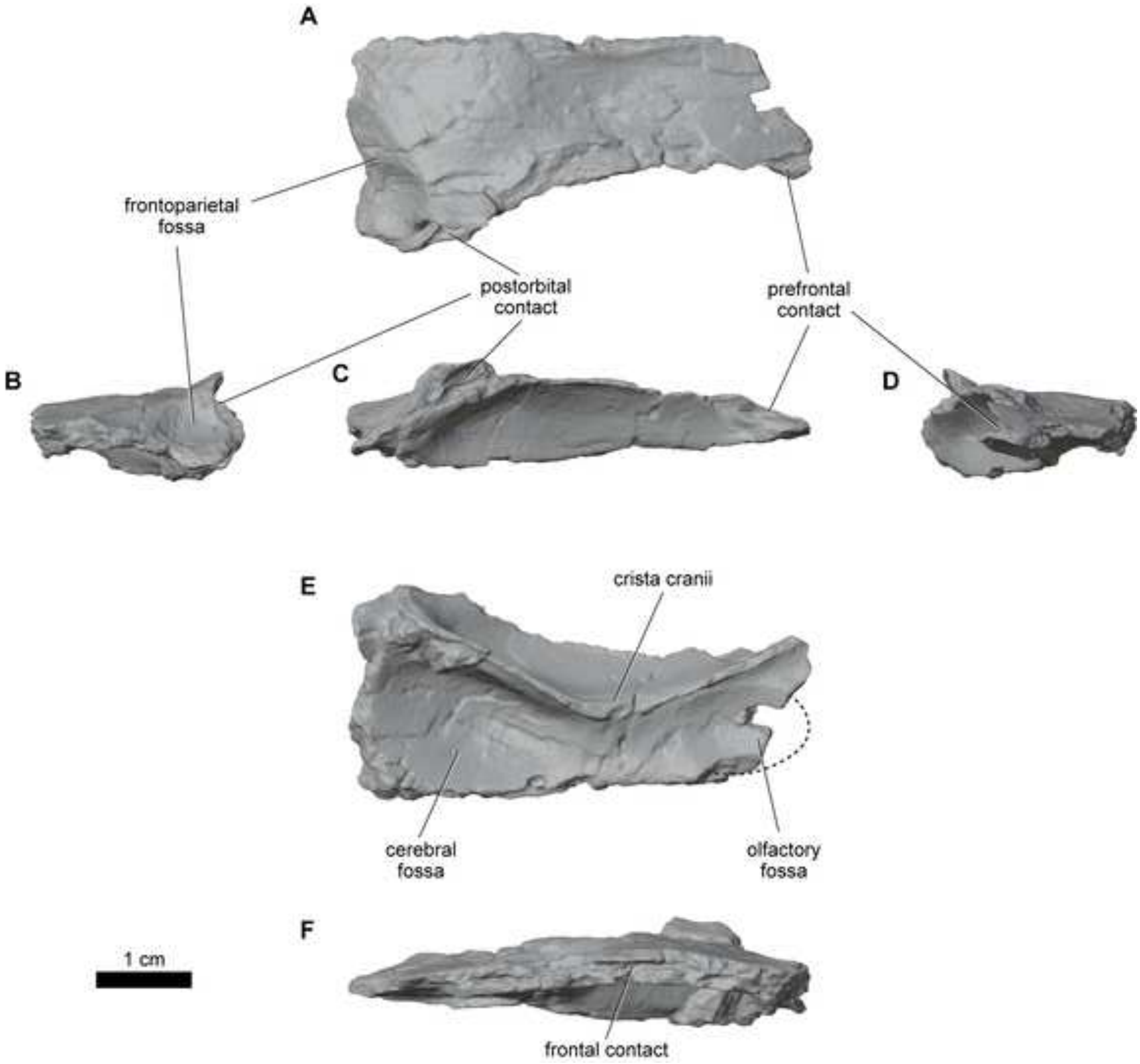

Click here to access/download;Figure;Fig 13.tif

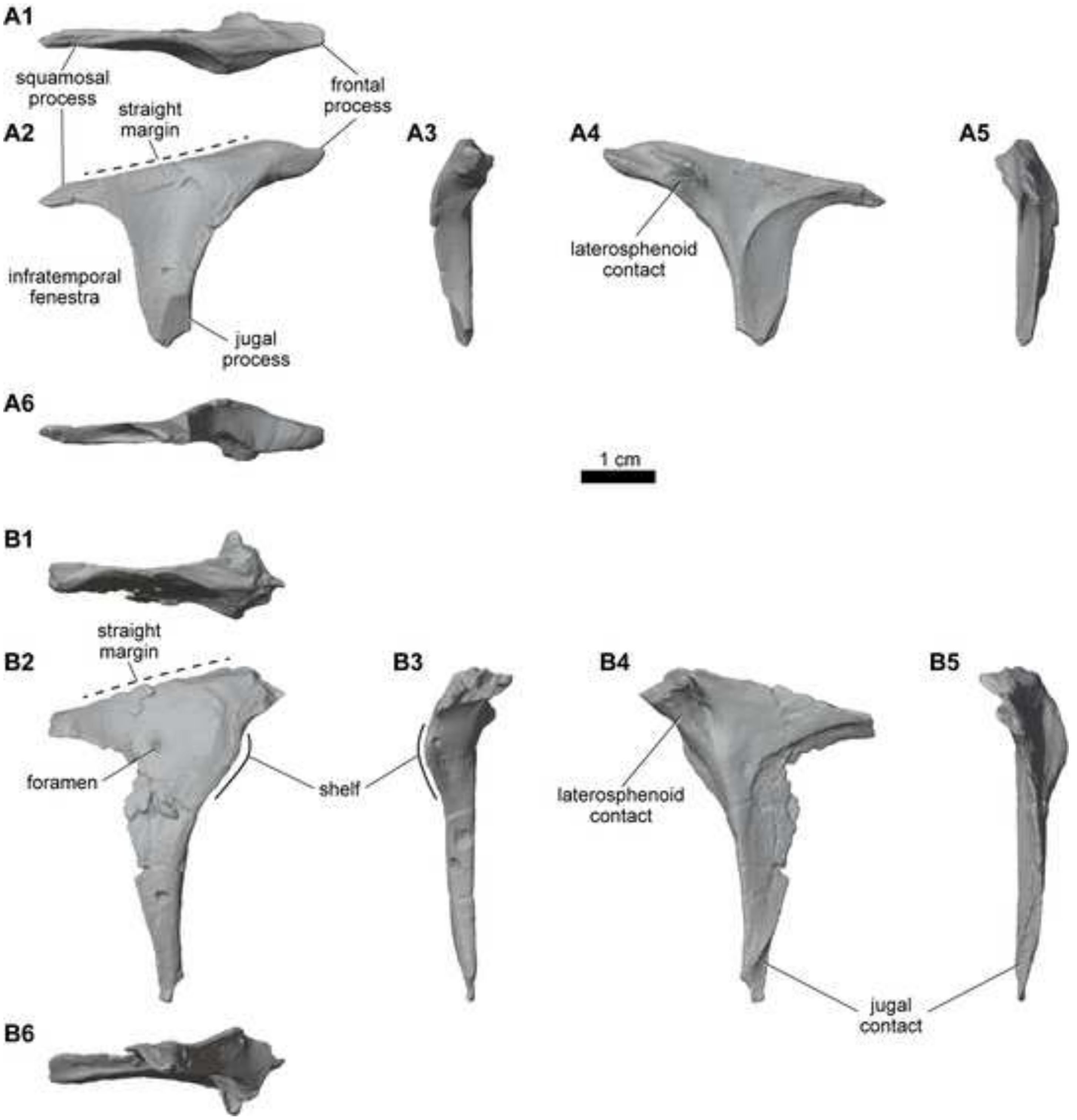

Click here to access/download;Figure;Fig 14.tif

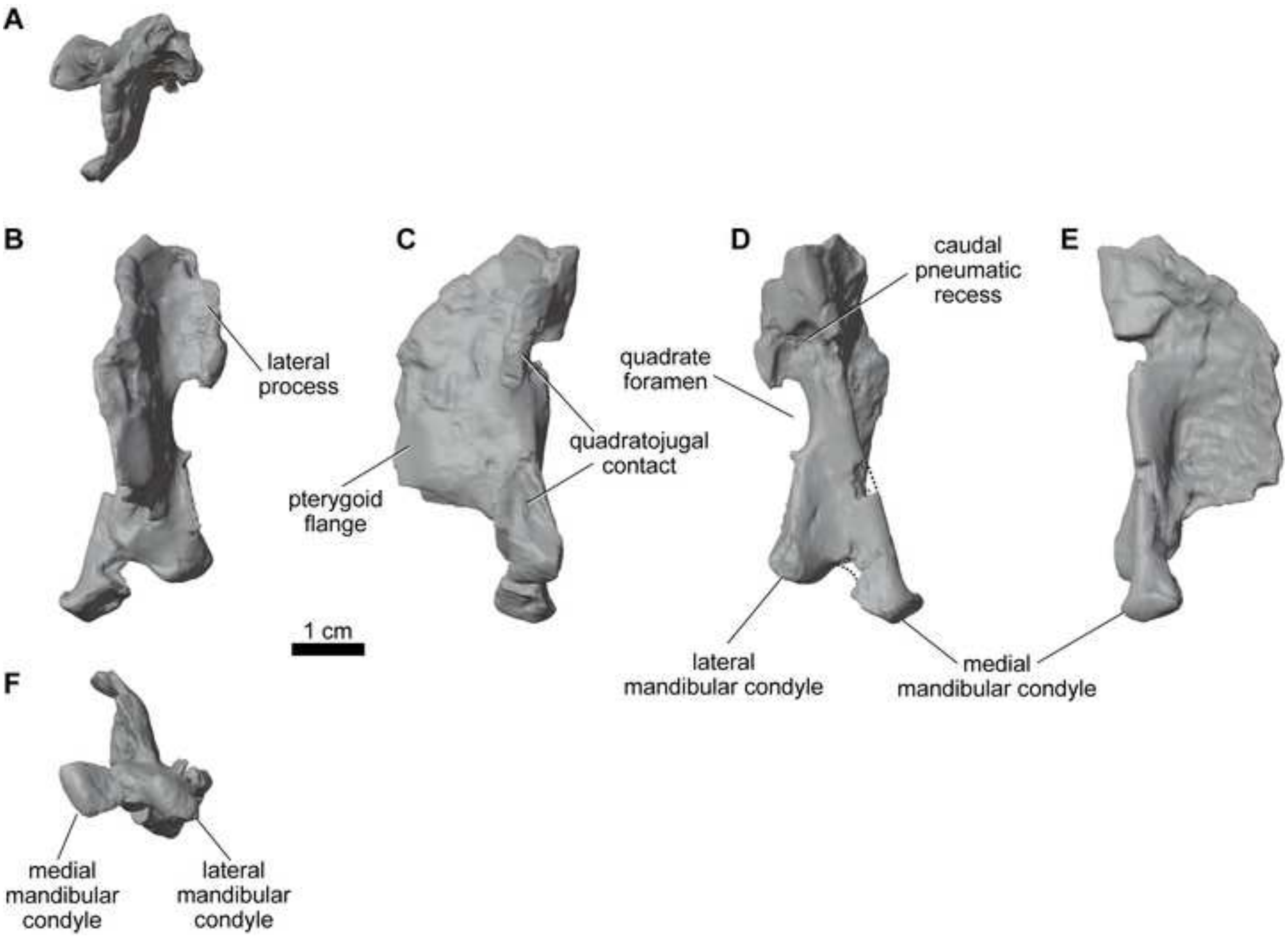

Fig 15

Click here to access/download;Figure;Fig 15.tif

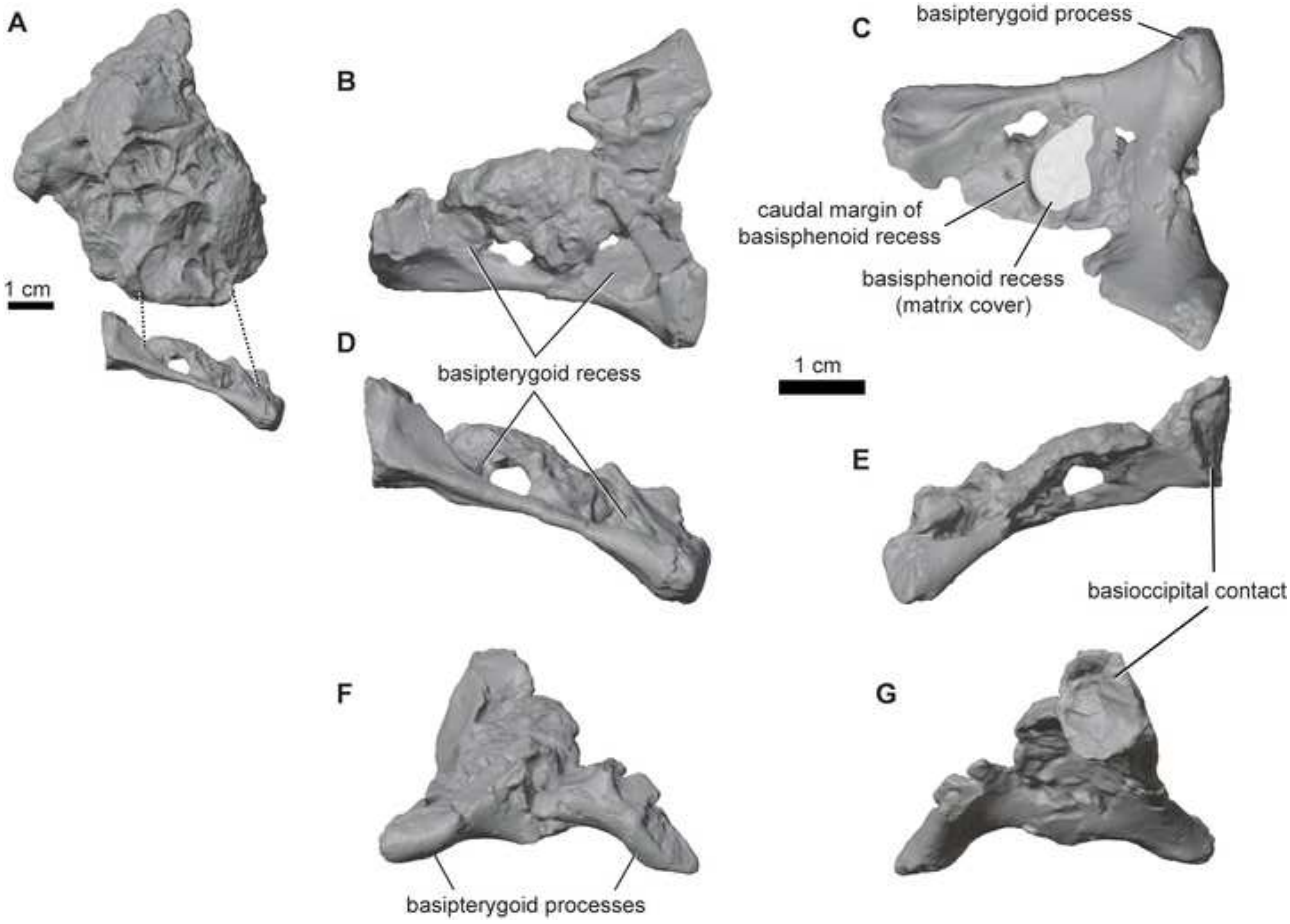

Fig 16

Click here to access/download;Figure;Fig 16.tif

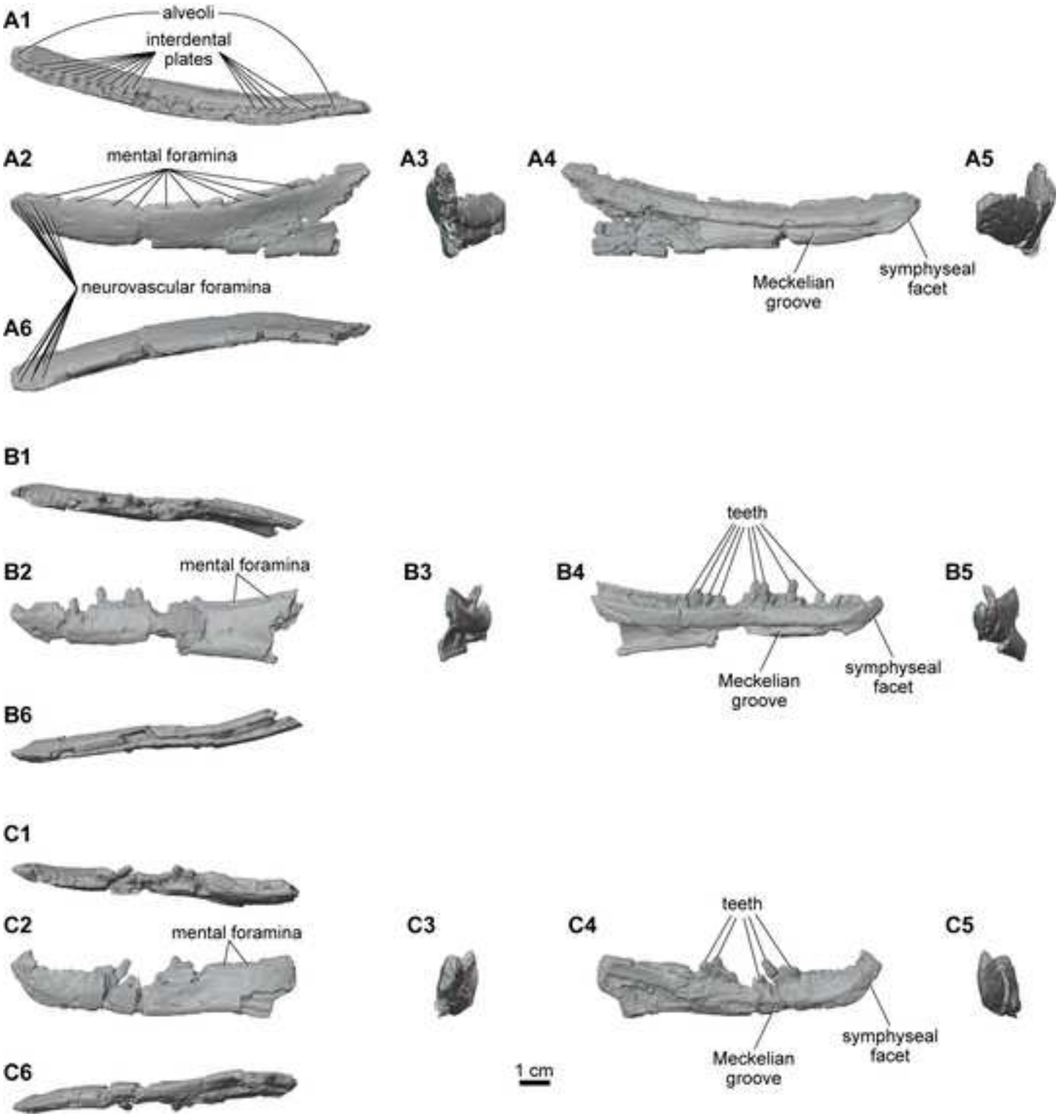

Fig 17

Click here to access/download;Figure;Fig 17.tif

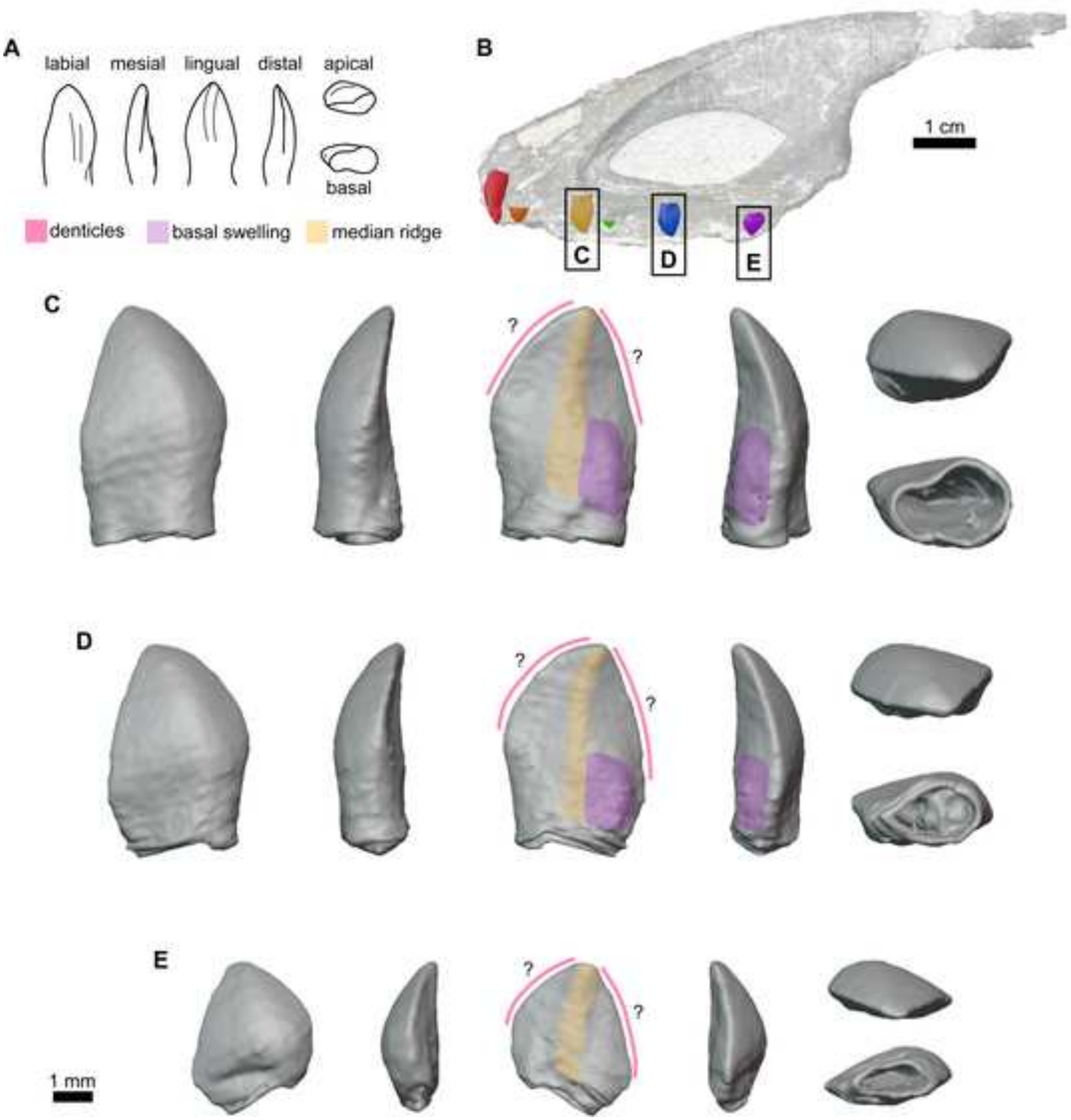

Fig 18

Click here to access/download;Figure;Fig 18.tif

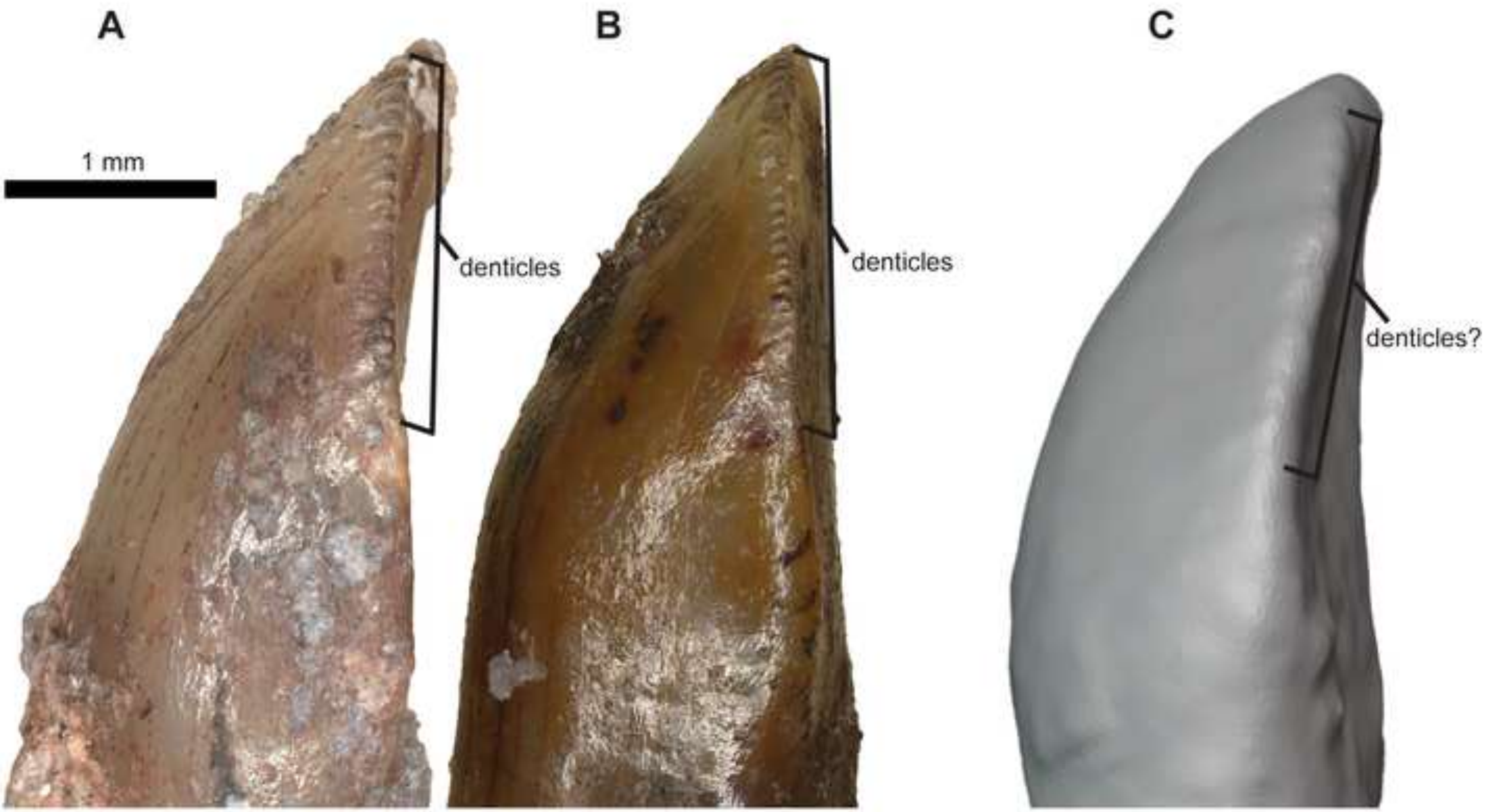

Fig 19

Click here to access/download;Figure;Fig 19.tif

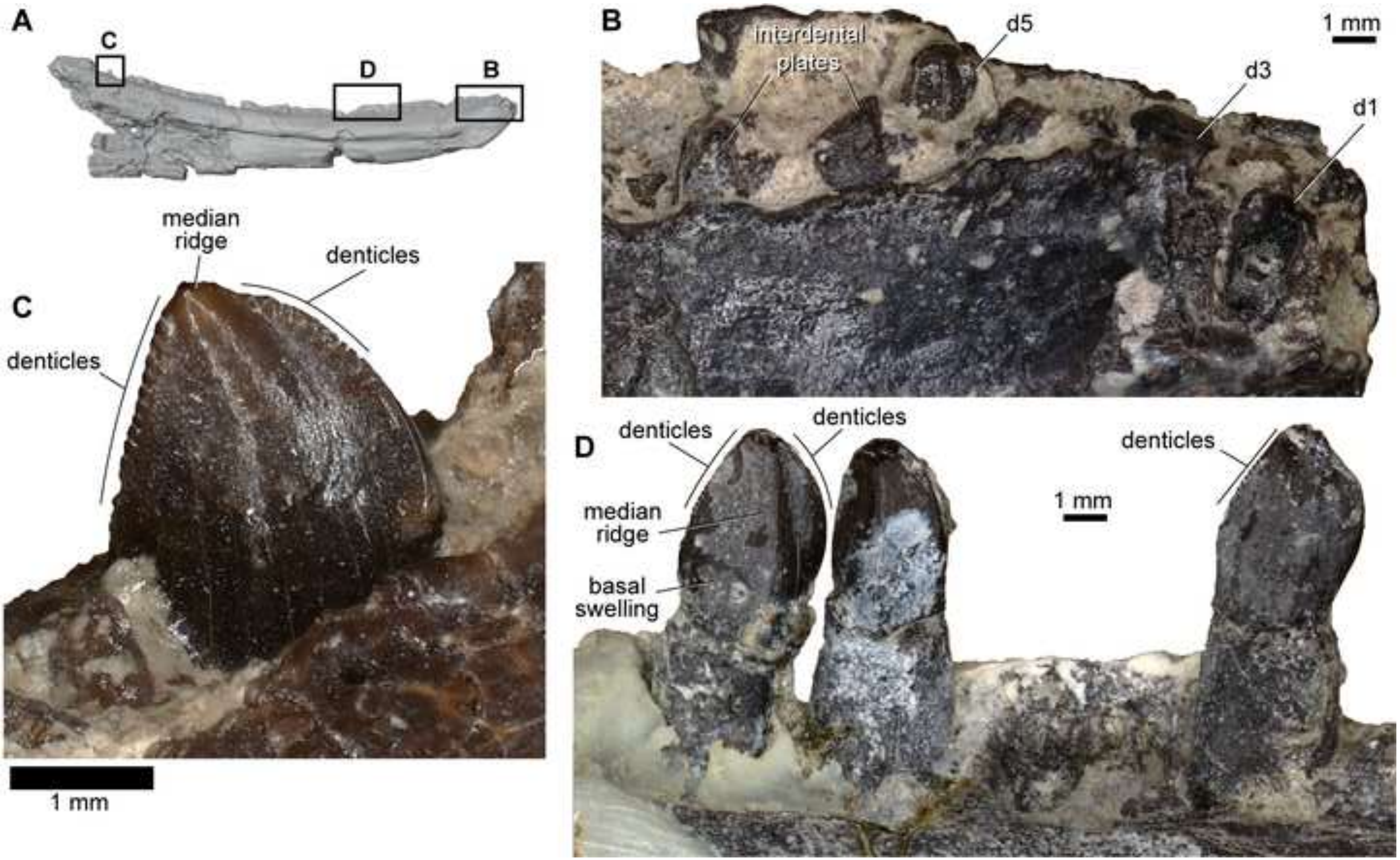

Click here to access/download;Figure;Fig 20.tif

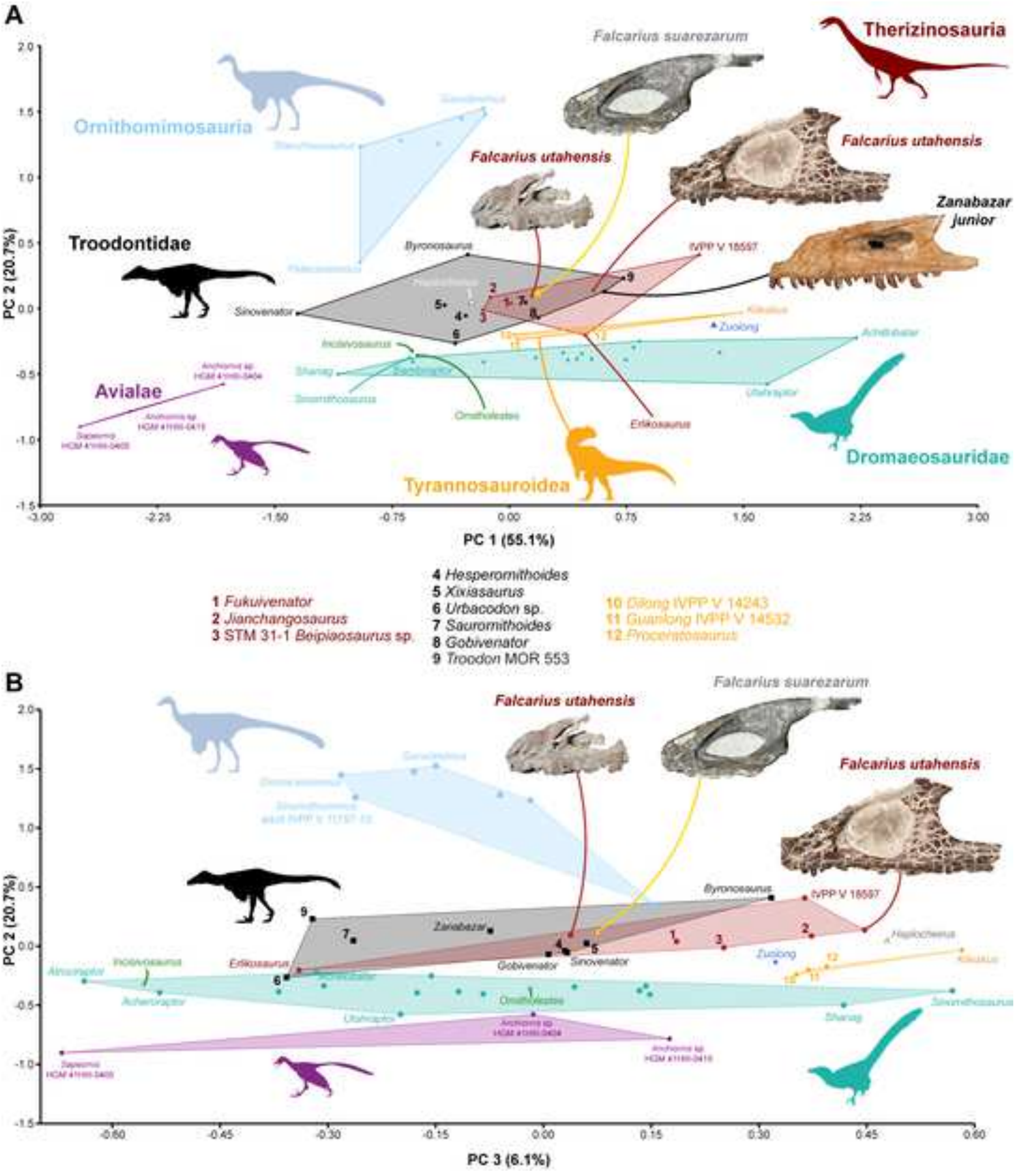

Click here to access/download;Figure;Fig 21.tif

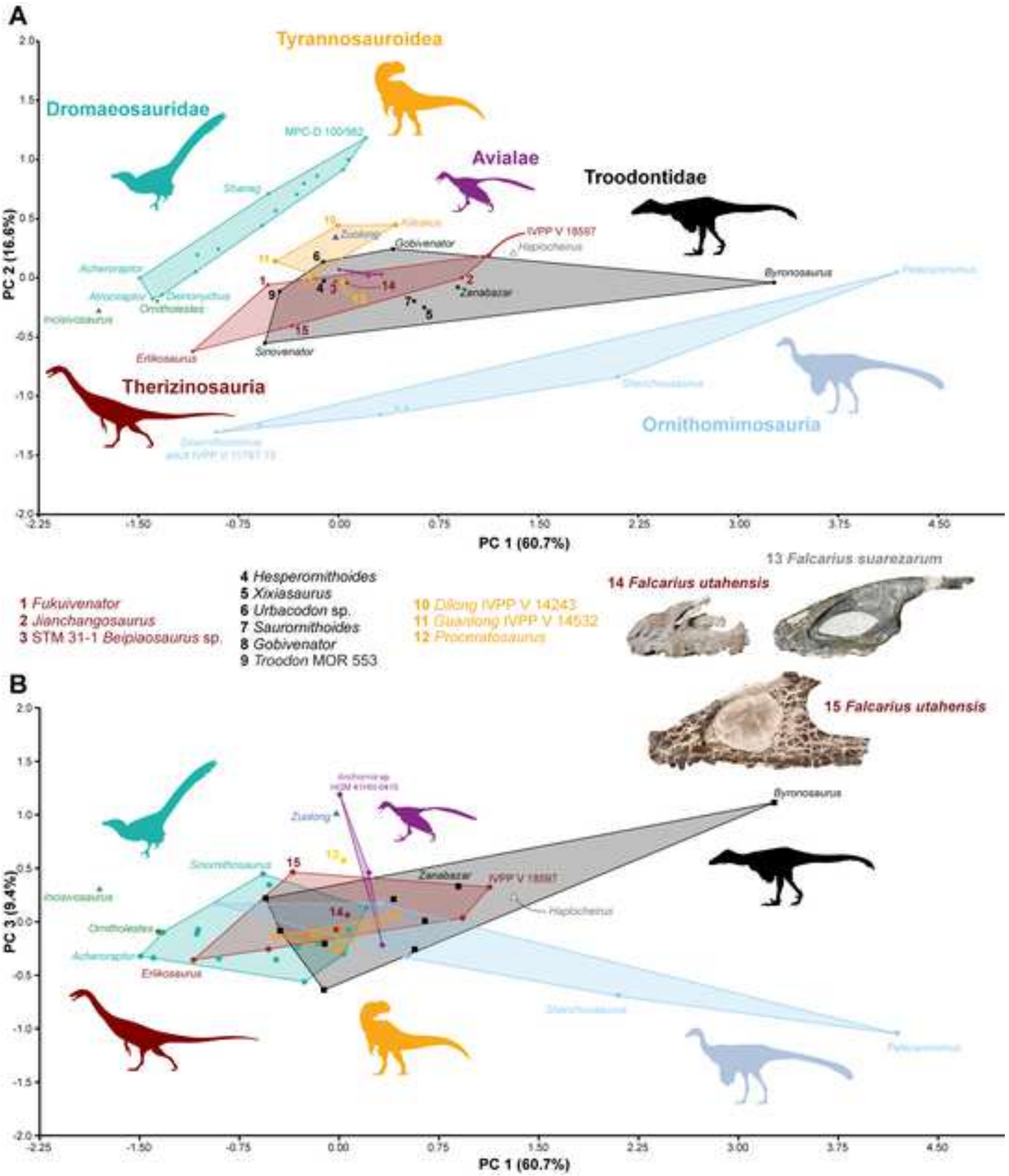

Fig 22 Click here to access/download;Figure;Fig 22.tif

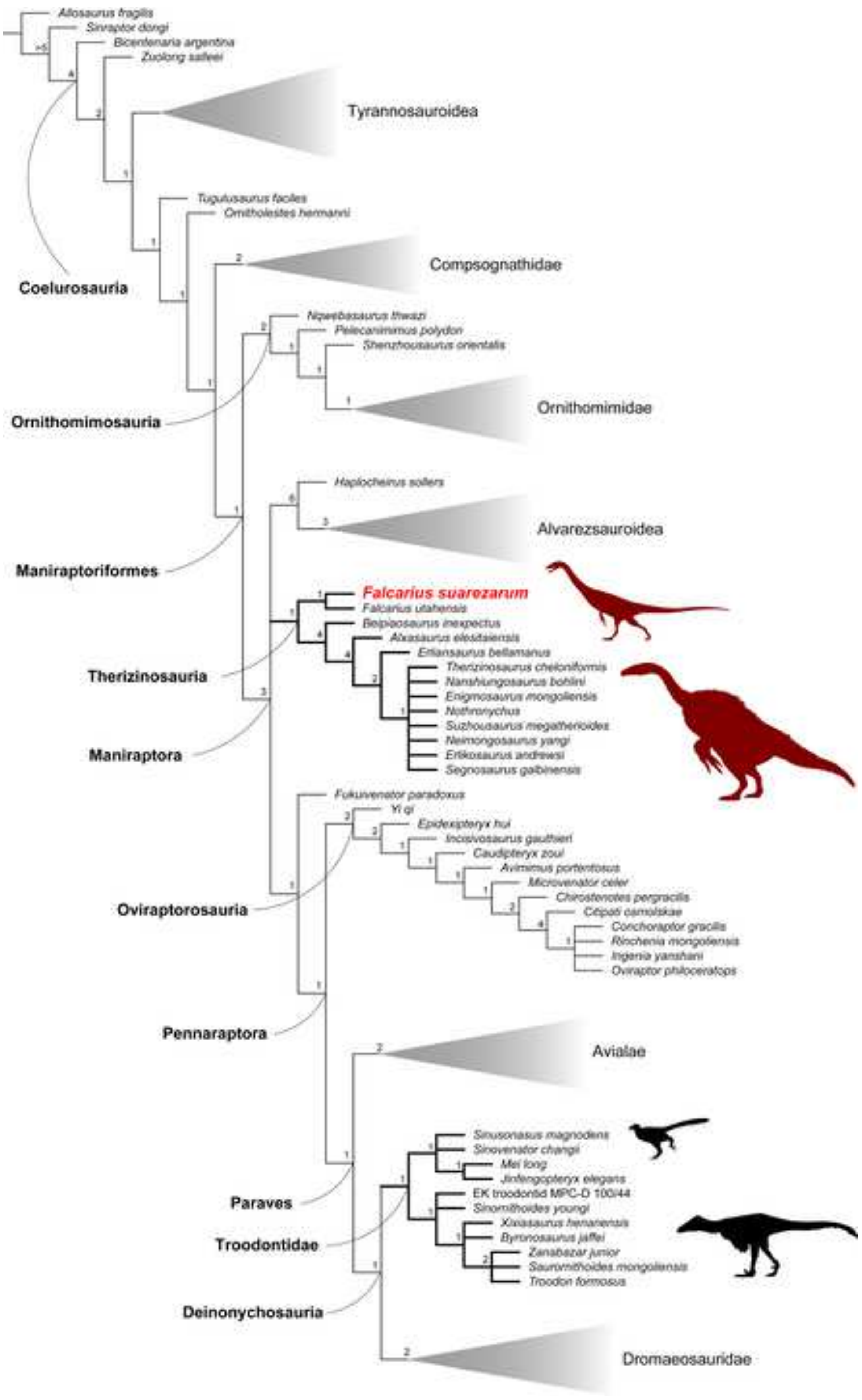

Fig 23

Click here to access/download;Figure;Fig 23.tif

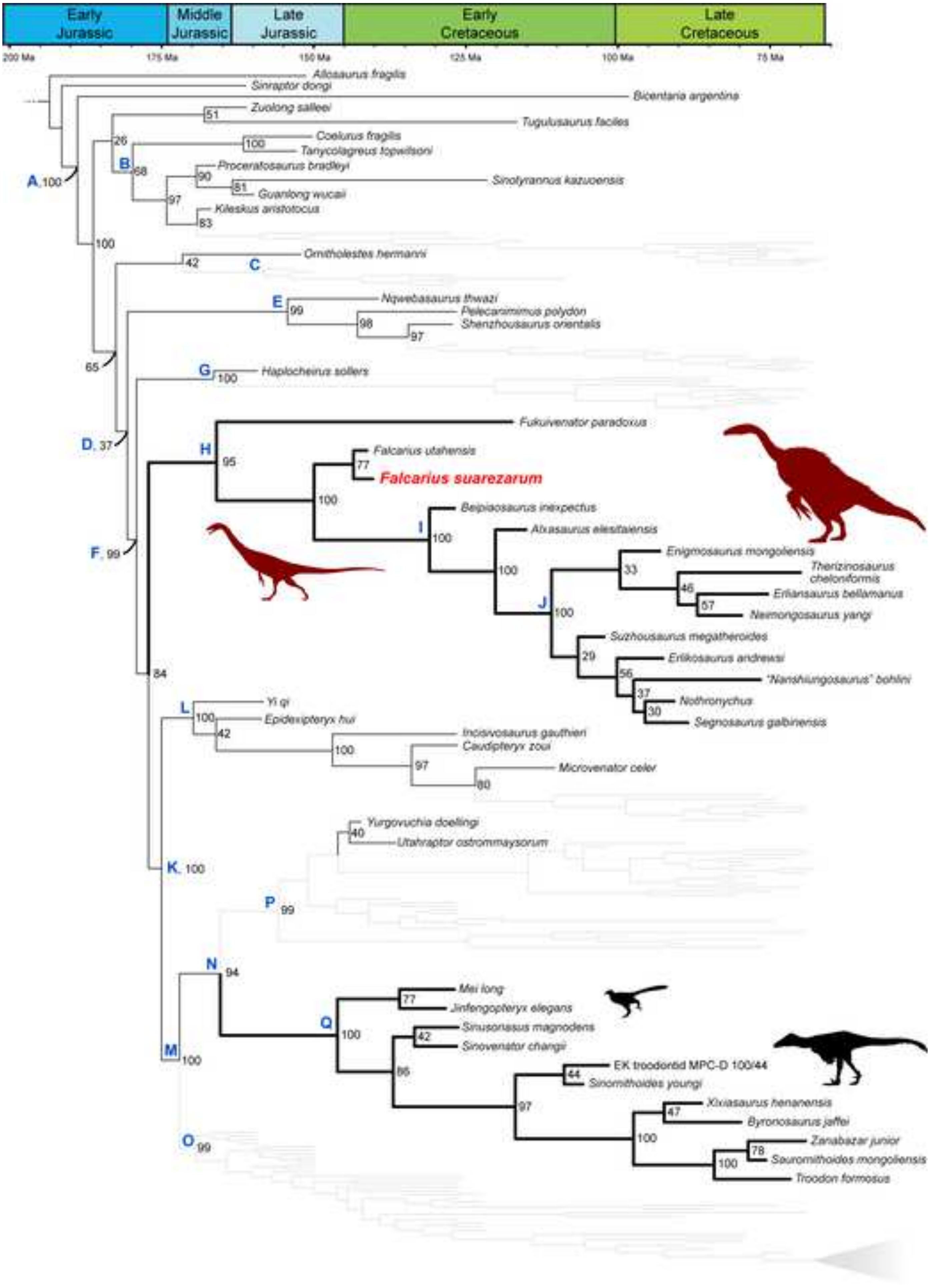

Click here to access/download
Supporting Information - Compressed/ZIP File Archive
Supporting Information.zip
