# Supplementary material for: Reassessment of the Early Cretaceous North American “troodontid” Geminiraptor suarezarum as a therizinosaurian and mosaic evolution of coelurosaurian maxillae
Source: PLoS One. 2026 Sep 30;21(9):e0356471. doi: 10.1371/journal.pone.0356471 (PMC13626363; doi:10.1371/journal.pone.0356471)
Supplement: Supplementary file 2 [file pone.0356471.s006.docx]

Please find below our responses to the thoughtful suggestions made by the editor and reviewer. We have also identified additional typographical errors upon revising the manuscript, and we have listed those changes here. For these typographical amendments, line numbers refer to those on the **resubmitted track changes version** of the manuscript.

Typographical errors:

**- Line 235:** added a space after “[11]”

**- Line 348:** changed “deinonychosaurs” to “deinonychosaurians”

**- Line 476**: changed “mono-dominant” to “monodominant”

**- Line 507**: Following several suggestions by reviewer elsewhere, changed “transversely” to “mediolaterally”

**- Line 693**: corrected spelling of “*miessleri*”

**- Line 783**: corrected spelling of “interalveolar”

**- Line 851**: changed “oviraptorosaurs” to “oviraptorosaurians”

**- Line 858–859**: changed “ornithomimosaurs” to “ornithomimosaurians”, “deinonychosaurs” to “deinonychosaurians”, and “oviraptorosaurs” to “oviraptorosaurians”

**- Line 868**: corrected spelling of “*salleei*”

**- Line 883**: corrected spelling of “Lautenschlager”

**- Line 887**: corrected spelling of “*salleei*”

**- Line 937, 942**: corrected grammar of “cranii”

**- Line 938**: corrected spelling of “*paradoxus*”

**- Line 1150**: changed full name “*Aorun zhaoi*” to abbreviation “*A. zhaoi*”

**- Line 1258**: corrected spelling of “*Hesperornithoides*”

**- Line 1284**: changed “ornithomimosaurs” to “ornithomimosaurians”

**- Line 1292**: corrected spelling of “*millenii*”

**- Line 1320, 1322, 1324**: changed “ornithomimosaurs” to “ornithomimosaurians”

**- Line 1369**: corrected spelling of “Therizinosauria”

**- Lines 1445–1448**: Changed font to Times New Roman

**- Line 1524**: corrected spelling of “*Martharaptor*”

**- Lines 1545–1581**: changed all instances of clade names ending in “-saurs” to “-saurians” (e.g., “ornithomimosaurs” to “ornithomimosaurians”)

**- Line 1568**: corrected punctuation and parentheses

**- Line 1676**: changed “oviraptorosaur” to “oviraptorosaurian”

**- Lines 1740–1745**: added text to Acknowledgements

- We have updated Fig 21B to include the numbered icons that were accidentally omitted in the original version

-------------------------------------------------------------------------------------------------------------------------------

Editor: The manuscript represents a relevant and well-supported contribution, with a clear anatomical description and an appropriate discussion of the taxonomic implications. However, some minor revisions are recommended before final acceptance. In particular, the authors should consider adjustments to the organization of part of the manuscript to improve the flow of the argument and treat with greater caution certain characters used in the differential diagnosis that may be based on limited sampling.

We thank the editor (and reviewer) for the positive comments and critical suggestions to improve this manuscript.

To the first point regarding adjustments to the organization in certain sections of the systematic paleontology (echoing comments of the reviewer): we acknowledge that discussion of potential pitfalls surrounding taxonomic assessments of bonebed material was out of place and potentially undermined our subsequent taxonomic assessments. We have moved this text, which was previously in the genus diagnosis, to the end of the *Falcarius suarezarum* species diagnosis, so that it occurs after our taxonomic arguments. We have also shortened this discussion and included a statement that reaffirms our taxonomic assessment.

We also understand the editor/reviewer concern related to differentially diagnostic characters based on limited sample sizes of cranial remains. We previously lacked explanatory text related to potential sources of variation outside of interspecific (e.g., taphonomic, intraspecific). To address this, we have included more detailed explanations in the descriptive text to affirm that the specimens in question (postorbitals, quadrate) are not taphonomically distorted beyond our ability to confidently interpret true morphology (i.e., we are not mis-interpreting taphonomic effects as species-level variation). We have also added text in the “Remarks” section of the *F. suarezarum* species diagnosis and in the Discussion (under “Intraspecific Variation”) that fleshes out our reasoning for distinguishing intra- and interspecific variation. Briefly, we highlight three points:

1) Overlapping size ranges of specimens from each assemblage suggest ontogenetic variation is not a major contributing factor to the observed morphological variation.
2) The position and relationships of pneumatic fossae are intraspecifically invariant in the cranium of archosaurs, including extant *Alligator* (Napoli 2024) and *F. utahensis* (Freimuth and Zanno 2025). For this reason, we interpret prominent differences in the pneumatic sinuses of the maxillae from each assemblage as representing species-level variation, despite having only one (variably taphonomically damaged) representative specimen from each assemblage.
3) Some diagnostic features of the postorbital and quadrate are only represented by one specimen from the Suarez Site. However, these features consistently differ from all known specimens from the Crystal Geyser Quarry, and these features are invariant among all specimens in the CGQ sample, which includes four postorbitals and eight quadrates of variable preservation quality. In the face of the intraspecific invariance of these features at CGQ, we thus consider the different morphology represented by the single Suarez Site specimens as interspecific.

Ultimately, larger sample sizes are needed to test these hypotheses, but the available evidence favors our species-level designations. We hope the editor and reviewer find these explanations sufficient to address their thoughtful suggestions.

-------------------------------------------------------------------------------------------------------------------------------

Reviewer #1: The authors reidentify a series of specimens, including the holotype of Geminiraptor originally described as a troodontid, as belonging to Falcarius, and further recognize them as representing a new species based on a number of subtle but consistent differences from the previously known species. The anatomical description is generally clear and well organized, and the conclusion that these specimens represent a distinct species appears reasonable. The broader implications of this reassessment are also thoughtfully considered. Overall, I find the manuscript to be a valuable contribution that is suitable for publication in this journal.

One point of concern is that the manuscript initially emphasizes factors that could potentially weaken the justification for recognizing two species, before moving directly into the systematic paleontology section. As a result, the reader may approach the taxonomic treatment with some hesitation. Clarifying this point, or slightly improving the structure of this part of the manuscript, may help the argument flow more smoothly.

In addition, although the extensive differential diagnosis is appreciated, some of the listed differences appear to be based on features that are represented by only a limited number of specimens in one or both taxa, making it difficult to fully exclude the effects of intraspecific variation or deformation. Given that the distinction between the two species seems clear even without some of these characters, the authors may wish to treat such potentially ambiguous features more cautiously.

Please also see the comments in the attached PDF for additional, more specific points.

We are grateful to the reviewer for their insightful suggestions and their time and efforts to improve this rather long manuscript. We address these general comments above alongside those to the editor, and we address each individual comment below. Line numbers here correspond to those on the **reviewer submitted pdf** version.

**- Line 173:** changed “Crystal Geyser Quarry” to “CGQ”

**- Line 328:** added “in other known” per reviewer suggestion

**- Line 334:** changed “dorsoventrally expanded” to “dorsoventrally deep” per suggestion

**- Line 344:** We have updated Fig 3 (*Falcarius* crania diagnosis) to include ventral view of the braincase, properly highlighting the hypertrophied basisphenoid recess.

**- Line 411**: We understand the confusion regarding our discussion of potential intraspecific variation here in the SysPaleo, prior to the species diagnoses. As discussed above in response to general comments, we have moved this discussion to the end of the *F. suarezarum* diagnosis, reduced the text, and clarified our taxonomic arguments.

**- Line 432**: Changed “transversely” to “mediolaterally”

**-Line 433:** This is an inherently difficult issue with small sample sizes; however we have added a paragraph in the Discussion under “Intraspecific variation” outlining our arguments for interpreting certain morphological variation as interspecific rather than intraspecific.

**- Line 441:** We agree that dividing the dentary dentition features in the figure is easier to comprehend, especially in the figure; however, we believe it is best to keep the combined features together in a single, detailed, robust diagnostic character. To accommodate this, we have updated the text in the diagnosis and Figs 5H–I with sub-labels (8a–c) as the reviewer suggested.

**- Line 479:** We agree with the reviewer—these images are difficult to interpret. We have replaced the photographs with 3D digital models (as per their suggestion) and included caudomedial view to better demonstrate the morphological variation in the caudal surface of the interfenestral bar. We have also included 3D models with the antorbital sinus and maxillary antrum reconstructed to display the differences in paranasal sinus relationships with respect to the interfenestral bar. Following suggestions of the reviewer for the dentary dentition characters, we have labelled these features in Fig 5 as “1a” and “1b”.

**- Line 484:** Regarding the differential feature related to the orbital surface of the postorbital as obscured (*F. suarezarum*) vs. unobscured (*F. utahensis*) in medial view: we have considered the effects of taphonomy on this trait, and we appreciate the concern of the reviewer. We exclude the damaged postorbital CEUM 73942 from the discussion of this trait because it is indeed taphonomically broken. However, CEUM 73787 is not deformed or distorted (there is some breakage along the caudal edge of the jugal process, but this does not affect the character in question). Because the morphology in CEUM 73787 is unaffected by taphonomic damage, and because it consistently differs from all known *F. utahensis* postorbitals (which we also consider unaffected by taphonomic distortion), we hypothesize that this represents species-level variation (a hypothesis that can be tested with the discovery of additional specimens). To clarify that these specimens are not distorted and well-preserved three dimensionally, we added text to the beginning of this descriptive section alongside the taphonomic notes.

**- Line 486:** We presume this highlighted section relates to questions regarding the effects of taphonomy as well, and we have a similar response as above. The intercondylar sulcus and the articular surfaces of the mandibular condyles of CEUM 73773 are damaged, but the remaining portions of the mandibular condyles are intact. Similarly, because the mandibular condyle arrangement of CEUM 73773 differs from all known quadrates of *F. utahensis*, we hypothesize that this represents a species difference (which can similarly be tested with the discovery of additional specimens in the future). We have made sure to emphasize the taphonomic patterns in the descriptive text here.

**- Line 507:** Thank you for catching the typo with the parentheses. We have included this, and also deleted the unnecessary semicolon preceding the references in this line.

**- Line 559:** We limited the identification of foramina in the maxilla rostral body to “at least two” because the rostral portion is broken and missing, and therefore could have housed additional foramina. We recognize the confusion with our writing, as well as the labeling of Fig 7, which we have updated to differentiate the foramina on the rostral body, and those that line the ventral margin (alveolar foramina). We have also updated the same labelling in Fig 8.

**- Line 597:** Text is highlighted, but no comment is present. We assume this was because the purpose of this sentence was confusing, so we deleted this sentence.

**- Line 618:** included “other” in front of “therizinosaurians”.

**- Line 625:** Changed “transversely” to “mediolaterally”.

**- Line 630:** We thank the reviewer for this helpful clarification, and we added text to clarify the morphology of the dorsal margin of the maxilla and interfenestral bar: “(which was collectively inset from the dorsal border of the antorbital fossa, presumably formed by the missing nasal)”.

**- Line 641:** we changed our unhelpful term “D-shaped” to “rostrally concave” to describe the caudal margin of the IFB.

**- Line 671–676:** Thank you for pointing this out. We have added clarifying text to indicate the differences in both Fig. 9F–I and Fig. 10F–I.

**- Line 689:** We have clarified the text relating to the position of the accessory foramen: “...occurs on the **maxilla** medial surface **at the junction of the rostral body and ascending ramus,** near the midpoint…”.

**- Line 705:** We changed “canals” to “foramina” as per the reviewer’s suggestion (which also matches the terminology in our Fig. 9).

**- Line 737:** Changed “transversely” to “mediolaterally”

**- Line 744:** We have included an additional sentence that clarifies the nature of the external nares along the maxillary ascending process and absence of a distinct opening on the IFB.

**- Line 811:** Changed “transversely” to “mediolaterally”.

**- Line 867:** Changed “transversely” to “mediolaterally”.

**- Line 878:** Thank you for catching this error, we changed “Fig 8” to “Fig 13”. We also understand that the resolution of the 3D models make identification of the temporo-orbital foramen difficult, so we included an inset, high-resolution image to indicate this opening.

**- Line 879, 880:** Changed “transversely” to “mediolaterally” and “transverse” to “mediolateral”.

**- Line 902:** Made “views” plural, and changed “transverse” to “mediolateral”

**- Line 936:** Thank you for catching this small but important error. Changed incorrect *“Fa.*” to “*Fu.*”

**- Line 939:** Thank you for correcting this; changed “right” to “left” quadrate.

**- Line 986–987:** This is a helpful clarification, and an oversight on our part. We have deleted this sentence.

**- Line 1014:** We agree, our phrasing is unclear. We amended this to describe the “articular surface faces medially”.

**- Line 1116:** Changed the regretful typo “detention” to proper “dentition”.

**- Line 1163:** We have included all loading scores of those variables mentioned in the main text. Complete distributions of loading scores and bar charts are included in the supplementary information.

**- Line 1209:** In all iterations of our principal component analyses, we grouped known therizinosaurians and ran the *Fa. suarezarum* maxilla on its own to test with which coelurosaurian clade(s) it shared the most overlap in morphospace. This iteration (Fig 21B) was the only one in which *Fa. suarezarum* fell outside of therizinosaurian morphospace. We recognize that this reasoning was not explained in the Materials and Methods or Results, so we added text to clarify in both sections.

**- Lines 1335–1338:** We clarified the gently arched circumorbital ridge that approaches the ventral margin of the squamosal process in *Fa. utahensis*, compared to the nearly right-angle circumorbital ridge that extends to the dorsal margin of the squamosal process in *Fa. suarezarum*. These descriptions now more closely match the phrasing used in the species diagnoses.

**- Line 1385:** Replaced “therizinosaurs” with “therizinosaurians”. Thank you for catching this.
