# Supplementary material for: Reassessment of the Early Cretaceous North American “troodontid” Geminiraptor suarezarum as a therizinosaurian and mosaic evolution of coelurosaurian maxillae
Source: PLoS One. 2026 Sep 30;21(9):e0356471. doi: 10.1371/journal.pone.0356471 (PMC13626363; doi:10.1371/journal.pone.0356471)
Supplement: Supplementary file 3 [file pone.0356471.s002.zip › S2_File_Morphometrics/Height corrected loadings/Height corrected loadings plot pc3.pdf]

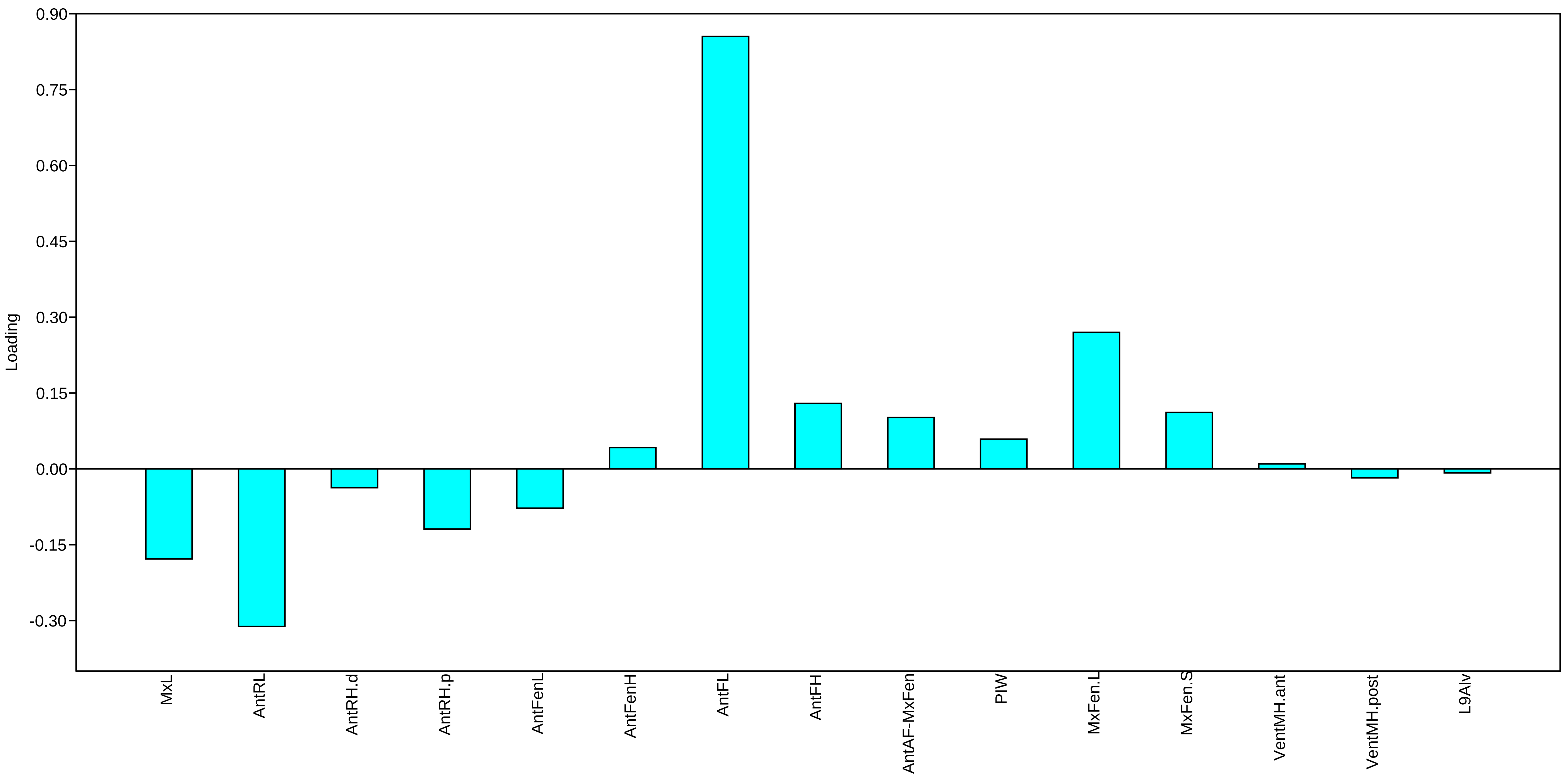
0.90
0.75
0.60
0.45
0.30
0.15
0.00
-0.15
-0.30
Loading
MxL
AntRL
AntRH.d
AntRH.p
AntFenL
AntFenH
AntFL
AntFH
AntAF-MxFen
PlW
MxFen.L
MxFen.S
VentMH.ant
VentMH.post
L9Alv
