# Supplementary material for: Reassessment of the Early Cretaceous North American “troodontid” Geminiraptor suarezarum as a therizinosaurian and mosaic evolution of coelurosaurian maxillae
Source: PLoS One. 2026 Sep 30;21(9):e0356471. doi: 10.1371/journal.pone.0356471 (PMC13626363; doi:10.1371/journal.pone.0356471)
Supplement: Supplementary file 3 [file pone.0356471.s002.zip › S2_File_Morphometrics/Log transformed loadings/log transformed Scree plot.pdf]

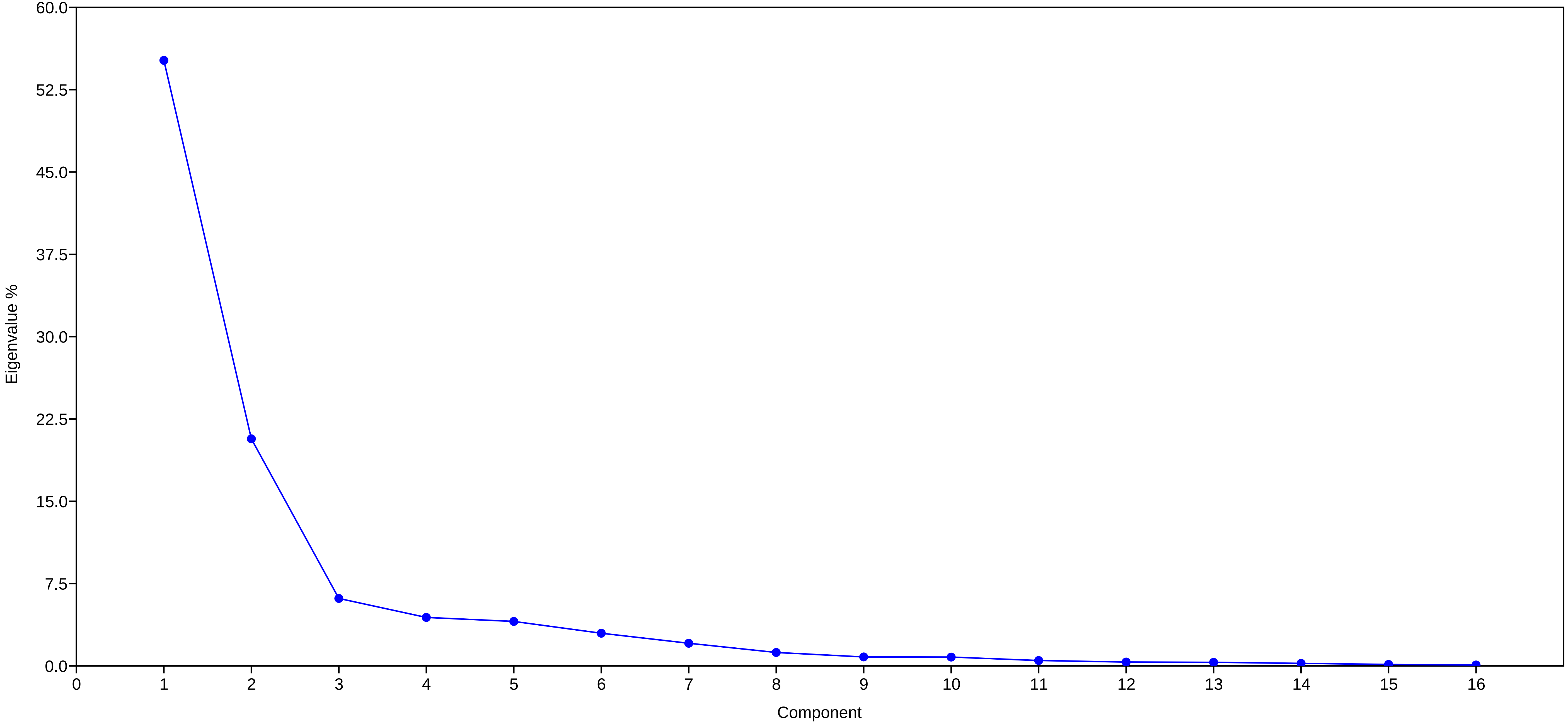
60.0
52.5
45.0
37.5
30.0
22.5
15.0
7.5
0.0
Eigenvalue %
0
1
2
3
4
5
6
7
8
9
10
11
12
13
14
15
16
Component
