# Supplementary material for: Reassessment of the Early Cretaceous North American “troodontid” Geminiraptor suarezarum as a therizinosaurian and mosaic evolution of coelurosaurian maxillae
Source: PLoS One. 2026 Sep 30;21(9):e0356471. doi: 10.1371/journal.pone.0356471 (PMC13626363; doi:10.1371/journal.pone.0356471)
Supplement: Supplementary file 4 [file pone.0356471.s003.zip › S3_File_Phylogenetics/Supplemental_trees_Figs_S1-S5.docx]

**Supplemental Figure S1.** 50% Majority-rule tree from parsimony analysis of Kobayashi et al. (2025) matrix including “*Geminiraptor” suarezarum* holotype maxilla only.

**Supplemental Figure S2.** 50% majority-rule tree from the parsimony analysis of Kobayashi et al. (2025) matrix including all *“Geminiraptor” suarezarum* cranial materials.

**Supplemental Figure S3.** Full compatibility consensus tree of tip-dated Bayesian analysis including *“Geminiraptor” suarezarum* holotype maxilla only. Horizontal axis represents millions of years. Numbers at nodes represent posterior probabilities as percentages.

**Supplemental Figure S4.** Half-compatibility consensus tree of tip-dated Bayesian analysis including *“Geminiraptor” suarezarum* holotype maxilla only. Horizontal axis represented millions of years. Numbers at nodes represent posterior probabilities as percentages.

**Supplemental Figure S5.** Full strict consensus tree of 70 MPTs (99,999 trees post-TBR branch swapping; length = 3395, consistency index = 0.319, retention index = 0.764). Numbers at nodes indicate Bremer support values.
