# Supplementary material for: Reassessment of the Early Cretaceous North American “troodontid” Geminiraptor suarezarum as a therizinosaurian and mosaic evolution of coelurosaurian maxillae
Source: PLoS One. 2026 Sep 30;21(9):e0356471. doi: 10.1371/journal.pone.0356471 (PMC13626363; doi:10.1371/journal.pone.0356471)
Supplement: Supplementary file 4 [file pone.0356471.s003.zip › S3_File_Phylogenetics/TWiG matrix/TWiG_character_list_Brusatte_etal_2014.docx]

**Brusatte et al. (2014) Character List	*Falcarius* *utahensis*	“*Geminiraptor*”	both**

**Character 1: Feathers, vaned feathers on forelimb, form:**

0: symmetric

1: asymmetric

**Character 2: Orbit, shape**

0: circular in lateral or dorsolateral view

1: dorsoventrally elongate

Note: A “dorsoventrally elongate” orbit was not explicitly defined by Turner et al. (2012) or previous versions of the TWiG dataset, but is here held to be any orbit that is greater than 1.5 times deeper dorsoventrally than long anterposteriorly at its midpoint. This character state is only present in some adult tyrannosauroid specimens among coelurosaurs. The presence of a circular orbit in *Alioramus* may be a consequence of the juvenile status of the one quality specimen of this taxon (IGM 100/1844), or alternatively, it could be a product of the dorsoventrally shallow and anteroposteriorly elongated snout of this taxon.

**Character 3: Postorbital, ventral ramus, projection into orbit: (ORDERED)**

0: does not project into orbit, suborbital process absent

1: projects into orbit, suborbital process present and large in adults and small and unpronounced in

sub-adults

2: projects into orbit, suborbital process present and large in sub-adults and adults

Note: Turner et al. (2012) and previous TWiG datasets utilized a binary character relating to the presence/absence of an anterior process projecting into the orbit. We here modify this character by splitting the “presence” state into two states, relating to the varying degree of postorbital projection in tyrannosauroids (to distinguish the hypertrophied condition in *Tyrannosaurus* and *Tarbosaurus* in which the postorbital projects strongly into the orbit in both sub-adults and adults). We have also reversed the 0 and 1/2 designation relative to Turner et al. (2012) so that “0” now refers to the lack of postorbital projection and “1” and “2” refer to the two conditions of postorbital projection, following Brusatte et al. (2010a). We have changed the score of *Haplocheirus* from present to absent based on Choiniere et al. (2010a).

**Character 4: Postorbital, shape of anterior (frontal) process in lateral view:**

0: straight

1: curving anterodorsally, such that dorsal border of temporal bar is dorsally concave

**Character 5: Postorbital, ventral ramus, orientation: (UNORDERED)**

0: parallels quadrate, lower temporal fenestra rectangular in shape

1: oriented strongly obliquely relative to quadrate, jugal and postorbital approach or contact quadratojugal to constrict lower temporal fenestra

2: oriented anteroventrally relative to the long axes of the quadrate and lacrimal, angle of postorbital ventral ramus long axis with the lacrimal long axis (if lacrimal is approximately vertical) greater than 30 degrees
Note: This is a modified version of the original TWiG character, which adds a third state denoting the anteroventrally oriented postorbital ventral rami of some basal coelurosaurs (e.g., tyrannosauroids).

**Character 6: Braincase, otosphenoidal crest, form and position:**

0: vertical on basisphenoid and prootic, and does not border an enlarged pneumatic recess

1: well developed, crescent shaped, thin crest forms anterior edge of enlarged pneumatic recess

**Character 7: Braincase, crista interfenestralis, location:**

0: confluent with lateral surface of prootic and opisthotic

1: distinctly depressed within middle ear opening

**Character 8: Braincase, subotic recess (pneumatic fossa ventral to fenestra ovalis):**

0: absent

1: present

Note: Carr et al. (2011) described a “subotic fossa and recesses” in *Teratophoneus*, but these are not similar to the large, invasive subotic recess of ornithomimosaurs and troodontids.

**Character 9: Basisphenoid recess: (UNORDERED)**

0: present between basisphenoid and basioccipital

1: entirely within basisphenoid

2: absent

Note: This multistate character is not considered ordered because there is not a clear nested set of primary homologies. The basisphenoid recess is primitive for coelurosaurs, so it is not clear whether the reduced and absent conditions form a transformational sequence. Therefore, the character is left unordered. The reduction of the basisphenoid recess to lie entirely within the basisphenoid in *Tyrannosaurus* and *Tarbosaurus* is most likely due to the extreme anterposterior foreshortening of the recess in these taxa (character 158 in Brusatte et al. 2010a).

**Character 10: Basisphenoid recess, posterior opening, form:**

0: single opening

1: divided into two small, circular foramina by a thin bar of bone

**Character 11: Parasphenoid, base of cultriform process (parasphenoid rostrum), pneumatization:**

0: not highly pneumatized

1: expanded and pneumatic (parasphenoid bulla present)

**Character 12: Braincase, basipterygoid processes, direction of projection: (UNORDERED)**

0: ventral or anteroventrally projecting

1: lateroventrally projecting

2: laterally projecting

**Character 13: Braincase, basipterygoid processes, form:**

0: well developed, extending as a distinct process from the base of the basisphenoid

1: processes abbreviated or absent

**Character 14: Braincase, basipterygoid processes, pneumatization:**

0: absent or very subtle, process solid

1: processes hollow, invaded by pneumatic recess (anterior tympanic recess and/or basisphenoid recess)

Note: Turner et al. (2012) scored hollow basipterygoid processes as absent in several tyrannosauroids. However, these are indeed present in taxa that have been CT scanned, including *Alioramus* (Bever et al. 2011), *Gorgosaurus* (Witmer and Ridgely 2009), and *Tyrannosaurus* (Witmer and Ridgely 2009). Therefore, we have scored the character as present in these tyrannosauroids and considered it questionable “?” in all tyrannosauroids (and other basal coelurosaurs) that have not been studied using CT. We anticipate that this character state will eventually be identified in many taxa once they have been the subject of CT study. We also suggest that there may be a difference between the extensively hollow basipterygoid processes of ornithomimosaurs and troodontids (which have long been noted without CT) and more subtle, less invasive recesses in other taxa (such as tyrannosauroids). For the time being, however, we consider these conditions homologous because it is difficult to differentiate them in a discrete manner.

**Character 15: Basisphenoid, basipterygoid recesses on dorsolateral surfaces of basipterygoid processes:**

0: absent

1: present

Note: This character refers only to the presence of discrete external pneumatic foramina or fenestrae on the lateral surface of the basipterygoid processes (or immediately above). Brusatte et al. (2010a, character 157) utilized a different character relevant to the ingroup relationships of tyrannosauroids, concerning the presence or absence of a single large, window-like fenestra in this region, which is present in juveniles of *Bistahieversor*, *Alioramus*, and *Daspletosaurus* (but not adults of *Bistahieversor* and *Daspletosaurus*). Because of its ontogenetically variable nature, we do not use the Brusatte et al. (2010a) character here, although we incorporate it into a character relating to the anterior tympanic recess below. Rather, we retain the original character of the TWiG dataset which refers to the simple presence or absence of pneumatic foramina on the basipterygoid processes.

**Character 16: Prootic, depression for pneumatic recess (Dorsal Tympanic Recess):** (**ORDERED)**

0: absent

1: present as dorsally open fossa on prootic/opisthotic

2: present as deep, posterolaterally directed concavity

Note: Brusatte et al. (2010a, character 163) utilized a similar character for tyrannosauroids, and we use those character scores here. Most notably, *Dilong* is scored as uncertain (not as possessing a dorsally open recess as in Turner et al. [2012]). *Guanlong*, however, does possess a dorsal tympanic recess.

**Character 17: Braincase, accessory tympanic recess dorsal to crista interfenestralis:** (**ORDERED)**

0: absent

1: small pocket present

2: extensive with indirect pneumatization

**Character 18: Braincase, caudal (posterior) tympanic recess: (ORDERED)**

0: absent

1: present as opening on anterior surface of paroccipital process

2: extends into opisthotic posterodorsal to fenestra ovalis, confluent with this fenestra

Note: Derived tyrannosauroids possess state “2”, as can be seen by their inflated and heavily pneumatized paraoccipital processes (Witmer and Ridgely 2009; Bever et al. 2011, 2013). *Guanlong* clearly does not possess such extensively inflated paroccipital processes, but in the absence of CT data it is uncertain whether it possesses state 0 or state 1. Because of this uncertainty we conservatively score it as “[01]”.

**Character 19: Braincase, exits of cranial nerves X-XII, location and form: (ORDERED)**

0: flush with surface of exoccipital

1: located together in a shallow bowl-like depression

2: located together in a deep, funnel-like depression

Note: We have modified the original binary character of Turner et al. (2012) and previous TWiG versions into an ordered multistate, to differentiate between two conditions of the “bowl-like depression”. The first condition, a shallow depression, is commonly seen in coelurosaurs. The second, an extremely deep funnel-like morphology, is present in some derived tyrannosauroids (Brusatte et al. 2010a, character 153). *Bistahieversor* and *Teratophoneus*, which were scored as “?” in Brusatte et al. (2010a), are now scored as possessing the funnel-like morphology based on recent reexaminations of both specimens by Thomas Carr (as noted in Bever et al. 2013).

**Character 20: Premaxilla, maxillary process, extent and articulation: (UNORDERED)**

0: contacts nasal to form posterior border of nares

1: reduced so that maxilla participates broadly in external naris

2: extends posteriorly to separate maxilla from nasal posterior to nares

**Character 21: Premaxilla and nasal, internarial bar, morphology of external surface:**

0: rounded

1: flat

**Character 22: Premaxilla, crenulated margin on buccal edge of bone:**

0: absent

1: present

**Character 23: External naris, position of posterior margin:**

0: farther anterior than antorbital fossa

1: nearly reaching or overlapping the anterior border of the antorbital fossa

**Character 24: Premaxilla, shape of conjoined left and right bones in ventral view: (ORDERED)**

0: acute, V-shaped

1: rounded, U-shaped, first two teeth oriented mediolaterally and third and fourth teeth oriented parasagitally
2: rounded, U-shaped, entire tooth row oriented mediolaterally

Note: We have expanded this character into consider this character into an ordered multistate to take into account variation in the “rounded” condition in tyrannosauroids, as denoted by Brusatte et al. (2010a, character 10). All non-tyrannosauroids with rounded snouts are scored for state 1, as are basal tyrannosauroids. Derived tyrannosauroids in which the entire premaxillary tooth row is oriented mediolaterally are scored for state 2.

**Character 25: Maxilla, anteromedial process (= “secondary palate”): (ORDERED)**

0: short

1: long, with extensive palatal shelves on maxilla

2: extremely elongated, extending back at least to the level of alveolus 4

Note: We have added a second derived state to the character of Turner et al. (2012) and previous TWiG analyses, to differentiate the extremely elongated anteromedial processes of tyrannosauroids, which extend back to at least the level of alveolus 4. The second derived state is present in all score-able tyrannosauroids, as well as ornithomimosaurs (e.g., *Struthiomimus*, *Pelecanimimus*, *Nqwebasaurus*) and the long-snouted dromaeosaurid *Austroraptor*). It is possible that this character may eventually diagnose a more inclusive clade of basal coelurosaurs, given that elongate anteromedial processes are present in nearly all coelurosaurs. However, it is currently difficult to distinguish between the long and extremely long processes of many coelurosaurs, because many specimens are preserved on slabs and therefore fine details of the medial maxilla are not visible. It is clear, however, that derived paravians such as *Dromaeosaurus* and *Deinonychus*, while possessing long anteromedial processes, do not possess the extremely elongate morphology of tyrannosauroids (e.g.., Currie 1995). This is also true of basal coelurosaurs such as *Zuolong* (Choiniere et al. 2010b). Whether compsognathids may have possessed the tyrannosauroid condition is somewhat unclear, because most preserved specimens are either preserved on slabs or are not preserved in disarticulation (precluding observation of the medial surface of the maxilla). However, high resolution photographs of *Compsognathus* published by Göhlich et al. (2006) indicate that this taxon had a somewhat elongate palatal process, and definitely did not possess the extremely elongated condition of tyrannosauroids (therefore, we score it for state 1).
**Character 26: Maxilla, palatal shelf, midline ventral “tooth-like” projection:**

0: absent, palatal shelf flat

1: present

**Character 27: Maxilla, maxillary fenestra: (ORDERED)**

0: absent

1: present, fenestra occupies less than half of the depressed area between the anterior margins of the antorbital fossa and antorbital fenestra

2: present, fenestra large and takes up most of the space between the anterior margins of the antorbital fenestra and fossa

Note: the second derived state refers only to the huge fenestrae of some troodontids, which are so large that they take up nearly the entire antorbital fossa region anterior to the antorbital fenestra, with only a thin strut of bone separating the two openings. Other characters referring to nuances of the size and position of the fenestrae are given below.

**Character 28: Maxilla, maxillary fenestra, location:**

0: situated at anterior border of antorbital fossa

1: situated posterior to anterior border of fossa

Note: This is equivalent to character 17 in Brusatte et al. (2010a). Among tyrannosauroids, *Daspletosaurus*, *Tarbosaurus*, *Tyrannosaurus*, and *Sinotyrannus* have state 0. We here score *Dilong* for state 1, contra Turner et al. (2012). In *Dilong* the maxillary fenestra approaches the anterior border of the antorbital fossa, but it doesn’t reach the border.

**Character 29: Maxilla, promaxillary fenestra:**

0: absent

1: present

Note: We score *Eotyrannus* as possessing the fenestra, based on personal observation of the holotype (MIWG 1997.550)

**Character 30: Nasal, pneumatization: (UNORDERED)**

0: apneumatic or poorly pneumatized

1: with extensive pneumatic fossae, especially along posterodorsal rim of naris

2: with 2-3 large pneumatic openings, set into a fossa, on the lateral surface above the antorbital fenestra, leading into an extensive internal pneumatic cavity

NOTE: Turner et al. (2012) utilized a binary character, and we added a new state based on Brusatte et al. (2010a, character 43). We do not consider the two pneumatic states to be directly homologous or form a nested series of homology, because of distinct differences in the region of the external pneumatopores (posterodorsal rim of naris vs. midpoint of bone) and the form of the pneumatopores (several pneumatopores posterodorsal to the rim of the naris and within the narial fossa vs. 2-3 discrete pneumatic openings centered in a fossa). The outgroups and some basal tyrannosauroids possess condition 2, whereas oviraptorosaurs possess condition 1. Turner et al. (2012) scored the outgroups (*Allosaurus* and *Sinraptor*) for state 0, and this was correct when the character was a simple binary distinction between no/poor pneumaticity and the extreme pneumaticity of oviraptorosaurs. The new condition added here recognizes morphological similarity between the outgroups and some basal coelurosaurs (basal tyrannosauroids), and probably optimizes as the ancestral condition for Coelurosauria.
**Character 31: Jugal and postorbital, contribution to postorbital bar:**

0: contribute equally to postorbital bar

1: ascending process of jugal reduced and descending process of postorbital ventrally elongate

**Character 32: Jugal, dorsoventral height beneath lower temporal fenestra:**

0: tall, twice or more as tall dorsoventrally as it is wide transversely

1: very short, jugal rod-like

**Character 33: Jugal, pneumatic recess in posteroventral corner of antorbital fossa:**

0: present

1: absent

Note: This character refers to an internal pneumatic recess of the jugal, which hollows out some or all of the bone internally. As shown by Tahara and Larsson (2011), the presence of a discrete jugal diverticulum (located in the jugal portion of the antorbital fossa) does not always equate to the presence of an internal recess hollowing the jugal. The presence of an internal recess can be confidently assessed by the presence of a discrete pneumatopore externally, which leads into the recess. *Pelecanimimus* possesses this pneumatopore (LH 7777) and is here scored for state 0, whereas all other ornithomimosaurs are scored for state 1 because they lack the external pneumatopore, and in the case of *Ornithomimus* are known to lack the internal recess based on CT scans (Tahara and Larsson 2011).

**Character 34: Jugal, medial jugal foramen:**

0: present on medial surface ventral to postorbital bar

1: absent

Note: Turner et al. (2012) scored the foramen as absent in *Dilong*, but the medial surface of the jugal is obscured in the holotype specimen, which has not yet been the subject of CT study. Therefore, we have changed the score to “?”.

**Character 35: Quadratojugal, shape:**

0: without horizontal process posterior to ascending process (reversed “L” shape)

1: with horiziontal posterior process (i.e., inverted ‘T’ or ‘Y’ shape)

**Character 36: Jugal and quadratojugal, fusion:**

0: absent

1: present, the two bones are not distinguishable from one another

**Character 37: Lacrimal, supraorbital crests in adult individuals: (UNORDERED)**

0: absent

1: dorsal crest above orbit

2: lateral expansion anterior and dorsal to orbit

Note: Following Turner et al. (2012), we consider this an unordered character because we do not hypothesize primary homology between state 1 (discrete crests or tubercles along the dorsal margin of the lacrimal, as present in outgroups and tyrannosauroids) and state 2 (a lamina-like triangular sheet that expands laterally above the orbit, as present in troodontids). Turner et al. (2012) scored *Shuvuuia* for state 1, but we have rescored this as state 0 because this taxon does not possess the discrete crest/tubercle along the dorsal margin of the lacrimal as in outgroups and tyrannosauroids.

**Character 38: Lacrimal, pneumatic foramina opening laterally at the junction of the anterior and ventral processes above the antorbital fenestra: (ORDERED)**

0: absent

1: present, extent of pneumaticity limited to partially hollowing the bone in the region where the anterior and ventral rami meet

2: present and extensive, completely hollowing the bone where the anterior and ventral rami meet

Note: Turner et al. (2012) used a binary character to distinguish between the presence/absence of lacrimal pneumaticity, and we add a third state to distinguish the greatly extensive pneumatic recess of some tyrannosauroids. Therefore, this is now an ordered character. Turner et al. (2012) scored *Eotyrannus* as lacking lacrimal pneumaticiy, but observation of the type specimen shows that it has a shallow pneumatic recess with external pneumatopores, as in *Guanlong*, *Dilong*, and other basal tyrannosauroids.

**Character 39: Lacrimal, posterodorsal process: (ORDERED)**

0: absent, lacrimal is inverted ‘L’ shaped or ‘7’ shaped in lateral view

1: present, lacrimal ‘T’ shaped in lateral view

2: present but reduced, anterodorsal process much longer than posterodorsal process

Note: Turner et al. (2012) and previous TWiG datasets have referred to the presence of an “anterodorsal process”, which creates a T-shaped lacrimal, in this character. This T-shaped condition contrasts with the “inverted L” shape of most theropod lacrimals. However, the inverted L shape is due to a pronounced anterior process along the dorsal margin of the bone (the anterodorsal process) and the lack of any posterior process along the dorsal margin (the posterodorsal process). Therefore, we have reworded the character to refer specifically to the posterodorsal process. We also consider this an ordered character, because state 2 (the presence of a posterodorsal process that is much shorter than the anterodorsal process) is a sub-condition of state 1, meaning that there is a nested primary homology hypothesis. Brusatte et al. (2010a, character 48) utilized a character distinguishing the T-shaped and 7-shaped lacrimals of tyrannosauroids. We utilize this as a separate character below. *Zulong*, which possesses a small posterodorsal process that appears large because of the presence of an autapomorphic notch ventral to it, is scored for state 0.

**Character 40: Prefrontal, exposure in dorsal view: (ORDERED)**

0: large, dorsal exposure similar to that of lacrimal, forms much of orbital rim and usually separates or nearly separates frontal and lacrimal

1: greatly reduced in size, not exposed widely along the orbital rim and allows for wide contact between frontal and lacrimal

2: absent

Note: We have slightly reworded this character based on language in Brusatte et al. (2010a, character 111). Turner et al. (2012) scored *Dilong* for a reduced frontal, based on the description in Xu et al. (2004). However, personal observation of the holotype (IVPP V14243) reveals that it is difficult to distinguish the prefrontal from surrounding bones, so we conservatively score this taxon as “?” following Brusatte et al. (2010a).

**Character 41: Frontals, shape of conjoined left and right elements in dorsal view:**

0: triangular, narrowing anteriorly as a wedge between nasals

1: rectangular, end abruptly anteriorly, suture with nasal transversely oriented

Note: Brusatte et al. (2010a, character 113) distinguished between the broad triangular shape of most tyrannosauroid frontals and the anteroposteriorly foreshortened shape of derived tyrannosaurid frontals. However, the frontals of all tyrannosauroids are triangular, so we retain the current character and add a new character below to distinguish between the tyrannosauroid morphologies.

**Character 42: Frontal, outline of anterior emargination of supratemporal fossa:**

0: straight or slightly curved

1: strongly sinusoidal and reaching onto postorbital process

**Character 43: Frontal, form of articulation between postorbital process and frontal orbital margin in dorsal view:**

0: smooth transition from orbital margin

1: sharply demarcated from orbital margin

Note: Turner et al. (2012) scored some derived tyrannosaurids for state “1”, but we here rescore these as state “0,” because they do not possess the strongly demarcated and discrete postorbital processes that are seen in some dromaeosaurids (which state “1” refers to).

**Character 44: Frontal, form of lateral margin of lacrimal (or prefrontal) suture:**

0: smooth

1: with discrete notch

**Character 45: Parietals, form of dorsal surface and presence of sagittal crest(s): (ORDERED)**

0: flat, lateral ridge borders supratemporal fenestra, sagittal crest (or crests) absent

1: convex with very low sagittal crest (or crests) along midline

2: convex with well developed sagittal crest (or crests)

Note: This character was unordered in Turner et al. (2012), but we here make it an ordered character because states 1 and 2 are a nested series of homologies defining progressively more prominent sagittal crests, whereas state 0 refers to the absence of a crest. This character subsumes character 122 in Brusatte et al. (2010a), which referred to the presence/absence of sagittal crests in tyrannosauroids.

**Character 46: Parietals, fusion of left and right elements:**

0: unfused

1: fused on the midline in sub-adults and adults (when known)

Note: This character is equivalent to character 127 in Brusatte et al. (2010a).

**Character 47: Squamosal, quadratojugal (descending) process, orientation of long axis:**

0: dorsoventral or slightly oblique, essentially parallels quadrate shaft

1: anteroposterior, nearly perpendicular to quadrate shaft

Note: This character is equivalent to character 92 of Brusatte et al. (2010a). Brusatte et al. (2010a) scored *Bistahieversor* for state 0, but we here change this to state 1. This miscoding was an error in the Brusatte et al. (2010a) analysis.

**Character 48: Squamosal, contact of descending (ventral) process with quadratojugal:**

0: present

1: absent, squamosal does not contact quadratojugal

**Character 49: Squamosal, posterolateral shelf overhanging quadrate head:**

0: absent

1: present

**Character 50: Quadrate, orientation of shaft when in articulation:**

0: vertical

1: strongly inclined anteroventrally so that ventral end lies far forward of dorsal end

**Character 51: Quadrate shaft, form of lateral surface:**

0: straight, without any prominent processes projecting laterally; quadrate foramen not visible in lateral view

1: with broad, triangular process along lateral edge of shaft contacting squamosal and quadratojugal above an enlarged quadrate foramen, which is often partially or largely visible in lateral view

Note: We have slightly reworded the character to explain it more clearly.

**Character 52: Foramen magnum, shape:**

0: subcircular, slightly wider than tall

1: oval, taller than wide

**Character 53: Occipital condyle, mediolateral width and dorsoventral height of neck linking condyle to remainder of braincase:**

0: approximately the same width and height as the condyle

1: constricted in width and height relative to the condyle

Note: We have reworded this character to make it clearer here, so that it refers specifically to a discrete neck that is constricted in width and height relative to the width of the condyle.

**Character 54: Paroccipital processes, shape:**

0: elongate mediolaterally and slender dorsoventrally compared to mediolateral length, with dorsal and ventral edges nearly parallel

1: process short mediolaterally, deep dorsoventrally compared to mediolateral length, with convex distal end

**Character 55: Paroccipital processes, orientation in posterior view:**

0: straight, projects laterally or slightly posterolaterally

1: downturned, distal end curves ventrally and is pendant

Note: This character is not equivalent to character 150 in Brusatte et al. (2010a), which refers to a distinct ventral flange at the distal end of the paroccipital processes of some tyrannosauroids. This character is utilized below.

**Character 56: Paroccipital process, orientation of dorsal edge:**

0: straight

1: twisted anterolaterally at distal end

**Character 57: Ectopterygoid, form of the opening into the pneumatic fossa on the ventral surface:**

0: constricted opening relative to fossa

1: widely opened fossa

Note: Turner et al. (2012) scored *Albertosaurus* and *Gorgosaurus* for state “0”, which is unusual among coelurosaurs. Our observations of tyrannosauroids do not reveal any discrete differences between these taxa and other tyrannosauroids, both basal (e.g., *Guanlong*, *Xiongguanlong*) and derived (e.g., *Alioramus*, *Tarbosaurus*, *Tyrannosaurus*) forms. Therefore, all tyrannosauroids are scored “1” here.

**Character 58: Ectopterygoid: dorsal recess:**

0: absent

1: present

Note: This character does not refer to the principal pneumatic recess of the ectopterygoid, which hollows out much of the bone and opens via a large pneumatopore on the ventral surface. Rather, it refers to a separate dorsal recess present in some dromaeosaurids.

**Character 59: Pterygoid, morphology of the posterior flange for articulation with quadrate and epipterygoid:**

0: well developed

1: reduced in size or absent

Note: This character has been reworded here for clarity. See Balanoff et al. (2009) for a good description of state 1, which is present in oviraptorosaurs and a handful of other coelurosaurs.

**Character 60: Palatine and ectopterygoid, articulation between the two bones:**

0: absent, the two bones separated from each other by the pterygoid

1: present, the two bones contact each other

**Character 61: Palatine, jugal process:**

0: present, palatine tetraradiate in shape

1: absent, palatine triradiate in shape

**Character 62: Skull, suborbital fenestra, size:**

0: large, similar in anteroposterior length to the anteroposterior length of the orbit

1: reduced in size (less than one quarter orbital length) or absent

**Character 63: Dentary, form of symphyseal region in dorsal or ventral view: (ORDERED)**

0: approximately straight anteroposteriorly, paralleling lateral margin of the remainder of bone; conjoined dentaries narrow

1: recurved slightly medially relative to remainder of bone, conjoined dentaries U-shaped

2: recurved strongly medially relative to remainder of bone, conjoined dentaries forming broad U-shaped muzzle

Note: This character has been reworded for clarity.

**Character 64: Dentary, orientation of dorsal margin of symphyseal region in lateral view:**

0: in line with the remainder of the dorsal (alveolar) margin of the dentary

1: downturned relative to the remainder of the dorsal margin

**Character 65: Lower Jaw, coronoid prominence (usually located on surangular):**

0: absent

1: present

**Character 66: Dentary, contribution to the dorsal margin of the external mandibular fenestra: (ORDERED)**

0: absent or slight, without discrete posterodorsal process

1: present, with discrete posterodorsal process above anterior end of fenestra

2: present and extensive, with elongate posterodorsal process extending over most of fenestra

**Character 67: Dentary, morphology of lateral surface:**

0: flat, tooth row approximately in line with the remainder of the lateral surface

1: bearing lateral ridge, tooth row inset from remainder of lateral surface

**Character 68: Dentary, shape:**

0: subtriangular in lateral view, with dorsoventral depth expanding posteriorly

1: strap-like in lateral view, with subparallel dorsal and ventral edges and a consistent dorsoventral depth across its length
Note: Turner et al. (2012) scored some derived tyrannosauroids (*Eotyrannus*, *Gorgosaurus*, *Tyrannosaurus*, *Daspletosaurus*) for state “1”, whereas the basal tyrannosauroids *Dilong* and *Proceratosaurus* were scored for state “0”. This character was introduced to the TWiG matrix based on the descriptions of Currie (1995), who noted that some dromaeosaurids (and other coelurosaurs) had dentaries that maintained an approximately even depth across their entire lengths, unlike the dentaries of most theropods that funnel out in depth posteriorly (so-called “subtriangular” dentaries). The subtriangular condition is indeed present in basal tyrannosauroids, but also in more derived tyrannosauroids, contra Turner et al. (2012) (e.g., Brochu 2003; Currie 2003; Carr et al. 2011; Brusatte et al. 2012a). We here score all tyrannosauroids for state “0”. Turner et al. (2012) scored *Eotyrannus* for state “1”, seemingly because the preserved portions of the left and right dentaries of the holotype (MIWG 1997.550) are gracile and do not expand posteriorly. However, neither of these dentaries are complete posteriorly, so the degree of expansion cannot be assessed. Therefore, we here score *Eotyrannus* as “?”.

**Character 69: Dentary, primary neurovascular foramina on lateral surface, arrangement:**

0: distinct but superficial foramina

1: distinct foramina lie within a deep and sharp groove across the middle and posterior regions of the dentary

Note: This character is equivalent to character 176 in Brusatte et al. (2010a). It has long been utilized in the TWiG matrix, with state 1 scored for a handful of coelurosaurs. Brusatte et al. (2010a) recognized that a distinct and sharp groove is also present in the basal tyrannosauroids *Guanlong*, *Proceratosaurus*, and *Sinotyrannus*.

**Character 70: Lower jaw, external mandibular fenestra, shape**

0: oval

1: subdivided by a spinous anterior process of the surangular

**Character 71: Lower jaw, internal mandibular fenestra (opening between splenial and prearticular on medial surface of mandible), size and shape:**

0: small and slit-like

1: large and rounded

Note: This character was introduced into the TWiG dataset based on Currie (1995), who describe an unusual morphology of the internal mandibular fenestra in some dromaeosaurids, in which this opening is large and circular. This contrasts with the smaller and more ovoid opening of most theropods, which is often reduced to a small slit. Turner et al. (2012) scored *Tyrannosaurus* for a large and rounded fenestra, but as shown by Brochu (2003) this opening, although absolutely large due to the large size of Tyrannosaurus, is not circular and is smaller in relation to the size of the dentary than in dromaeosaurids. Therefore, *Tyrannosaurus* and all other tyrannosauroids with known splenials are here scored for state 0. This state can be inferred in isolated splenials by the size and shape of the notch along the posterior margin, which forms the anterior margin of the internal mandibular fenestra.

**Character 72: Surangular, foramen in lateral surface of surangular anterior to the mandibular articulation: (ORDERED)**

0: absent

1: present but small

2) present and large, approximately 30% of the dorsoventral depth of the posterior surangular

Note: This multistate ordered character combines two characters in the Turner et al. (2012) dataset: character 74 that scores the presence or absence of the surangular foramen and character 258 that scores variability in size of the foramen. The latter character is also used by Brusatte et al. (2010a, character 179) to distinguish between the small and extremely large foramina of some tyrannosauroids. This ordered multistate better encapsulates the full range of variation among not only coelurosaurs, but also tyrannosauroids (basal taxa such as *Guanlong* lack the foramen all together, whereas intermediate taxa such as *Eotyrannus* have a small foramen and derived tyrannosauroids have a derived enlarged foramen). Turner et al. (2012) scored *Eotyrannus* as “?”, but the recent realization that the holotype (MIWG 1997.550) includes surangular material shows that *Eotyrannus* has a small foramen.

**Character 73: Splenial, exposure in lateral view:**

0: not widely exposed on lateral surface of mandible

1: exposed as a broad triangle between dentary and angular on lateral surface of mandible

**Character 74: Lower jaw, coronoid ossification, presence and shape: (ORDERED)**

0: present as a large, triangular bone (fused with the supradentary)

1: present but reduced to a thin splint

2: absent

**Character 75: Articular, elongate and slender medial process emanating from retroarticular process (can project medially, posteromedially, or dorsomedially):**

0: absent

1: present

Note: Turner et al. (2012) scored *Dilong* and *Daspletosaurus* as lacking the process, and *Gorgosaurus* and *Tyrannosaurus* as possessing it. We could not confirm any observation of the articular medial process on *Dilong*, so we score this taxon as “?”. *Daspletosaurus* has the same general morphology of *Tyrannosaurus* and *Gorgosaurus*, which a pronounced dorsally and medially extending process at the posteromedial corner of the retroarticular process (Currie 2003). Therefore, we score it for state 1. We also have identified this process in *Tarbosaurus* and *Proceratosaurus*.

**Character 76: Articular, retroarticular process, presence and shape: (UNORDERED)**

0: present, short and stout, with a distinct region between the glenoid and the portion of the retroarticular process to which the jaw depressors attached

1: present, elongate and slender

2: present but extremely reduced, with no (or only a very short) margin between the glenoid and the muscle attachment region

Note: We have modified this character to include an additional state signifying the extremely reduced retroarticular processes of tyrannosauroids. In tyrannosauroids, including basal taxa such as *Guanlong* and *Dilong*, the retroarticular process is so short that there is essentially no separation between the mandibular glenoid and the attachment site (on the retroarticular process) for the jaw depressor muscles. This is not the case in *Tanycolagreus*, which has a longer retroarticular process and a wider margin between the glenoid and muscle attachment site (Carpenter et al. 2005a, fig. 2.4). In tyrannosauroids lacking an articular, the short retroarticular process can be inferred with a high degree of confidence by the short retroarticular region of the surangular, which articulates with the angular. This inferral is based on the one-to-one correspondence of the short surangular process and short articular retroarticular process in all known tyrannosauroids.

**Character 77: Lower jaw, glenoid articular surface for mandible, anteroposterior length:**

0: approximately as long as distal quadrate condyles

1: twice or more as long as distal quadrate condyles, allowing anteroposterior movement of mandible

Note: This character can usually be confidently inferred from the shape of all or part of the glenoid articular surface on the surangular and articular. Oviraptorosaurs, which possess state 1, have a radically different morphology compared to other theropods. In oviraptorosaurs, the quadrate condyles do not tightly articulate with the mandibular glenoid, and the lack of a clear, corresponding fit can be seen by observing the surangular and articular.

**Character 78: Premaxilla, teeth:**

0: present

1: absent

**Character 79: Premaxillary dentition, size of second premaxillary tooth:**

0: approximately equivalent in size to other premaxillary teeth

1: markedly larger than third and fourth premaxillary teeth

**Character 80: Maxilla, teeth:**

0: present

1: absent

**Character 81: Maxillary and dentary teeth, serrations: (UNORDERED)**

0: present on all known teeth

1: some teeth without serrations on mesial (anterior) carina (except at base in *S. mongoliensis*)

2: all known teeth without serrations on both mesial and distal carinae

Note: This character is considered unordered, because we do not hypothesize a nested sequence of primary homology. In other words, we do not hypothesize that there is a clear transformational sequence from serrations to only distal serrations to no serrations, although we recognize that this is a possibility that needs testing without assuming that the character is ordered. We score *Zuolong* for state 1. Choiniere et al. (2010b) described the mesial carina of an isolated lateral tooth as lacking serrations, but considered it difficult to rule out the absence of serrations across the entire carina because of the missing apical tip of the tooth. We feel that enough of the tooth is preserved to show that serrations certainly weren’t present across most of the length of the carina, and if were present, were restricted to the very apical tip of the tooth. We consider this equivalent to state 1.

**Character 82: Maxillary and dentary teeth, size:**

0: large

1: small (25-or more teeth in dentary when complete series is observable)

Note: The “large” condition is the normal condition for theropods in which 10-20 distinct maxillary teeth and 10-20 distinct dentary teeth are present. The “small” condition refers to the higher number of tightly packed teeth in the maxillae and dentaries of some coelurosaurs. Although a complete or near-complete maxilla and/or dentary are needed to score this character confidently, usually the size of individual teeth can be used to predict the state of this character.

**Character 83: Dentary teeth, implantation:**

0: in separate alveoli

1: set in open groove

**Character 84: Maxillary and dentary teeth, serrations (denticles):**

0: large

1: small

Note: Turner et al. (2012) and previous versions of the TWiG matrix have followed Farlow et al. (1991) in quantifying this difference. In the current dataset, along with previous TWiG matrices, the “large” condition refers only to the pronounced, coarse denticles of therizinosauroids and troodontids (~1-5 serrations per millimeter), whereas other coelurosaurs are scored for the “small” condition (~more than 5 serrations per millimeter).

**Character 85: Maxillary and dentary teeth, serrations, form:**

0: simple, convex, and approximately perpendicular to the long axis of the tooth

1: large, hooked and pointing toward the tip of the crown on the distal and often mesial carinae

**Character 86: Maxillary and dentary teeth, constriction between root and crown:**

0: present

1: absent, root and crown confluent

**Character 87: Dentary teeth, spacing between teeth:**

0: evenly spaced across tooth row

1: anterior dentary teeth smaller, more numerous, and more closely appressed than those in middle of tooth row

**Character 88: Dentary, interdental plates, form:**

0: distinct internal plates absent (although a homologue to the plates is probably present)

1: distinct, clearly demarcated interdental plates medially between teeth

Note: As reviewed by Turner et al. (2012), state 0 refers to the condition in dromaeosaurids and troodontids in which clear, distinct, unambiguously demarcated interdental plates are absent. It is likely, however, that bony tissue homologous to the interdental plates is present in this region but has been morphologically transformed.

**Character 89: Premaxillary tooth crowns, position of mesial carina: (ORDERED)**

0: along mesial margin of tooth, tooth cross section sub-oval to sub-circular

1: rotated distally on premaxillary teeth 1 and 2, such that anterior teeth have an asymmetrical cross section (D-shaped, with highly convex labial surface and flat lingual surface, with narrow spacing between mesial and distal carinae)

2: rotated distally on all premaxillary teeth, such that all teeth are D-shaped in cross section.

Note: Turner et al. (2012) and previous TWiG matrices have utilized a binary character relating to tooth cross sectional shape, differentiating “sub-oval” and “sub-circular” teeth, which are present in the outgroups and most coelurosaurs, from “D-shaped” teeth, which are present in tyrannosauroids and a few other coelurosaurs. We note that some outgroups (allosauroids) and many coelurosaurs do possess premaxillary teeth that are roughly D-shaped in cross section, due to a labial margin that is somewhat convex and a lingual margin that is somewhat flat (and always flatter than the labial margin). For this character, however, we define D-shaped teeth based on the morphology of the mesial carina. A “D-shaped” tooth is held to be a tooth in which the mesial carina is rotated strongly distally, such that it is present on the lingual surface of the tooth. This condition is seen almost exclusively in tyrannosauroids, and roughly follows the definition of D-shaped teeth by Currie et al. (1990), Choiniere et al. (2010b), and previous authors. This condition is always associated with a highly convex labial surface and a flat lingual surface, on which the two carinae are only very narrowly separated. In contrast, the somewhat D-shaped teeth of allosauroids and other coelurosaurs exhibit wider separation between both carinae on the lingual surface and a less convex labial surface. Therefore, these morphologies are not considered homologous to the “D-shaped” condition as defined here, although we recognize that there may be some homology shared between these taxa and coelurosaurs with true D-shaped teeth that is not present in more basal theropods such as coelophysoids, ceratosaurs, and some basal tetanurans (assessing this hypothesis awaits further study of theropod teeth more generally). Furthermore, following Brusatte et al. (2010a, character 196), we divide the “D-shaped” condition into two character states, which we consider to be nested homologies. Although these characters refer to the presence “D-shaped” morphology of either some (state 1) or all (state 2) of the tooth row, we here score all taxa lacking any sign of “D-shaped” teeth with rotated mesial carinae for state 0. It is possible that some of these taxa that do not preserve entire premaxillary tooth rows may eventually be scored for state 1, but we conservatively score them 0 here to minimize missing data.

**Character 90: Cervical vertebrae, number:**

0: ≤10

1: 12 or more

**Character 91: Axis, epipophyses, form:**

0: absent or poorly developed as a small pyramidal mound, not extending past posterior edge of postzygapophyses

1: large, rugose, and posteriorly directed flange, extending far beyond postzygapophyses

Note: This character is equivalent to Brusatte et al. (2010a, character 210). The form of the axial epipophyses in tyrannosauroids conforms to the form of the epipophyses in the anterior-middle postaxial cervicals, so if these cervicals are well preserved they can be used as a confident proxy for scoring this character. Because this correspondence is not necessarily clear for coelurosaurs as a whole, we only use anterior-middle cervicals as a proxy for tyrannosauroids.

**Character 92: Axis, neural spine, form of dorsal portion:**

0: flared transversely

1: compressed mediolaterally

**Character 93: Cervical vertebrae, epipophyses, position:**

0: placed distally on postzygapophyses, above postzygapophyseal facets

1: placed proximally, anterior to postzygapophyseal facets

**Character 94: Cervical vertebrae, position of centrum in anterior cervical vertebrae:**

0: terminates level with or anterior to the posterior extent of the neural arch

1: extending beyond the posterior limit of the neural arch

**Character 95: Cervical vertebrae, carotid process on posterior cervical vertebrae:**

0: absent

1: present

**Character 96: Cervical vertebrae, shape of anterior articular surface of anterior cervical centra:**

0: subcircular or square in anterior view

1: distinctly wider than high, kidney shaped

**Character 97: Cervical vertebrae, neural spines, shape in dorsal view:**

0: anteroposteriorly long

1: short and centered on neural arch, giving arch an “X” shape in dorsal view

Note: This is equivalent to character 211 in Brusatte et al. (2010a).

**Character 98: Cervical vertebrae, number of pneumatic foramina on lateral surface of centra:**

0: one on each side

1: two on each side

**Character 99: Cervical and anterior trunk vertebrae, form: (UNORDERED)**

0: amphiplatyan or weakly opisthocoelous (anterior surface flat or weakly convex, posterior surface is flat or weakly concave)

1: strongly opisthocoelous (anterior surface is convex and posterior surface concave)

2: at least partially heterocoelous

Note: Although the cervicals of tyrannosauroids and some other coelurosaurs are often referred to as “opisthocoelous,” this character distinguishes between very weak opisthocoely in which the anterior surface is only very slightly convex (state 0, considered homologous to the amphiplatyan condition) and strong opisthocoely in which the anterior face is strongly convex and fits into a deep concave socket on the posterior surface of the preceding vertebra (state 1). This latter condition is present in *Dilong* and *Eotyrannus* among tyrannoauroids, *Compsognathus*, some alvarezsaurids, and rarely among other coelurosaurs.

**Character 100: Dorsal vertebrae, hypapophyses in anterior trunk vertebrae:**

0: absent or very small

1: large and pronounced

**Character 101: Dorsal vertebrae, parapophyses in posterior trunk vertebrae, form:**

0: flush with neural arch

1: distinctly projected on pedicels

**Character 102: Dorsal vertebrae, hyposphene-hypantrum articulations:**

0: absent

1: present

**Character 103: Dorsal vertebrae, opposing zygapophyses on the same vertebra: (ORDERED)**

0: abutting or nearly abutting one another above neural canal, opposite hyposphenes (if present) meet to form a single structure (lamina or rectangular projection)

1: zygapophyses placed distinctly lateral to neural canal and hyposphenes (if present) separated as two widely-spaced laminae, which are joined medially by an inset web of bone

2: zygapophyses placed distinctly lateral to neural canal and hyposphenes (if present) separated as two widely-spaced lamina, which are separated medially by a deep groove (the inset web of bone in state 1 is absent)

Note: We have modified this character by dividing state 1 of Turner et al. (2012), which scores for the absence/presence of abutting postzygapophyses, into two states. Both of these states are applicable to taxa with widely separated postzygapophyses and hyposphenes on the midline. Most coelurosaurs have this condition, in which separate left and right hyposphenes take the form of vertical sheets, which are distinctly separated from each other. However, the region between the separated hyposphenes can either house a web of bone, which is only slightly inset from the hyposphenes (state 1), or the web of bone is lost and the hyposphenes and zygapophyses are separated by a deep groove (state 2). Some coelurosaurs have left and right hyposphenes that are appressed to each other on the midline due to closely appressed left and right zygapophyses (*Alxasaurus*, derived tyrannosauroids). This is also the condition in outgroups (e.g., *Allosaurus*), and these taxa and the outgroups are scored for state 0. This character is ordered because the states describe a progressive sequence of conjoined-separation-loss of separating web.

**Character 104: Cervical vertebrae, pneumaticity:**

0: absent

1: present

**Character 105: Dorsal vertebrae, transverse processes of anterior dorsals, form:**

0: long (in mediolateral direction) and thin (in anteroposterior direction)

1: short (in mediolateral direction), wide (in anteroposterior direction), and only slightly inclined

**Character 106: Dorsal vertebrae, neural spines, mediolateral expansion of dorsal end:**

0: absent

1: present, expanded to form ‘spine table’

**Character 107: Dorsal vertebrae, scars for interspinous ligaments, position:**

0: terminate at apex of neural spines

1: terminate below apex of neural spine

**Character 108: Sacral vertebrae, number: (ORDERED)**

0: 5 or less

1: 6

2: 7

3: 8

4: 9

5: 10

6: 11 or more

7: 15 or more

**Character 109: Sacral vertebrae, fusion of zygapophyses:**

0: absent, or zygapophyses partially fused to each other but still retaining the morphology of the original discrete structures

1: present, completely fused zygapophyses forming a sinuous ridge in dorsal view

**Character 110: Sacral centra, ventral surface of posterior sacrals, form: (UNORDERED)**

0: gently rounded, convex

1: ventrally flattened, sometimes with shallow sulcus

2: centrum strongly constricted transversely, ventral surface keeled

**Character 111: Sacral vertebrae, pneumatic foramina on lateral surfaces of centra: (ORDERED)**

0: absent on sacral vertebrae

1: present on anterior sacrals only

2: present on all sacrals

**Character 112: Sacral vertebrae, morphology of the posterior articular face of the last sacral centrum:**

0: flat or slightly concave

1: convex

**Character 113: Caudal vertebrae, change in morphology of free caudals along the tail:**

0: present, with distinct transition point from shorter centra with long transverse processes proximally to longer centra with small or no transverse processes distally

1: absent, vertebrae homogeneous in shape, without transition point

**Character 114: Caudal vertebrae, location of transition point along the tail: (ORDERED)**

0: begins distal to the 10th caudal vertebra

1: between the 7^th^ and 10th caudal vertebra

2: or proximal to the 7^th^ caudal vertebra

**Character 115: Caudal vertebrae, morphology of anterior caudal centra: (UNORDERED)**

0: tall, oval in cross section

1: with box-like centra in caudals I-V

2: anterior caudal centra laterally compressed with ventral keel

**Character 116: Caudal vertebrae, neural spines, form:**

0: simple, undivided

1: separated into anterior and posterior alae throughout much of caudal sequence

**Character 117: Caudal vertebrae, neural spines on distal caudals (distal to ~caudal 15), form: (ORDERED)**

0: present as a low ridge

1: absent

2: absent and location of spine replaced by midline sulcus in center of neural arch

Note: Turner et al. (2012) scored *Dilong* for state 1, but we conservatively rescore this taxon as “?” because we have been unable to observe the caudal vertebrae directly, and also based on the illustrations in Xu et al. (2004), which seem to indicate that there may indeed be remnants of the neural spines on caudals in the vicinity of caudal 15 (state 0). Turner et al. (2012) may have scored this taxon as state 1 based on Xu et al.’s (2004) illustrations of extreme distal caudal vertebrae, which do lack neural spines. However, this is also the case in the extreme distal caudals of tyrannosaurids (e.g., *Tyrannosaurus*) and outgroups (*Allosaurus*), both of which Turner et al. (2012) scored for state 0. What we recognize as state 1 here is the condition in taxa such as *Compsognathus* (Peyer 2006), in which neural spines cease to be recognizable as discrete structures around ca. caudal 15 (in *Tyrannosaurus* and *Allosaurus* this happens much more posteriorly in the tail, ca. caudal 30: Brochu 2003).

**Character 118: Caudal vertebrae, length of prezygapophyses of distal caudals: (UNORDERED)**

0: between 1/3 and whole centrum length

1: extremely long (up to 10 vertebral segments long in some taxa)

2: strongly reduced or absent, terminate at approximately the anterior level of the centrum

3: prezygapophyses present but negligible in size, clasping the posterior surface of neural arch of preceding vertebrae, postzygapophyses negligible (this is an autapomorphy of *Ichthyornis dispar*)

Note: Turner et al. (2012) scored *Dilong* for state 2, but personal observation of the holotype and the figures of Xu et al. (2004) show that state 0 is the correct score.

**Character 119: Caudal vertebrae, number: (ORDERED)**

0: more than 40

1: 35-40

2: 25-35

3: 8-25

4: less than 8 free caudal vertebrae, tail very short

Note: We have modified this character by breaking down what was previously a single state (25-40 caudals) into two states, which better allows this character to differentiate the moderately elongate tails of ornithomimosaurs and alvarezsauroids (which have approximately 35 vertebrae) from the shortened tails of therizinosauroids, ornithomimosaurs, and many paravians (which have approximately 25 vertebrae).

**Character 120: Chevrons, form of chevrons from proximal part of tail:**

0: long and slender, proximal end short anteroposteriorly and shaft cylindrical

1: short and stout, proximal end elongate anteroposteriorly and shaft flattened and plate-like

Note: Turner et al. (2012) scored various compsognathids for state 1, but we have rescored these taxa for state 0, as their proximal chevrons are elongate bones (with anteroposteriorly short proximal ends) that are proportionally similar to other taxa with state 0, such as outgroups (*Allosaurus*) and tyrannosauroids (e.g., *Tyrannosaurus*), and distinct from the short and stout proximal chevrons of other taa scored for state 1 (such as dromaeosaurids).

**Character 121: Chevrons, form of chevrons from distal part of tail: (ORDERED)**

0: simple

1: anteriorly bifurcate

2: bifurcate at both ends

**Character 122: Cervical ribs, shaft: (UNORDERED)**

0: slender and longer than vertebra to which they articulate

1: broad and shorter than vertebra

2: extremely thin and slender, hair-like

Note: We have modified this character to include a third state designating the extremely thin and hair-like cervical ribs of compsognathid-grade theropods, as recognized by Göhlich and Chiappe (2006). This character is unordered because there is no clear set of nested homologies or no clear character transformation sequence.

**Character 123: Ossified uncinate processes: (ORDERED)**

0: absent

1: present and unfused to ribs

2: present and fused to ribs

**Character 124: Ossified ventral (sternal) rib segments:**

0: absent

1: present

**Character 125: Gastralia, lateral gastral segment, size:**

0: shorter than medial one in each lateral-medial gastralium set

1: longer than the medial one

Note: We have modified this character following Claessens (2004). Claessens showed that in outgroups (*Allosaurus*) and tyrannosaurids (*Gorgosaurus*, *Tyrannosaurus*) the medial gastral segment is longer than the lateral gastral segment (state 0), contra the scores in Turner et al. (2012) and previous TWiG matrices. Furthermore, Norell and Makovicky (1997) showed that in *Velociraptor* the lateral segment is longer than the medial segment (state 1), which was affirmed by Claessens (2004) but contra the score in Turner et al. (2012). We suspect that scores 0 and 1 may have been swapped for some or all taxa accidentally in the TWiG matrix. Because of the confusion of scores in the original TWiG matrix, we conservatively take all character scores from Claessens (2004) and leave all other taxa as “?”, unless observed personally by S. Brusatte or specifically reported in the literature.

**Character 126: Sternum, ossified sternal plates, fusion:**

0: absent, left and right plates separate in adults

1: present, left and right plates fused

**Character 127: Sternum, lateral xiphoid process posterior to costal margin:**

0: absent

1: present

**Character 128: Sternum, groove on anterior edge for reception of coracoid:**

0: present

1: absent

**Character 129: Sternum, position of articular facet for coracoid (conditions may be determined by the position of the the articular facet on coracoid in taxa without ossified sternum):**

0: anterolateral or more lateral than anterior

1: almost anterior

**Character 130: Furcula, hypocledium**: (**ORDERED)**

0: absent

1: present as tubercle

2: present as an elongate process

**Character 131: Scapula, orientation of acromion margin:**

0: continuous with blade

1: anterior edge laterally everted relative to blade

**Character 132: Coracoid, expansion of posterolateral surface ventral to glenoid fossa:**

0: unexpanded

1: posterolateral edge of coracoid expanded to form triangular subglenoid fossa bounded laterally by enlarged coracoid tuber

**Character 133: Scapula and coracoid, fusion:**

0: absent, two bones separate

1: present, two bones fused into scapulacoracoid

**Character 134: Coracoid, shape in lateral view: (UNORDERED)**

0: subcircular, with shallow ventral blade

1: subquadrangular with extensive ventral blade

2: shallow ventral blade with elongate posteroventral process

3: height more than twice width, coracoid strut-like

**Character 135: Scapula and coracoid, form of their articulation:**

0: form a continuous arc in posterior and anterior views

1: coracoid inflected medially relative to scapula, scapulocoracoid ‘L’ shaped in lateral view

**Character 136: Scapula and coracoid, glenoid fossa, orientation of articular surface:**

0: faces posteriorly or posterolaterally

1: faces laterally

**Character 137: Scapula, length compared to length of humerus:**

0: longer

1: approximately same length or shorter

Note: In *Guanlong*, the scapula and humerus are approximately the same length (Xu et al. 2006), so this taxon is scored for state 1. The same scoring is used for all ornithomimosaurs, which have humeri that are slightly longer than the scapula (Osmólska et al. 1972) or have humeri and scapulae of approximately the same length (e.g., Nicholls and Russell 1985; Kobayashi and Lü 2003; Kobayashi and Barsbold 2005; Makovicky et al. 2010).

**Character 138: Humerus, deltopectoral crest, extent and morphology: (UNORDERED)**

0: large and distinct, proximal end of humerus quadrangular or triangular in anterior view

1: deltopectoral crest less pronounced, forming an arc rather than being quadrangular

2: deltopectoral crest very weakly developed, proximal end of humerus with rounded edges

3: deltopectoral crest extremely long and rectangular

**Character 139: Humerus, deltopectoral crest, form of anterior surface:**

0: smooth

1: with distinct muscle scar near lateral edge along distal end of crest for insertion of biceps muscle

**Character 140: Ulna, olecranon process, size:**

0: weakly developed

1: distinct and large

**Character 141: Ulna, morphology of distal articular surface (dorsal condyle and dorsal trochlea in birds):**

0: flat

1: convex, semilunate surface

**Character 142: Ulna, morphology of proximal surface:**

0: a single continuous articular facet

1: divided into two distinct fossae (one convex, the other concave) separated by a median ridge

**Character 143: Lateral proximal carpal (ulnare?), shape in proximal view:**

0: quadrangular

1: triangular

**Character 144: Distal carpals in contact with metacarpals, number:**

0: two separate carpals, one covering the base of metacarpal I (and perhaps contacting metacarpal II) the other covering the base of metacarpal II

1: a single distal carpal capping metacarpals I and II

**Character 145: Semilunate distal carpal, size: (UNORDERED)**

0: well developed, covering all of proximal ends of metacarpals I and II

1: small, covers about half of base of metacarpals I and II

2: covers bases of all metacarpals

3: covers MC II and MC III

**Character 146: Metacarpal I, length: (ORDERED)**

0: less than half the length of metacarpal II

1: approximately half of the length (~50-70%) of metacarpal II

2: subequal in length to metacarpal II

Note: We have modified the character of Turner et al. (2012) to include a new state, signifying the condition in some tyrannosauroids (and outgroups and other coelurosaurs) in which metacarpal I is approximately half of the length of metacarpal II. In essence, we have divided the original “half or less of the length of metacarpal II” character into two distinct states. This follows the character of Brusatte et al. (2010a, character 252). We have also removed the state in Turner et al.’s (2012) character that refers to the autapomorphically short and robust metacarpals I of alvarezsaurids, which are wider transversely than long proximodistally. We now provide a separate character denoting the presence or absence of this peculiar morphology below.

**Character 147: Third manual digit, size:**

0: present and large, phalanges present

1: reduced to no more than metacarpal splint

Note: This character is equivalent to Brusatte et al. (2010a, character 249).

**Character 148: Manual unguals, curvature: (ORDERED)**

0: strongly curved, flexor margin deeply concave

1: weakly curved, flexor margin shallowly concave (third ungual may be straight)

2: all manual unguals straight

Note: We have divided the original character of Turner et al. (2012) and previous TWiG matrices, which related to both the curvature and size of the flexor tubercle, into two separate characters. This character now refers to the degree of curvature, whereas a separate character has been added below for the size of the flexor tubercle. Furthermore, a separate character has also been added to simply denote the presence and absence of manual unguals (to distinguish the condition of derived avialans in which unguals are absent, which was originally one of several unordered character states in Turner et al.’s character). As constructed, this current character on manual ungual curvature subsumes c haracter 255 of Brusatte et al. (2010a). We here score *Dryptosaurus* for state 0 (strongly curved manual unguals) based on the recent redescription of this taxon by Brusatte et al. (2011c), contra the “weak curvature” score in Brusatte et al. (2010a). Many ornithomimosaurs have a straight manual ungual 3 but more curved manual unguals 1 and 2. These taxa are here scored for state 1, whereas those ornithomimosaurs with straight manual digits 1-3 are scored as state 2. We do not differentiate state 1 into multiple states for “weak curvature in all unguals” vs. “weak curvature in unguals 1 and 2 and a straight ungual 3,” because derived tyrannosauroids do not possess a manual ungual 3, which would make it impossible to score them for this character as currently constructed. Therefore, we conservatively lump together these two conditions into state 1, but recognize that future authors may advocate subdividing this character (but this would necessitate scoring derived tyrannosaurids as inapplicable, or dividing this character into multiple characters).

**Character 149: Manual unguals, size of ungual on digit I compared to the other manual unguals in the hand:**

0: generally similar in size

1: distinctly larger

**Character 150: Manual unguals, a transverse ridge immediately dorsal to the articulating surface (“proximodorsal lip”):**

0: absent

1: present

**Character 151: Ilium, preacetabular process, anteroventral corner, form: (UNORDERED)**

0: subtriangular, ventral margin of preacetabular process is shallowly concave

1: subquadrate with recurved anterior margin, ventral margin of preacetabular process is deeply concave such that the open region between the pubic peduncle and anteroventral region of the preacetabular process defines much of a circle

2: process strongly hooked

Note: This character subsumes Brusatte et al. (2010a, character 259). State 1 is seen in some derived tyrannosauroids and ornithomimosaurs (and a few other coelurosaurs), and specifically refers to an anteroventral corner of the preacetabular process that is so recurved and ventrally projecting that the notch between it and the pubic peduncle is wide and essentially circular. Turner et al. (2012) and previous TWiG matrices scored the ougroups (*Allosaurus* and *Sinraptor*) as possessing state 1. However, although these taxa do have somewhat of a hooked anterior margin of the preactabular process (as do some compsognathids such as *Sinocalliopteryx*), they do not have the extremely pronounced condition of derived tyrannosauroids.

**Character 152: Ilium, preacetabular process, length: (ORDERED)**

0: roughly as long as postacetabular process

1: markedly longer (more than 2/3 of total ilium length) than postacetabular process

2: postacetabular blade much longer than postacetabular process

**Character 153: Ilium, morphology of anterior margin of preacetabular process: (UNORDERED)**

0: gently rounded or straight

1: anterior end strongly convex, lobate

2: pointed at anterodorsal corner with concave anteroventral edge

3: distinctly concave dorsally

Note: Turner et al. (2012) scored *Dilong* for state 0, which is unexpected if it is a tyrannosauroid (as all known tyrannosauroids possess state 3). However, based on personal observation of the specimen (IVPP V14243) the anterodorsal margin of the ilium is broken, so we here score this taxon as “?”. *Sinocallipteryx* is described as possessing a “slightly concave” anterodorsal margin of the preacetabular process, and this is clearly shown in an accompanying figure (Ji et al. 2007). Although we would prefer to confirm this score based on the original specimen, in order to determine whether it is genuine or a potential artifact of breakage, we here score *Sinocallipteryx* for state 3. Li et al. (2010) noted that *Mirischia* may possess an anterodorsal concavity, but personal observation of the specimen (SMNK 2349 PAL) shows that this region is broken, and therefore we score this taxon as “?”.

**Character 154: Ilium, supraacetabular crest on ilium, form: (ORDERED)**

0: present as a separate process from the antitrochanter, and forming a “hood” over the femoral head

1: reduced but still separate from the antitrochanter, not forming “hood”

2: absent

**Character 155: Ilium, postacetabular process, shape of distal end: (UNORDERED)**

0: squared

1: acuminate

2: squared, with nearly vertical posterior margin that is nearly equivalent in depth to the anterior margin of the preacetabular process

Note: We have expanded this character into an unordered multistate, by adding a third state referring to the extremely deep and squared-off postacetabular processes of some derived tyrannosauroids such as *Alioramus* and *Tyrannosaurus*, as noted by Brusatte et al. (2010a, character 267). This condition differs from the shallower postacetabular processes of more basal tyrannosauroids such as *Guanlong*, *Dilong*, and *Juratyrant*. In these taxa the postacetabular process is squared off posteriorly, but is much shallower than the preacetabular process.

**Character 156: Ilium, opposing postacetabular blades, orientation in dorsal view:**

0: subparallel to each other

1: diverge posteriorly from each other

Note: The “subparallel” condition includes those iliac blades which are oriented medially to contact each other (or nearly contact) above the sacrum (these blades are parallel to each other, but instead of their lateral surfaces being oriented straight laterally they are instead oriented dorsolaterally, hence the convergence above the midline). In some of these taxa, such as the ornithomimosaurs *Gallimimus* and *Garudimimus*, the postacetabular blades diverge from each other when seen in dorsal view, but this is a result of the reorientation of the lateral surface to face dorsolaterally, due to the medial orientation of the two blades to contact each other (or nearly contact) above the acetabulum. Without this medial rotation the postacetabular processes would be parallel or nearly parallel to each other. State 1 refers to the condition in some maniraptorans in which the two iliac blades clearly diverge from each other posteriorly when seen in dorsal view.

**Character 157: Ilium, tuber along dorsal edge of ilium dorsal or slightly posterior to acetabulum:**

0: absent

1: present

**Character 158: Ilium, brevis fossa, form:**

0: shallowly inset into bone, brevis shelf projects strongly medially

1: deeply inset into bone, with both lateral lamina and medial brevis shelf curving ventrally to demarcate the deeply concave fossa

**Character 159: Ilium, antitrochanter, form:**

0: absent or poorly developed

1: prominent

Note: This character is equivalent to Brusatte et al. (2010a, character 261). In scoring this character, we hold that the large antitrochanters of derived tyrannosauroids, which are extensive flanges that are deeply inset from the remainder of the ischial peduncle, are primarily homologous to the prominent antitrochanters of most other coelurosaurs. Therefore, tyrannosauroids with flange-like antitrochanters and other coelurosaurs with prominent antitrochanters are both scored for state 1.

**Character 160: Ilium, ridge bounding cuppedicus fossa, extension:**

0: terminates anterior to acetabulum or curves ventrally onto anterior end of pubic peduncle

1: extends far posteriorly and to become confluent or almost confluent with acetabular rim

**Character 161: Ilium, cuppedicus fossa, form: (ORDERED)**

0: deeply inset into the anterior and lateral surfaces of the pubic peduncle, with a pronounced dorsal rim

1: reduced, fossa shallow or flat, with little or no overhanging dorsal rim

2: absent

**Character 162: Ischium, prominent median posterior process along posterior edge of bone:**

0: absent, posterior edge of bone straight

1: present

**Character 163: Ischium, morphology of shaft distal to the acetabular (obturator) region: (ORDERED)**

0: extremely rod-like, midshaft diameter 30-50% midshaft diameter of pubis

1: rod-like, midshaft diameter 60-100% midshaft diameter of pubis

2: wide, flat, and plate-like, midshaft diameter greater than midshaft diameter of pubis

Note: This is a modified character that subsumes character 280 in Brusatte et al. (2010a). In essence, we here separate the “rod-like” character into two characters: extremely rod-like (in which the ischial shaft is less than 50% of the width of the pubic shaft, seen in some derived tyrannosauroids) and somewhat rod-like (in which the ischial shaft and pubic shaft are approximately the same diameter, seen in many coelurosaurs). We have rescored *Ornitholestes* for state 1 (somewhat rod-like), from Turner et al.’s (2012) polymorphic score for both a rod-like and a plate-like shaft in this taxon. We also score the tyrannosauroids *Dryptosaurus* and *Alioramus* as polymorphic for states 0 and 1. This is a conservative score, because it is clear that the ischium of these taxa is rod-like but it is unclear how the midshaft diameter compares to the pubic midshaft diameter.

**Character 164: Ischium, orientation of shaft: (UNORDERED)**

0: straight

1: curved anteriorly at the ventral end

2: hooked posteriorly at the ventral end

**Character 165: Ischium, surface morphology of lateral face of ischiadic blade: (UNORDERED)**

0: flat or gently rounded

1: concave

2: with longitudinal ridge subdividing lateral surface into anterior (including obturator process) and posterior parts

**Character 166: Ischium, obturator process of ischium: (ORDERED)**

0: absent

1: proximal in position (midpoint of process positioned at less than 30% of the total length of the ischium)

2: located near middle of ischiadic shaft (midpoint of process positioned at approximately 40-50% of the total length of the ischium)

3: located at distal end of ischium (midpoint of process positioned distal to the midpoint of the ischium)

Note: The language in this character has been modified following the quantitative character description of Brusatte et al. (2010a, character 281).

**Character 167: Ischium, obturator process, contact with pubis:**

0: absent

1: present

**Character 168: Ischium, obturator foramen or notch in proximal portion of obturator process: (ORDERED)**

0: enclosed foramen present

1: open notch present (i.e., a foramen that is no longer completely enclosed)

2: notch or foramen absent

Note: This character has been modified to take into account the closed obturator foramen of *Guanlong* and *Mirischia*, which although unusual among coelurosaurs and proximal outgroups (allosauroids) is also seen in more distal basal theropod outgroups. Although Naish et al. (2004) described the left and right obturator foramina in *Mirischia* as asymmetrical (one open as a notch, one enclosed), we interpret the supposedly open notch as a closed foramen that has been broken, based on personal observation of the specimen (SMNK 2349 PAL). Turner et al. (2012) scored derived tyrannosauroids for “notch or foramen absent,” whereas *Dilong*, compsognathids, and ornithomimosaurs were scored for “open notch present.” However, all tyrannosauroids have an open obturator notch that is essentially identical in morphology to those of ornithomimosaurs and compsognathids. Therefore, these taxa are here scored for state 1.

**Character 169: Ischium, ischial tubercle (“semicircular scar”) ventral to iliac peduncle on the posterior margin of the proximal end of ischium: (ORDERED)**

0: absent or potentially homologous structure present as a groove

1: present as a convex bulge on the posterior surface of the ischium

2: present as a rugose, ovoid or triangular flange whose lateral surface is depressed relative to the remainder of the ischium

Note: This character has been modified, and an additional state added, based on Brusatte et al. (2010a, character 278). We consider the condition in ornithomimosaurs to be equivalent to the “convex bulge” condition of the tyrannosauroid *Juratyrant*, and score more derived tyrannosauroids for a hierarchically nested condition of an especially rugose, flange-like tubercle. We also consider the “groove-like” morphology of this region of the ischium to be homologous, at some level, to the ischial tubercle itself (see Hutchinson 2001). Because it is difficult to distinguish the groove-like condition from true absence of any muscular attachment in this region, we lump together both conditions as state 0 but recognize that future work may be able to subdivide these conditions into separate character states.

**Character 170: Ischium, length of bone:**

0: greater than two-thirds of pubis length

1: two-thirds or less of pubis length

**Character 171: Ischium, morphology of distal ends of opposing ischia: (ORDERED)**

0: form symphysis

1: approach one another but do not form symphysis

2: widely separated

Note: We here score all tyrannosauroids for state 0, contra Turner et al. (2012) who score derived taxa for state 1. As discussed by Brusatte et al. (2012a), the distal ischia of tyrannosauroids do make contact with each other, although this contact is unfused. This is equivalent to the condition in ornithomimosaurs and compsognathids, and we here score all of these taxa for state 0.

**Character 172: Ischium, distal end, expansion relative to midshaft:**

0: present, resulting in an ischial “boot”

1: absent, ischial “boot” absent

Note: This character is equivalent to Brusatte et al. (2010a, character 279).

**Character 173: Ischium, tubercle on anterior edge of bone:**

0: absent

1: present

**Character 174: Pubis, orientation of shaft when in articulation: (UNORDERED)**

0: projecting anteroventrally (propubic)

1: vertical

2: projecting posteroventrally (opisthopubic)

3: appressed to ischium

Note: This character subsumes Brusatte et al. (2010a, character 275). We consider this character unordered because there is not a clear transformation sequence or nested set of homologies, although it is possible that future authors may argue for an anteroventral-vertical-posteroventral trend in theropod pelvic evolution.

**Character 175: Pubis, pubic boot, anterior projection of boot relative to posterior projection: (ORDERED)**

0: equal in size or anterior process larger than posterior process

1: anterior process small, approximately 10-40%

2: anterior process completely absent, posterior process large

3: both anterior and posterior processes absent (i.e., boot present but without distinct anterior and posterior projections)

Note: This character subsumes Brusatte et al. (2010a, character 273). We have added an additional state here, to distinguish between taxa in which the anterior and posterior projections are equal in size (some derived tyrannosaurids) and those taxa in which anterior projections are present but are approximately 10-40% of the length of the posterior projection (many other coelurosaurs). Using our character scoring scheme, compsognathids and various paravians are scored for state 2, which refers to the complete lack of an anterior process and a pubic boot that is often “hook-like” in its morphology.

**Character 176: Pubis, shelf on shaft proximal to symphysis (‘pubic apron’): (UNORDERED)**

0: extends medially from middle of shaft

1: shelf extends medially from anterior edge of shaft

2: strongly reduced (restricted to distal end of pubis) or absent

Note: Turner et al. (2012) scored tyrannosauroids (including *Dilong* and tyrannosaurids) for state 0, but we here rescore all score-able tyrannosauroids for state 1, based on personal observation of specimens. In all known tyrannosauroid pubes the pubic apron arises from the anterior margin of the shaft, not the middle of the shaft as in outgroups like *Allosaurus* (Madsen 1976). We have also removed the language in the original TWiG character relating to the cylindrical or anteroposteriorly flattened nature of the shafts, as these conditions do not correspond 1-to-1 with the location of the apron. Future authors may wish to reintroduce a character relating to the morphology of the shaft (cylindrical vs. flattened), but we could not identify clear, distinct categories of variation among taxa and hence do not utilize such a character here.

**Character 177: Pubis, curvature of shaft: (UNORDERED)**

0: absent, shaft straight

1: distal end curves anteriorly, anterior surface of shaft concave

2: distal end curves posteriorly, anterior surface of shaft convex

Note: This character subsumes character 269 of Brusatte et al. (2010a). Derived tyrannosauroids are here scored for state 1, a concave anterior margin of the pubic shaft, based on Brusatte et al. (2010a).

**Character 178: Pubis, length of pubic apron: (ORDERED)**

0: about half of pubic shaft length

1: less than 1/3 of shaft length

2: greatly reduced, restricted to far distal end of pubis

3: absent

Note: We modified this character to make it into an additive multistate referring to the presence/absence and size of the pubic apron. For all other pubic apron characters, those taxa without an apron are scored as inapplicable (“?”).

**Character 179: Pubic apron, form of contact between opposing pubes distally: (ORDERED)**

0: both pubes meet extensively

1: contact between pubes disrupted by a slit (pubic foramen)

2: no contact between pubes distally, pubic apron absent in this part of pubis but present further proximally

Note: The pubic fenestra is usually considered a synapomorphy of Tetanurae (Rauhut 2003; Benson et al. 2009). Turner et al. (2012) scored *Dilong* for state 0, but personal observation of the specimen indicates that the distal portion of the conjoined pubes is too damaged (and not complete enough) to permit observation of the fenestra. Therefore, it is here scored “?”. Turner et al. (2012) also scored *Haplocheirus* for state 0, which would be unexpected, but because we cannot confirm this with a personal observation we conservatively score it as “?”.

**Character 180: Femur, head, fovea capitalis for attachment of capital ligament:**

0: absent or subtle

1: present as a distinct circular fovea located in center of the medial surface of the head

**Character 181: Femur, interaction of lesser and greater trochanters: (ORDERED)**

0: separated from each other by deep cleft

1: separated from each other by small groove

2: completely fused to each other (absent as distinct structures), forming a trochanteric crest

**Character 182: Femur, lesser trochanter, shape in lateral view:**

0: alariform, projects anteriorly as a broad flange that is anteroposteriorly wider than the greater trochanter

1: reduced to approximately the same anteroposterior width as the greater trochanter, cylindrical in cross section

**Character 183: Femur, ridge on lateral surface distal to lesser and greater trochanters (homologous to the trochanteric shelf):**

0: absent or represented only by faint rugosity or bulge

1: distinctly raised from shaft as a pronounced, mound-like ridge

**Character 184: Femur, fourth trochanter:**

0: present

1: absent (or reduced to a subtle and barely distinguishable margin)

**Character 185: Femur, accessory trochanteric crest distal to lesser trochanter:**

0: absent

1: present

Note: We consider the “accessory trochanteric crest” (identified by Makovicky and Sues [1998] in *Microvenator*) as equivalent to the accessory trochanter that is present in some tetanurans (including some coelurosaurs: e.g., Brusatte et al. 2008c), as both structures are accessory flanges or ridges along the anterior margin of the distl portion of the lesser trochanter. Therefore, this character is equivalent to Brusatte et al. (2010a, character 287). Accessory trochanters are large and prominent in basal tyrannosauroids (*Guanlong*, *Xiongguanlong*) but are lost in more derived tyrannosauroids. They are also large and prominent in several basal coelurosaurs (e.g., ornithomimosaurs, *Mirischia*, *Zuolong*, *Coelurus*, *Tanycolagreus*).

**Character 186: Femur, mesiodistal crest, form:**

0: absent or present as a subtle structure

1: present as a pronounced longitudinal crest extending proximally from medial condyle, which is seen to strongly overhang the remainder of the medial margin of the femur in posterior view (i.e., is visible as a broad flange in posterior view

Note: We have reworded this character to make it refer specifically to the pronounced mesiodistal crest of some coelurosaurs. This crest is either located on the anterior surface of the femur (as described in the original TWiG character) or along the anteromedial corner of the femur, but in all cases extends proximally from the medial condyle.

**Character 187: Femur, flexor groove, morphology of distal end:**

0: open distally, smoothly confluent with distal articular surface

1: closed off distally by contact between distal condyles, separated from distal articular surface

**Character 188: Fibula, distal extent:**

0: reaches proximal tarsals

1: short, tapering distally, and not in contact with proximal tarsals

**Character 189: Fibula, medial margin of proximal end in proximal view:**

0: concave

1: flat

**Character 190: Fibula, deep oval fossa, with well defined margins, on medial surface near proximal end:**

0: absent

1: present

Note: This character refers specifically to the discrete, ovoid fossa of some tyrannosauroids and ornithomimosaurs, as well as outgroup taxa. Some other basal coelurosaurs such as *Coelurus* and *Zuolong* have a depressed groove excavating much of the medial surface of the proximal fibula, but this is not present as a discrete, indented fossa with well defined margins (instead, the groove has a well defined anterior margin but opens posteriorly). The outgroups are scored for state 0, because they possess an open fossa (without a distinct posterior margin) as in most coelurosaurs.

**Character 191: Astragalus and calcaneum, morphology of distal condyles:**

0: condyles separated by shallow, indefinite sulcus

1: condyles separated by prominent tendoneal groove on anterior surface

**Character 192: Tibia, number of cnemial crests:**

0: single pronounced crest

1: two crests (main crest with accessory anterior crest)

**Character 193: Astragalus, ascending process, form: (UNORDERED)**

0: tall and broad, covering most of anterior surface of distal end of tibia (70% or more of mediolateral width of surface) and dorsoventral height greater than twice the height of the main body of the astragalus

1: process short and slender, covering only lateral half of anterior surface of tibia (includes derived therizinosauroid condition in which a lateral extension of the ascending process contacts the fibula) and dorsoventral height less than twice the height of the main body of the astragalus

2: ascending process tall, but with medial notch that restricts it to lateral side of anterior face of distal tibia

Note: This character includes information on both the dorsoventral height and mediolateral width of the ascending process. Sometimes these measurements are given separate characters, but we here hold that they are generally correlated, in the sense that taxa with a very tall ascending process always have a mediolaterally broad process as well, and taxa such as *Guanlong* and *Falcarius* with a short ascending process also have a mediolaterally restricted process. We recognize, however, that future authors may want to break this character into separate characters referring to length and width, but we hesitate to do so because we do not wish to introduce a second character that is likely correlated with the first.

The ascending processes of compsognathids have been described as somewhat reduced, in that they only cover approximately 70% of the anterior surface of the tibia (Ostrom 1978; Currie and Chen 2001; Hwang et al. 2004). We consider this condition equivalent to state 0 here, but recognize that future authors may want to subdivide state 0 into separate characters for a broad ascending process that covers most of the anterior surface of the tibia and a reduced process that covers approximately 70% of the surface. These could be associated with state 1 here (a process covering half the tibial surface) to create an ordered multistate, and state 2 here (which is unique to some alvarezsaurids) could be made into its own character. We do not do this, however, because we hesitate to divide state 0 into additional states because the “reduced” ascending processes of compsognathids are all based on slab specimens, which are difficult to measure and often prone to misinterpretation. It is clear, however, that these compsognathids do not possess the reduced and laterally restricted ascending processes of some basal coelurosaurs (e.g., *Guanlong*, *Coelurus*, *Tugulusaurus*) and the ougroups (*Allosaurus*, *Sinraptor*), which are denoted by state 1. This character is an equivalent, but expanded, version of Brusatte et al. (2010a, character 297).

**Character 194: Astragalus, ascending process, articulation with condyles distally:**

0: confluent with condylar region of astragalus

1: separated from condylar region by transverse groove or fossa across base

**Character 195: Astragalus and calcaneum, fusion: (ORDERED)**

0: absent, astragalus and calcaneum unfused to each other or to tibia in adults

1: present, astragalus and calcaneum fused to each other, unfused to tibia

2: present, astragalus and calcaneum completely fused to each other and to tibia

**Character 196: Distal tarsals, fusion with metatarsals:**

0: absent, distal tarsals and metatarsals separate

1: present, distal tarsals and metatarsals fused into tarsometatarsus

**Character 197: Metatarsals, fusion: (ORDERED)**

0: absent, metatarsals not co-ossified

1: present, metatarsals co-ossified proximally

2: present, metatarsals co-ossified proximally and distally

3: present, metatarsals co-ossified proximally and distally, with distal fusion extreme and distal vascular foramen closed

**Character 198: Metatarsal II, morphology of distal end:**

0: smooth, not ginglymoid

1: with developed ginglymus that extends onto extensor surface, giving the distal end a strongly concave profile in extensor (anterior) view

**Character 199: Metatarsal III, morphology of distal end:**

0: smooth, not ginglymoid

1: with developed ginglymus: smooth articular region extends proximally onto extensor surface and is broadly exposed

Note: We here consider the condition in some basal coelurosaurs (*Zuolong*, *Tugulusaurus*, *Dilong*, *Guanlong*) to represent a well developed ginglymus, as defined for character state 1. Xu et al. (2004) recognized that *Dilong* was unusual among tyrannosauroids in possessing a gingylmus on metatarsal III (although it is absent on metatarsal II), a character score that was followed by Turner et al. (2012). Later discoveries showed that this character state is also present in some other basal coelurosaurs: Rauhut and Xu (2005) later noted that one was present on *Tugulusaurus* and Choiniere et al. (2010b) described and figured it for *Zuolong*. Our personal observations of *Guanlong* show that it is present in this taxon as well. *Bicentenaria* is described by Novas et al. (2012) as lacking a ginglymoid distal end of metatarsal III, but we score this taxon here as “?” because we have not been able to observe it, and therefore cannot assess if it has the basal coelurosaur-style ginglymoid condition of *Guanlong*, *Tugulusaurus*, and *Zuolong*.

**Character 200: Metatarsal III, exposure of proximal shaft in extensor view: (ORDERED)**

0: prominently exposed between MT II and MT IV along entire metapodium

1: MT III proximal shaft constricted and much narrower than either II or IV, but still exposed along most of metapodium, subarctometatarsal

2: very pinched, not exposed along proximal section of metapodium, arctometatarsal

3: proximal part of MT III lost entirely

Note: We here consider this character ordered, because we hypothesize nested homology between the subarctometatarsal-arctometatarsal-mt III lost states (i.e., these form an ordered, progressive sequence of metatarsal III pinching and eventual loss). This character is equivalent to Brusatte et al. (2010a, Character 299).

**Character 201: Pedal digit II, ungual and penultimate phalanx, morphology:**

0: similar to those of III

1: penultimate phalanx highly modified for extreme hyper-extension, ungual more strongly curved and significantly larger than that of digit III

**Character 202: Metatarsal II, site of articular surface for metatarsal I: (UNORDERED)**

0: the middle of the medial surface of metatarsal II

1: the posterior surface of distal quarter

2: the medial surface near the proximal end

3: the medial surface at or near the distal end

**Character 203: Metatarsal I, form of proximal end:**

0: attenuates proximally, articular surface for metatarsal II is a simple butt joint lying against the shaft of metatarsal II

1: large and robust, similar to those of metatarsals II-IV

Note: Although character states could probably be inferred by the form and position of the metatarsal I articular scar on metatarsal II, we conservatively score taxa for an affirmative score only if we can observe metatarsal I itself.

**Character 204: Metatarsal IV, shaft thickness:**

0: round or thicker dorsoventrally (extensor-flexor direction) than wide mediolaterally in cross section

1: mediolaterally widened and flat in cross section

**Character 205: Foot, symmetry:**

0: symmetrical

1: asymmetrical with slender MTII and very robust MT IV, excluding flange

**Character 206: Dorsal vertebrae, neural spines on posterior dorsals, shape in lateral view:**

0: rectangular or square

1: fan-shaped, anteroposterior length of spine expanding dorsally

Note: State 1 refers specifically to the fan-shaped neural spines of compsognathids and *Pelecanimimus*, whose dorsal extremity is substantially longer anteroposteriorly than is the base of the spine. This is due to a “funneling outwards) in anteroposterior length dorsally across the length of the spine. Some theropods (e.g., *Tanycolagreus*, some ornithomimosaurs in intermediate phylogenetic position between *Pelecanimimus* and derived taxa with rectangular neural spines) have some dorsal vertebrae with neural spines that are moderately expanded at their dorsal tips, but these do not exhibit the striking fan shape of compsognathids, and are thus scored for state 0.

**Character 207: Manual phalanx I-1, shaft diameter:**

0: less than or approximately equal to the shaft diameter of radius.

1: greater than the shaft diameter of radius.

Note: State 1 of this character refers to a phalanx that is substantially thicker in diameter than the radius. Several coelurosaurs (e.g., *Tanycolagreus*, tyrannosauroids, ornithomimosaurs) have a phalanx I-1 that is approximately the same diameter of the radius, and sometimes is slightly larger (~10%). These taxa are scored for state 0, however, because this condition differs from the condition in compsognathids in which the phalanx is dramatically larger than the radius (state 1).

**Character 208: Angular, extent in lateral view:**

0: widely exposed, suture between surangular and angular reaches or nearly reaches posterior end of mandible

1: reduced in exposure, suture between surangular and angular does not reach posterior end of the mandible

**Character 209: Surangular, laterally inclined flange along dorsal edge of bone for articulation with the lateral process of lateral quadrate condyle:**

0: absent

1: present

**Character 210: Distal articular ends of metacarpals I + II: (UNORDERED)**

0: ginglymoid

1: rounded, smooth

2: II ginglymoid and MC I shelf

**Character 211: Radius and ulna, articulation with each other:**

0: well separated

1: with distinct adherence or syndesmosis distally

**Character 212: Upper and lower jaws, occlusion:**

0: occlude for their full length

1: diverge anteriorly due to kink and downward deflection of the dentary (pronounced downward deflection of dentary buccal margin anteriorly)

**Character 213: Quadrate, exposure of head in lateral view:**

0: absent, covered by squamosal laterally

1: present, exposed because quadrate cotyle of squamosal opens laterally due to a wide notch between the ventral and posterior processes

**Character 214: Ilium, brevis fossa, orientation and exposure in lateral view:**

0: faces primarily ventrally, but it is widely visible in lateral view (especially anteriorly)

1: faces primarily ventrally and medially, and it is obscured in lateral view across its entire length by a well developed lateral lamina of the postacetabular process of the ilium

Note: This character is equivalent to character 310 in the revised version of the Brusatte et al. (2010a) analysis published by Brusatte and Benson (2013). We have altered the wording here to reflect the character language of Brusatte and Benson (2013), which focuses specifically on whether the fossa is visible or obscured in lateral view.

**Character 215: Lesser trochanter, vertical ridge on lateral surface:**

0: present

1: absent

**Character 216: Supratemporal fossa, extension onto the squamosal:**

0: absent, fenestra bounded laterally and posteriorly by the squamosal, with no distinct fossa present on the dorsal surface of the squamosal

1: present, distinct fossa extends onto the dorsal surface of the squamosal

**Character 217: Dentary, teeth: (ORDERED)**

0: present across much of bone, dentary fully toothed

1: present but only at the far anterior tip

2: absent, dentary edentulous

**Character 218: Coracoid, ventral (=anterior) margin of bone anterior (=ventral) to glenoid, form:**

0: straight or shallowly indented by a notch

1: deeply indented by a pronounced notch separating the glenoid and postglenoid process, glenoid lip everted

Note: State 1 refers to the deep notch of ornithomimosaurs, which separates the glenoid from a pronounced and hook-shaped postglenoid process anteriorly. Many other coelurosaurs have a shallow notch in this region, but only ornithomimosaurs have a discrete, deep notch, which helps to define the characteristic hook-like postglenoid process in these taxa.

**Character 219: Articular, retroarticular process, orientation:**

0: points posteriorly

1: curves gently posterodorsally

**Character 220: Scapula, flange on supraglenoid buttress:**

0: absent

1: present

**Character 221: Laterosphenoid, depression (possibly pneumatic) on ventral surface of postorbital process of bone:**

0: absent

1: present

**Character 222: Braincase, basal tubera, mediolateral width of conjoined tubera relative to occipital condyle: (ORDERED)**

0: as wide as or wider than the occipital condyle

1: narrower than occipital condyle, reduced to a set of small processes directly below the condyle and separated by a narrow notch

2: tubera absent

Note: This character is not equivalent to characters 154 and 155 in Brusatte et al. (2010a), which refer to the dorsoventral depth of the basal tubera and the depth of the notch between the tubera, respectively. This character refers solely to the mediolateral width of the tubera, and is considered an ordered character because we hypothesize a nested set of homologies (large-small-absent). The highly modified basal tubera of derived therizinosauroids are here regarded as possessing state 0 and not state 2 (absent), as remnants of the tubera are still present despite their incorporation into the basisphenoidal bulla.

**Character 223: Ilium, postacetabular process, morphology of dorsal edge:**

0: convex or straight

1: deeply concave, ventral portion of postacetabular process (housing brevis shelf medially) extending posterior to dorsal portion of the postacetabular process as a tab-like structure

Note: State 1 refers to the condition in some dromaeosaurids (e.g., *Unenlagia*), in which the dorsal margin of the postacetabular process is deeply concave, due to the extension of the ventral portion of the postacetabular process posteriorly as a discrete, rectangular, tab-like structure. This tab-like structure is ornamented by the brevis shelf medially, and therefore houses an extension of the brevis fossa itself. Some ornithomimosaurs have a subtly concave dorsal border of the postacetabular process, but this is not considered equivalent to the marked condition of some dromaeosaurids.

**Character 224: Ilium, posterior end of postacetabular process, morphology in dorsal view:**

0: terminating in rounded or square end, due to a brevis shelf that extends no farther than the posterior margin of the lateral lamina (lateral surface) of the postacetabular process

1: with lobate brevis shelf projecting beyond posterior end of lateral lamina of postacetabular process

**Character 225: Pedal phalanx II-2, flexor heel, form:**

0: small and asymmetrically developed only on medial side of vertical ridge subdividing proximal articulation

1: heel long and lobate, with extension of midline ridge extending onto its dorsal surface

Note: Taxa without a discrete flexor heel are scored as inapplicable (“?”).

**Character 226: Metatarsal IV, large, longitudinal flange along posterior or lateral surface of bone:**

0: absent

1: present

**Character 227: Ischium, proximodorsal process, form:**

0: small, tab-like or pointed process along posterior edge of ischium

1: large, proximodorsally hooked, and separated from iliac peduncle of the ischium by a notch

Note: Taxa without a discrete proximodorsal process are scored as inapplicable (“?”).

**Character 228: Pubis, prominent tubercle on lateral surface of shaft, at approximately the midpoint of the shaft:**

0: absent, shaft smooth

1: present

**Character 229: Ischium, distally placed dorsal process along posterior edge of shaft:**

0: absent

1: present

**Character 230: Ischium, obturator process, shape:**

0: tab-like, with distal end separated from shaft by a discrete notch

1: triangular, with distal end confluent with shaft

**Character 231: Ischium, morphology of triangular obturator process (only in those taxa with a triangular process as defined in character 233):**

0: longer proximodistally than wide anteroposteriorly at the center of the process

1: shorter proximodistally than wide anteroposteriorly at the center of the process, resembles an elongate triangle extending anteriorly

**Character 232: Metatarsal II, tuber along extensor surface (associated with the insertion of the tendon of the m. tibialis cranialis in Aves): (ORDERED)**

0: absent

1: present, on approximately the center of the proximodorsal surface of metatarsal II

2: present, developed on lateral surface of metatarsal II, at contact with metatarsal III or on lateral edge of metatarsal III

**Character 233: Ulna: femur length ratio:**

0: substantially less than one

1: equal to or greater than one

**Character 234: Maxilla, position of maxillary fenestra relative to ventral margin of antorbital fossa: (ORDERED)**

0: abuts ventral margin

1: dorsal to ventral margin, at approximately the dorsoventral midpoint of the antorbital fossa

2: far dorsal to the ventral margin, dorsal to the dorsoventral midpoint of the antorbital fossa

Note: This character has been modified to take into account variation in the position of the maxillary fenestra in tyrannosauroids, as noted by Brusatte et al. (2010a, character 18). State 0 here refers to the condition in *Daspletosaurus*, *Tarbosaurus*, and *Tyrannosaurus*, in which the maxillary fenestra abuts the ventral margin of the antorbital fossa.

**Character 235: Maxilla, dorsoventral depth of main body of bone (=jugal process) at the midpoint of the antorbital fenestra compared to the depth of the entire skull at this measuring point: (ORDERED)**

0: shallow, less than 16% of the depth of the entire skull

1: between 16-22% of skull depth

2: deep, greater than 22% of skull depth

Note: This character has been modified into an ordered multistate to take into account additional variation noted by Brusatte et al. (2010a, character 23).

**Character 236: Maxilla, maxillary fenestra, recessed within a shallow, posteriorly or posterodorsally open fossa, which is itself located within the maxillary antorbital fossa:**

0: absent

1: present

**Character 237: Maxilla, ascending process (=dorsal ramus or nasal process), form:**

0: prominent, exposed medially and laterally

1: absent or reduced to a process with slight medial and no lateral exposure

**Character 238: Maxilla, participation of the ventral ramus of the nasal process (=ascending process) in the anterior margin of the antorbital fenestra (as seen in lateral view): (ORDERED)**

0: present extensively

1: small dorsal projection of the maxilla participates in the anterior margin

2: no dorsal projection of maxilla participates in the anterior margin

**Character 239: Antorbital fenestra, composition of the dorsal border (as seen in lateral view):**

0: lacrimal and maxilla

1: lacrimal and nasal

**Character 240: Antorbital fossa, composition of the dorsal border (as seen in lateral view): (UNORDERED)**

0: lacrimal and maxilla

1: lacrimal and nasal

2: maxilla, premaxilla, and lacrimal

**Character 241: Maxilla, lateral lamina of the ascending ramus:**

0: present and broadly exposed in lateral view

1: present but reduced to small triangular exposure in lateral view

**Character 242: Frontal, supratemporal fossa, anteroposterior length compared to overall length of exposed portion of frontal on skull roof: (ORDERED)**

0: less than 30%

1: between 30-60%

2: greater than 60%

Note: This character has been modified and transformed into an ordered multistate using the quantitative character descriptions of Brusatte et al. (2010a, character 115). As worded here, this character now refers solely to the extent of the supratemporal fossa onto the frontal, unlike the previous TWiG character that referred to the qualitative extent (minor, extensive) of the fossa onto the frontal and postorbital. We note, however, that taxa with an extensive fossa on the frontal usually also have an extensive fossa on the dorsal surface of the postorbital.

**Character 243: Jugal, contribution to antorbital fenestra margin (in lateral view):**

0: absent

1: present

**Character 244: Maxillary and dentary teeth, size of mesial (=anterior) and distal (=posterior) denticles:**

0: not substantially different in size

1: mesial denticles, when present, substantially smaller than distal denticles

**Character 245: Maxillary teeth, orientation relative to the long axis of the lower jaw:**

0: almost perpendicular to jaw margin

1: inclined strongly posteroventrally

**Character 246: Maxillary teeth, apicobasal height of teeth along tooth row:**

0: highly variable with gaps evident for replacement

1: almost isodont with no replacement gaps

**Character 247: Splenial, posterior margin (anterior margin of internal mandibular fenestra):**

0: smooth

1: with distinct notch

**Character 248: Premaxillary teeth, cross sectional size of first tooth crown compared with crowns of premaxillary teeth 2 and 3: (UNORDERED)**

0: slightly smaller or same size

1: much smaller

2: much larger

**Character 249: Maxilla, promaxillary fenestra in adults, visibility in lateral view:**

0: visible (either as complete fenestra or portion of the fenestra)

1: completely obscured by ascending ramus of maxilla

Note: This character is equivalent to character 15 in Brusatte et al. (2010).

**Character 250: Nasal, shape of dorsal surface: (ORDERED)**

0: flat or slightly convex

1: convex (vaulted) anteriorly, above and immediately posterior to the external naris

2: vaulted across most of their length

Note: This character has been modified following Brusatte et al. (2010, character 38). Tyrannosauroids with a midline nasal crest are scored as inapplicable (“?”).

**Character 251: Nasal, fusion between left and right nasals:**

0: absent

1: present

**Character 252: Quadratojugal and squamosal, constriction of lateral temporal fenestra: (ORDERED)**

0: absent, anterior margins of both bones are approximately vertical

1: present, convex kink along the suture between the two bones that projects into the fenestra, constricting it to approximately one half or less of its maximum anteroposterior length

2: present, dorsal region of quadratojugal moderately expanded anteroposteriorly relative to the remainder of the bone, constricting fenestra to approximately one half of its maximum anteroposterior length

3: present, dorsal region of quadratojugal expanded anteroposteriorly by at least twice the minimum anteroposterior dimension of the bone, forming a flange that meets the ventral ramus of the squamosal to nearly divide the fenestra

Note: This character is expanded into an ordered multistate using the character state definitions of Brusatte et al. (2010a, character 98).

**Character 253: Supraoccipital, pronounced and strongly demarcated median ridge on posterior (occipital) surface:**

0: absent

1: present

**Character 254: Surangular, anteroventral extension divides external mandibular fenestra by contacting angular anteriorly**

0: absent

1: present

**Character 255: Ilium, vertical ridge on lateral surface of iliac blade above acetabulum: (ORDERED)**

0: absent or poorly developed

1: present as a well-developed, linear structure extending vertically or anterodorsally

2: present as a well-developed, linear structure extending posterodorsally

Note: This character is modified into an ordered multistate to take into account variation within tyrannosauroids as noted by Brusatte et al. (2010a, character 258).

**Character 256: Premaxilla, main body, dorsoventral depth compared to anteroposterior length: (ORDERED)**

0: less than or equal to

1: between 1.0-1.9 times larger

2: greater than 2 times larger

Note: This character is modified into an ordered multistate to take into account variation within tyrannosauroids as noted by Brusatte et al. (2010a, character 8).

**Character 257: Nasal, external texture of mid section of bone:**

0: smooth to slightly rugose

1: pronounced rugosities and accessory vascular foramina present

Note: This character is equivalent to Brusatte et al. (2010a, character 40).

**Character 258: Jugal, shape of anterior process underneath the lacrimal: (UNORDERED)**

0: tapering

1: bluntly squared anteriorly

2: expanded

3: bifurcated

**Character 259: Axis, neural spine, morphology in lateral view:**

0: extensive and anteroposteriorly elongate, sheet-like

1: anteroposteriorly reduced, rod-like

**Character 260: Cervical vertebrae, prezygapophyses in anterior postaxial cervicals, orientation:**

0: straight across their lengths

1: anteroposteriorly convex, flexed ventrally anteriorly

**Character 261: Dorsal vertebrae, extent of pneumaticity (pneumatic foramina on lateral surfaces): (ORDERED)**

0: foramina absent

1: foramina present in anterior dorsals

2: present in all dorsals

Note: This character is equivalent to Brusatte et al. (2010, character 218).

**Character 262: Femur/humerus length ratio: (ORDERED)**

0: more than 3.3

1: between 3.3-2.5

2: between 1.2 and 2.5

3: less than 1

Note: This character has been modified to include additional variation within tyrannosauroids, following Brusatte et al. (2010a, character 239).

**Character 263: Humerus, shape in lateral view:**

0: sigmoidal

1: straight

Note: We consider this character to be equivalent to character 242 in Brusatte et al. (2010a), which scores for the presence/absence of humeral shaft rotation (quantified by the angle between the long axes of the proximal and distal ends). Brusatte et al. (2010a) scored *Dryptosaurus* for such rotation, but this taxon has a straight humerus in lateral view and is thus scored for state 1 here. It is possible that there is a subtle amount of variation within this character, such that taxa like *Dryptosaurus* have a straight humerus in lateral view but still retain some degree of rotation (a 30-45 degree angle between the long axes of the proximal and distal ends). However, we do not want to subdivide this character too finely, because it makes recognizing homologies across a wide swath of taxa more difficult. Therefore, we retain a simple binary character here.

**Character 264: Radius, length compared to that of humerus:**

0: more than half the length of humerus

1: less than half the length of humerus

**Character 265: Premaxilla, fusion of opposing premaxillae at the symphysis: (ORDERED)**

0: unfused in adults

1: fused anteriorly in adults, posterior nasal [frontal] processes not fused to each other

2: fused anteriorly and opposing frontal processes completely fused

**Character 266: Dentary, form of articulation between opposing dentaries at the symphysis:**

0: joined proximally by ligaments

1: joined by bone

**Character 267: Dentary, symphysis region, two strong grooves forming an anteriorly opening ‘v’ in ventral view:**

0: absent

1: present

**Character 268: Cranium, facial margin, composition: (ORDERED)**

0: primarily formed by the maxilla, with the maxillary process of the premaxilla restricted to the anterior tip

1: maxillary process of the premaxilla extending along half of facial margin

2: maxillary process of the premaxilla extending more than half of facial margin

**Character 269: Premaxilla, nasal (frontal) process, length:**

0: short

1: long, closely approaching frontal

**Character 270: External naris, size compared to the antorbital fenestra: (ORDERED)**

0: considerably smaller

1: long axis approximately same length as long axis of antorbital fenestra

2: larger

Note: This character has been expanded into an ordered multistate to take into account variation within tyrannosauroids noted by Brusatte et al. (2010a, character 4). *Sinotyrannus* is here scored for state 1; although the antorbital fenestra is not preserved in this taxon, state 1 is hypothesized because the external naris long axis is as long as the first seven teeth of the maxillary tooth row, similar to the proportions in *Guanlong* and *Proceratosaurus*, which are scored for state 1, but differing from the state in other tyrannosauroids (and most other coelurosaurs) in which the naris is no longer than the first 4-5 teeth.

**Character 271: Ectopterygoid:**

0: present

1: absent

**Character 272: Vomer and pterygoid, form of articulation between bones: (UNORDERED)**

0: present, well developed

1: reduced, narrow process of pterygoid passes dorsally over palatine to contact vomer

2: absent, pterygoid and vomer do not contact

Note: This character is left unordered because it is unclear if states 1 and 2 form a nested set of homologies.

**Character 273: Palatine and pterygoid, form of articulation between bones:**

0: long, anteroposteriorly overlapping contact

1: short, primarily dorsoventral contact

**Character 274: Palatine, contacts with bones of the facial region:**

0: contacts maxillae only

1: contacts premaxillae and maxillae

**Character 275: Vomer, contact with premaxilla:**

0: present

1: absent

**Character 276: Basisphenoid, projecting articulation with pterygoid via discrete basipterygoid processes:**

0: present

1: absent

**Character 277: Basisphenoid, location of pterygoid articular surface:**

0: located basal on basisphenoid

1: located markedly anterior on basisphenoid (parasphenoid rostrum) such that the articulations are subadjacent on the narrow rostrum

**Character 278: Basisphenoid and pterygoid articulation, orientation of contact: (UNORDERED)**

0: anteroventral

1: mediolateral

2: entirely dorsoventral

**Character 279: Pterygoid, articular surface for basisphenoid, morphology: (ORDERED)**

0: concave ‘socket’ or short groove enclosed by dorsal and ventral flanges

1: flat to convex

2: flat to convex facet, stalked, variably projected

**Character 280: Pterygoid, shape of bone:**

0: kinked, surface for basisphenoid articulation at high angle to axis of palatal process of pterygoid

1: straight, basisphenoid articulation in line with axis of palatal process

**Character 281: Ossified interorbital septum, sphenethmoid:**

0: absent

1: present

Note: This character is equivalent to Brusatte et al. (2010a, character 167), which recognizes the presence of an ossified mesethmoid and sphenethmoid in derived tyrannosauroids. In the absence of developmental data, it is not clear if the ossification referred to as the sphenethmoid in tyrannosauroids is homologous to that in birds. However, we retain a single character scoring for the presence/absence of an ossified sphenethmoid for simplicity. The Brusatte et al. (2010a) character refers to both an ossified sphenethmoid and mesethmoid, but here we consider only the sphenethmoid in defining this character, so that it is equivalent to the original character in Turner et al. (2012). All known tyrannosauroids with an ossisfied sphenethmoid also possess an ossified mesethmoid, so their presence is probably correlated.

**Character 282: Ossified interorbital septum, mesethmoid:**

0: unossified or small, restricted to the ventral surface of the frontal or marginally extends anteriorly past the premaxillae/frontal contact but does not surpass posterior edge of external nares

1: large, extends anterior to the posterior extent of the frontal processes of premaxillae and anterior to the posterior edge of the external nares

**Character 283: Eustachian tubes, morphology: (UNORDERED)**

0: paired and lateral

1: paired, close to cranial midline

2: paired and adjacent on midline or single anterior opening

**Character 284: Eustachian tubes, ossification:**

0: absent

1: present

**Character 285: Squamosal, ventral (=zygomatic) process, morphology:**

0: large and elongate, dorsally encloses otic process of the quadrate and extends anteroventrally along shaft of quadrate, dorsal head of quadrate not visible in lateral view

1: short, head of quadrate exposed in lateral view

Note: This character is not redundant with Turner et al. (2012, character 216), because this character refers specifically to the size of the ventral process. A reduced ventral process is one means of exposing the quadrate head in lateral view, as is a wide notch between the ventral and posterior processes (which is what is scored in character 216).

**Character 286: Quadrate, orbital process (=pterygoid flange) of quadrate, form of articulation with pterygoid:**

0: pterygoid broadly overlapping medial surface of orbital process (i.e., ‘pterygoid ramus’ present)

1: pterygoid contact restricted to anteromedial edge of orbital process

**Character 287: Quadrate, form of pterygoid articulation with orbital process in those taxa where the contact is restricted to the anteromedial edge of the process: (ORDERED)**

0: pterygoid articulates with anterior-most tip

1: pterygoid articulation does not reach tip

2: pterygoid articulation with no extent up orbital process, restricted to quadrate corpus

Note: All taxa scored for state 0 in the previous character are here scored as inapplicable (“?”).

**Character 288: Quadrate/pterygoid contact, morphology in those taxa where the contact is restricted to the anteromedial edge of the orbital process:**

0: as a facet, variably with slight anteromedial projection cradling base

1: condylar, with a well-projected tubercle on the quadrate

Note: All taxa scored for state 0 for character 286 are here scored as inapplicable (“?”).

**Character 289: Quadrate, well-developed tubercle on anterior surface of dorsal process:**

0: absent

1: present

**Character 290: Quadrate, form of articulation with quadratojugal:**

0: overlapping

1: peg and socket articulation

**Character 291: Quadrate, dorsal process, articulation with other bones:**

0: with squamosal only

1: with squamosal and prootic

**Character 292: Quadrate, dorsal process, development of intercotylar incisure between prootic and squamosal cotylae: (ORDERED)**

0: absent, articular surfaces not differentiated (usually meaning prootic contact was absent)

1: two distinct articular facets, incisure not developed

2: incisure present, ‘double headed’

**Character 293: Quadrate, mandibular articulation, form:**

0: divided into two condyles

1: divided into three condyles, due to additional posterior condyle or broad surface

**Character 294: Quadrate, pneumaticity:**

0: absent

1: present

Note: This character scores specifically for the presence or absence of an internal pneumatic recess in the quadrate. Many coelurosaurs have an internally pneumatic quadrate, but this recess communicates with the external surface of the bone in two distinct ways (Tahara and Larsson 2011). Most coeluorsaurs have a small foramen, usually on the posterior surface of the quadrate, that leads into the recess. Derived tyrannosauroids, on the other hand, possess a large, deep, funnel-like pneumatic opening on the anterior surface of the quadrate where the pterygoid wing and mandibular condyles meet. Both conditions are considered homologous at the level of absence/presence of quadrate pneumaticity here, but we utilize separate characters below for the absence/presence of the posterior foramen and the absence/presence of the anterior funnel. The homologous nature of both conditions is supported by the morphology of *Dilong*, which clearly has an anterior funnel and also a posterior foramen (IVPP V14243; Xu et al. 2004).

**Character 295: Quadrate, cluster of pneumatic foramina on the posterior surface of the tip of the dorsal process:**

0: absent

1: present

Note: Because we are uncertain whether this condition may be homologous to the broader condition of quadrate pneumaticity, we score taxa lacking this character for state 0, even if they are also scored for state 0 (absence) for the general character of quadrate pneumaticity.

**Character 296: Quadrate, form of pneumatization, large single pneumatic foramen on the posterior or posteromedial surface of the shaft:**

0: absent

1: present

Note: See discussion regarding quadrate pneumaticity above. All taxa without quadrate pneumaticity are scored as inapplicable (“?”) for this character.

**Character 297: Articular, pneumaticity:**

0: absent

1: present

**Character 298: Dentary, morphology of posterior end:**

0: approximately straight or with a weakly developed separation into dorsal and ventral forks (i.e., small dorsal ramus)

1: strongly forked with the dorsal and ventral rami approximately equal in posterior extent

**Character 299: Splenial, anterior extent: (ORDERED)**

0: limited, stops well posterior to mandibular symphysis

1: elongate, extends to mandibular symphysis but does not participate in symphysis

2: elongate, extends to anterior tip of mandible to contacting on midline and participate in symphysis

**Character 300: Mandibular symphysis, anteroposteriorly extensive, flat to convex, dorsal-facing surface:**

0: absent, symphysis concave

1: present

**Character 301: Mandibular symphysis, symphyseal foramina:**

0: absent

1: present

**Character 302: Mandibular symphysis, symphyseal foramina, number:**

0: single

1: paired

**Character 303: Mandibular symphysis, symphyseal foramina, location:**

0: opening on posterior edge of symphysis

1: opening on dorsal surface of symphysis

**Character 304: Dentary, Meckelian groove, exposure in medial view:**

0: exposed as deep and conspicuous groove, not completely covered by splenial

1: not exposed, covered by splenial

**Character 305: Lower jaw, anterior external mandibular fenestra:**

0: absent

1: present

**Character 306: Jugal, contact with postorbital:**

0: present

1: absent

**Character 307: Frontal/parietal suture**

0: open

1: fused

**Character 308: Thoracic vertebrae (with ribs articulating with the sternum, or in the middle-posterior dorsal series of theropods without preserved sterna), one or more with prominent hypapophyses:**

0: absent

1: present

**Character 309: Thoracic vertebrae, number: (ORDERED)**

0: 12 or more

1: 11

2: 10 or fewer

**Character 310: Thoracic vertebrae, form of articular surfaces:**

0: at least part of series with round or ovoid articular surfaces (e.g. amphicoelous/ opisthocoelous) that lack the dorsoventral compression seen in heterocoelous vertebrae

1: series completely heterocoelous

**Character 311: Thoracic vertebrae, parapophysis, position:**

0: anterior to transverse process

1: directly ventral to transverse process (close to midpoint of vertebra)

**Character 312: Thoracic vertebrae, centrum, proportions:**

0: approximately equal in anteroposterior length and midpoint mediolateral width

1: length markedly greater than midpoint width

**Character 313: Thoracic vertebrae, morphology of lateral surfaces of centra: (UNORDERED)**

0: flat to slightly depressed

1: deep, emarginated fossae

2: central ovoid foramina

Note: Turner et al. (2012) scored some taxa with large pneumatic foramina in the dorsals for character state 2 (e.g., *Balaur*). Because the size and shape of pneumatic foramina are variable, and because dorsal pneumaticity is already scored in other characters, we restrict state 2 to representing the large, ovoid, central foramina of some avialans (and also *Linhenykus*). Turner et al. (2012) also scored *Tyrannosaurus* for state 1, possibly because of the pneumatic foramen and surrounding fossa on the dorsal centra. We do not consider this homologous to the deeply emarginated fossae on the lateral surfaces of some avialan and oviraptorosaur vertebrae, and therefore consider *Tyrannosaurus* (as well as all other tyrannosauroids and non-maniraptoran coelurosaurs) to be scored for state 0.

**Character 314: Thoracic vertebrae with ossified connective tissue bridging transverse processes:**

0: absent

1: present

**Character 315: Notarium:**

0: absent

1: present

**Character 316: Sacral vertebrae, series of short vertebrae, with dorsally directed parapophyses just anterior to the acetabulum: (ORDERED)**

0: absent

1: present, three such vertebrae

2: present, four such vertebrae

**Character 317: Caudal vertebrae, anterior free caudals prior to transition point, length of transverse processes:**

0: subequal to width of centrum

1: substantially shorter than centrum width

**Character 318: Caudal vertebrae, fusion of distal caudals:**

0: unfused to each other

1: fused to each other

**Character 319: Caudal vertebrae, extent of fused distal caudals: (ORDERED)**

0: fused element length equal or greater than 4 free caudal vertebrae

1: length less than 4 caudal vertebrae

2: less than 2 caudal vertebrae in length

**Character 320: Gastralia:**

0: present

1: absent

**Character 321: Sternum, carina or midline ridge, morphology: (ORDERED)**

0: absent

1: slightly raised

2: distinctly projected

**Character 322: Sternum, position of carina or midline ridge: (UNORDERED)**

0: restricted to posterior half of sternum

1: approaches anterior limit of sternum

2: restricted to the anterior half of the sternum

**Character 323: Sternum, dorsal surface, pneumatic foramen (or foramina):**

0: absent

1: present

**Character 324: Sternum, pneumatic foramina in the depressions (loculi costalis) between rib articulations (processi articularis sternocostalis):**

0: absent

1: present

**Character 325: Sternum, coracoidal sulci spacing on anterior edge: (UNORDERED)**

0: widely separated mediolaterally

1: adjacent

2: crossed on midline

**Character 326: Sternum, number of processes for articulation with the sternal ribs: (ORDERED)**

0: three

1: four

2: five

3: six

4: seven or more

**Character 327: Sternum, raised, paired intermuscular ridges (linea intermuscularis) parallel to sternal midline:**

0: absent

1: present

**Character 328: Sternum, posterior margin, distinct posteriorly projected medial and/or lateral processes, morphology:** (**ORDERED)**

0: absent

1: with distinct posterior processes

2: midpoint of posterior sternal margin connected to medial posterior processes to enclose paired fenestrae

**Character 329: Clavicles, fusion:**

0: left and right bones fused together

1: left and right bones unfused

Note: We here score *Juravenator* as polymorphic “[01]”. Chiappe and Göhlich (2010) described an unfused furcula in *Juravenator*, but because this specimen is such a young individual we cannot rule out that the two clavicles may have later fused in adults. Therefore, we conservatively score it as polymorphic for both conditions.

**Character 330: Clavicles, interclavicular angle between left and right clavicles:**

0: greater than, or equal, to 90 degrees

1: less than 90 degrees

**Character 331: Furcula, lateral excavation:**

0: absent

1: present

**Character 332: Furcula, dorsal (omal) tip, form:**

0: flat or blunt

1: with a pronounced posteriorly pointed tip

**Character 333: Furcula, ventral margin of apophysis, form:**

0: curved, angling

1: with a truncated or squared base

**Character 334: Scapula and coracoid, form of articulation: (UNORDERED)**

0: pit-shaped scapular cotyla developed on the coracoid, and coracoidal tubercle developed on the scapula (‘ball and socket’ articulation)

1: scapular articular surface of coracoid convex

2: flat

**Character 335: Coracoid, procoracoid process:**

0: absent

1: present

**Character 336: Coracoid, lateral margin, form:**

0: straight to slightly concave

1: convex

**Character 337: Coracoid, dorsal surface (= posterior surface of taxa less derived than Paraves), form:**

0: strongly concave

1: flat to convex

Note: This character can be scored by examining the trace of the scapula-coracoid suture in lateral view. Those taxa with state 0 have a strong concavity on the coracoid, whereas those scored for state 1 have either a flat suture or one with a convexity on the coracoid.

**Character 338: Coracoid, pneumaticity:**

0: absent

1: present

**Character 339: Coracoid, position of pneumatic foramen:**

0: proximal

1: distal

**Character 340: Coracoid, lateral process:**

0: absent

1: present

**Character 341: Coracoid, ventral surface (=anterior surface of taxa less derived than Paraves), lateral intermuscular line or ridge:**

0: absent

1: present

**Character 342: Coracoid, position of glenoid facet:**

0: dorsal to, or at approximately same level as, acrocoracoid process/‘biceps tubercle’ (or estimated position of biceps tubercle is a discrete tubercle is absent)

1: far ventral to acrocoracoid process

**Character 343: Coracoid, acrocoracoid process, shape:**

0: straight

1: hooked medially

**Character 344: Coracoid, n. supracoracoideus passes through coracoid (usually via coracoid foramen):**

0: present

1: absent

Note: This character is not equivalent to the presence or absence of a large, discrete coracoid foramen (Turner et al. 2012, character 440). Although a noticeable foramen is sometimes absent in non-avialan coelurosaurs, the overall morphology of the coracoid (and pectoral region in general) suggests that the n. supracoracoideus still passes through the coracoid, unlike the condition in some derived avialans. In the absence of soft tissue information in fossil taxa, we consider all non-paravian theropods with a traditional plate-like coracoid to possess state 0.

**Character 345: Coracoid, medial surface, area of the foramen n. supracoracoideus (when developed)**

0: strongly depressed

1: flat to convex

Note: Turner et al. (2012) scored the outgroup *Allosaurus* and derived tyrannosauroids for state 1, but in these taxa this surface of the coracoid is indeed depressed (the condition in these taxa is basically identical to the ornithomimosaur *Gallimimus*, for instance, which was scored for state 0). Therefore, we score these taxa for state 0. State 1 is now scored only in some avialans, some alvarezsaurids, and *Microvenator*.

**Character 346: Coracoid and scapula, angle between bones at glenoid:**

0: greater than 90 degrees

1: 90 degrees or less

**Character 347: Scapula, ratio of dorsoventral depth of distal end to minimum dorsoventral depth of blade: (ORDERED)**

0: greater than 2.5

1: slightly taller or approximately the same depth as proximal dorsoventral shaft width, ratio between 1.0 and 2.5

2: tapering distally, ratio less than 1.0

Note: This character has been modified into an ordered multistate to take into account variation within tyrannosauroids noted by Brusatte et al. (2010a, character 235).

**Character 348: Scapula, curvature in lateral view:**

0: absent, bone straight

1: present, bone dorsoventrally curved

Note: Turner et al. (2012) scored some basal coelurosaurs (*Haplocheirus*, *Mononykus*, *Archaeornithomimus*, *Gallimimus*) for the curved condition, but these taxa have straight scapular blades that are extremely similar in overall shape to closely related that that were scored for the straight condition. We score them for the straight condition here, meaning that the curved condition is restricted to some derived maniraptorans.

**Character 349: Scapula, position of acromion process:**

0: extends anteriorly to surpass the articular surface for coracoid (facies articularis coracoidea)

1: does not extend further anteriorly than the articular surface for coracoid

**Character 350: Scapula, orientation of acromion process:**

0: straight

1: laterally hooked tip

Note: Turner et al. (2012) scored tyrannosauroids for state 1, which is otherwise present only in some derived avialans. We do not recognize any discrete difference in the orientation of the acromion process of derived tyrannosauroids relative to other basal coelurosaurs, and therefore score all tyrannosauroids with well-preserved scapulae for state 0.

**Character 351: Humerus and ulna, length comparison: (ORDERED)**

0: humerus longer than ulna

1: ulna and humerus approximately the same length (within ~10% of each other)

2: ulna substantially longer than humerus

**Character 352: Humerus, head, shape in anterior or posterior view**

0: strap-like, articular surface flat or weakly convex, no proximal midline convexity

1: prominent and highly convex (domed) proximally

Note: We consider this character equivalent to character 240 in Brusatte et al. (2010a), which scores for the absence/presence of an enlarged head that occupies the majority of the proximal end, is bulbous in roximal view, and overhangs both anterior and posterior surfaces. This feature, which is seen in some derived tyrannosauroids, is here considered equivalent to state 1.

**Character 353: Humerus, proximal end, shape of proximal projection:**

0: dorsal edge projected farthest

1: midline projected farthest

**Character 354: Humerus, ventral tubercle and capital incisure:**

0: absent

1: present

**Character 355: Humerus, capital incisure, morphology:**

0: an open groove

1: closed by tubercle associated with a muscle insertion just distal to humeral head

**Character 356: Humerus, anterior surface, well-developed fossa on midline making proximal articular surface appear v-shaped in proximal view:**

0: absent

1: present

**Character 357: Humerus, ‘transverse groove’:**

0: absent

1: present, developed as a discrete, depressed scar on the proximal surface of the bicipital crest or as a slight transverse groove

**Character 358: Humerus, deltopectoral crest, orientation:**

0: projected laterally or dorsally, such that it is in line with the long axis of humeral head

1: projected anteriorly, such that it is approximately perpendicular to the long axis of the head

**Character 359: Humerus, deltopectoral crest, thickness compared with thickness of shaft: (ORDERED)**

0: less

1: same width

2: dorsoventral thickness greater than shaft thickness

**Character 360: Humerus, deltopectoral crest, proximoposterior surface (=proximolateral surface in most non-avialan theropods), form:**

0: flat to convex

1: concave

**Character 361: Humerus, deltopectoral crest, large fenestra:**

0: absent, crest not perforate

1: present, crest perforate

**Character 362: Humerus, bicipital crest, pit-shaped scar/fossa for muscular attachment on anterodistal, distal, or posterodistal surface of crest:**

0: absent

1: present

**Character 363: Humerus, bicipital crest, position of pit-shaped fossa for muscular attachment: (UNORDERED)**

0: anterodistal on bicipital crest

1: directly ventrodistal at tip of bicipital crest

2: posterodistal, variably developed as a fossa

**Character 364: Humerus, bicipital crest, anterior projection: (ORDERED)**

0: absent or subtle

1: present, developed as an anterior projection relative to shaft surface in ventral view

2: present as a hypertrophied, rounded tumescence

**Character 365: Humerus, proximal end, one or more pneumatic foramina:**

0: absent

1: present

**Character 366: Humerus, distal articular condyles, position:**

0: on distal surface of bone

1: on anterior surface of bone

**Character 367: Humerus, long axis of dorsal (lateral) distal condyle, orientation:**

0: at low angle to humeral axis, proximodistally orientated

1: at high angle to humeral axis, almost transversely orientated

**Character 368: Humerus, distal condyles, form:**

0: prominent, subround and bulbous

1: weakly defined, ‘strap-like’

**Character 369: Humerus, distal margin, orientation:**

0: approximately perpendicular to long axis of humeral shaft

1: oblique (angling strongly ventrally) to long axis of humeral shaft, ventrodistal margin projected significantly distal to dorsodistal margin (sometimes described as a well-projected flexor process)

**Character 370: Humerus, distal end, compressed anteroposteriorly and flared dorsoventrally:**

0: absent

1: present

**Character 371: Humerus, brachial fossa:**

0: absent

1: present, developed as a flat scar or as a scar-impressed fossa

**Character 372: Humerus, ventral (medial) distal condyle, length of long axis:**

0: less than the long axis of the dorsal (lateral) condyle

1: the same or greater than the long axis of the dorsal (lateral) condyle

**Character 373: Humerus, demarcation of muscle origins (e.g. m. extensor metacarpi radialis in Aves) on the dorsal (=lateral in non-avialan theropods) edge of the distal humerus:**

0: no indication of origin as a scar, a pit, or a tubercle

1: indication as a pit-shaped scar or as a variably projected scar-bearing tubercle or facet

**Character 374: Humerus, groove for passage of m. scapulotriceps on distal end of posterior surface:**

0: absent

1: present

**Character 375: Humerus, m. humerotricipitalis groove:**

0: absent

1: present as a ventral depression contiguous with the olecranon fossa

**Character 376: Ulna, cotylae, orientation of dorsal (lateral) and ventral (medial) cotylae:**

0: dorsoventrally adjacent

1: widely separated by a deep groove

**Character 377: Ulna, dorsal cotyla, form: convex**

0: flat or non-distinct

1: convex

**Character 378: Ulna, distal end, dorsal (lateral) condyle, dorsal trochlear surface, extent along posterior margin:**

0: less than transverse measure of dorsal trochlear surface

1: approximately equal in extent

**Character 379: Ulna, bicipital scar: (ORDERED)**

0: absent

1: present, developed as a slightly raised scar

2: present, developed as a conspicuous tubercle

**Character 380: Ulna, brachial scar**

0: absent

1: present

**Character 381: Radius, posteroventral surface (=posteromedial surface of non-avialans), texture: (UNORDERED)**

0: smooth

1: with muscle impression along most of surface

2: deep longitudinal groove

**Character 382: Ulnare:**

0: absent

1: present

**Character 383: Ulnare, shape:**

0: circular, triangular, or ‘heart-shaped’, with no or minimal differentiation into short dorsal and ventral rami

1: V-shaped, well- developed dorsal and ventral rami

**Character 384: Ulnare, ventral ramus (crus longus), length: (ORDERED)**

0: shorter than dorsal ramus (crus brevis)

1: same length as dorsal ramus

2: longer than dorsal ramus

**Character 385: Semilunate carpal and metacarpals, fusion: (ORDERED)**

0: separate from each other, no fusion

1: incomplete proximal fusion

2: complete proximal fusion

3: complete proximal and distal fusion

**Character 386: Metacarpal III, anteroposterior (extensor-flexor) diameter at midshaft compared to anteroposterior diameter of metacarpal II:**

0: approximately equal or greater than 50%

1: less than 50%

**Character 387: Metacarpal I, anteroproximally projected muscular process, form: (ORDERED)**

0: absent, no distinct process visible

1: present as small knob at anteroproximal tip of metacarpal

2: present, tip of process marginally surpasses the distal articular facet for phalanx 1 in anterior extent

3: present, tip of process conspicuously surpasses articular facet by approximately half the width of the facet, producing a pronounced knob

4: present, tip of process conspicuously surpasses articular facet by approximately the entire width of the facet, producing a pronounced knob

**Character 388: Metacarpal I, anterior (extensor) surface, shape:**

0: roughly hourglass-shaped proximally, at least moderately expanded anteroposteriorly, and constricted just before flare of articulation for phalanx 1

1: anterior surface broadly convex

**Character 389: Carpometacarpus, pisiform process:**

0: absent

1: present

**Character 390: Carpometacarpus, ventral surface, supratrochlear fossa deeply excavating proximal surface of pisiform process or adjacent region:**

0: absent

1: present

**Character 391: Carpometacarpus, intermetacarpal space (between metacarpals II and III):**

0: reaches proximally as far as the distal end of metacarpal I

1: terminates distal to end of metacarpal I

Note: All taxa without a carpometacarpus are scored as inapplicable (“?”).

**Character 392: Metacarpals II and III, distal ends, position of articular surfaces for digits:**

0: articular surface on metacarpal II located at same distal level as, or surpasses distally, articular surface on metacarpal III

1: articular surface on metacarpal III extends further distally than articular surface on metacarpal II

**Character 393: Metacarpals, intermetacarpal process or tubercle: (ORDERED)**

0: absent

1: present as scar

2: present as tubercle or flange

**Character 394: Manual digit II, phalanx 1, shape:**

0: subcylindrical to subtriangular in cross section

1: strongly dorsoventrally compressed, flat posterior surface

**Character 395: Manual digit II, length of phalanx II-1 compared to that of II-2:**

0: less than or equal to

1: longer

**Character 396: Manual digit II, phalanx 2, internal index process on posterodistal edge:**

0: absent

1: present

**Character 397: Pelvis, ilium, ischium, and pubis, proximal contact between bones in adult individuals: (ORDERED)**

0: unfused to each other

1: partially fused to each other (pubis not ankylosed)

2: completely fused to each other

Note: State 0 includes the condition in derived therizinosauroids in which the pubis and obturator process of the ischium meet and fuse ventral to the acetabulum, which does not include fusion between the ilium and other pelvic bones.

**Character 398: Ilium/ischium, distal co-ossification to completely enclose the ilioischiadic fenestra:**

0: absent

1: present

**Character 399: Ischium, dorsal process**

0: does not contact ilium

1: contacts ilium

Note: All taxa without a dosrsal process are scored as inapplicable (“?”).

**Character 400: Ilium, antitrochanter, position:**

0: directly posterior to acetabulum

1: posterodorsal to acetabulum

**Character 401: Ilium, preacetabular pectineal process: (ORDERED)**

0: absent

1: present as a small flange

2: present as a well-projected flange

**Character 402: Ilium, preacetabular processes, orientation of left and right processes:**

0: approach on midline but do not contact (open space between them, possibly a cartilaginous connection)

1: make contact with each other on midline and fuse, dorsal closure of ‘iliosynsacral canals’

**Character 403: Ilium, preacetabular process, morphology of process as it extends anterior to first sacral vertebra:**

0: no free ribs overlapped

1: one or more ribs overlapped

**Character 404: Ilium, postacetabular process, orientation:**

0: dorsoventrally orientated, such that broad external surface faces laterally

1: mediolaterally orientated, such that broad external surface faces dorsally

**Character 405: Ilium, postacetabular process, ventral surface, renal fossa:**

0: absent

1: present

**Character 406: Ilium, cuppedicus fossa, form:**

0: broad, mediolaterally oriented surface directly anteroventral to acetabulum

1: small and entirely laterally facing fossa anterior to the acetabulum

Note: All taxa without a cuppedicus fossa (scored as absent for the character denoting the absence/presence of the fossa above) are scored here as inapplicable (“?”).

**Character 407: Pubis, cross section:**

0: suboval

1: compressed mediolaterally

**Character 408: Pubes, contact of distal ends of left and right pubes:**

0: present, variably co-ossified into symphysis

1: absent, pubes noncontacting

**Character 409: Femur, posterior trochanter: (ORDERED)**

0: present, developed as a slightly projected tubercle or flange

1: present, hypertrophied into a ‘shelf-like’ conformation (in combination with development of the trochanteric shelf)

2: absent

**Character 410: Femur, patellar groove**

0: absent

1: present

Note: Turner et al. (2012) scored derived tyrannosauroids for possessing a patellar groove, probably because these taxa have a deep extensor groove for muscle attachment (a feature that is variably developed in tetanurans). We do not consider this feature homologous to the distinct patellar groove of birds, which houses a patella ossification (which is not known for tyrannosauroids or any other non-avialan theropod). Instead, we utilize a separate character (below) to refer to the extensor groove.

**Character 411: Femur, ectocondylar tubercle and lateral condyle, separation:**

0: present, separated by deep notch

1: absent, form single trochlear surface

**Character 412: Femur, posterior projection of the lateral border of the distal end, continuous with lateral condyle:**

0: absent

1: present

**Character 413: Femur, laterally projected fibular trochlea: (ORDERED)**

0: absent

1: present, developed as small notch

2: present, developed as a shelf-like projection

**Character 414: Tibia/tarsals, condyles, extent of medial and lateral condyles:**

0: medial condyle projecting further anteriorly than lateral

1: equal in anterior projection

**Character 415: Tibia/tarsals, condyles, extensor canal: (ORDERED)**

0: absent

1: present as an emarginated groove

2: present as a groove bridged by an ossified supratendoneal bridge

**Character 416: Tibia/tarsals, condyles, tuberositas retinaculi extensoris indicated by short medial ridge or tubercle proximal to the condyles close to the midline and a more proximal second ridge on the medial edge:**

0: absent

1: present

**Character 417: Tibia/tarsals, condyles, mediolateral widths of lateral and medial condyles: (ORDERED)**

0: medial condyle wider

1: approximately equal

2: lateral condyle wider

**Character 418: Tibia/tarsals, condyles, medial constriction of lateral and medial condyles:**

0: present, gradual sloping medial constriction of condyles

1: absent, no medial tapering of either condyle

**Character 419: Tibia/tarsals, condyles, intercondylar groove, mediolateral width:**

0: broad, approximately 1/3 width of anterior surface

1: narrow, less than 1/3 width of total anterior surface

**Character 420: Tibia/tarsals, position of articular surface for distal tarsals/tarsometatarsus: (ORDERED)**

0: on distal surface or restricted to distal-most edge of posterior surface, no broad extension of trochlear surface onto posterior surface of bone

1: well-developed articular surface extending up the posterior surface of the tibiotarsus (sulcus cartilaginis tibialis of Aves)

2: well-developed articular surface extending up the posterior surface of the tibiotarsus, with well-developed and posteriorly projecting medial and lateral crests

**Character 421: Tibia, mediolateral width of distal end:**

0: wider than midpoint of shaft, giving distal profile a weakly developed triangular form

1: approximately equal to midshaft width, no distal expansion of whole shaft (although condyles may be variably splayed mediolaterally)

**Character 422: Metatarsal V:**

0: present

1: absent

**Character 423: Metatarsal III, position of proximal end:**

0: proximally in same plane with II and IV

1: proximally displaced plantarly (to the extensor surface), relative to metatarsals II and IV

**Character 424: Tarsometatarsus or metatarsals, intercotylar eminence:**

0: absent

1: well developed, globose

**Character 425: Tarsometatarsus or metatarsals, projected surface or grooves on proximoposterior surface (associated with the passage of tendons of the pes flexors in Aves; hypotarsus): (ORDERED)**

0: absent

1: developed as posterior projection with flat posterior surface

2: developed as a posterior projection, with distinct crests and grooves

3: developed as a posterior project with distinct crests and grooves, at least one groove enclosed by bone posteriorly

**Character 426: Tarsometatarsus or metatarsals, proximal vascular foramina: (ORDERED)**

0: absent

1: single foramen present, between metatarsals III and IV

2: two foramina present

**Character 427: Metatarsal I, shape: (ORDERED)**

0: straight

1: curved or distally deflected but not twisted, ventral surface convex (‘J shaped’)

2: distally deflected and twisted, such that the ventromedial surface is concave proximal to trochlear surface for phalanx I

**Character 428: Metatarsal II, distal extensor surface, fossa for metatarsal I: (ORDERED)**

0: absent

1: present as a shallow notch

2: present as a conspicuous ovoid fossa

**Character 429: Metatarsal IV, mediolateral width at midshaft: (UNORDERED)**

0: metatarsal IV approximately the same width as metatarsals II and III

1: metatarsal IV narrower than MII and MIII

2: metatarsal IV greater in width than either metatarsal II or III

**Character 430: Metatarsals, comparative trochlear width (UNORDERED):**

0: II approximately the same size as III and/or IV (this includes taxa in which metatarsal III is slightly wider than the other metatarsals disally)

1: II markedly wider than III and/or IV

2: II markedly narrower than III and/or IV

3: IV markedly narrowest.

**Character 431: Metatarsus, distal vascular foramen, form:**

0: simple, with one exit

1: forked, two exits (plantar and distal) between metatarsals III and IV.

**Character 432: Metatarsal III, distal trochlea in extensor view, proximal extent of lateral and medial edges:**

0: equally extended proximally

1: lateral edge extends further proximally

**Character 433: Metatarsal II, distal extent of metatarsal II relative to metatarsal IV:** (**ORDERED)**

0: approximately equal in distal extent

1: metatarsal II shorter than metatarsal IV, but reaching distally further than base of metatarsal IV trochlea

2: metatarsal II shorter than metatarsal IV, reaching distally only as far as base of metatarsal IV trochlea

**Character 434: Caudal vertebrae, middle to posterior caudals, anteroposterior length: (ORDERED)**

0: shortened, less than 1.5x length of dorsal vertebrae (where known) and anteroposterior length of centrum less than twice its maximum mediolateral width

1: 1.5x-2x or less the length of dorsal vertebrae

2: 3x-4x length of dorsal vertebrae

Note: This is a modified version of the original Turner et al. (2012) character, which takes into account an additional state (denoted as state 0) noted by Zanno et al. (2009).

**Character 435: Coracoid, coracoid fenestra:**

0: absent

1: present

**Character 436: Metatarsal V, elongated and bowed**

0: absent

1: present

**Character 437: Chevrons, degree of posterior extension of posterior chevrons:**

0: not substantially elongated

1: very elongated

**Character 438: Radius, width at midshaft:**

0: roughly half or greater than width of ulna

1: less than half width of ulna

**Character 439: Manus, combined length of metacarpal I and phalanx I-1:**

0: greater than length of MC II

1: equal to or less than length of MC II

**Character 440: Metacarpal III, shape:**

0: straight

1: bowed

**Character 441: Metatarsal I, position of distal trochlea:**

0: proximally placed relative to trochleae of other metatarsals

1: inline distally with others

**Character 442: Metatarsal I:**

0: present

1: absent

**Character 443: Braincase, preotic pendant, form: (ORDERED)**

0: absent

1: present but small

2: present and robust

**Character 444: Braincase, metotic strut, shape:**

0: short and robust

1: long and narrow

**Character 445: Prootic, pneumatic excavation on lateral surface leading into anterior tympanic recess: (ORDERED)**

0: absent

1: present and shallow

2: present and deep

Note: Witmer (1997), Makovicky and Norell (1998), Bever et al. (2013) and others have described lateral pneumatic excavations on the prootic, which are present in some coelurosaurs, to be associated with the anterior tympanic recess, which is present in most theropods. The character here does not code for the absence/presence of the recess, but rather for the absence/presence of discrete external pneumatic openings on the lateral surface of the prootic that lead into the recess. Contra Turner et al. (2012), tyrannosauroids are scored for state 1, as they possess a distinct fossa on the lateral surface of the prootic that includes the external foramina of the trigeminal and facial nerves, along with pneumatic openings that lead into the anterior tympanic recess (Bever et al. 2013). This fossa itself is a distinct tyrannosauroid character, which is listed as a separate character below.

**Character 446: Braincase, anterior tympanic recess (ATR), manifestation on external surface of basisphenoid: (ORDERED)**

0: subtle, external fossa not deeply impressed into the lateral surface of the basisphenoid

1: deeply impressed into the lateral surface of the basisphenoid in juveniles but not adults

2: deeply impressed into the lateral surface of the basisphenoid in adults

Note: Turner et al. (2012) originally scored for the absence/presence of an anterior tympanic recess. Witmer (1997), Bever et al. (2013), and others have shown that this recess is present in most theropods, so we have modified this character to refer specifically to the external manifestation of the recess on the basisphenoid. This character scores for a shallow/deep lateral fossa or fossae on the basisphenoid. We have also subdivided the “deeply impressed” condition into two states, to take into account the recognized loss of a deeply impressed recess during ontogeny in some tyrannosauroids (Bever et al. 2013). This character is therefore equivalent to Brusatte et al. (2010a, character 157).

**Character 447: Braincase, anterior tympanic recess and anterior tympanic crista (crest marking the posterior and dorsal border of the ATR), location:**

0: below the exit foramen of cranial nerve VII exit and just proximal to the otic recess

1: anteriorly, with little or no development of the recess posterior to the basipterygoid processes

**Character 448: Braincase, anterior tympanic recess confluent with the subotic recess:**

0: absent

1: present, forming the Lateral Depression

Note: All taxa without a subotic recess are scored as inapplicable (“?”).

**Character 449: Braincase, V-shaped opening between basal tubera (or remnants of tubera):**

0: absent

1: present

**Character 450: Braincase, small tubera medial to basal tubera (or remnants of basal tubera) and ventral to occipital condyle:**

0: absent

1: present

**Character 451: Pedal phalanx II-2, size of distal articular surface relative to proximal articular surface:**

0: approximately equal in size or distal surface slightly smaller than proximal surface

1: distal surface less than half the size of proximal surface

**Character 452: Sternum, ossification of sternal plates:**

0: absent, plates unossified

1: present, plates ossified

**Character 453: Ulna, size of proximal cotylae:**

0: unequal in size, lateral (=dorsal in avialans) condyle smaller

1: equal in size

**Character 454: Braincase, middle ear resides within the Lateral Depression (formed by merged anterior tympanic recess and subotic recess):**

0: absent

1: present

**Character 455: Feathers, filamentous integumentary structures (Stage 1 feathers):**

0: absent

1: present

**Character 456: Feathers, vaned feathers (Stage 4 feathers):**

0: absent

1: present

**Character 457: Quadratojugal, size:**

0: large

1: greatly reduced

**Character 458: Frontal, notch for postorbital contact on postorbital process of frontal:**

0: absent, process smooth or facet small

1: large notch present

**Character 459: Frontal and parietal, position of fronto-parietal suture relative to postorbital processes of frontal: (ORDERED)**

0: well posterior to the postorbital processes

1: at the level of the postorbital processes

2: anterior to postorbital processes

**Character 460: Cervical vertebrae, orientation of articular surfaces between cervical vertebrae:**

0: surfaces vertical to subvertical

1: strongly slanted anteroventrally

**Character 461: Skull roof, accessory depression in supratemporal fossa:**

0: absent

1: present

**Character 462: Ilium, relative ventral extension of pubic and ischiadic peduncles: (ORDERED)**

0: equal

1: pubic peduncle extends farther ventrally

2: pubic peduncle hyperelongate, approximately 2.5-3 times the proximodistal length of the ischial peduncle

Note: This is a modified version of the original Turner et al. (2012) character, which incorporates a second derived state referred to the hyperelongate condition of some derived therizinosauroids (see Zanno et al. 2009: character ZCD 311). We also consider this character to be equivalent to Zanno et al. (2009: character ZCD 309).

**Character 463: Parasphenoid, ala parasphenoidalis:**

0: absent

1: present, well-developed and crest-shaped forming anterior edge of enlarged pneumatic recess with the ala continuous with the anterior tympanic crista

**Character 464: Furcula, cross-section:**

0: nearly circular

1: anteroposteriorly compressed near the symphysis

**Character 465: Furcula, shape:**

0: V-shaped

1: U-shaped

**Character 466: Furcula, epicledial processes, form:**

0: unexpanded

1: expanded

**Character 467: Furcula, lateral expansion of the rami between the hypocledium and the epicledial process:**

0: absent

1: present

**Character 468: Furcula, hypocledium, shape:**

0: rounded

1: keeled

**Character 469: Furcula, symmetry:**

0: asymmetrical

1: nearly symmetrical

**Character 470: Furcula, thickness of rami:**

0: thin

1: thick

**Character 471: Metatarsal III, extensor (=anterior or dorsal) surface, shape:**

0: relatively narrow and flat

1: transversely expanded and slightly concave

**Character 472: Metatarsal IV, accessory longitudinal ridge on anterolateral side of the distal end of the bone:**

0: absent

1: present

**NEW CHARACTERS ADDED TO THE TWiG DATASET HERE:**

***Expansions of Turner et al. (2012) Characters:**

**Character 473: Metacarpal I, width of proximal articular surface compared to proximodistal length of entire metacarpal:**

0: shorter

1: wider

Note: This character is a new character, originally part of Turner et al. (2012, character 149), which distinguishes the extremely short and wide metacarpals I of some alvarezsaurids.
**Character 474: Manual unguals:**

0: present

1: absent

Note: This character is a new character, originally part of Turner et al. (2012, character 151), which distinguishes the absent manual unguals of derived avialans.

**Character 475: Manual unguals, form of flexor tubercle: (ORDERED)**

0: large, robust, rugose, conical structure

1: reduced to a small convexity

2: absent

Note: This character is a new character, originally part of Turner et al. (2012, character 151), which distinguishes the large flexor tubercles of most coelurosaurs from the small flexor tubercles of some tyrannosauroids, ornithomimosaurs, and other taxa. Some alvarezsauroids have completely absent flexor tubercles, which is denoted by state 2. This character is equivalent to Brusatte et al. (2010a, character 255).

***Characters Relevant to Tyrannosauroidea (from Brusatte et al. 2010a):**

**Character 476: Skull, occipital region, orientation:**

0: posteriorly

1: posteroventrally

**Character 477: Skull, general shape:**

0: long and low, length: height ratio greater than 3.2

1: deep, length: height ratio less than 3.2

Note: Length is premaxilla-quadrate cotyle length; height is maximum height of the upper jaw, not counting any cranial crests.

**Character 478: Skull, anteroposterior length:**

0: less than 40% trunk length

1: greater than 40% trunk length

Note: Trunk length is the anterior extremity of the pectoral girdle to the posterior extremity of the pelvic girdle, as defined by Sereno et al. (2009).

**Character 479: Lateral temporal fenestra, orientation of long axis relative to long axis of orbit:**

0: posterodorsal

1: approximately parallel

**Character 480: Premaxilla, nasal process of opposing premaxillae, orientation:**

0: divergent from each other, with small process of nasals fitting in between them

1: closely appressed to each other

**Character 481: Premaxilla, deep foramen or fossa on the lateral surface of the base of the nasal process, within the anteroventral corner of the narial fossa:**

0: absent

1: present

**Character 482: Premaxilla, maxillary process orientation: (ORDERED)**

0: mostly laterally (and resultantly widely visible in lateral view)

1: dorsolaterally (facing almost equally dorsally and laterally)

2: dorsally (and resultantly mostly hidden in lateral view)

**Character 483: Premaxilla, form of narial fossa ventral to external naris:**

0: shallowly excavated

1: deeply excavated, anterior margin invaginated as a deep groove

**Character 484: Premaxilla, extent of narial fossa:**

0: limited to region immediately ventral to external naris

1: extensive, covers most of main body of premaxilla

**Character 485: Premaxilla, orientation and shape of anterior margin: (ORDERED)**

0: smoothly curved and projecting posterodorsally, angle between ventral margin of premaxilla and anterior margin is less than 90 degrees;

1: smoothly curved and projecting vertically or slightly anterodorsally, angle between ventral margin of premaxilla and anterior margin is equal to or greater than 90 degrees

2: projecting vertically or slightly anterodorsally, with a discrete inflection point between a more vertical ventral portion and a more horizontal dorsal portion

Note: We have transformed the original character of Brusatte et al. (2010a: character 13) into an ordered multistate by adding a new intermediate state referring to an approximately vertical (or slightly anterodorsally inclined) anterior margin of the ventral portion of the premaxilla. State 2 now refers to a special condition of this state, in which the anterior margin is vertical (or anterodorsally inclined) and there is also a discrete inflection point between the more vertical ventral portion of the anterior margin and the more horizontal dorsal portion of the margin. Taxa with this condition, therefore, do not possess a smoothly curved anterior margin of the premaxilla. Tyrannosauroids are characterized by either state 1 or state 2, with state 2 referring to a subset of basal tyrannosauroids (*Dilong*, *Guanlong*, *Kileskus*, *Proceratosaurus*, *Sinotyrannus*).

**Character 486: Premaxilla, position of palatal process:**

0: immediately above interdental plates

1: separated from interdental plates by deep lingual surface of premaxilla

**Character 487: Maxilla, promaxillary fenestra, position:**

0: anterior margin of antorbital fossa

1: extreme anteroventral corner of antorbital fossa

**Character 488: Maxilla, maxillary fenestra, anteroposterior length compared to the distance between the anterior margins of the antorbital fossa and fenestra: (ORDERED)**

0: less than half

1: greater than half

2: greater than half and also greater than half of the length of the eyeball-bearing portion of the orbit

**Character 489: Maxilla, maxillary fenestra, position within maxillary antrum:**

0: does not abut dorsal border of the antrum in medial view

1: abuts dorsal border of the antrum in medial view

**Character 490: Maxilla, antorbital fossa, extent:**

0: reaches nasal suture

1: does not reach nasal suture

**Character 491: Maxilla, interfenestral strut, anteroposterior length:**

0: greater than 50% of long axis of maxillary fenestra

1: less than 50% of long axis of maxillary fenestra

Note: In oviraptorosaurs, the size of the interfenestral strut is measured relative to the “accessory antorbital fenestra,” whose homology with the maxillary and/or premaxillary fenestrae of other theropods is not clear (e.g., Balanoff et al. 2009).

**Character 492: Maxilla, antorbital fossa, trend of dorsoventral depth across main body:**

0: uniform

1: diminishes

**Character 493: Maxilla, subcutaneous flange bordering the antorbital fossa laterally on the posterior end of the main body, resulting in the fossa forming a channel between the flange and the main body:**

0: absent

1: present

**Character 494: Maxilla, dorsolateral process, coverage by antorbital fossa: (ORDERED)**

0: process absent

1: process covered by subcutaneous surface only

2: ventral half of process covered by antorbital fossa

3: antorbital fossa completely excluded

**Character 495: Maxilla, narrow region of smooth surface texture between anterior margin of antorbital fossa and the subcutaneous surface:**

0: absent

1: present

**Character 496: Maxilla, ventral margin of the anterior region of the bone, profile:**

0: straight

1: convex

**Character 497: Maxilla, joint surface for palatine, depth:**

0: shallow, does not obscure the tooth root bulges from view

1: deep, obscures tooth root bulges from view

**Character 498: Maxilla, anterior ramus (demarcated by concave step in anterior margin of maxilla):**

0: absent

1: present

**Character 499: Maxilla, form of contact with nasal in subadult to adult specimens: (ORDERED)**

0: smooth

1: weakly scalloped

2: deeply scalloped with interlocking transverse ridges on both elements

**Character 500: Maxilla, form of external subcutaneous surface texturing:**

0: random foramina and shallow grooves and ridges

1: deep, prominent, dorsoventrally trending grooves and ridges

**Character 501: Maxilla, swollen rim separating antorbital fossa and subcutaneous surface:**

0: present

1: absent

**Character 502: Maxilla, size of ascending ramus, anteroposterior chord directly above maxillary fenestra compared to dorsoventral depth of maxilla below anterior edge of antorbital fenestra:**

0: greater than 1.75 times (ascending ramus large)

1: less than 1.60 times (ascending ramus small)

**Character 503: Maxilla, posterior region of the main body (portion including the final 3-5 teeth and anterior to the jugal process), shape:**

0: maintains a relatively constant dorsoventral depth

1: tapers in depth posteriorly

**Character 504: Maxilla, primary row of neurovascular foramina, form:**

0: continues as a row posteriorly

1: transitions posteriorly into a sharp groove, paralleling the antorbital fossa rim

**Character 505: Maxilla, antorbital fossa, extent on main body:**

0: covers more than half of the depth of the main body beneath the anterior margion of the antorbital fenestra

1: covers less than half of this depth

**Character 506: Nasals, midline crest on dorsal surface:**

0: absent

1: present

**Character 507: Nasal, shape in dorsal view: (UNORDERED)**

0: expands in width posteriorly

1: relatively constaint width across the length of the bone, due to subparallel lateral sides

2: tapers in width posteriorly

**Character 508: Nasal, frontal process, mediolateral width:**

0: unconstricted

1: constricted, less than ½ width of widest point of nasal

**Character 509: Nasal, posterolateral process that overlaps the lateral surface of the lacrimal:**

0: absent

1: present

**Character 510: Nasal, extent of narial fossa on premaxillary process:**

0: limited to ventral margin of process

1: covers entire process, and thus meets opposite fossa on dorsal midline

**Character 511: Nasal, medial processes of frontal articulation, shape: (UNORDERED)**

0: processes absent or very subtle

1: lanceolate

2: tapered

**Character 512: Nasal, thin, low, and laterally projecting crest at the corner where lateral and dorsal surfaces meet:**

0: absent

1: present

**Character 513: Lacrimal, angle between anterior and ventral rami**

0: 90 degrees (=inverted L shaped) or greater

1: approximately 70-80 degrees (=7 shaped)

**Character 514: Lacrimal, cornual process on dorsal surface, form: (UNORDERED)**

0: barely perceptible ridge across entire length of anterior ramus (equivalent to the state “cornual process absent” in tyrannosauroid-specific datasets)

1: broad, shallow, dorsally convex, laterally overhanging swelling across most of the length of the anterior ramus

2: discrete conical projection

3: small, conical, smooth projection that rises 2-3 millimeters from skull roof

Note: All taxa without a cornual process (those scored as absent for character 37) are here scored as inapplicable (“?”).

**Character 515: Lacrimal, cornual process, form:**

0: smoothly rounded

1: discrete apex present

Note: All taxa without a discrete cornual process (those scored as absent for character 37 and those scored as 0 for character 514) are here scored as inapplicable (“?”).

**Character 516: Lacrimal, cornual process, position of apex:**

0: dorsal to ventral ramus

1: anterior to ventral ramus

Note: All taxa without a discrete cornual process (those scored as absent for character 37 and those scored as 0 for character 514 and 515) are here scored as inapplicable (“?”).

**Character 517: Lacrimal, anterior ramus, pneumaticity:**

0: not inflated

1: inflated by pneumatic recess

**Character 518: Lacrimal, size of primary external opening for lacrimal recess:**

0: small, anterior end located approximately at the same level as the anterior end of the ventral ramus

1: large, anterior end located far anterior to the ventral ramus

**Character 519: Lacrimal, interaction of primary external opening for lacrimal recess and antorbital fossa:**

0: separate

1: blend

**Character 520: Lacrimal, accessory external openings for lacrimal recess on the anterior ramus: (ORDERED)**

0: absent

1: present and proximally located (i.e., near to primary recess)

2: present and distally located

**Character 521: Lacrimal, pneumatic recess opening internally onto medial surface of bone as a discrete pneumatic fenestra (pneumatopore):**

0: absent

1: present

**Character 522: Lacrimal, dorsal prong of anterior ramus for articulation with maxilla (“anterodorsal process”), size:**

0: absent or small

1: present and elongate

Note: We have combined “absent” and “small” into a single character statement, because the subtle process can easily be mistaken for absent if it is broken or poorly preserved.

**Character 523: Lacrimal, ventral ramus, extent of medial lamina:**

0: greater than half of the dorsoventral depth of the ramus

1: half or less of the dorsoventral depth of the ramus

**Character 524: Lacrimal, orbitonasal ridge on medial surface, position:**

0: anterior to posterior margin of ventral ramus

1: adjacent to or contacting posterior margin of ventral ramus

**Character 525: Lacrimal, articulation with frontal, form:**

0: squamous

1: conical lacrimal process set into deep pit in frontal

**Character 526: Lacrimal, posterior process for articulation with frontal, inflated by pneumatic recess:**

0: no

1: yes

**Character 527: Lacrimal, extent of antorbital fossa on ventral ramus:**

0: covers greater than 60% of anteroposterior length along the contact with the jugal

1: covers less than this measure

**Character 528: Lacrimal, maxillary process of anterior ramus, visibility in lateral view:**

0: both dorsal and ventral margins visible

1: dorsal margin concealed by subcutaneous surface abouve antorbital fossa and only ventral margin visible

Note: Those taxa without a maxillary process (“dorsal prong” in character 522) are here scored as inapplicable (“?”).

**Character 529: Jugal, maxillary ramus, depth:**

0: shallow, not expanded relative to subortibal portion of bone

1: deep, expanded relative to suborbital portion of bone

**Character 530: Jugal, antorbital fossa, extent on maxillary ramus:**

0: edge of fossa undercut and continues posterodorsal to jugal recess

1: fossa edge does not extend past the jugal recess

Note: Those taxa without a jugal recess (e.g., most ornithomimosaurs) are here scored as inapplicable (“?”) for this character.

**Character 531: Jugal, pneumatic recess, location relative to ventral ramus of lacrimal:**

0: ventral

1: anterior

Note: Those taxa without a jugal recess (e.g., most ornithomimosaurs) are here scored as inapplicable (“?”) for this character.

**Character 532: Jugal, pneumatic recess, orientation of long axis:**

0: approximately horizontal

1: inclined at approximately 45 degrees relative to the ventral skull margin

Note: Those taxa without a jugal recess (e.g., most ornithomimosaurs) are here scored as inapplicable (“?”) for this character.

**Character 533: Jugal, secondary fossa for pneumatic recess, position relative to recess:**

0: ventral

1: dorsal
Note: Those taxa without a jugal recess (e.g., most ornithomimosaurs) are here scored as inapplicable (“?”) for this character.

**Character 534: Jugal, suture with lacrimal, angle of the posterior half of the contact**

0: low

1: steep

**Character 535: Jugal, fossa on lateral surface of postorbital ramus, depth inset into bone:**

0: shallow

1: deep

**Character 536: Jugal, articulation with postorbital, form of ventral extremity of suture:**

0: tapering scarf joint

1: interlocking notch for postorbital

**Character 537: Jugal, articulation with postorbital, extent of scarf joint on lateral surface of postorbital ramus:**

0: limited, occupies less than 50% of anteroposterior length of the process

1: extensive, occupies approximately 50-75% of the anteroposterior length of the process

**Character 538: Jugal, articulation with postorbital, braced by a pronounced ridge on the lateral surface of the postorbital ramus, which borders the postorbital posteriorly:**

0: no

1: yes

**Character 539: Jugal, postorbital ramus, orientation relative to ventral margin of jugal:**

0: approximately perpendicular

1: posterodorsal (obtuse angle between the long axis of the process and the ventral margin)

**Character 540: Jugal, cornual process: (ORDERED)**

0: absent

1: present

2: present and distinctive (mediolaterally wide and heavily rugose)

**Character 541: Jugal, dorsal prong of quadratojugal ramus, slope in lateral view:**

0: horizontal

1: posterodorsal

**Character 542: Jugal, ventral prong of quadratojugal ramus, slope of joint surface in lateral view:**

0: approximately anteroposteriorly oriented, angled less than 45 degrees from horizontal

1: angled anterodorsally at greater than 45 degrees from horizontal

**Character 543: Jugal, shape of orbital margin:**

0: weakly concave, approximately level with lacrimal-jugal suture

1: U-shaped, extends ventral to lacrimal-jugal suture

**Character 544: Jugal, raised rim on the lateral surface, paralleling the ventral margin of the bone and anteriorly confluent with the antorbital fossa rim of the maxilla:**

0: absent

1: present

**Character 545: Postorbital, cornual process: (ORDERED)**

0: absent

1: limited to rugose rim at posterodorsal corner of orbit

2: present as a rugose, convex boss

**Character 546: Postorbital, cornual process, position:**

0: separated from dorsal margin of postorbital by a smooth, convex region

1: approaches or extends past dorsal margin of bone

Note: Those taxa without a cornual process are scored as inapplicable (“?”).

**Character 547: Postorbital, cornual process, position:**

0: located at orbital margin

1: located posterodorsal to orbital margin

Note: Those taxa without a cornual process are scored as inapplicable (“?”).

**Character 548: Postorbital, squamosal ramus, form of posterodorsal margin:**

0: uninterrupted convex arc

1: emarginated by squamosal (discrete concave notch within the margin)

**Character 549: Postorbital, squamosal ramus, extent relative to posterior margin of lateral temporal fenestra:**

0: reaches or extends posterior to

1: terminates anterior to

**Character 550: Postorbital, suborbital process, position:**

0: at ventral end of ventral process

1: flange-like, separated from ventral tip of the ventral process by a notch

Note: Taxa without a suborbital process are here scored as inapplicable (“?”).

**Character 551: Postorbital, anterior ramus, form:**

0: short and stout, long axis is approximately half the length of the ventral ramus and the thickness at the base is approximately the same as the thickness of the midpoint of the ventral ramus

1: long and slender, long axis is greater than 60% of the length of the ventral ramus and the thickness at the base is approximately half that of the midpoint of the ventral ramus

**Character 552: Postorbital, ventral ramus, anteroposterior width at midpoint: (UNORDERED)**

0: approximately the same width as ventral ramus of the lacrimal

1: substantially wider than ventral ramus of lacrimal

2: substantially narrower than ventral ramus of lacrimal

**Character 553: Squamosal, lateral ridge delimiting supratemporal fossa, form:**

0: ridge unpronounced or undivided

1: divided

**Character 554: Squamosal, supratemporal fossa, surface morphology:**

0: flat or concave

1: convex

**Character 555: Squamosal, quadratojugal process, morphology of anterior tip in those taxa with horizontal processes:**

0: tapered point

1: squared off

**Character 556: Squamosal, quadratojugal process, flange that is covered laterally by the quadratojugal, dorsoventral depth of entire process compared to portion of process that is exposed in lateral view when in articulation with quadratojugal: (ORDERED)**

0: flange absent

1: thinner

2: substantially thicker
**Character 557: Squamosal, pneumaticity: posterior process, inflated by squamosal recess: (ORDERED)**

0: absent

1: present as a deep, concave depression on the ventral surface of the main body

2: present as a deep, concave depression on the ventral surface of the main body, and extending posteriorly to inflate the squamosal posterior process

Note: This is a modified version of the original character in Brusatte et al. (2010a, character 95) which includes a second derived state referring to squamosal pneumaticity in general. This character is ordered, as state 2 is a special condition of state 1 in which the pneumatic recess that invades the ventral surface of the main body of the squamosal also extends posteriorly into the posterior process of the squamosal.

**Character 558: Squamosal, posterior process, length of the long axis:**

0: long, approximately 1/3-1/2 length of quadratojugal process

1: short, approximately 1/6 length of quadratojugal process

**Character 559: Squamosal, anterior process, flange that extends dorsal to the postorbital posterior process:**

0: absent

1: present

**Character 560: Quadratojugal, dorsal process, ridge along anterior margin of lateral surface: (ORDERED)**

0: absent

1: present, subtle and fades in strength dorsally

2: present, robust and extends to the dorsal margin of the bone

**Character 561: Quadratojugal, form of jugal articulation, dorsal prong of posterior process of jugal approaching the base of the quadratojugal (the corner where the anterior and dorsal processes of the quadratojugal meet):**

0: absent

1: present

**Character 562: Quadratojugal, anterior process for articulation with jugal, form of anterior region: (UNORDERED)**

0: tapered

1: rounded

2: squared off or double pronged

**Character 563: Quadratojugal, anterior process, extent related to anterior margin of lateral temporal fenestra:**

0: terminates posterior to fenestra margin

1: level with or anterior to fenestra margin

**Character 564: Quadratojugal, curvature of bone:**

0: mediolaterally compressed and flat

1: posterior region flexed so that it curves posteriorly, thus delimiting the lateral edge of a deep pocket that borders the quadrate foramen laterally in posterior view

**Character 565: Quadratojugal, posterior process, length and orientation:**

0: short, oriented mostly laterally

1: elongate, wraps onto the posterior surface of the quadrate cotyles

**Character 566: Quadrate foramen, size:**

0: small, long axis approximately 10% of the dorsoventral depth of the quadrate shaft

1: large, long axis greater than 20% of the dorsoventral depth of the quadrate shaft

**Character 567: Quadrate, form of pneumatization, deep recess on the anterior surface where the psterygoid wing and condyles meet:**

0: absent

1: present

**Character 568: Quadrate, condyles, position relative to occipital condyle when skull is in articulation:**

0: aligned (i.e., quadrate condyles approximately ventral to occipital condyle, or slightly anterior to condyle)

1: completely posterior

**Character 569: Quadrate, quadratojugal articulation, extent on lateral surface of lateral condyle:**

0: limited, occupies only part of the surface

1: extensive, covers entire lateral surface and extends dorsally to partially enclose quadrate foramen laterally

**Character 570: Quadrate, articular surface for quadratojugal on quadrate lateral condyle, orientation of medial margin as seen in posterior view where quadratojugal wraps around quadrate:**

0: vertical or dorsomedial

1: dorsolateral

**Character 571: Prefrontal, contacts nasal:**

0: yes

1: no, excluded by frontal-lacrimal contact

**Character 572: Prefrontal, ventral process, extent:**

0: large, extends 1/2-1/4 of the way down the ventral ramus of the lacrimal to make an extensive contribution to the preorbital bar

1: reduced or absent, ventral process is a thin flange that is continuous with the crista cranii of the frontal, and does not extend more than approximately ¼ of the length of the preorbital bar

**Character 573: Frontal, shape (in those taxa with frontals that narrow anteriorly as a wedge between the nasals):**

0: triangular

1: posterior end expanded into a rectangular shape, with a small anterior triangle

Note: Taxa with frontals that do not narrow anteriorly between the nasals (scored in character 41) are scored here as inapplicable (“?”).

**Character 574: Frontal, size of single frontal, ratio of anteroposterior length of exposed portion on skull roof to mediolateral width at midpoint:**

0: greater than 2.0 (usually greater than 2.5)

1: less than 2.0

**Character 575: Frontal, supratemporal fossa, medial extension:**

0: fossa restricted to posterolateral corner of frontal

1: meets opposing fossa at the midline

**Character 576: Frontal, sagittal crest: (UNORDERED)**

0: absent or subtle, only discernable as a slight midline bulge

1: present and pronounced (dorsoventrally tall), single structure

2: present and pronounced (dorsoventrally tall), paired structure

Note: This charater is not equivalent to a parietal sagittal crest. Rather, it refers specifically to raised crests on the dorsal surface of the frontal, which may or may not be continuous with a sagittal crest on the parietal.

**Character 577: Frontal, sagittal crest, anteroposterior length:**

0: short, less than 15% length of the frontal

1: extensive, approximately 25% of the length of the frontal

Note: Those taxa without a frontal sagittal crest are scored as inapplicable (“?”).

**Character 578: Frontal, postorbital suture: (ORDERED)**

0: dorsoventrally shallow and undifferentiated

1: dorsoventrally shallow (approximately 6 times longer than deep) and differentiated into a vertical region anteriorly and a horizontal region posteriorly

2: dorsoventraly deep (approximately twice as long as deep) and subtly differentiated into vertical and horizontal regions

**Character 579: Frontal, contribution to orbital rim: (ORDERED)**

0: extensive

1: present but limited to a small notch

2: excluded by postorbital-lacrimal contact in large specimens

3: exclueed by postorbital-lacrimal articulation and oval “palpebral” ossification

**Character 580: Parietal-frontal suture, form:**

0: transversely smooth

1: tab-like wedge from parietal extends anteriorly to overlie frontal on mindline

**Character 581: Parietal, sagittal crest, form:**

0: comprised of two parallel crests

1: comprised of a single midline crest

Note: Those taxa without a parietal sagittal crest are here scored as inapplicable (“?”).

**Character 582: Parietal, skull table between supratemporal fossae, width:**

0: broad, more than 10% of the mediolateral width of the fossa

1: extremely reduced, sagittal crest or crests (if present) pinched between opposing fossae

**Character 583: Parietal, sagittal crest, dorsoventral depth:**

0: consistent across length of crest

1: peaked anteriorly at frontal-parietal suture

Note: Those taxa without a parietal sagittal crest are here scored as inapplicable (“?”).

**Character 584: Parietal, nuchal crest, dorsoventral depth:**

0: as low as or lower than dorsal surface of the interorbital region

1: extends higher than the dorsal surface of the interorbital region

**Character 585: Vomer, shape of anterior end:**

0: lanceolate (lateral margins parallel-sided)

1: expanded into a diamond

**Character 586: Ectopterygoid, extent of internal recess:**

0: does not inflate body of the bone and the pterygoid process

1: inflates body of the bone and the pterygoid process

**Character 587: Ectopterygoid, jugal process, external pneumatic foramina leading into ectopterygoid recess:**

0: absent

1: present

**Character 588: Ectopterygoid, jugal process, pneumaticity:**

0: is not inflated by the internal recess

1: is visibly inflated by the internal recess

**Character 589: Ectopterygoid, external opening of pneumatic recess, shape:**

0: thin ovoid slot

1: large, round or triangular

**Character 590: Ectopterygoid, surface posteriorly adjacent to external opening of pneumatic recess, form:**

0: flat, recess grade smoothly into the floor of the lateral temporal fenestra (=subtemporal fenestra)

1: lip, recess separated from lateral temporal fenestra (=subtemporal fenestra)

**Character 591: Palatine, vomeropterygoid process, ratio of anteroposterior length of dorsal margin to length of greatest constriction of process neck:**

0: greater than 2.0

1: less than 2.0
**Character 592: Palatine, vomeropterygoid process, orientation of neck:**

0: inclined anterodorsally

1: vertical

**Character 593: Palatine, pneumaticity:**

0: absent

1: present

**Character 594: Palatine, pneumatic recess, number of external pneumatic openings:**

0: one

1: two

**Character 595: Palatine, primary external opening of palatine recess, location of posterior margin:**

0: level with or extends posterior to posterior margin of the vomeropterygoid process neck

1: located far anterior to posterior margin of the vomeropterygoid process neck

**Character 596: Palatine, primary opening of palatine recess, location of anterior margin:**

0: level with or extends posterior to anterior margin of the vomeropterygoid process neck

1: located far anterior to anterior margin of the vomeropterygoid process neck

**Character 597: Palatine, jugal process, location of contact surface for lacrimal:**

0: posterior (“distal”), separated from opening of palatine recess by wide margin

1: anterior (“proximal’), closely approaches opening of palatine recess

**Character 598: Palatine, maxillary process, form of maxillary articulation:**

0: flat

1: deeply excavated as a slot, demarcated dorsally by a pronounced lip of bone

**Character 599: Palatine, extension of pneumatic recess into jugal process:**

0: no

1: yes, process visibly inflated

**Character 600: Palatine, maxillary articulation, form:**

0: maxilla abuts lateral surface of maxillary process and anterior region of jugal process

1: contact reinforced by a “brace” at the anteroventral corner of the jugal process, which sits within internal antorbital fossa

**Character 601: Palatine, morphology of maxillary articulation brace:**

0: projects ventrally due to a jugal process that extends further ventrally than the maxillary process, such that there is a discrete corner between the two processes in lateral view

1: projects laterally, with no discrete corner between the smoothly confluent jugal and maxillary processes in lateral view

**Character 602: Internal choana, shape:**

0: anteroposteriorly elongate oval

1: nearly circular

**Character 603: Suborbital fenestra, shape:**

0: anteroposteriorly elongate oval

1: nearly circular

**Character 604: Braincase, orientation of occipital surface:**

0: faces posteriorly

1: faces posteroventrally

**Character 605: Supraoccipital, contribution to dorsal rim of foramen magnum (ORDERED)**

0: forms entire rim

1: makes limited contribution to rim via triangular ventral process

2: completely excluded from rim

**Character 606: Supraoccipital, form of dorsal margin**

0: smoothly convex and undivided

1: divided into two processes (“forked”)

**Character 607: Exoccipital-opisthotic, paroccipital process, ventral flange at distal end:**

0: absent

1: present

**Character 608: Exoccipital-opisthotic, paroccipital processes, deep fossa on posterior surface dorsolateral to the foramen magnum:**

0: present

1: absent

**Character 609: Exoccipital-opisthotic, crista tuberalis (=metotic strut), extent in posterior view:**

0: limited, mediolateral width across opposing cristae less than one half the dorsoventral depth of the braincase from the dorsal tip of the supraoccipital to the ventral tip of the basal tubera

1: extensive, width greater than one half braincase depth

**Character 610: Basioccipital, basal tubera, dorsoventral depth:**

0: less than depth of occipital condyle

1: greater than depth of occipital condyle

**Character 611: Basioccipital, basal tubera, concave notch ventrally between opposing tubera, dorsoventral depth:**

0: shallow, less than 40% depth of tubera

1: deep, approximately 50% depth of tubera

**Character 612: Basioccipital, subcondylar recess, depth of pneumatic fossae on posterior surface of basal tubera:**

0: absent or shallow

1: deep

**Character 613: Basisphenoid, basisphenoid recess, orientation of central axis: (ORDERED)**

0: vertical, recess obscured in posterior view

1: posteroventral, recess partially visible in posterior view

2: extremely posteroventral, recess compressed anteroposteirorly and widely visible in posterior view, and basipterygoid processes located beneath the basal tubera

**Character 614: Basisphenoid, basisphenoid recess, inflation of the ceiling of the recess:**

0: absent

1: present

**Character 615: Basisphenoid, basisphenoid recess, shape in ventral view:**

0: funnel-like, expands in mediolateral width posteriorly

1: ovoid or circular, no posterior expansion

**Character 616: Basisphenoid, shape of basicranium (rectangle defined by positions of both basal tubera and both basipterygoid processes):**

0: anteroposteriorly longer than mediolaterally wide

1: wider than long

**Character 617: Parasphenoid, shape of rostrum:**

0: anteroposteriorly expanded, ventral margin is a smooth concave arch

1: dorsoventrally expanded, ventral margin is nearly vertical posteriorly and then abruptly transitions to horizontal trend anteriorly

**Character 618: Laterosphenoid, antotic crest separating lateral wall of braincase from orbital and temporal spaces:**

0: absent or indistinct

1: present, robust and rugose

**Character 619: Laterosphenoid, antotic crest, form:**

0: single structure

1: bifurcates ventrally

**Character 620: Laterosphenoid, fossa on lateral surface that houses head of epipterygoid:**

0: absent or shallow

1: present, deep and rugose

**Character 621: Mandibular ramus, dorsoventral depth of dentary at level of dentary-surangular contact on the dorsal margin of the lower jaw:**

0: less than 18% of the total anteroposterior length of the lower jaw

1: greater than 18% of the total anteroposterior length of the lower jaw

**Character 622: External mandibular fenestra, dorsoventral depth relative to depth of mandible at midpoint of fenestra: (ORDERED)**

0: greater than 25% depth of mandible

1: less than 25% depth of mandible

2: absent

**Character 623: Lower jaw, articulation, glenoid position relative to level of alveolar margin of dentary: (UNORDERED)**

0: approximately level with

1: dorsal to

2: strongly ventral to

**Character 624: Dentary, position of the transition point between the ventral and anterior margins of the bone in lateral view:**

0: below alveoli 1-3, anterior margin of bone rounded (or in some cases nearly straight)

1: below alveolus 4 (or more posteriorly), anterior margin nearly straight and projects posteroventrally

**Character 625: Dentary, ventrally projecting rugose process (“chin”) where the anterior and ventral margins of the dentary meet:**

0: absent

1: present, visible as a pointed projection in lateral view and convex in medial view, braces dentary symphysis

**Character 626: Dentary, symphysis, texture:**

0: generally smooth

1: strongly rugose and beveled, with interlocking ridges and convexities for articulation with the opposing symphysis

**Character 627: Dentary, articular surface for splenial along ventral region of dentary ramus below the Meckelian fossa, form:**

0: dorsoventrally shallow and smooth

1: dorsoventrally deep (nearly as deep as anterior depth of fossa) and rugose

**Character 628: Dentary, anterior alveoli, size in comparison to alveoli in middle of tooth row: (ORDERED)**

0: approximately same size

1: first two alveoli substantially smaller

2: first alveolus substantially smaller

Note: This character is ordered here, because we hypothesize that state 2 (which is seen only in a small subset of derived tyrannosaurids: *Tarbosaurus* and *Tyrannosaurus*) is a special condition of state 1 (which is present in all score-able tyrannosauroids except for the derived subset).

**Character 629: Dentary, dorsal margin of bone in lateral view, profile: (UNORDERED)**

0: straight

1: strongly concave

2: strongly convex

**Character 630: Dentary, Meckelian groove, form:**

0: dorsoventrally deep and shallowly inset into medial surface of bone

1: dorsoventrally shallow and deeply inset into bone, groove appears as a thin, sharp structure

**Character 631: Surangular, surangular shelf on lateral surface, form: (ORDERED)**

0: low ridge or absent

1: prominent ridge that is offset laterally from the bone but dorsoventrally thin

2: prominent shelf that is dorsoventrally deep

Note: The “low ridge” and “absent” conditions are considered primarily homologous here, because a very low ridge may appear absent on a specimen due to poor preservation. Taxa that genuinely appear to lack a ridge (e.g., *Shuvuuia*) are scored as inapplicable (“?”) for the following characters related to the form and the position of the ridge.

**Character 632: Surangular, surangular shelf on lateral surface, position and form: (ORDERED)**

0: placed far dorsal to posterior surangular foramen

1: foramen abuts shelf but shelf projects laterally and does not overhang foramen

2: shelf projects ventrolaterally to overhang foramen

**Character 633: Surangular, surangular shelf on lateral surface, orientation relative to the long axis of the lower jaw: (UNORDERED)**

0: anterodorsal

1: anteroventral

2: straight anteroposteriorly

**Character 634: Surangular, pneumatic fossa posterodorsal to posterior surangular foramen:**

0: absent

1: present

**Character 635: Surangular, adductor muscle attachment site dorsal to surangular shelf, orientation: (ORDERED)**

0: faces primarily dorsally

1: faces almost equally dorsally and laterally

2: faces primarily laterally

**Character 636: Surangular, triangular fossa on the lateral surface of the surangular shelf immediately anteroventral to glenoid:**

0: absent

1: present

**Character 637: Surangular, fossa on the lateral surface of the bone immediately ventral to, and separated from, the glenoid:**

0: absent

1: present

**Character 638: Surangular, anteroposterior length of anterior flange (region anterior to anterior margin of external mandibular fenestra) compared to overall length of surangular:**

0: less than 30%

1: greater than 30%

**Character 639: Angular, ventral margin, form:**

0: smoothly convex

1: anterior region “flexed” relative to posterior region, such that there is a discrete step between them

**Character 640: Articular, mediolateral width of jaw muscle attachment site:**

0: less than width of glenoid for articulation with quadrate

1: approximateily equal to width of glenoid

**Character 641: Articular, smooth non-articular region between glenoid and attachment site for depressor mandibular muscles:**

0: present

1: absent

**Character 642: Splenial, anterior mylohoid foramen, shape and size: (ORDERED)**

0: small circular or ovoid opening, or absent

1: large, anteroposteriorly ovoid shape

2: extremely large, approximately as deep dorsoventrally as the anterior process of the splenial

Note: The small and absent conditions are here treated as a single character state, because poor preservation often makes a very small foramen appear absent.

**Character 643: Splenial, dorsal region overlapped medially by prearticular:**

0: absent

1: present

**Character 644: Prearticular, ventral bar, series of ridges on lateral surface to strengthen articulation with angular:**

0: absent

1: present

**Character 645: Supradentary ossification, shape:**

0: elongate, shallow strip

1: deep, crescentic shape

**Character 646: Supradentary and coronoid ossifications, form of contat at their zone of fusion:**

0: ossifications smoothly confluent

1: ossifications offset by a concave notch

**Character 647: Premaxillary tooth crown 4, apicobasal height relative to largest maxillary crown:**

0: subequal

1: approximately 50%

**Character 648: Premaxillary teeth, median vertical ridge on lingual surface: (ORDERED)**

0: absent

1: present as a subtle structure in anterior (mesial) premaxillary teeth

2: present as pronounced structure in all premaxillary teeth

**Character 649: Premaxillary teeth, curvature of distal (posterior) teeth:**

0: recurved

1: straight

**Character 650: Maxillary teeth, number:**

0: 13 or more

1: less than 13 (in the largest adult specimens when growth series are known)

**Character 651: Maxillary and dentary teeth, form: (ORDERED)**

0: ziphodont, transverse width of base less than 60% of mesiodistal length

1: incrassate, width greater than 60% of length

2: incrassate, width nearly equal to length

Note: Those taxa with conical or leaf-like teeth are scored as inapplicable (“?”) for this character (e.g., therizinosauroids, most alvarezsauroids).

**Character 652: Axis and postaxial cervicals, anteroposterior length of centrum compared to dorsoventral height of posterior centrum face:**

0: greater

1: less than or equal to

**Character 653: Axis, pneumatic foramen (pleurocoel), position:**

0: near midheight of entrum

1: dorsally located, directly underneath neurocentral suture and directly posterior to diapophysis

**Character 654: Axis, pneumatic foramen (pleurocoel), extent of surrounding fossa:**

0: limited to margins of foramen

1: extensive, occupies most of lateral surface of centrum

**Character 655: Axis, ridge on ventral surface of centrum:**

0: absent

1: present

**Character 656: Axis, pneumatic foramina and fossae on each side of the anterior ridge on the neural spine:**

0: absent

1: present

**Character 657: Axis, neural spine, texture of dorsal region of anterior surface:**

0: generally smooth or with subtle texture

1: highly rugose, with series of grooves, ridges, and eminences

**Character 658: Axis, dorsal region of neural spine, number of projections on “crown” region:**

0: two lateral projections, dorsal surface of spine smoothly concave

1: two lateral projections and one dorsal projection on the midline

Note: Only those taxa scored for a mediolaterally broad (“transversely flared”) neural spine for character 92 are relevant to this charater. All taxa with a mediolaterally compressed neural spine are scored as inapplicable (“?”).

**Character 659: Axis, supradiapophyseal fossa (fossa posterodorsal to diapophysis), form:**

0: absent or shallow

1: deeply excavated and funnel-like

**Character 660: Cervical vertebrae, neural spines in middle-posterior cervicals, dorsoventral height:**

0: substantially shorter than height of posterior centrum face

1: approximately same length as or longer than height of posterior centrum face

**Character 661: Cervical vertebrae, morphology of posterior centrodiapophyseal laminae in anterior-middle cervicals:**

0: absent or present as a weak ridge

1: present as a thick, laterally offset lamina that demarcates a deep infradiapophyseal fossa anteriorly

**Character 662: Cervical vertebrae, hypapophysis on anterior region of ventral surface:**

0: absent

1: present

**Character 663: Cervical vertebrae, position of prezygapophysis in middle cervicals:**

0: slightly overhangs centrum laterally

1: strongly overhangs centrum laterally, entire prezygapophyseal facet placed lateral to centrum

**Character 664: Cervical vertebrae, orientation of posterior centrodiapophyseal lamina in anterior-middle cervicals:**

0: projects posteroventrally, infrapostzygapophyseal fossa located primarily posterior to lamina

1: nearly horizontal, fossa located primarily dorsal to lamina

**Character 665: Cervical and dorsal vertebrae, rugose ligament attachment scars in pre- and postspinal fossae: (ORDERED)**

0: absent or weakly developed

1: present as prominent, rectangular flanges that extend outside of the fossae and are visible in posterior view, but on the dorsal vertebrae only

2: prominent in dorsals and cervicals

**Character 666: Dorsal vertebrae, neural spine, level of posterior termination:**

0: at approximately the same level as the posterior centrum face

1: far posterior to the posterior centrum face

**Character 667: Dorsal vertebrae, anteroposterior length of middle-posterior dorsal centra compared to dorsoventral height of posterior centrum face:**

0: greater

1: less than or equal

**Character 668: Dorsal vertebrae, middle-posterior dorsals, position of postzygapophysis relative to prezygapophysis:**

0: at same level

1: elevated dorsally

**Character 669: Dorsal vertebrae, middle-posterior dorsals, form of anterior and posterior centrodiapophyseal laminae: (ORDERED)**

0: discrete laminae absent, or laminae present but do not demarcate a deep infradiapophyseal fossa between them

1: present and make contact on ventral surface of transverse process, demarcating a triangular infradiapophyseal fossa

2: present and do not make contact but roughly parallel each other, infraprezygapophyseal and infradiapophyseal fossa merged into a single fossa

**Character 670: Sacral vertebrae, fenestrae between fused neural spines: (ORDERED)**

0: neural spines unfused

1: spines fused but fenestrae absent

2: spines fused and fenestrae present

**Character 671: Sacral ribs, position of central ribs on sacrum:**

0: span two sacrals

1: limited to a single sacral

**Character 672: Sacral ribs, position of rib attachment for central ribs on individual sacrals:**

0: span centrum and neural arch

1: limited to neural arch only

**Character 673: Sacral vertebra five, position of ventral margin of posterior articular face in lateral view:**

0: at same level as ventral margin of anterior articular face

1: positioned ventral to ventral margin of anterior articular face

**Character 674: Sacral vertebrae, form of hyposphene in posteriormost sacral:**

0: absent or present as a single midline structure

1: present and comprised of two parallel-sided sheets

**Character 675: Caudal vertebrae, anterior caudals, position of base of neural spine:**

0: anterior to posterior surface of centrum

1: level with or posterior to posterior surface of centrum

**Character 676: Caudal vertebrae, anterior caudals, shape of transverse processes in dorsal view:**

0: rectangular, with parallel anterior and posterior sides, or slightly ovoid with a gradual expansion in width distally

1: distal end expanded into a spatulate bulb

**Character 677: Caudal vertebrae, anterior caudals, two laminae linking prezygapophysis and transverse process, between which is a deep, triangular fossa:**

0: absent

1: present

**Character 678: Scapula, angle between posterior margin of glenoid and dorsal margin of blade:**

0: greater than 90 degrees

1: approximately 90 degrees

**Character 679: Scapula, acromion, dorsoventral depth:**

0: less than 3.0 times minimum dorsoventral depth of blade

1: greater than 3.0 times minimum dorsoventral depth of blade

**Character 680: Scapula, ratio of anteroposterior length of bone to minimum dorsoventral depth of blade:**

0: less than 10.0

1: greater than 10.0

**Character 681: Scapula and coracoid, glenoid, position relative to posteroventral margin of blade:**

0: offset posteroventrally (by a distance equivalent to the width of the neck of the blade to ½ of the width of neck of the blade)

1: offset only slightly posteroventrally (less than 50% the width of the neck of the blade)

**Character 682: Coracoid, anteroposterior length at midpoint:**

0: approximately 100-150% of the length of the scapular acromion at midheight

1: 200% or greater than the length of the scapular acromion at midheight

**Character 683: Coracoid, coracoid foramen:**

0: present

1: absent or extremely small

**Character 684: Humerus, apex of deltopectoral crest, location from proximal end: (ORDERED)**

0: 35-50% of the length of the humerus

1: 25-35% of the length of the humerus

2: less than 25% of the length of the humerus

**Character 685: Humerus, additional muscle attachment tubera at the corner of the anterior and lateral surfaces distal to the deltopectoral crest:**

0: absent

1: present

**Character 686: Humerus, concave notch between external tuberosity and deltopectoral crest:**

0: present, two structures clearly separated

1: absent, two structures smoothly confluent

**Character 687: Humerus, form of distal condyles:**

0: lateral and medial condyles expanded equally (offset from shaft in anterior or posterior view is approximately equal)

1: medial condyle expanded further medially than the lateral condyle is laterally

**Character 688: Ulna, shaft axis, form:**

0: bowed

1: straight

**Character 689: Principal distal carpal, shape:**

0: semilunate in lateral view with trochlear proximal surface

1: discoid with flat proximal surface

**Character 690: Metacarpal I, medial distal condyle, form:**

0: well formed and large

1: rudimentary

**Character 691: Metacarpal I, medial margin, shape in proximal view:**

0: concave

1: smoothly convex or straight

**Character 692: Metacarpals, metacarpal II, mediolateral width at midpoint compared to midpoint width of metacarpal I:**

0: equal to or narrower than

1: more robust than

**Character 693: Manual phalanx II-1, length compared to that of metacarpal 1:**

0: longer

1: subequal to

**Character 694: Ilium, anteroposterior length compared to length of femur: (ORDERED)**

0: 70-85%

1: 95-105%

2: 105-115% (or greater, in some birds)

**Character 695: Ilium, dorsal margin of blade, position relative to sacral neural spines: (UNORDERED)**

0: separated by a gap

1: lies against neural spines and opposing iliac blaces may make contact above neural spines in some individuals

2: separated by a wide gap

Note: This is a modified version of the Brusatte et al. (2010a) character, which adds a second derived state (“separated by a wide gap”) to refer to the condition of some derived therizinosauroids, as used by Zanno et al. (2009, character ZCD 306).

**Character 696: Ilium, supraacetabular crest, maximum lateral projection relative to ischial peduncle:**

0: significantly greater

1: subequal

**Character 697: Ilium, supraacetabular crest, extent on pubic peduncle:**

0: extensive, extends along most or all of the edge of the peduncle

1: limited, discretely offset from acetabular edge of pubic peduncle

**Character 698: Ilium, pubic and ischial peduncles, anteroposterior lengths at dorsal base: (ORDERED)**

0: pubic peduncle substantially longer than ischial peduncle

1: both peduncles approximately the same length

2: ischial peduncle longer than pubic peduncle

**Character 699: Ilium, ventral margin of postacetabular process, shape:**

0: straight to slightly convex

1: hightly convex, forming a discrete “lobe”-like flange

**Character 700: Ilium, dorsal margin, shape:**

0: smoothly convex or straight across entire length

1: convex anteriorly and straightens out posteriorly

Note: In dromaeosaurids such as *Unenlagia*, which possess a separate posterior tab-like process of the postacetabular process, this character refers to the shape of the remainder of the ilium (i.e., the entirety of the bone except the tab-like process). Therefore, these taxa are scored for state 0.

**Character 701: Ilium, ratio of anteroposterior length to dorsoventral depth above acetabulum:**

0: greater than 3.0, ilium is long and low

1: less than 2.8, ilium is subovoid in shape

**Character 702: Pubis, pubic tubercle: (ORDERED)**

0: absent

1: present as a convexity on the anterior margin of the pubis

2: present as a rugose flange that is discretely offset from the anterior margin of the pubis and is bordered posteriorly by heavy rugosities on the lateral surface on the obturator region of the pubis

**Character 703: Pubis, pubic tubercle, position:**

0: distally positioned, located ventral to the level of the obturator notch

1: proximally positioned, located level with or dorsal to the obturator notch

Note: All taxa without a pubic tubercle are here scored as inapplicable (“?”).

**Character 704: Pubis, pubic boot, anteroposterior length relative to total long axis length of pubis:**

0: less than 60%

1: greater than 60%

**Character 705: Pubis, pubic boot, position of anterior process relative to posterior process:**

0: displaced dorsally, resulting in a highly convex ventral margin of the boot

1: placed at the same level, ventral margin of the boot essentially straight

Note: Taxa without a discrete anterior process (e.g., compsognathids) are here scored as inapplicable (“?”).

**Character 706: Pubis, anteroposterior expansion of proximal obturator plate region relative to the anterior edge of the pubis shaft at its midpoint:**

0: less than twice the anteroposterior thickness of the shaft at its midpoint

1: greater than twice the anteroposterior thickness of the shaft at its midpoint

**Character 707: Pubis, obturator notch, form:**

0: discrete structure, demarcated ventrally by extensive obturator flange

1: essentially absent, no ventral flange

Note: The enclosed obturator foramen of *Mirischia* is here scored as equivalent to state 0. The presence of a completely enclosed foramen is autapomorphic of *Mirischia* among coelurosaurs.

**Character 708: Ischium, position of medial apron:**

0: along posterior margin of shaft

1: along anterior margin of shaft

**Character 709: Femur, circular scar on posterior surface of shaft distal to fourth trochanter, position:**

0: absent, low, or positioned approximately centrally on the shaft

1: abuts medial edge of shaft

**Character 710: Femur, lesser trochanter, height relative to greater trochanter:**

0: shorter, terminates further distally

1: subequal or slightly taller, the two structures extend to approximately the same level proximally

Note: This is equivalent to Zanno et al. (2009: character ZCD 325).

**Character 711: Femur, proximal margin in anterior view: (ORDERED)**

0: approximately straight and perpendicular to the long axis of the shaft

1: approximately straight and oriented at an obtuse angle to the long axis of the shaft (=dorsally or proximally inclined head)

2: concave and oriented at an obtuse angle to the long axis of the shaft, due to head that is proximally inclined and a greater trochanter that is elevated substantially relative to the central portion of the proximal surface of the femur

Note: Characters relating to the inclination of the femoral head are often used in theropod phylogenetic datasets, but it is often difficult to develop a standard system of measurement for quantifying the orientation of the head. We use the character as defined here, which concerns the angle between the long axis of the shaft and the long axis of the proximal end, as measured in anterior view (note that many taxa with obtuse angles between the head and shaft in anterior view, such as *Tyrannosaurus*, appear to have perpendicular angles when measured in posterior view). Usually, taxa with an obtuse angle between head and shaft (i.e., a dorsally inclined femoral head) have a proximomedially projecting proximal surface of the femur when seen in anterior view, and this may indicate the presence of an inclined head in specimens that do not preserve the shaft. Furthermore, taxa with an obtuse angle between head and shaft (a dorsally inclined femoral head) possess a head that is elevated proximally relative to the greater trochanter, which is another means for recognizing the inclined condition in specimens that do not preserve the shaft. We also recognize a second derived state, encoded by Zanno et al. (2009: character oZCD 339) and Brusatte et al. (2010a: character 285), referring to the deeply concave proximal margins of the derived tyrannosaurids *Tyrannosaurus* and *Tarbosaurus* and some derived therizinosauroids, which have dorsally inclined heads but also greater trochanters that are raised substantially relative to the remainder of the proximal femur (except for the head), giving the proximal margin a deeply concave profile in posterior view. This character is equivalent to Zanno et al. (2009, character oZCD 320), which refers to the distinction between a medially (“perpendicular to shaft”) and dorsally inclined femoral head. Brusatte et al. (2012a) stated that *Tanycolagreus* possesses a dorsally inclined (elevated) femoral head, but we here note that this is not correct and that this taxon possesses a head that is perpendicular to the shaft (state 0).

**Character 712: Femur, trochanteric fossa on the posterior surface of the head, lateral to the ligament sulcus (for the capital ligament), form: (ORDERED)**

0: absent or shallow

1: deep fossa

2: deep, extensive triangular depression that covers most of the posterior surface of the femur proximally and is demarcated medially and ventrally by a pronounced, curving, swollen ridge

**Character 713: Femur, fourth trochanter, position, measurement from proximal margin of head to distal termination of trochanter relative to total length of the femur:**

0: 40% or less

1: greater than 40%

**Character 714: Femur, lateral condyle, shape in distal view:**

0: circular or ovoid

1: ovoid, but with an anterior bulge that is slightly separated from the remainder of the condyle

**Character 715: Femur, extensor groove on anterior surface of distal end, form: (ORDERED)**

0: absent or extremely shallow, anterior surface flat between the condyles in distal view (extensor groove may be present but does not manifest itself as a groove on the anterior margin in distal view)

1: groove present but shallow, expressed as a broad concave margin in distal view but present as an extensive depression on the anterior surface of the femur

2: groove present and deep, expressed as a deep, U-shaped cleft in distal view and present as an extensive depression on the anterior surface of the femur

**Character 716: Femur, mesiodistal crest, form:**

0: single structure

1: bifurcates distally to enclose fossa on the medial surface of the medial condyle

**Character 717: Tibia, length relative to the femur:**

0: 1.05 or greater

1: less than 1.00

**Character 718: Tibia, lateral condyle of proximal end, anterior process:**

0: absent

1: present

**Character 719: Tibia, lateral malleolus, lateral extent:**

0: limited, mediolateral measure is less than 40% of mediolateral width of adjacent shaft

1: extensive, mediolateral measure greater than 40% of mediolateral width of adjacent shaft

**Character 720: Tibia, lateral malleolus, position relative to medial malleolus:**

0: extent to approximately the same level distally

1: lateral malleolus extends substantially further distally than medial malleolus

**Character 721: Fibula, iliofibularis tubercle, form:**

0: single crest

1: large, rugose, and formed by two crests separated by a depressed fossa (“bipartite” condition)

**Character 722: Astragalus, fossa on anterior surface of ascending process, form:**

0: shallow concavity that covers most of the ventral region of the ascending process

1: deep, triangular or ovoid fossa immediately above midpoint of condyles, set within a broad fossa that covers most of the ventral region of the ascending process

**Character 723: Pes, metatarsal III, form of medial surface in anterior or posterior view:**

0: straight or subtly convex

1: with medial convex expansion forming a bulge along the distal part of the shaft

Note: This character is equivalent to L. Xu et al. (2011: character 48), who utilized it as a character relevant to ornithomimosaurs.

**Character 724: Pes, metatarsal III, ventral nonarticular surface (on the flexor surface) immediately proximal to the distal condyles, form:**

0: concave

1: raised subtriangular platform

**Character 725: Pes, metatarsals II-IV, distal separation when in articulation:**

0: metatarsals closely appressed and distance between II-III and III-IV is approximately equal

1: distal ends of II and IV diverge from III, and distance between III-IV greater than that between II-III

**Character 726: Pes, metatarsal II, articular scar for metatarsal III on distal portion of lateral surface of shaft, form:**

0: subtle or absent

1: enlarged as a rugose fossa that occupies more than half of the proximodistal length of the shaft and expands in anteroposterior width distally

**Character 727: Pes, metatarsal II, lateral surface in proximal view, shape: (ORDERED)**

0: flat or weakly concave

1: moderately concave

2: strongly concave (deep concave notch is present)

Note: This is an expanded version of the original Brusatte et al. (2010a, character 304) character, which adds a new derived state to take into account the subtly concave condition in some ornithomimosaurs, which differs from the flat condition in outgroups and many basal coelurosaurs (e.g., *Ornitholestes*) and the deeply concave notched condition of derived tyrannosaurids.

**Character 728: Pes, metatarsal IV, distal end, ratio between anteroposterior long axis (measured from midpoint of condyles posteriorly to anterior surface of bone) and mediolateral width (measured at midpoint: (ORDERED)**

0: greater than 1.40, distal surface is elongate anteroposteriorly

1: between 1.40 and 1.20

2: less than 1.20, distal surface nearly square shaped with nearly flat anterior surface

**Character 729: Pes, proximal pedal phalanges of digits II and III, ratio of length to midshaft width:**

0: greater than 3.0

1: less than 3.0

**Character 730: Pes, pedal unguals, lip overhanging proximal articular surface dorsally (on extensor surface):**

0: present

1: absent or reduced to a subtle tuber

***Characters Relevant to Ornithomimosauria (from L. Xu et al. 2011):**

**Character 731: Maxilla, series of discrete foramina along ventral edge of lateral surface:**

0: present

1: absent

**Character 732: Lacrimal, prominence on lateral surface of bone:**

0: absent

1: present

**Character 733: Dentary, morphology of dorsal border in transverse cross section:**

0: rounded and lacks “cutting edge”

1: sharp with a “cutting edge”

**Character 734: Surangular, foramen on dorsal edge of bone dorsal to mandibular fenestra (anterior surangular foramen):**

0: present

1: absent

**Character 735: Neck, length compared to that of skull:**

0: less than twice skull length

1: greater than twice skull length

**Character 736: Coracoid, biceps tubercle, position:**

0: positioned close to base of posterior process (closer to coracoid-scapula suture than to anterior edge of coracoid)

1: positioned more anterior than the base of the posterior process (closer to anterior edge of coracoid than to coracoid-scapula suture)

**Character 737: Coracoid, position of infraglenoid buttress relative to that of posterior process when bone is seen in dorsal view:**

0: two structures extend to same level laterally

1: buttress offset laterally from posterior process

**Character 738: Metacarpal II, length compared to metacarpal III:**

0: shorter

1: equal to or longer

**Character 739: Manual digit I, length of phalanx I-1 compared to metacarpal II:**

0: shorter

1: longer

**Character 740: Manual unguals, flexor tubercles, position:**

0: near proximal end

1: distal to proximal end

Note: Those alvarezsauroids without discrete flexor tubercles are scored as inapplicable (“?”).

**Character 741: Pubis, shape of ventral margin of pubic boot:**

0: straight or slightly convex

1: strongly convex with ventral expansion

**Character 742: Pes, pedal digit I:**

0: present

1: absent

**Character 743: Pes, phalanx II-2, length:**

0: more than 60% of length of pedal phalanx II-1

1: less than 60% of length of pedal phalanx II-1

**Character 744: Pubis, angle between anterior process of pubic boot and shaft:**

0: greater than 90 degrees

1: approximately 90 degrees

**Character 745: Pubis, position of anterior edge of anterior process of pubic boot:**

0: approximately same level as anterior margin of pubic shaft

1: markedly anterior to the anterior margin of pubic shaft

**Character 746: Pes, pedal unguals, shape:**

0: curved in lateral view

1: straight

***Characters Relevant to Basal Coelurosauria (from Li et al. 2010):**

**Character 747: Premaxillary teeth, size compared to mesial (anterior) maxillary teeth:**

0: approximately same size, slightly smaller, or larger

1: considerably smaller

**Character 748: Ischium, form of articulation with ilium on proximal surface of iliac peduncle:**

0: approximately flat or slightly concave

1: deeply concave as a deep socket to receive a peg-like ischial peduncle of the ilium

Note: Some tyrannosauroids and ornithomimosaurs have been described as possessing an accessory peg-like structure on the distal edge of the ischial peduncle of the ilium, which fits into a deep socket on the ischium (e.g.., *Alioramus*, *Gallimimus*: Brusatte et al. 2012a). Because it is often difficult to determine the presence or absence of this subtle peg-like process, we here consider the peg-like process and a peg-like ischial peduncle itself as primarily homologous. The presence of either of these processes can be clearly determined by the presence of a funnel-like socket on the iliac peduncle of the ischium. Because both structures leave a similar mark on the ischium, this supports the hypothesis of their primary homology (at least at some level).

**Character 749: Scapula, acromion process, shape:**

0: much deeper dorsoventrally than long anteroposteriorly, generally tapering or triangular in shape

1: approximately as long as or longer anteroposteriorly than deep dorsoventrally, with short reach beyond scapular blade and squared-off profile

**Character 750: Basisphenoid, pronounced muscle scars flanking basisphenoid recess:**

0: absent

1: present

**Character 751: Ilium, form of distal articular surface of pubic peduncle:**

0: convex

1: flat or concave (notched)

***Characters Relevant to Alvarezsauroidea (from Choiniere et al. 2010a,b, 2012 and Choiniere, pers. comm.):**

**Character 752: Lacrimal, orientation of ventral ramus relative to the long axis of the alveolar margin of the upper jaw when the articulated skull is seen in lateral view**

0: approximately vertical or inclinded slightly anteroventrally

1: inclined strongly posteroventrally

**Character 753: Exoccipital-opisthotic, position of ventral edge of the base of the paroccipital process:**

0: level with or dorsal to the dorsal border of the occipital condyle

1: situated at mid-height of occipital condyle or further ventrally

**Character 754: Maxillary teeth, shape: (UNORDERED)**

0: mediolaterally thin and recurved

1: lanceolate (as in therizinosauroids)

2: conical (as in alvarezsauroids)

**Character 755: Dorsal vertebrae, form of opisthocoely in taxa with opisthocoelous dorsals:**

0: some dorsal vertebrae opisthocoelous

1: most or all dorsal vertebrae opisthocoelous

Note: Only those taxa with strongly opisthocoelous dorsal vertebrae (character 99) are scored for an affirmative score here. All other taxa are scored as inapplicable (“?”).

**Character 756: Dorsal vertebrae, shape of neural spine in posterior dorsals:**

0: approximately square-shaped or slightly rectangular, with a dorsoventral height that is equal to or slightly greater than the anteroposterior length at the base

1: rectangular, much higher dorsoventrally than long anteroposteriorly at the base

**Character 757: Dorsal vertebrae, position of parapophysis in posterior dorsals:**

0: distinctly ventral to transverse process

1: at approximately same dorsoventral level as transverse process

**Character 758: Caudal vertebrae, morphology of anterior caudals:**

0: amphiplatyan

1: procoelous

**Character 759: Caudal vertebrae, position of transverse process on anterior caudals:**

0: approximately centered (in the anteroposterior dimension) on the centrum

1: anteriorly displaced

**Character 760: Coracoid, biceps tubercle:**

0: absent or subtle

1: present as a discrete, mound-like structure

**Character 761: Coracoid, strong lateral ridge on lateral surface, extending posteriorly from biceps tubercle along posteroventral process:**

0: absent

1: present

**Character 762: Scapula, tubercle on posterior surface of bone dorsal to glenoid:**

0: absent

1: present

**Character 763: Humerus, shape of internal tuberosity in anterior view: (ORDERED)**

0: triangular or rounded, not discretely separated from remainder of humerus

1: rectangular, separated from the humeral head by a small but distinct notch

2: rectangular and hypertrophied, separated from the humeral head by a large notch

Note: This character is modified into an ordered multistate with the addition of a second derived state, to distinguish between the condition in some therizinosauroids in which the internal tuberosity is rectangular and offset by a small notch (*Falcarius*, *Erliansaurus*) and that in which the internal tuberosity is rectangular but also proportionally hypertrophied and set off by a larger notch (*Segnosaurus*, *Erlikosaurus*, *Neimongosaurus*, *Suzhousaurus*, *Nothronychus*). This distinction follows Zanno et al. (2009, character ZCD 285).

**Character 764: Ulna, shape of olecranon process:**

0: transversely broad

1: mediolaterally thin and blade-like

**Character 765: Ulna, position of distal articular surface:**

0: limited to distal end

1: trochlear articular surface extends onto dorsal surface of ulna, bulbous in shape

**Character 766: Metacarpal III, form of proximal articular surface:**

0: flat or slightly convex

1: deeply concave and cup-like

**Character 767: Metacarpal III, length:**

0: considerably longer than length of metacarpal I

1: approximately same length as, or slightly longer than, metacarpal I

**Character 768: Manual digits, paired flexor processes on proximal portion of ventral surfaces of proximal-most phalanges:**

0: absent

1: present

**Character 769: Manus, shape of proximal articular surface of ungual of first digit:**

0: ovoid, dorsoventrally taller than mediolaterally wide

1: approximately square shaped, as mediolaterally wide as dorsoventrally tall

**Character 770: Manus, form of lateral groove on ungual of first digit:**

0: unenclosed or partially enclosed proximally by small flange

1: proximal end of grooves passes through foramena on ventral surface of ungual

**Character 771: Femur, interaction of head and greater trochanter:**

0: confluent

1: separated by a cleft

**Character 772: Femur, shape of lateral distal condyle:**

0: distally rounded

1: distally conical, projecting substantially further distally than medial condyle

**Character 773: Tibia, shape of medial posterior condyle of proximal end in proximal view:**

0: posteriorly rounded

1: posteriorly conical, projecting substantially further posteriorly than lateral condyle

**Character 774: Fibula, orientation of proximal margin (as seen in lateral view):**

0: horizontal or nearly horizontal

1: anterior portion of proximal margin extends substantially further proximally than posterior portion

Note: This character is equivalent to Zanno et al. (2009: character ZCD 329).

**Character 775: Fibula, shape of proximal surface in proximal view:**

0: anterior and posterior portions of surface with nearly equal mediolateral widths

1: anterior portion markedly wider mediolaterally than posterior portion

Note: This character is equivalent to Zanno et al. (2009: character ZCD 331).

**Character 776: Astragalus, fossa on anterior surface of lateral portion of base of ascending process, sometimes bearing accessory fenestrations:**

0: absent

1: present

**Character 777: Astragalus, horizontal groove on proximal portion of anterior surface of condyles, separating condyles from ascending process:**

0: present

1: absent

**Character 778: Pes, phalanges of pedal digit IV, shape:**

0: anteroposteriorly long, proximal and distal articular surfaces well separated

1: anteroposteriorly short, with proximal and distal articular surfaces very close together, particularly in distal elements

***Characters Relevant to Therizinosauroidea (from Zanno et al. 2009 and Zanno 2010):**

**Character 779: Basisphenoid, inflated basisphenoidal bulla:**

0: absent

1: present

**Character 780: Braincase, foramen magnum, size (area): (ORDERED)**

0: smaller than size of occipital condyle

1: approximately equal in size with occipital condyle

2: larger than size of occipital condyle

**Character 781: Ectopterygoid, position:**

0: posterior to palatine

1: lateral to palatine

**Character 782: Dentary, mediolateral width of symphyseal region of conjoined dentaries:**

0: narrower than width of post-symphyseal region

1: broader than width of post-symphyseal region

**Character 783: Premaxillary teeth, serrations:**

0: present

1: absent

Note: It has been shown that unserrated premaxillary teeth are present in some juvenile tyrannosaurids, but serrations are gained during ontogeny (Carr and Williamson 2004). Taxa in which this ontogenetic shift occurs are scored for the present condition (state 0). Any taxa with any trace of serrations on any premaxillary tooth (whether all teeth are serrated or not) are also scored for the present condition. Only those taxa in which all known premaxillary teeth are lacking serrations are scored for the absent condition (state 1). It is likely that there is positional variation in serration presence/absence along the premaxillary tooth row, but this is difficult to parse out because complete dentitions are not known for most taxa. We recognize that future authors may wish to divide this character into additional states referring to serration presence/absence on individual premaxillary teeth or regions of the premaxillary dentition.

**Character 784: Dentary teeth, shape of mesial (anterior) teeth:**

0: not conical (i.e., ziphodont or lanceolate)

1: conical

Note: Although the first one or two dentary teeth of derived tyrannosauroids such as *Alioramus* and *Tyrannosaurus* are small and somewhat conical (in comparison to the remainder of the dentary tooth row), these taxa are here scored for state 0. State 1 refers to taxa in which the first several teeth of the dentary (at least the first four, where visible) are distinctly conical compared to the more posterior (distal) dentary teeth, but are of approximately the same size as the more distal premaxillary teeth. Oftentimes this condition is referred to in the literature as “anterior dentary teeth that are similar to the premaxillary teeth in shape”.

**Character 785: Dentary, extent of tooth row:**

0: teeth present at anterior (mesial) tip of dentary

1: teeth absent at anterior dentary but present further posteriorly (distally)

Note: This character encapsulates Zanno et al.’s (2009) revision of the original TWiG character number 220, which is character number 217 in the current analysis. We retain character 217 in the original language of Turner et al. (2012) here, as it refers to an ordered progression of fully toothed-posteriorly edentulous-completely edentulous, and therefore the addition of a fourth state for an anteriorly edentulous dentary (which is unique to some derived therizinosauroids) would disrupt this ordered sequence. Our preference is to include the therizinosauroid condition as a separate character, with those taxa that are completely edentulous scored as inapplicable (“?”).

**Character 786: Cervical vertebrae, length of anterior centra:**

0: up to three times longer anteroposteriorly than the minimum transverse centrum width in ventral view (or the height of the anterior articular surface in lateral view, if the centrum cannot be viewed ventrally)

1: hyperelongate, approximately five times longer than wide

Note: Zanno et al. (2009) presented a three-state character, but we do not want to over divide a ratio character such as this, and therefore recognize the major distinction among coelurosaurs as between a centrum that is fairly anteroposteriorly short (somewhere between 1-3 times longer than wide) and the so-called “hyperelongate” condition in which the centrum is approximately five times longer than wide. Those taxa that do not preserve anterior cervical, but have middle cervical that are hyperelongate, are scored for state 1.

**Character 787: Cervical vertebrae, morphology of ventral surface of centrum:**

0: smoothly flat or convex

1: with distinct depression anteriorly (at the level of the parapophyses)

**Character 788: Cervical vertebrae, posterolateral margins of ventral surface of centrum, form:**

0: unpronounced

1: developed into prominent crests (ventrally projecting “fins”)

Note: Zanno (2010) notes that less pronounced crests, potentially homologous to state 1, are present on the cervical vertebrae of some ornithomimosaurs, but Zanno et al. (2009) scores all score-able ornithomimosaurs for state 0. We agree that ornithomimosaurs should be scored for state 0, as taxa such as *Gallimimus* (ZPAL MgD/I-94) clearly do not possess the prominent “fin-like” crests of therizinosauroids and some basal oviraptorosaurs.

**Character 789: Dorsal vertebrae, anterior dorsals, dorsoventral depth of neural arch compared to that of centrum:**

0: depth of region between prezygadiapophyseal lamina and base of neural spine less than or equal to depth of anterior and/or posterior articular surface of centrum

1: depth of region between prezygadiapophyseal lamina and base of neural spine greater than depth of anterior and/or posterior articular surface of centrum (neural arch hypaxially inflated relative to centrum)

**Character 790: Dorsal vertebrae, size of parapophyses on anterior dorsals:**

0: moderate in size, articular facet of parapophysis less than half dorsoventral depth of anterior articular surface of centrum

1: hypertrophied, articular facet greater than two thirds of the dorsoventral depth of the anterior articular surface of centrum

**Character 791: Caudal vertebrae, pneumatic foramina on the centra of anterior caudals:**

0: absent

1: present

**Character 792: Scapula, dorsal flange on dorsal margin of scapular blade:**

0: absent

1: present

**Character 793: Humerus, crest on posteromedial surface of humeral shaft:**

0: absent

1: present

**Character 794: Humerus, tuberosity on anterior surface of distal humerus, proximal to entepicondyle:**

0: absent

1: present

**Character 795: Humerus, entepicondyle:**

0: present and prominent as a spherical, conical, or crest-like structure in cranial view

1: extremely reduced or absent

Note: This is a modified version of Zanno et al. (2009: character ZCD 291). We find it difficult to distinguish between Zanno et al.’s discrete conditions of “spherical” and “crest-like,” which are given separate states in the original analysis. Therefore, we combine these conditions into a single “present and prominent” state, and recognize the major difference between coelurosaurs as the presence or absence of a prominent entepicondyle. Taxa with a prominent entepicondyle usually, if not always, exhibit a prominent groove proximal to the entepicondyle, whereas those that have a reduced or absent condyle do not. Therefore, we do not utilize Zanno et al.’s (2009: character oZCD 292) separate character for the groove.

**Character 796: Humerus, mediolateral width of distal end:**

0: greater than 2x minimum width of shaft

1: less than 2x minimum width of shaft

Note: This is a modified version of Zanno et al. (2009: character oZCD 293), which combines two of the original states (“moderately expanded” and “significantly expanded”) into a single state of “greater than 2x minimum width of shaft. Therefore, the character as worded here distinguishes the aberrantly unexpanded distal humeri of ornithomimosaurs and some tyrannosauroids from the standard condition among theropods.

**Character 797: Humerus, morphology of distal humerus in anterior view:**

0: medial aspect of distal humerus unexpanded, entepicondyle situated proximal to ulnar condyle

1: medial aspect of distal humerus expanded and subtriangular in anterior view, entepicondyle located well medial to ulnar condyle

**Character 798: Metacarpal I, rectangular buttress on ventrolateral aspect of proximal surface that underlies ventromedial surface of metacarpal II:**

0: absent

1: present

**Character 799: Manus, ratio of the proximodistal length of metacarpal II to that of the combined proximodistal lengths of phalanges II-1 and II-2:**

0: less than or equal to 1.0

1: greater than 1.0 (i.e., metacarpal II longer than combined lengths of phalanges II-1 and II-2)

**Character 800: Manus, ligament pits on manual phalanges:**

0: strongly developed

1: weakly developed or absent

**Character 801: Manus, manual unguals, proximodistal length:**

0: shorter to, equal to, or slightly longer than length of penultimate phalanx

1: elongate, twice or more as long as penultimate phalanx

**Character 802: Ilium, orientation of ventral portion of preacetabular process:**

0: parasagittal, in line with dorsal portion of process

1: laterally deflected, extends nearly perpendicular from sagittal plane of ilium

**Character 803: Ilium, size of preacetabular process:**

0: moderately developed, anteroposterior length of process subequal with dorsoventral height of ilium directly dorsal to the center of the acetabulum

1: hyperelongate, length of process at least twice the height of ilium above acetabulum

**Character 804: Ilium, orientation of blade:**

0: parallel or gently inclined relative to dorsoventral plane passing through the pubic and ischial peduncles (the ventral portion of the ilium)

1: angled laterally relative to the ventral portion of the ilium, rising steeply at at least a 30 degree angle from the dorsoventral plane passing through the peduncles

**Character 805: Ilium, morphology of dorsal surface of postacetabular process in dorsal view: (ORDERED)**

0: smooth and non-rugose

1: with rugosity causing transverse expansion of the posterior dorsal margin

2: hyperrugose, with hypertrophied posterior tuberosity

**Character 806: Ilium, orientation of pubic peduncle: (ORDERED)**

0: straight

1: posteriorly recurved, articular face posteroventrally directed

2: posteriorly recurved, articular face posteriorly directed

**Character 807: Ilium, morphology of antitrochanter: (ORDERED)**

0: separated from ischial peduncle

1: merged with ischial peduncle, both structures together form enlarged ventrolaterally flattened boss

2: merged with ischial peduncle as a boss, which is hypertrophied and spherical

**Character 808: Ischium, position of distally placed dorsal process along posterior edge of shaft: (UNORDERED)**

0: entirely proximal to obturator process

1: opposite obturator process

2: extending distal to obturator process

Note: This character refers to the position of the dorsal process of the ischium, whose presence or absence is scored in character 226. Those taxa without a process are scored as inapplicable (“?”).

**Character 809: Ischium, shape of obturator process in those taxa in which the process contacts the pubis:**

0: approximately square-shaped

1: elongate anteroposteriorly, approximately twice as long as dorsoventrally deep

Note: The presence/absence of an obturator process contacting the pubis is scored in character 164. Those taxa scored for the absence of this feature in character 164 are scored as inapplicable (“?”) for the current character.

**Character 810: Ischium and pubis, morphology of contact surfaces between the two bones:**

0: flat

1: markedly sinuous

**Character 811: Pubis, shape of shaft:**

0: rod-like

1: mediolaterally flattened

**Character 812: Pubis, morphology of distal shaft:**

0: distal portion of shaft approximately equal (or less than) in anteroposterior length to proximal portion of shaft

1: distal portion of shaft greatly enlarged, more than twice the anteroposterior length of the proximal portion of shaft

**Character 813: Femur, anteroposterior width of region bridging femoral head and greater trochanter in proximal view:**

0: smoothly confluent with head and greater trochanter

1: anteroposteriorly constricted relative to head and greater trochanter

**Character 814: Femur, hook-like process at distal edge of femoral head, demarcating a notch between the head and the shaft**

0: present

1: absent, shaft and head smoothly confluent

Note: This is equivalent to Zanno et al. (2009: character oZCD 323), which refers to the presence or absence of a “raised ventral rim” that separates the femoral head from the femoral neck (essentially the shaft). The “ventral rim” is equivalent to the hook-like process of the femoral head, which extends further distally relative to the remainder of the head to demarcate a notch between the head and shaft in anterior and posterior views.

**Character 815: Tibia, morphology of incisura tibialis (lateral fossa on the tibia in proximal view):**

0: deeply inset

1: wide and shallow, nearly absent

**Character 816: Tibia, shape of tibia in proximal view:**

0: anteroposteriorly longer than mediolaterally wide (measurements taken through midpoint of proximal surface)

1: mediolaterally wider than anteroposteriorly long

**Character 817: Tibia, length of fibular crest:**

0: short and proximally positioned, extends up to approximately 1/3 of the length of the tibia

1: long and distally extensive, extends to approximately the midshaft of the tibia

**Character 818: Tibia, exposure and morphology of the anteromedial region of the distal tibia: (ORDERED)**

0: covered anteriorly by the astragalus

1: not covered anteriorly by the astragalus, exposed

2: exposed and developed into an anterior tuberosity

**Character 819: Fibula, iliofibularis tubercle, position:**

0: proximal to midshaft of fibula

1: approximately at midshaft of fibula

Note: In those alvarezsauroids with an apomorphically shortened fibula (e.g., *Shuvuuia*, *Xixianykus*, *Parvicursor*), the position of the tubercle is measured relative to the length of the shaft of the tibiotarsus. Therefore, these taxa are scored for state 0 although their tubercles are located near the distal end of the shortened fibula.

**Character 820: Astragalus, ascending process position:**

0: lateral edge terminates approximately at the lateral edge of the tibia, or medial to the lateral edge of the tibia

1: lateral edge extends lateral to the tibial shaft to contact and overlap the fibula

**Character 821: Astragalus, lateral condyle of distal end, morphology:**

0: well developed

1: strongly reduced, lateral tibia exposed on distal and anterior surfaces of tibiotarsal region when tibia and astragalus are in articulation

**Character 822: Metatarsus, overall shape:**

0: elongate, unit comprised of metatarsals II-IV much longer proximodistally than wide mediolaterally

1: short and broad, unit comprised of metatarsals II-IV less than twice as long as wide

**Character 823: Metatarsus, proximodistal length: (ORDERED)**

0: greater than 45% length of tibia

1: between 44-38% length of tibia

2: less than 36% length of tibia

Note: For those taxa in which only isolated metatarsals are present (i.e., not a complete metatarsus), the length of metatarsals II, III, or IV is taken as a proxy for the length of the metatarsus as a whole.

**Character 824: Metatarsus, orientation of individual metatarsals II-IV:**

0: not closely appressed, divergent from each other

1: appressed throughout most of metatarsus

Note: The outgroups *Allosaurus* and *Sinraptor* are scored for state 0, as in these taxa metatarsals II and III are closely appressed but metatarsal IV is divergent. *Zuolong* has closely appressed metatarsals II and III, but because only a fragment of metatarsal IV is known it cannot be determined if IV was appressed or divergent, and therefore this taxon is scored as “?”. Within coelurosaurs, all therizinosauroids are scored for a divergent metatarsus, following Zanno et al. (2009: character ZCD 335). Taxa without a complete metatarsus can be scored based on the shape of metatarsal IV (a laterally divergent kink along the shaft is indicative of state 0, whereas a straight shaft is indicative of state 1).

**Character 825: Pes, pedal unguals of digits III-IV, proximodistal length:**

0: approximately the same length as the penultimate phalanx (ungual may be slightly shorter or longer than the penultimate phalanx)

1: twice as long or more than the penultimate phalanx

***New Characters Relevant to Basal Coelurosaurs Established in this Dissertation (Note that some are inspired by published literature, as described in the main text, and others may have been added to published analyses during the time this dissertation was written).**

**Character 826: Premaxilla, anteroposterior length compared to that of maxilla:**

0: length of ventral (alveolar) margin greater than 10% total anteroposterior length of maxilla

1: length of ventral (alveolar) margin less than 10% total anteroposterior length of maxilla

**Character 827: Supraoccipital, tab-like processes on left and right sides of dorsal margin of bone:**

0: absent

1: present

Note: This character has been included in previous phylogenetic analyses of tyrannosauroid interrelationships (e.g., Carr and Williamson 2010), but Brusatte et al. (2010a) combined this and the absence/presense of a dorsal bifurcation of the supraoccipital into a single character, due to a presumed equivalency between the characters. As outlined by Bever et al. (2013), however, the tabs are separate from the bifurcation, as many tyrannosauroids have tabs but only a few taxa exhibit a bifurcation. The tabs themselves are created by midline depression of supraoccipital relative to left and right sides, as explained by Bever et al. (2013).

**Character 828: Nasal, premaxillary processes (=supranarial processes), extent of their apposition to each other on the midline in dorsal view:**

0: apposed for nearly their entire length (may abruptly separate from each other at their tips)

1: not apposed for most of their length, and therefore do not abruptly separate from each other at their tips

**Character 829: Dentary, Meckelian groove, position:**

0: approximately centered at dorsoventral midheight of dentary

1: positioned ventrally, located closer to the ventral margin than the dorsal margin

Note: This character was originally used by Carr and Williamson (2010: character 211), in which state 0 was scored for all score-able tyrannosauroids. We here note that this condition is also present in outgroups, *Compsognathus*, and derived therizinosauroids (*Erlikosaurus*), whereas state 1 is present in *Ornitholestes*, ornithomimosaurs (*Harpymimus*), basal therizinosauroids (*Falcarius*, *Beipiaosaurus*, *Alxasaurus*), and paravians (e.g.., *Deinonychus*, *Zanabazar*).

**Character 830: Dentary, row of foramina on lateral surface paralleling ventral margin:**

0: absent or limited to a small series of foramina at the anterior end of the dentary (at the level of the first 1-4 alveoli)

1: present as a distinct row that extends along most of the lateral surface of the dentary (across the entire length of the tooth row at the very least)

**Character 831: Splenial, notch along dorsal margin of anterior process, where the splenial contacts the supradentary (if present):**

0: absent

1: present

**Character 832: Cervical vertebrae, position of prezygapophyses in anterior-middle cervicals:**

0: prezygapophyseal facet approximately level with anterior face of centrum

1: prezygapophyseal facet entirely anterior to anterior face of centrum

**Character 833: Caudal vertebrae, morphology of dorsal portion of neural spines of anterior caudals:**

0: unexpanded

1: expanded anteroposteriorly relative to the remainder of the neural spine (and often mediolaterally as well)

**Character 834: Ulna, morphology of distal end:**

0: expanded mediolaterally at least 1.5x relative to midshaft mediolateral width

1: expanded less than 1.5x midshaft mediolateral width

Note: The denoted measurements are taken in anterior (extensor) and posteror (flexor) views.

**Character 835: Ilium, medial surface of preacetabular process, pronounced horizontal shelf continuing from anterior margin of pubic peduncle to demarcate the cuppedicus fossa dorsally:**

0: absent

1: present

**Character 836: Metatarsal III, mediolateral width of distal end compared to mediolateral widths of distal ends of metatarsals II or IV (whichever of the latter is greater):**

0: less than 1.3 times as wide

1: greater than 1.3 times as wide

**Character 837: Lacrimal, extent of antorbital fossa on region where anterior and ventral rami meet:**

0: extensive, fossa excavates nearly entire region and nearly extends to the posterodorsal corner of the lacrimal (the lacrimal angle), leaving only a thin region of bone at the posterodorsal corner of the lacrimal

1: reduced, fossa stops well short of the posterodorsal corner of the lacrimal

**Character 838: Scapula and coracoid, deep fossa on lateral surfaces of both bones in the region of their suture (covering the posterior half of the coracoid and anterior portion of the acromion plate region of the scapula):**

0: present

1: absent

**Character 839: Scapula and coracoid, contribution of each bone to the glenoid: (UNORDERED)**

0: both bones contribute approximately equally in the anteroposterior dimension

1: scapula contribution markedly anteroposteriorly longer than coracoid contribution

2: coracoid contribution markedly anteroposteriorly longer than scapula contribution

**Character 840: Femur, shape of anterior margin in proximal view:**

0: strongly convex, due to a midline tubercle

1: essentially straight or slightly concave

**Character 841: Femur, horizontal ridge on the anterior surface of the head and neck, demarcating a deep fossa ventrally:**

0: absent

1: present

**Character 842: Femur, extent of crista tibiofibularis in distal view: (ORDERED)**

0: projects further posteriorly than medial condyle

1: extends to the same approximate posterior level as medial condyle

2: terminates well short of the posterior level of the medial condyle (medial condyle projects substantially further posteriorly)

**Character 843: Tibia, position of medial ridge on posterior surface of distal end: (ORDERED)**

0: displaced laterally, positioned lateral to the medial edge of the distal tibia by approximately 25-33% of the mediolateral width of the distal tibia

1: positioned lateral to the medial edge of the distal tibia approximately 10-20% of the mediolateral width of the distal tibia

2: positioned medially, positioned at approximately the posteromedial corner of the distal tibia in distal view

**Character 844: Scapula, fossa on lateral surface of bone immediately above glenoid, which is demarcated dorsally by a convex bulge or ridge spanning the scapula-coracoid suture:**

0: absent

1: present

**Character 845: Humerus, deltopectoral crest, orientation relative to mediolateral long axis of proximal end of humerus in proximal view:**

0: straight, approximately perpendicular to long axis of proximal end of humerus

1: curves strongly medially as it continues anteriorly, such that anterior end of crest is oblique to long axis of proximal end of humerus

Note: Those taxa scored for state 0 for character 358 (a deltopectoral crest that is projected laterally or dorsally, such that it is in line with the long axis of proximal end of the humerus) are here scored as inapplicable (“?”).

**Character 846: Femur, position of fourth trochanter: (UNORDERED)**

0: along posteromedial corner of shaft along its entire length

1: positioned near center of posterior surface of shaft distally and extending proximomedially to become confluent with posteromedial corner of shaft proximally

2: positioned near center of posterior surface of shaft distally and extending proximolaterally to bcome confluent with the greater trochanter

**Character 847: Fibula, anteroposterior width of the minimum point of the midshaft compared to the maximum anteroposterior width of the proximal end:**

0: 20% or greater

1: less than 18%, fibula shaft exceptionally gracile

**Character 848: Maxillary teeth, posterior (distal) extent of tooth row:**

0: extensive, extends posterior to the level of the maxillary ascending ramus and underneath the antorbital fenestra (if present)

1: limited, terminates posteriorly at the level of the maxillary ascending ramus

**Character 849: Dentary, shape of ventral margin in lateral view:**

0: approximately straight or slightly convex

1: broadly concave

**Character 850: Maxilla, posterior extent of the ascending ramus relative to that of the main body (jugal ramus):**

0: the two rami extend to approximately the same level posteriorly, distance between posterior tip of ascending ramus and posterior tip of main body no more than 1/3 of the anteroposterior length of the entire maxilla

1: main body extends considerably further posteriorly relative to the ascending ramus, distance between the posterior tips of the two rami is greater than 1/3 of the length of the maxilla

**Character 851: Tibia, proximal surface, proximal extent of cnemial crest relative to the proximal extent of the posterior condyles:**

0: cnemial crest extends further proximally than condyles

1: cnemial crest and condyles extend to same approximate level proximally

**Character 852: Prootic, prominent fossa on the lateral surface of the bone, anterior to the otic recess and posterior to the preotic pendant, that houses the external foramina of the trigeminal and facial nerves and pneumatic openings:**

0: absent

1: present

**Character 853: Palatine, form of pneumaticity in those taxa with pneumatic palatines:**

0: pneumatic fossa on the external surface of the bone

1: large internal chamber that opens externally via a window-like pneumatic opening
